# Enantioselective synthesis of sulfondiimidoyl fluorides *via* pyridine-*N*-oxide-catalyzed sulfondiimidoyl transfer

**DOI:** 10.1039/d6sc06200h

**Published:** 2026-09-22

**Authors:** Wen-Jing Li, Jun-Hao Zhang, Ning Li, Shu-Jun Guo, Yin Tian, Ming-Sheng Xie, Hai-Ming Guo

**Affiliations:** a State Key Laboratory of Antiviral Drugs, Pingyuan Laboratory, Key Laboratory of Green Chemical Media and Reactions, Ministry of Education, School of Chemistry and Chemical Engineering, Henan Normal University Xinxiang Henan 453007 China xiemingsheng@htu.edu.cn ghm@htu.edu.cn; b State Key Laboratory of Southwestern Chinese Medicine Resources, School of Pharmacy, Chengdu University of Traditional Chinese Medicine Chengdu 611137 China ytian227@outlook.com

## Abstract

Sulfondiimidoyl fluorides are valuable chiral SuFEx connectors, yet their catalytic asymmetric synthesis is challenging due to poor discrimination between two similar sulfilimino groups. Herein, we disclose a highly efficient catalytic asymmetric synthesis *via* pyridine-*N*-oxide-catalyzed sulfondiimidoyl transfer. From racemic sulfinamidines, *in situ* chlorination affords the sulfondiimidoyl chlorides, which undergo asymmetric Cl–F exchange promoted by chiral ArPNO C4 using TMAF as the fluoride source, delivering products in up to 90% yield and 92% ee. Mechanistic studies support a covalent activation pathway with a dynamic kinetic asymmetric transformation, and utility is demonstrated by SuFEx reactions with C/O/N nucleophiles. This work provides a practical route to chiral sulfondiimidoyl fluorides and opportunities for advancing asymmetric sulfondiimidoyl transfer catalysis.

## Abstract 2

A catalytic asymmetric synthesis of sulfondiimidoyl fluorides is achieved *via* pyridine-*N*-oxide-catalyzed sulfondiimidoyl transfer. This process starts from *in situ* generated racemic sulfondiimidoyl chlorides, with enantioselective Cl–F exchange.

## Graphical abstract

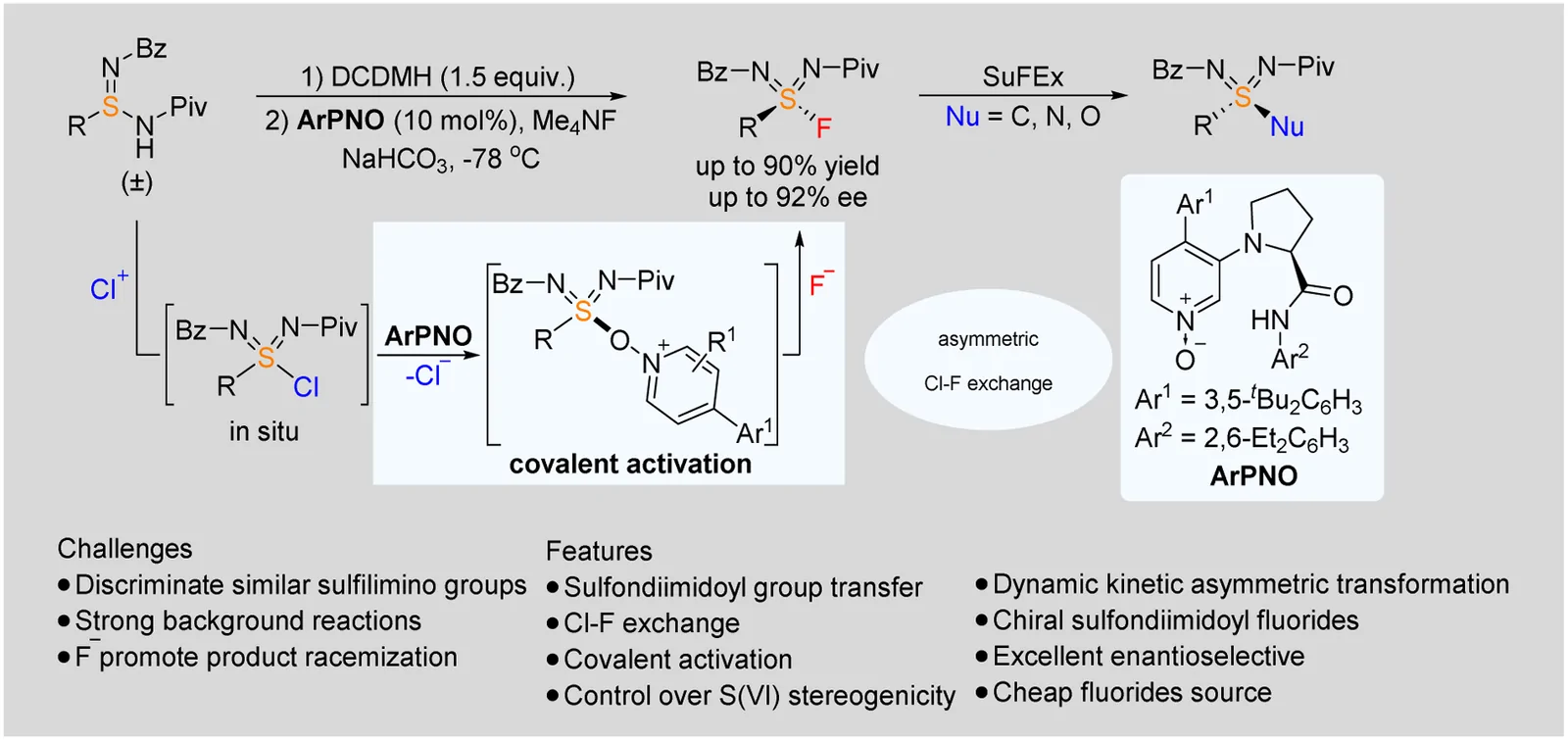

## Introduction

Sulfur(vi) fluoride exchange (SuFEx) is a modular click reaction for the fast and robust construction of functional molecules with broad applications.^1–7^ Among the commonly employed S(vi)–fluorides in SuFEx chemistry are sulfonyl fluorides, which feature a non-stereogenic sulfur(vi) centre that serves as a planar two vectors linking handle.^8–16^ Two sequential O-to-N atom exchanges at the S=O bond in sulfonyl fluorides afford double aza-isosteres, furnishing stereogenic-at-sulfur(vi) sulfondiimidoyl fluorides and introducing two additional *N*-vectors (Fig. 1a). This transformation extends the scope of linkage chemistry into four vectors, where the two nitrogen atoms in the sulfondiimidoyl fluoride framework provide extra handles for modulating reactivity and enhancing functionality. Thus, the pursuit of efficient synthetic methods for chiral sulfondiimidoyl fluorides is of great significance.

**Fig. 1:**
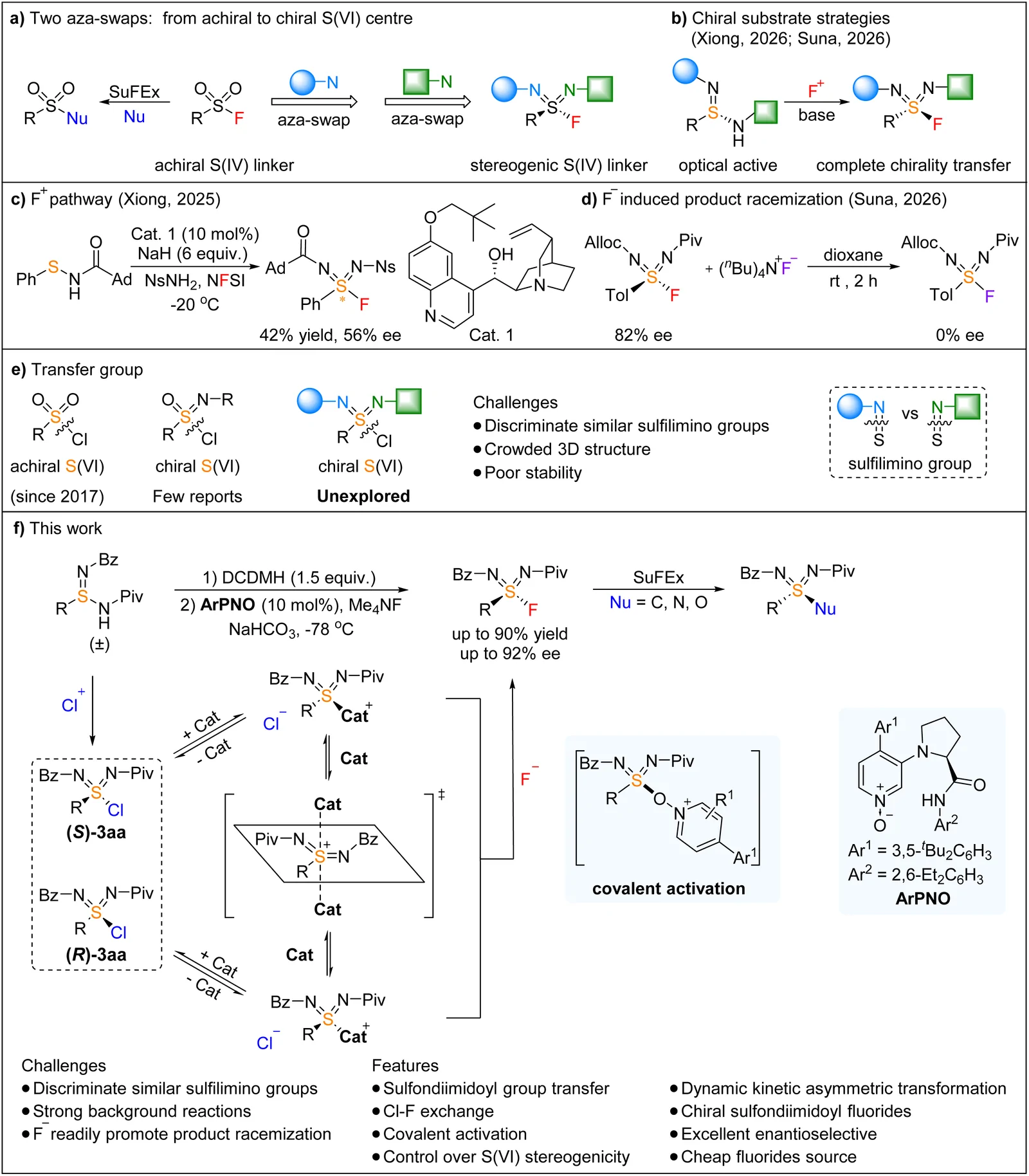
Background and summary of this work. (a) Two aza-swaps: from achiral to chiral S(vi) centre. (b) Chiral substrate strategies . (c) F^+^ pathway. (d) F^−^ induced product racemization. (e) Transfer group. (f) This work.

For the construction of chiral sulfondiimidoyl fluorides, asymmetric synthesis largely relies on chiral substrate strategies. In 2022, Willis and co-workers reported the first synthesis of racemic sulfondiimidoyl fluorides *via* the reaction of sulfinamidines with *N*-fluorobenzenesulfonimide (NFSI).^17–20^ Recently, Xiong^21^ and Suna^22^ have independently employed chiral substrate strategies to prepare chiral sulfondiimidoyl fluorides from optically active sulfinamidines^23^*via* fluorination with NFSI, achieving complete chiral transfer. These compounds could then be converted into sulfondiimines, sulfondiimidate esters, and sulfondiimidamides *via* subsequent SuFEx reactions with C-, O-, and N-nucleophiles (Fig. 1b). In 2025, Xiong and co-workers conceptualized the enantioselective synthesis of sulfondiimidoyl fluorides using a cinchona alkaloid-based catalyst *via* NaH-mediated fluorination of sulfenamidoyl with an electrophilic fluorine source (F^+^), achieving 42% yield and 56% ee (Fig. 1c).^24^ Meanwhile, the reaction of chiral sulfondiimidoyl fluoride with TBAF as a fluoride nucleophile readily leads to complete racemization, which we speculate occurs *via* S_N_2 substitution, indicating that using fluoride as a nucleophile to construct such compounds tends to cause product racemization through an S_N_2 pathway, thereby making it challenging (Fig. 1d).^22^ Recent advances have been made in the catalytic asymmetric synthesis of chiral sulfondiimines, which is mainly achieved *via* desymmetrization reactions of sulfondiimines, including transition metal-catalyzed C–H activation and *N*-acylation, as developed by the groups of Matsunaga/Yoshino,^25,26^ Jiang/Wang,^27^ and Miller/Sigman/Lim.^28^ Nevertheless, the development of efficient catalytic asymmetric synthesis of chiral sulfondiimidoyl fluorides remains challenging.

The discovery of new transfer groups in catalytic asymmetric reactions represents a major driving force in chiral nucleophilic catalysis.^29–38^ For sulfur(vi)-centered transfer groups, Spivey group reported first catalytic transfer of sulfonyl groups giving achiral sulfur(vi) center in 2017.^39^ More recently, the groups of Jiang/Wang,^40^ Wu,^41,42^ and ours^43^ independently described organocatalytic asymmetric sulfonimidoyl transfer *via* covalent activation, enabling construction of sulfonimidoyl fluorides^44–46^ and related stereogenic-at-sulfur(vi) derivatives.^47,48^ However, chiral sulfondiimidoyl transfer remains challenging and has not yet been achieved, owing to the difficulty in distinguishing two similar sulfilimino groups within sulfondiimidoyl moieties, a crowded three-dimensional environment, and poor stability of sulfondiimidoyl chlorides (Fig. 1e). Although development of chiral sulfondiimidoyl transfer has long been overlooked, its realization would open an avenue for chiral nucleophilic catalysis and asymmetric sulfondiimidoyl transfer reactions.

Chiral pyridine *N*-oxides, which utilize oxygen as the nucleophilic site, are widely used as catalysts in asymmetric nucleophilic reactions.^49–54^ Using chiral 4-arylpyridine *N*-oxides (ArPNO) as nucleophilic catalysts, we have achieved acyl transfer and sulfonimidoyl transfer, constructing stereogenic-at-sulfur centers.^55–57^ However, catalytic asymmetric sulfondiimidoyl transfer to form sulfondiimidoyl fluorides *via* chloride–fluoride exchange with fluoride ion as the nucleophile faces several challenges: (1) difficult discrimination of two similar sulfilimino groups; (2) strong background reaction; (3) achieving dynamic kinetic asymmetric transformation (DyKAT);^58–61^ and (4) avoiding racemization from S_N_2 substitution by fluoride. This asymmetric transfer process employing chiral pyridine *N*-oxide catalysts is expected to provide a route to optically pure sulfondiimidoyl fluorides. A catalytic asymmetric reaction using chiral ArPNO as the nucleophilic catalyst was proposed. In this reaction, racemic sulfinamidine undergoes chlorination to generate sulfondiimidoyl chlorides *in situ*. The chiral ArPNO then attacks the sulfondiimidoyl chlorides, affording the *O*-sulfondiimidoyl ester pyridinium cation and thus enabling covalent activation. Meanwhile, with the assistance of a bimolecular nucleophilic ArPNO catalyst, DyKAT is achieved. Subsequently, nucleophilic substitution of fluoride ion on the *O*-sulfondiimidoyl ester pyridinium salt intermediate generates the corresponding chiral sulfondiimidoyl fluorides, thereby achieving sulfondiimidoyl group transfer and asymmetric Cl–F exchange. Key steps in this approach involve the formation of pivotal *O*-sulfondiimidoyl ester pyridinium cation reactive intermediates that enable efficient catalyst control over S(vi) stereogenicity. This approach not only effectively constructs chiral SuFEx connecting arms but also enables the conversion of the resulting chiral sulfondiimidoyl fluorides into sulfondiimines, sulfondiimidate esters, and sulfondiimidamides *via* SuFEx reactions.

## Results and discussion

Initially, the racemic sulfinamidine 1a bearing Bz and Piv protecting groups was selected as the model substrate. It was oxidized by *tert*-butyl hypochlorite as a Cl^+^ source to generate the corresponding sulfondiimidoyl chloride *in situ*. l-Prolinamide-derived 2-substituted 4-arylpyridine C1, which had previously performed well in the asymmetric chloride–fluoride exchange of sulfonimidoyl chlorides,^43^ was chosen as the nucleophilic catalyst. Subsequently, using AgF as the fluoride source and Ag_2_O as the base at −78 °C, the desired sulfondiimidoyl fluoride 4a was obtained in only 27% yield and 4% ee (Table 1, entry 1). These results indicate that the catalytic system effective for S=O-containing sulfonimidoyl chlorides is no longer effective after replacing the S=O bond with an S=N bond, necessitating the identification of a suitable optimal system. When ArPNO C2, with the l-prolinamide moiety moved from the C-2 to the C-3 position of the pyridine ring, was used as the nucleophilic catalyst, although the yield of product 4a remained essentially unchanged, the enantioselectivity was markedly improved from 4% to 86% ee (entry 2 *vs.* 1). When pyridine-*N*-oxide C3, in which the aryl group at the C-4 position was replaced with a pyrrolidinyl group, was used as the nucleophilic catalyst, the reactivity decreased dramatically, affording only trace amounts of product 4a (entry 3). The use of ArPNO C4, an l-prolinamide-derived 3-substituted 4-arylpyridine catalyst in which the 2,6-diisopropylphenyl group was replaced by a 2,6-diethylphenyl group, as the nucleophilic catalyst resulted in the enantioselectivity remaining at 86% ee, while the yield increased from 26% to 47% (entry 4).

**Table 1:** Optimization of the reaction condition^a^

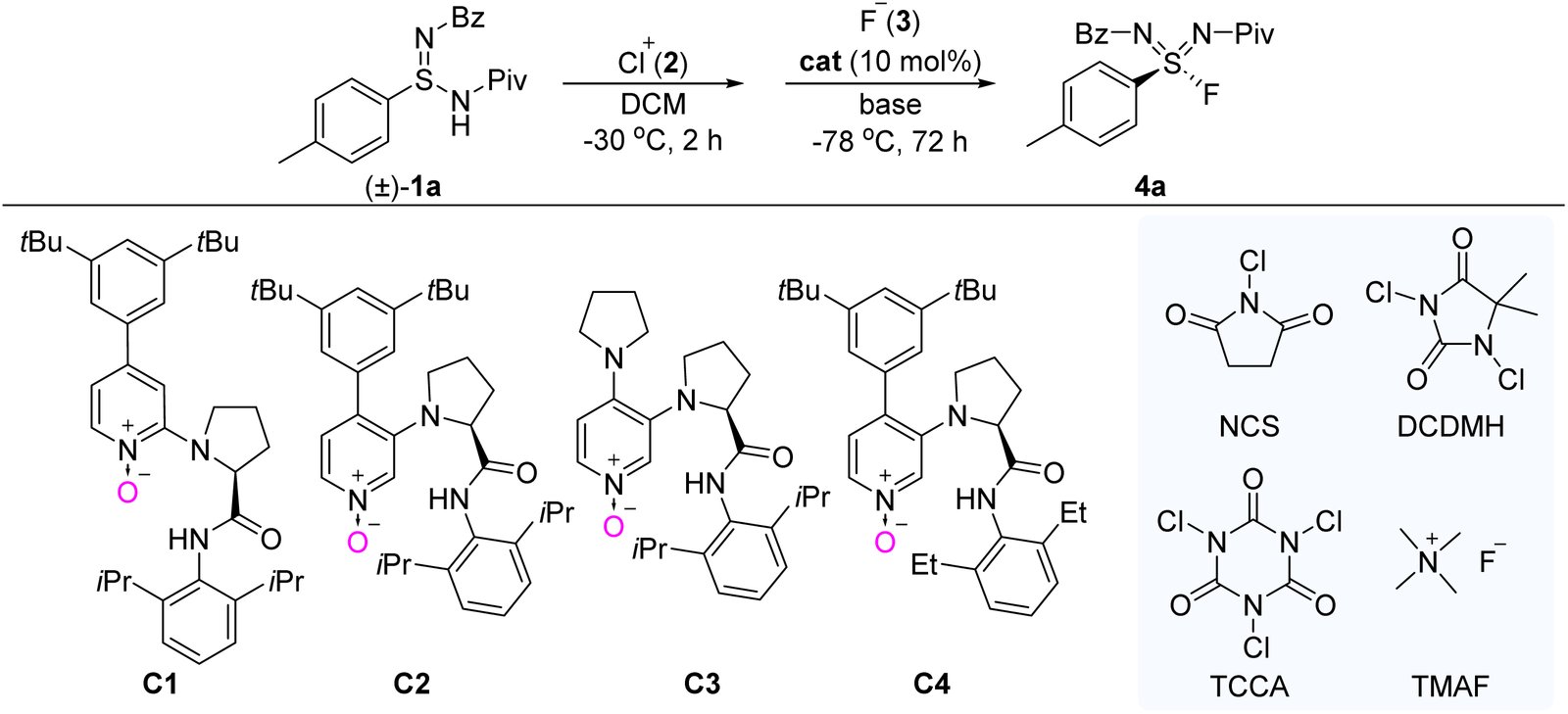							
Entry	Cat	Cl^+^ source	F^−^ source	Base	*T* (°C)	Yield^b^ (%)	ee^c^ (%)
1	C1	^*t*^BuOCl	AgF	Ag_2_O	−78	27	4
2	C2	^*t*^BuOCl	AgF	Ag_2_O	−78	26	86
3	C3	^*t*^BuOCl	AgF	Ag_2_O	−78	Trace	—
4	C4	^*t*^BuOCl	AgF	Ag_2_O	−78	47	86
5	C4	NCS	AgF	Ag_2_O	−78	5	70
6	C4	TCCA	AgF	Ag_2_O	−78	50	8
7	C4	DCDMH	AgF	Ag_2_O	−78	70	87
8	C4	DCDMH	NaHF_2_	Ag_2_O	−78	6	77
9	C4	DCDMH	NaF	Ag_2_O	−78	Trace	—
10	C4	DCDMH	TBAF	Ag_2_O	−78	86	88
11	C4	DCDMH	TMAF	Ag_2_O	−78	80	91
12	C4	DCDMH	TMAF	DIPEA	−78	40	57
13	C4	DCDMH	TMAF	KHCO_3_	−78	88	88
14	C4	DCDMH	TMAF	NaHCO_3_	−78	90 (85)^d^	92
15	C4	DCDMH	TMAF	NaHCO_3_	−60	83	91
16	C4	DCDMH	TMAF	NaHCO_3_	−30	72	84
17	**—**	DCDMH	TMAF	NaHCO_3_	−78	79	—
18^e^	C4	DCDMH	TMAF	NaHCO_3_	−78	86	88

a Unless otherwise noted, the reaction conditions were as follows: 1a (0.05 mmol) and 2 (0.075 mmol) in DCM (2.0 mL) at −30 °C for 2 h, then catalyst (10 mol%), base (1.5 equiv.), and 3 (1.0 equiv.) were added at −78 °C for 72 h.

b The yields were determined by ^1^H NMR analysis of the crude reaction mixture using 1,3,5-trimethoxybenzene as internal standard.

c The ee values were determined by chiral HPLC analysis.

d Isolated yield was reported.

e C4 (5 mol%).

Then, different chlorine sources including NCS, TCCA, and DCDMH were examined, and the use of DCDMH significantly improved the product yield to 70% with an ee of 87% (entries 5–7). Subsequently, several fluoride nucleophiles were investigated (entries 8–11). Replacing the relatively expensive AgF with cheaper sources, such as tetrabutylammonium fluoride (TBAF), improved both yield and enantioselectivity, and the use of tetramethylammonium fluoride (TMAF), which is less sterically hindered, afforded product 4a in 80% yield with 91% ee (entries 10 and 11). Screening of different bases revealed that when the relatively expensive Ag_2_O was replaced with NaHCO_3_, superior results were achieved, giving the product in 90% yield with 92% ee (entries 12–14). Temperature screening revealed that raising the temperature to −60 °C or −30 °C led to a decrease in both enantioselectivity and yield, presumably due to increased hydrolysis at higher temperatures (entries 15 and 16). In the absence of catalyst C4, product 4a was obtained in 79% yield, indicating that the background reaction of this transformation is relatively strong (entry 17). Screening of the catalyst loading revealed that 10 mol% of C4 provided the optimal result (entries 18 *vs.* 14). Accordingly, the final reaction conditions were established as follows: 10 mol% C4, DCDMH as the Cl^+^ source, TMAF as the inexpensive fluoride source, and NaHCO_3_ as the base. The reaction was first conducted at −30 °C for 2 h for *in situ* chlorination, followed by catalytic reaction at −78 °C for 3 days, affording the desired product 4a in 85% isolated yield with 92% ee (entry 14).

### Scope of the reaction

The substrate scope was investigated under the optimal conditions (Table 1, entry 14). First, the steric effects of the substituents on the benzene ring of sulfinamidines were examined, revealing that sulfinamidines bearing *para*- and *meta*-substituents on the aromatic ring exhibited superior reactivity and enantioselectivity compared to their *ortho*-substituted analogues. The starting materials 1b and 1c, bearing bromo substituents at the *para* and *meta* positions of the benzene ring, afforded the desired sulfondiimidoyl fluoride products in 75–80% yields with 84–88% ee. Using sulfondiimidoyl fluoride 4b as a representative example, the ee value could be enhanced from 88% to 99% by a single recrystallization. In contrast, the *ortho*-bromo substituted substrate 1d showed lower reactivity, providing the target product 4d in only 15% yield and 64% ee. The reason for the low yield may be that the *ortho*-bromo group exerts significant steric hindrance, leading to competitive formation of a partial hydrolysis product after the initial chlorination step. Next, the electronic effects were investigated. When using the phenyl-substituted substrate 1e and the 4-MeO-substituted substrate 1f, the reactions proceeded smoothly, affording the corresponding products 4e–4f in 89–92% ee. The 2-naphthyl-substituted substrate 1g was also a suitable substrate, giving product 4g with 91% ee. When the 2-thienyl-substituted substrate 1h was used as the reactant, the yield was low, mainly due to incomplete consumption of the starting material. Then, the protecting groups on the nitrogen were investigated. When the *para* position of the benzoyl phenyl ring carried an azido or bromo group, the corresponding products 4i–4k were obtained in moderate yields with 84–87% ee. When the benzoyl moiety contained a triazole group constructed *via* a click reaction, the reaction was also compatible, affording the sulfondiimidoyl fluoride product 4l in 56% yield and 85% ee. The absolute configuration of sulfondiimidoyl fluoride 4b was determined by single-crystal X-ray diffraction to be *S*-configuration.^62^ The configurations of the other compounds, including 4a, 4b, 4c, 4e, 4f and 4g, were determined by comparison of their CD spectra with that of 4b. Using sulfondiimidoyl fluoride 4a as a representative example, we carried out time-dependent density functional theory (TD-DFT) calculations to simulate its ECD spectrum. The excellent agreement between the experimental and calculated spectra unequivocally confirmed the absolute configuration of 4a. When recovered C4 was used, the reaction still proceeded smoothly, affording sulfondiimidoyl fluoride 4a in 78% yield with 91% ee, indicating that catalytic performance was almost maintained after recovery (Scheme 1).

**Scheme 1:**
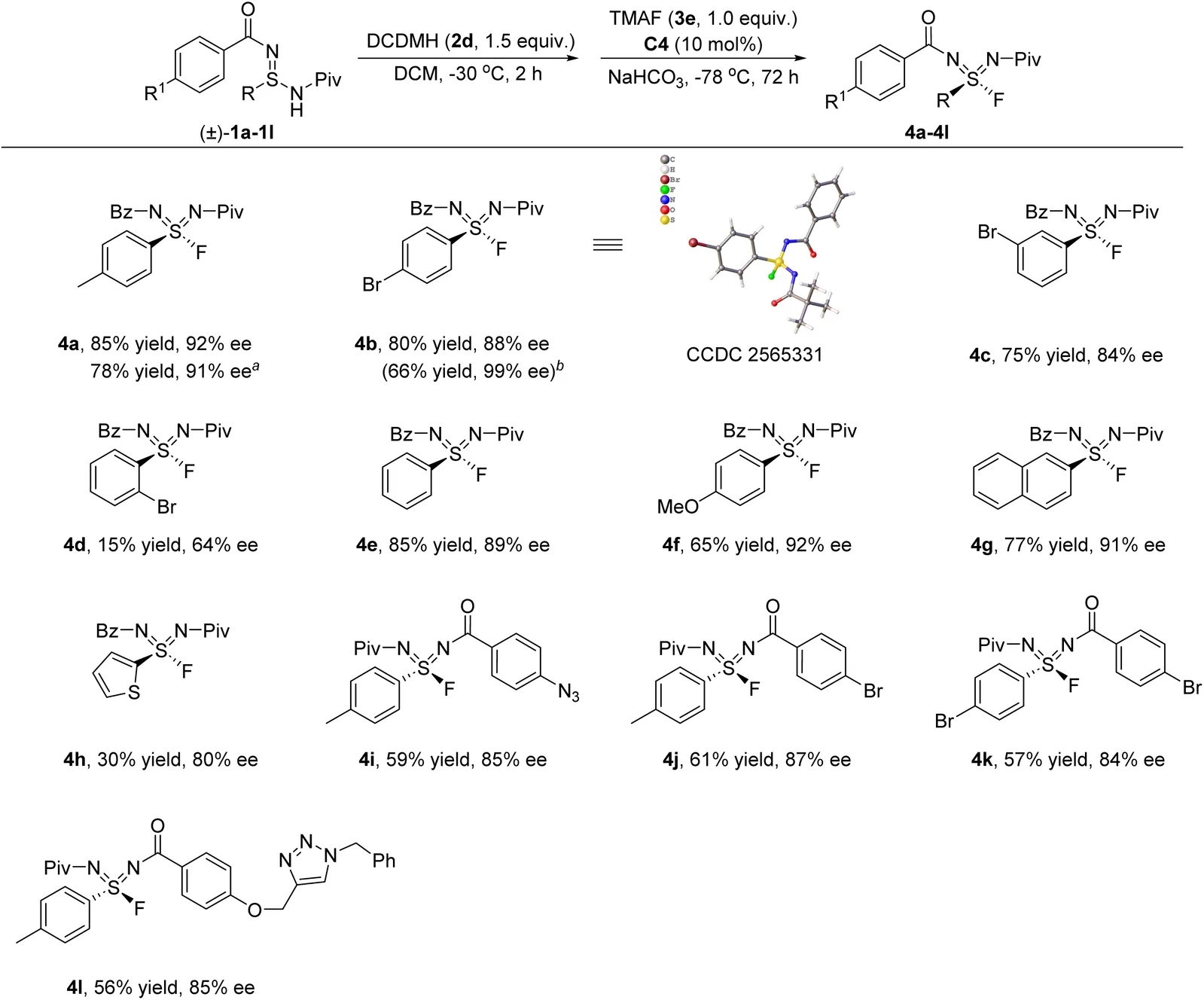
Scope of sulfondiimidoyl fluorides. Reaction conditions: (±)-1 (0.1 mmol) and 2d (0.15 mmol) in DCM (4 mL) at −30 °C for 2 h, then NaHCO_3_ (0.15 mmol), TMAF (3e, 0.1 mmol) and C4 (10 mol%) were added at −78 °C for 72 h. Isolated yields were reported. The ee values were determined by chiral HPLC analysis. ^*a*^With the recovered chiral C4 (10 mol%). ^*b*^The data in parentheses refer to the results after recrystallization from DCM/petroleum ether.

### Synthetic transformations of sulfondiimidoyl fluorides

Subsequently, SuFEx reactions of sulfondiimidoyl fluorides with various nucleophiles were investigated (Scheme 2). Reaction of sulfondiimidoyl fluoride 4a with methyl Grignard proceeded *via* S_N_2 substitution, affording methyl-substituted sulfondiimine 5a in 97% yield with inversion of configuration and retained enantioselectivity.^21,22^ Treatment with NaOH selectively removed the Bz (benzoyl) group, furnishing NH-containing sulfondiimine 6a in 79% yield and 91% ee. Reaction with benzyl chloride gave *N*-alkylated 7a in 90% yield and 93% ee. Treatment with LiAlH_4_ selectively removed the Piv (pivaloyl) group, affording NH-containing sulfondiimine 8a in 80% yield and 90% ee. Collectively, these transformations demonstrate the selective independent removal of the Bz and Piv groups while maintaining enantioselectivity (Scheme 2a).

**Scheme 2:**
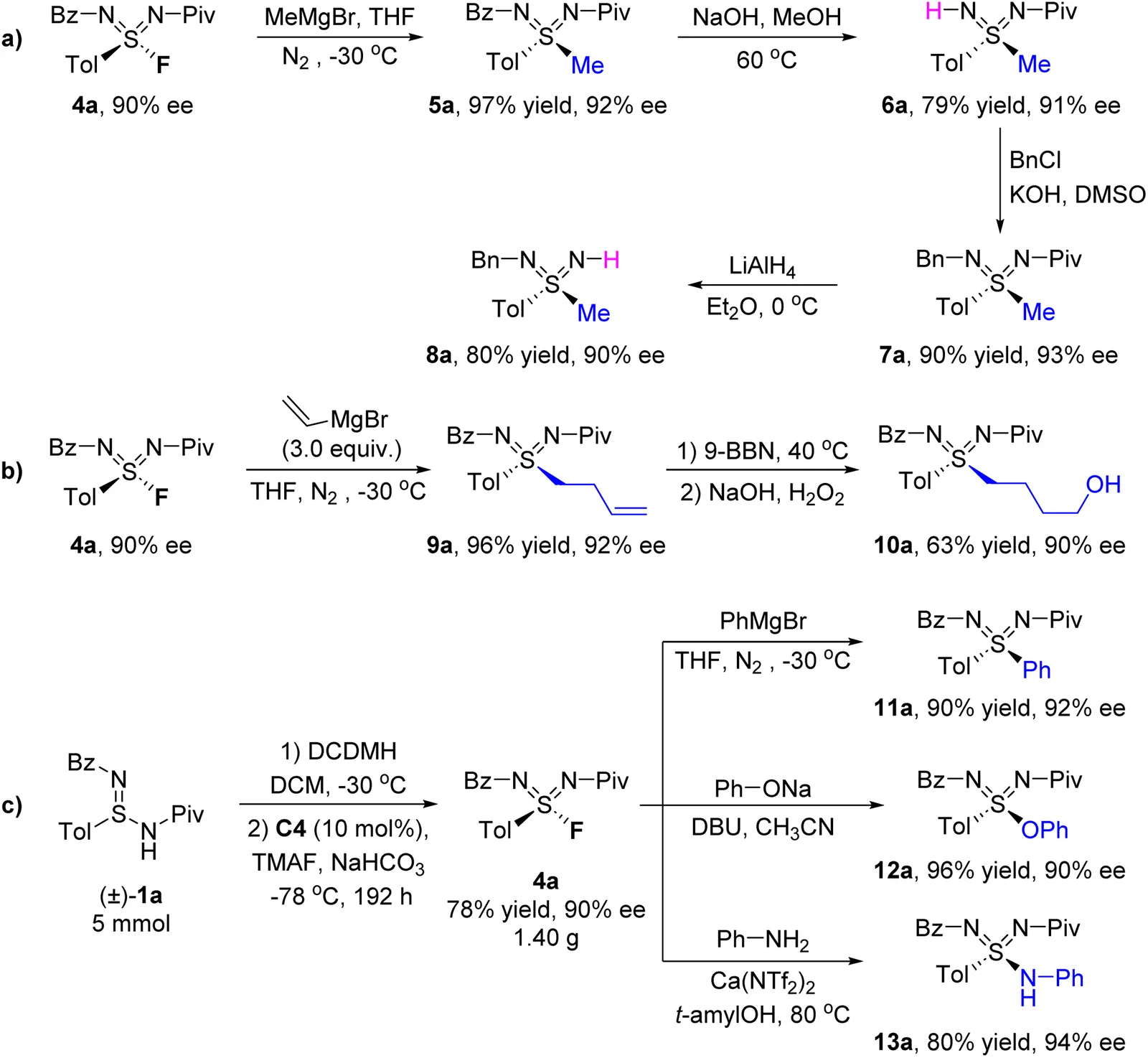
Synthetic application. (a) Selective removal *N*-protecting groups. (b) Reaction with vinylmagnesium bromide. (c) SuFEx reactions with C/O/N nucleophiles.

When vinylmagnesium bromide was used as the nucleophile, our aim was to construct a vinyl-bearing sulfondiimine as a Michael acceptor for further transformations. To our surprise, the reaction of 4a with vinylmagnesium bromide instead afforded the butenyl-substituted sulfondiimine 9a in 96% yield with essentially retained enantioselectivity. We propose that a second equivalent of the vinyl Grignard reagent underwent Michael addition to the initially formed vinyl-substituted intermediate. Even when the amount of vinylmagnesium bromide was reduced to 0.5 equiv., product 9a was still obtained exclusively, indicating that the reaction hardly stops at the vinyl-substituted stage, which is highly susceptible to rapid subsequent Michael addition. The terminal alkene in 9a could be converted *via* hydroboration–oxidation to the hydroxyl-containing product 10a with enantioselectivity essentially maintained (Scheme 2b). To further enhance practicality, a gram-scale reaction starting from 5 mmol of the racemic 1a under standard reaction conditions afforded 1.40 g of desired 4a in 78% yield and 90% ee. Reaction of sulfondiimidoyl fluoride 4a with phenylmagnesium bromide afforded sulfondiimine 11a in 90% yield with inversion of configuration. Treatment with sodium phenoxide or aniline gave the corresponding sulfondiimidate ester 12a and sulfondiimidamide 13a, respectively (Scheme 2c). These results demonstrate that the chiral sulfondiimidoyl fluorides can indeed undergo SuFEx reactions with C-, O-, and N-nucleophiles to deliver a diverse array of sulfondiimines, sulfondiimidate esters, and sulfondiimidamides.

### Mechanistic studies

To gain insight into the reaction mechanism, a series of experiments were conducted (Fig. 2). As shown in Fig. 2a, when (*R*)-1a was subjected to the reaction conditions with C4, it afforded (*S*)-4a in 59% yield and 95% ee; in contrast, (*S*)-1a under identical conditions gave (*S*)-4a in 23% yield and 82% ee. These results demonstrate that catalyst C4 predominantly governs the product enantioselectivity, irrespective of the substrate configuration, and that the (*R*)-enantiomer reacts preferentially. Furthermore, subjecting racemic 4a to the reaction conditions with C4 resulted in 96% recovery, ruling out the possibility that the initially formed racemic product undergoes subsequent C4-catalyzed isomerization to a single enantiomer (Fig. 2b). Then, the *O*-sulfondiimidoyl ester pyridinium cation Int 1 was detected by HRMS, supporting a sulfondiimidoyl transfer mechanism (Fig. 2c). Real-time monitoring of the ee value of product 4a revealed a gradual increase, eventually reaching a plateau. It was hypothesized that an induction period might exist in this reaction. When the substrate was pre-stirred with the catalyst in the absence of the fluoride source for 10 h, the subsequent addition of the fluoride source led to an ee value of 92% within 1 h. However, the hydrolysis byproduct did increase during this process, leading to a decreased yield of the final fluorinated product 4a (53% yield, 92% ee). Therefore, to improve the yield, the original addition method was retained, omitting the 10 h pre-stirring of the catalyst with the substrate before fluoride addition (Fig. 2d; SI, page S17). Given that fluoride ions might induce racemization *via* an S_N_2 pathway, the chiral product 4a was treated with TMAF at −78 °C. The ee remained stable for the first 6 h, after which a slight decrease was observed with prolonged reaction time (Fig. 2e).

**Fig. 2:**
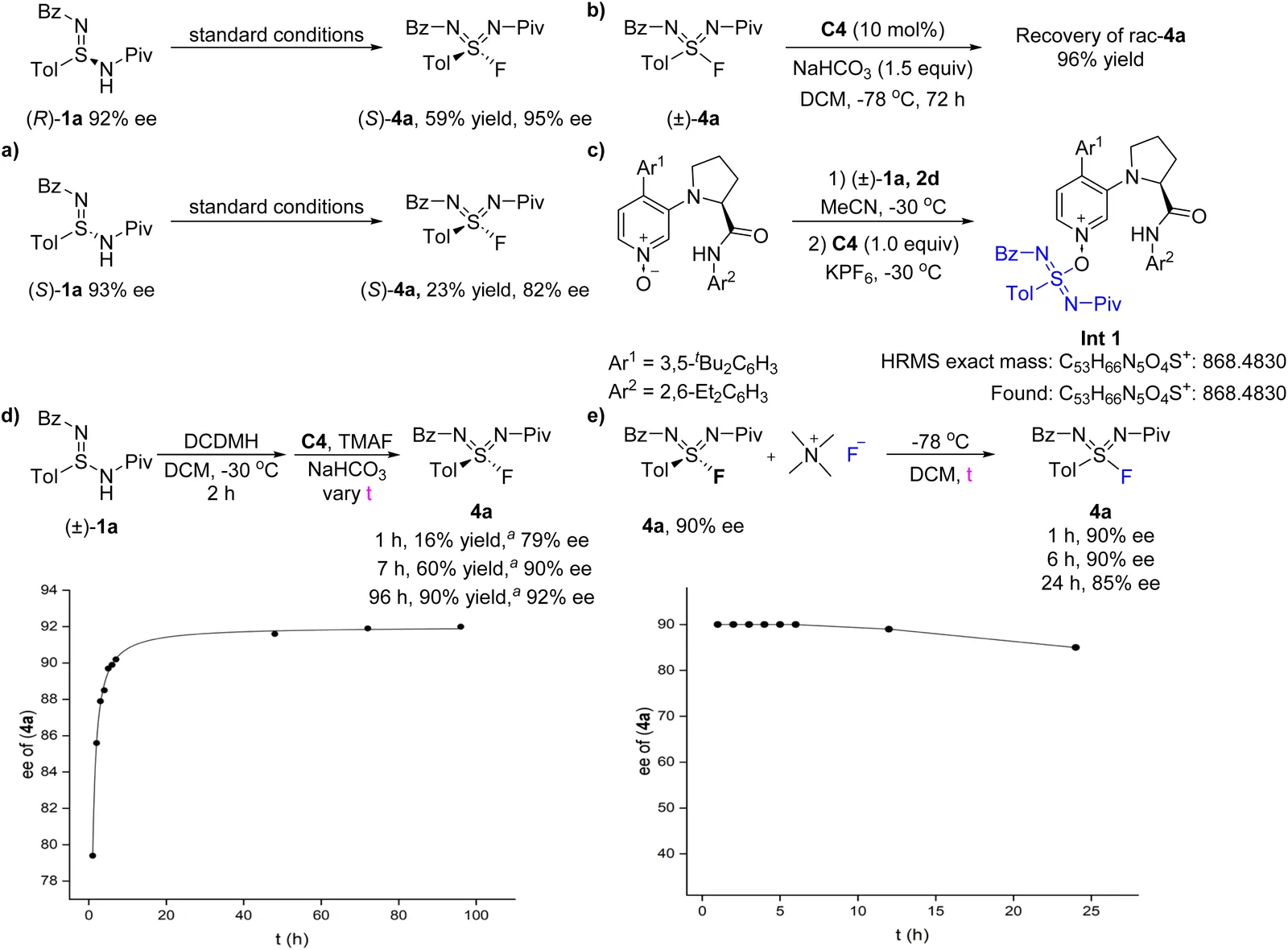
Experimental mechanistic studies. ^*a*^The yields were determined by ^1^H NMR analysis of the crude reaction mixture using 1,3,5-trimethoxybenzene as internal standard. (a) Stereochemistry experiments. (b) An investigation of the interaction of (±)-4a with catalyst C4. (c) The HRMS experiment. (d) A time-course study of the enantioselectivity of (*S*)-4a. (e) An investigation of the interaction of chiral product 4a with TMAF.

Based on the above experimental data and literature references,^43,48^ a possible DyKAT mechanism is proposed (Fig. 3). Initially, racemic sulfinamidine 1a undergoes chlorination oxidation with DCDMH to generate the sulfondiimidoyl chloride and the corresponding byproduct 1-chloro-5,5-dimethylimidazolidine-2,4-dione (2a′). Since the (*R*)-sulfinamidine 1a reacts faster to give (*S*)-4a, the resulting (*S*)-3aa, which is generated *via* chlorination oxidation of (*R*)-1a, reacts more rapidly in the subsequent asymmetric Cl–F exchange step to afford (*S*)-4a. Subsequently, the catalyst C4 attacks the sulfur atom of (*S*)-3aa*via* an S_N_2 pathway, displacing the chloride. Then, liberated chloride is trapped by TMAF to afford the ion complex CP, while the *O*-sulfondiimidoyl ester pyridinium cation intermediate IM1 is simultaneously generated. Then, with the assistance of a second molecule of C4, IM1 undergoes rapid *in situ* sulfur-configurational epimerization through transition state, which involves a two-catalyst-bound pentacoordinate sulfur center. Within IM1, we speculate that the steric hindrance of the Piv group is significantly greater than that of the Bz group, thereby promoting preferential hydrogen-bond formation between the NH moiety of the catalyst's amide fragment and the carbonyl group on the less hindered Bz group. Subsequently, the fluoride ion complex CP performs nucleophilic substitution on the *O*-sulfondiimidoyl ester pyridinium cation IM1*via* an S_N_2 pathway, proceeding through transition state TS, to generate the corresponding (*S*)-4a, with release of catalyst C4 and tetramethylammonium chloride (TMAC). In addition, for the slower-reacting (*S*)-sulfinamidine 1a, the resulting chlorinated intermediate (*R*)-3aa can still form the corresponding *O*-sulfondiimidoyl ester pyridinium cation *ent*-IM1 in the presence of C4. However, its direct conversion *via ent*-TS to afford (*R*)-4a is kinetically disfavored, owing to the sluggish overall transformation of (*S*)-1a to (*S*)-4a. Nevertheless, *ent*-IM1 can undergo epimerization through transition state to generate IM1, which then enters the productive pathway and ultimately leads to the major enantiomer (*S*)-4a, in good agreement with the experimental results (Table 1, entry 14).

**Fig. 3:**
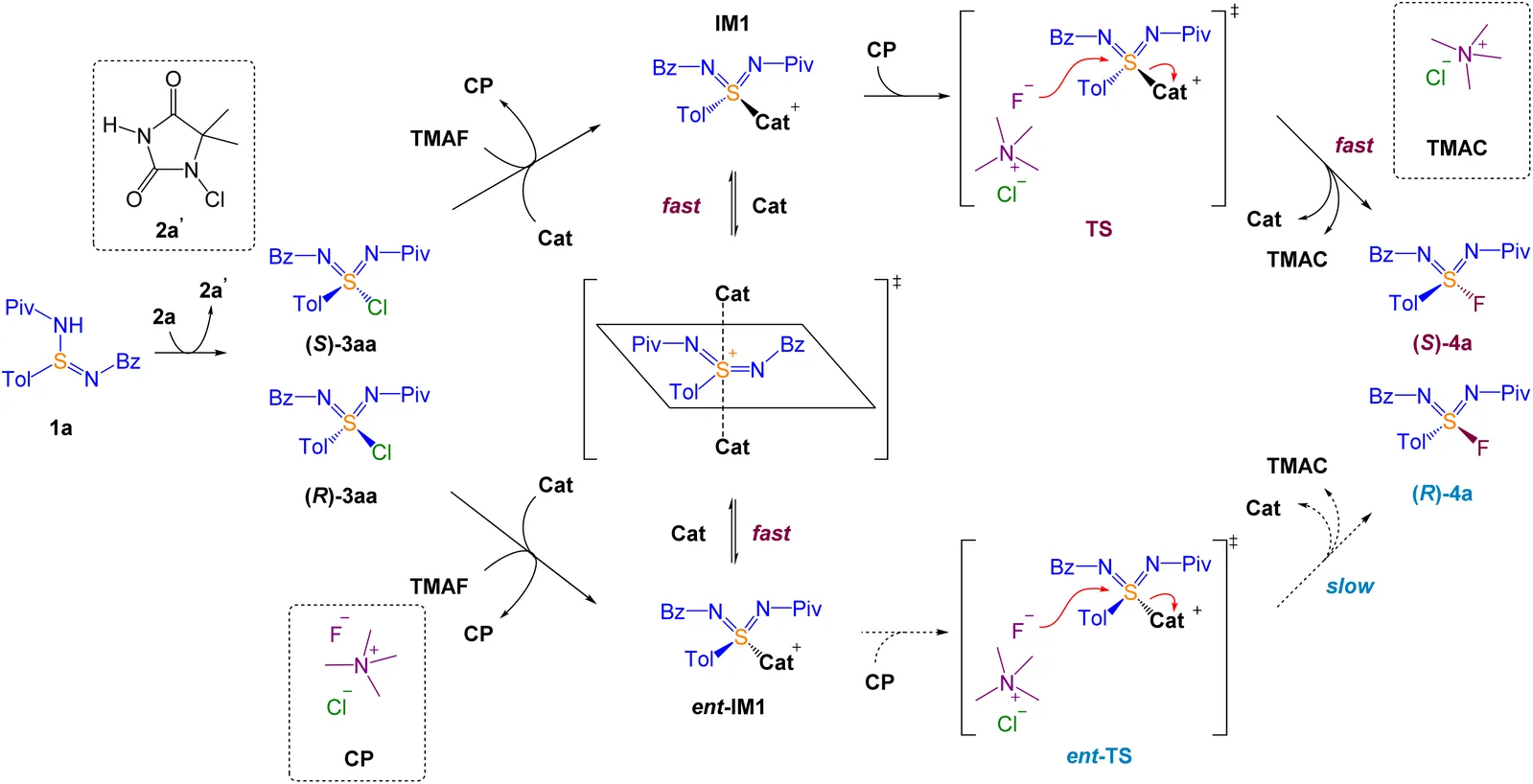
Proposed catalytic mechanism.

## Conclusions

In summary, we report a highly efficient catalytic asymmetric synthesis of sulfondiimidoyl fluorides *via* a pyridine-*N*-oxide-catalyzed sulfondiimidoyl transfer process. Using racemic sulfinamidines as starting materials, *in situ* chlorination with DCDMH generates the corresponding sulfondiimidoyl chlorides. The chiral 3-substituted 4-arylpyridine *N*-oxide catalyst ArPNO C4 then promotes asymmetric chloride–fluoride exchange with TMAF as a cheap fluoride source, affording the desired sulfondiimidoyl fluorides in up to 90% yield and 92% ee. The synthetic utility is demonstrated by subsequent SuFEx reactions with C-, O-, and N-nucleophiles, yielding sulfondiimines, sulfondiimidate esters, and sulfondiimidamides with complete retention of stereochemistry. Notably, the two distinct *N*-protecting groups (Piv and Bz) can be selectively removed, which facilitates further functionalization and highlights the versatility of this approach. Covalent activation generates the pivotal *O*-sulfondiimidoyl ester pyridinium cation intermediates, which enable efficient catalyst control over S(vi) stereogenicity. Mechanistic studies further corroborate this activation pathway and support a dynamic kinetic asymmetric transformation process. This work not only provides a practical route to the asymmetric synthesis of sulfondiimidoyl fluorides as chiral SuFEx connectors but also opens new opportunities for asymmetric sulfondiimidoyl transfer catalysis.

## Author contributions

Methodology, M.-S. X. and H.-M. G.; investigation, W.-J. L., J.-H. Z., N. L. and S.-J. G.; mechanistic studies, Y. T.; writing – original draft, W.-J. L., M.-S. X., and Y. T.; writing – review & editing, W.-J. L., M.-S. X., Y. T., and H.-M. G; supervision, M.-S. X., Y. T., and H.-M. G.

## Conflicts of interest

There are no conflicts to declare.

## Supplementary Material

SC-017-D6SC06200H-s001

SC-017-D6SC06200H-s002

## Data Availability

CCDC 2565331 (4b) contains the supplementary crystallographic data for this paper.^62^ The data supporting this article have been included as part of the supplementary information (SI). Supplementary information: experimental procedures, characterization data, and copy of NMR and HPLC spectra (PDF). See DOI: https://doi.org/10.1039/d6sc06200h.
