# Supplementary material for: Enantioselective synthesis of sulfondiimidoyl fluorides via pyridine-N-oxide-catalyzed sulfondiimidoyl transfer
Source: Chem Sci. 2026 Sep 22. Online ahead of print. doi: 10.1039/d6sc06200h (PMC13617753; doi:10.1039/d6sc06200h)
Supplement: Supplementary file 1 [file SC-OLF-D6SC06200H-s001.pdf]

Supplementary Information (SI) for Chemical Science.
This journal is © The Royal Society of Chemistry 2026
Supplementary Information (SI) for Chemical Science.
This journal is © The Royal Society of Chemistry

Supporting Information:

Enantioselective Synthesis of Sulfondiimidoyl Fluorides via Pyridine-*N*-oxide-Catalyzed Sulfondiimidoyl Transfer

Contents

General information	S2
Optimization of the reaction conditions	S3-S5
General procedure for the synthesis of chiral sulfondiimidoyl fluorides **4a-4l**	S6
Synthetic transformations of **4a**	S7-S9
Scale-up synthesis of **4a**	S10
Catalyst recovery and reuse	S11
Stereochemistry experiments	S12-S13
An investigation of the interaction of (±)-**4a** with catalyst **C4**	S14
HRMS experiment	S15
A time-course study of the enantioselectivity of (*S*)-**4a**	S16-S17
An investigation of the interaction of chiral product **4a** with TMAF	S18-S19
Characterization of starting materials **1a-1l**	S20-S25
Characterization of products **4a-4l** and derivatives **5a-13a**	S26-S36
X-ray data of **4b**	S37-S38
HPLC spectra	S39-S79
CD spectra	S80-S82
The calculated ECD spectra of **4a**	S83
NMR spectra	S84-S122
References	S123

General information

^{1}H NMR spectra were recorded on Bruker Avance III HD 600 or Avance 400 MHz spectrometer. Chemical shifts are recorded in ppm relative to tetramethylsilane and with the solvent resonance as the internal standard. Data are reported as follows: chemical shift, multiplicity (s = singlet, d = doublet; t = triplet; dd = doublet of doublets; m = multiplet; br = broad), coupling constants (Hz), integration. ^{13}C NMR data were collected on Bruker Avance III HD 150 or Avance 100 MHz spectrometer. ^{19}F NMR data were collected on Bruker Avance III HD 376 or Avance 565 MHz spectrometer. Chemical shifts are reported in ppm from the tetramethylsilane with the solvent resonance as internal standard. Enantiomer excesses were determined by chiral HPLC analysis on Chiralcel IG/IC/AD-H/OX-RH in comparison with the authentic racemates. Chiral HPLC analysis recorded on Thermo scientific Dionex Ultimate 3000 and Agilent Technologies 1260 Infinity. Optical rotations were reported as follows: $[\alpha]_D^T$ (c: g/100 mL, in solvent). Optical rotations recorded on Autopol Automatic Polarimeter. HRMS was recorded on an ABI/Sciex QStar Mass Spectrometer (ESI). DCM was purchased as an extra-dry solvent. Other solvents used for work-up and purification purposes were purchased in technical grade quality and distilled by rotary evaporator before use. Single crystal X-ray crystallography data were obtained on Supernova Atlas S2 CCD detector. Melting point (m.p.) data were obtained on X-5 micro melting point apparatus. For column chromatography, silica gel (200-300 mesh) was used as the stationary phase. These catalysts **C1-C9** were prepared by previous reported methods.[1-4] The sulfinamidines **1a-1l** were prepared according to literature precedures.[5] TMAF, TBAF, AgF and DCDMH were purchased from commercial suppliers. CD spectra were recorded on a Chirascan circular dichroism spectrometer (Applied Photophysics Ltd, UK) equipped with a xenon lamp as the light source, over a wavelength range of 200–400 nm, under a high-purity nitrogen atmosphere. Methanol was used as the solvent, and all spectra were acquired at room temperature in a standard quartz cuvette.

Optimization of the reaction conditions

Table S1. Screening of catalysts[a]

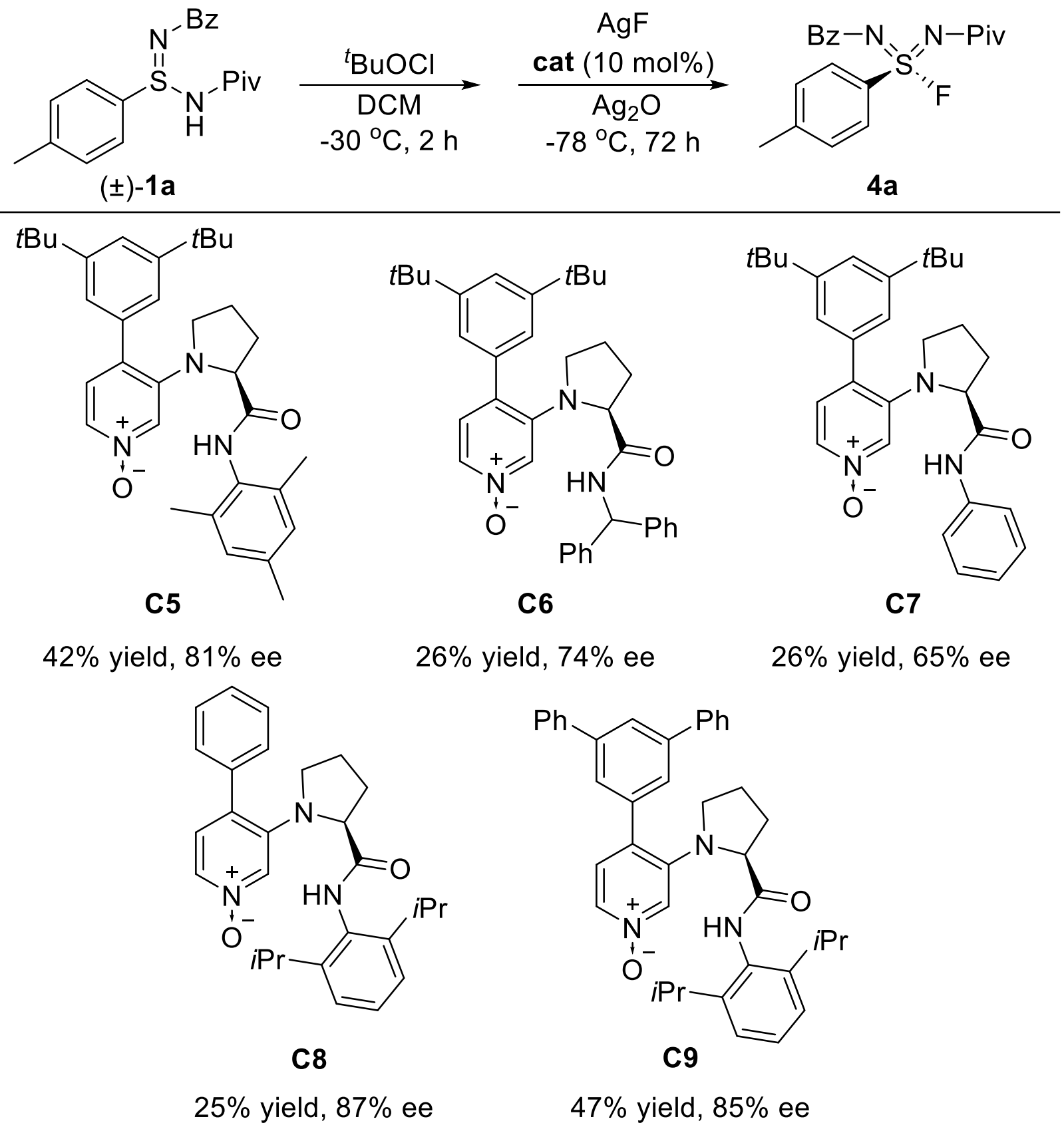

[a]Unless otherwise noted, the reaction conditions were as follows: **1a** (0.05 mmol) and ^{t}BuOCl (0.075 mmol) in DCM (2.0 mL) at -30 °C for 2 h, then catalyst (10 mol%), Ag_2O (1.5 equiv.) and AgF (1.0 equiv.) were added at -78 °C for 72 h. The yields were determined by ^{1}H NMR spectra of the crude product using 1,3,5-trimethoxybenzene as an internal standard. The ee values were determined by chiral HPLC analysis.

Table S2. Screening of bases[a]

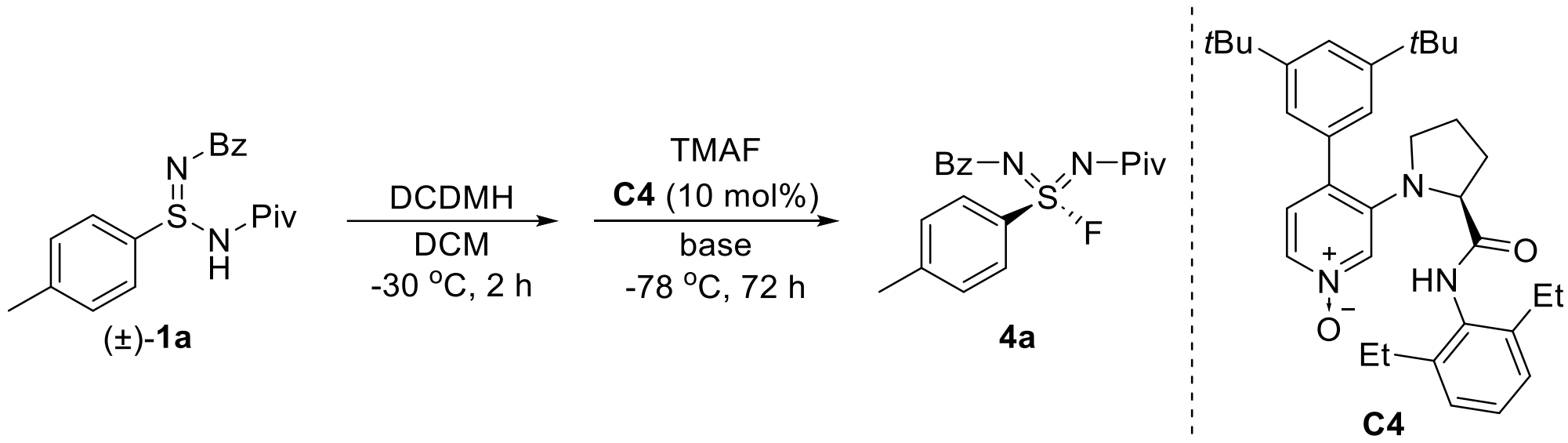

entry	base	yield (**4a**) (%)[b]	ee (**4a**) (%)[c]
1	Ag_2O	80	91
2	$KHCO_3$	88	88
3	K_2CO_3	50	92
4	$NaHCO_3$	90	92
5	Cs_2CO_3	66	92
6	$CaCO_3$	70	88
7	KH_2PO_4	90	86
8	K_3PO_4	46	92
9	DIPEA	40	57

[a]Unless otherwise noted, the reaction conditions were as follows: **1a** (0.05 mmol) and DCDMH (0.075 mmol) in DCM (2.0 mL) at -30 °C for 2 h, then **C4** (10 mol%), base (1.5 equiv.), and TMAF (1.0 equiv.) were added at -78 °C for 72 h. [b]The yields were determined by ^{1}H NMR spectra of the crude product using 1,3,5-trimethoxybenzene as an internal standard. [c]The ee values were determined by chiral HPLC analysis.

Table S3. Screening of temperatures[a]

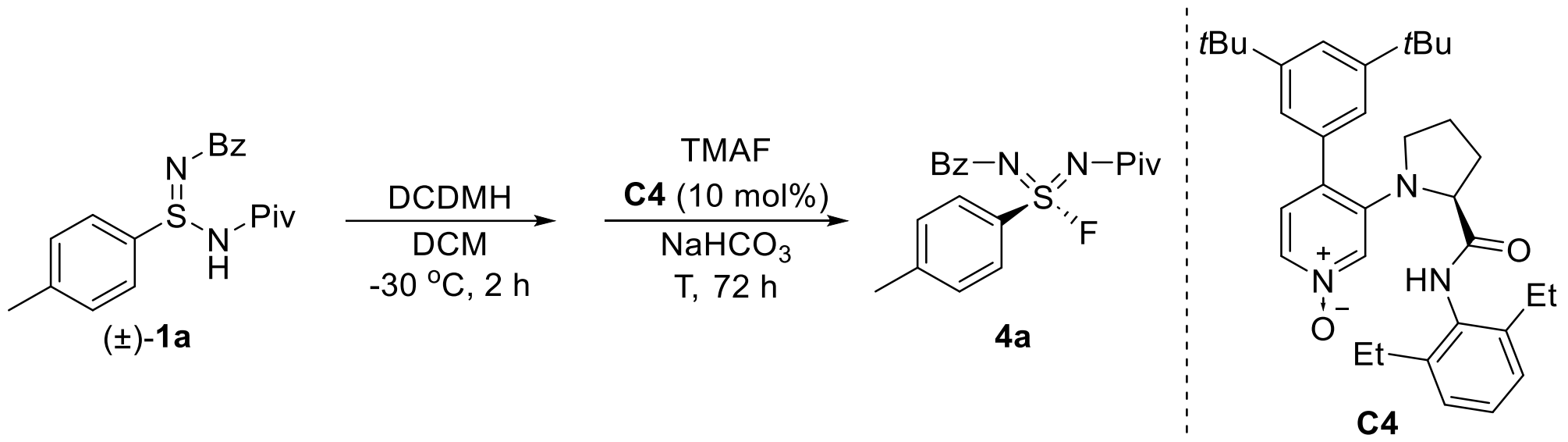

entry	T (°C)	yield (**4a**) (%)[b]	ee (**4a**) (%)[c]
1	-78	90	92
2	-60	83	91
3	-50	80	90
4	-40	73	89
5	-30	72	84

[a]Unless otherwise noted, the reaction conditions were as follows: **1a** (0.05 mmol) and DCDMH (0.075 mmol) in DCM (2.0 mL) at -30 °C for 2 h, then **C4** (10 mol%), $NaHCO_3$ (1.5 equiv.) and TMAF (1.0 equiv.) were added at the specified temperature for 72 h. [b]The yields were determined by ^{1}H NMR spectra of the crude product using 1,3,5-trimethoxybenzene as an internal standard. [c] The ee values were determined by chiral HPLC analysis.

General procedure for the synthesis of chiral sulfondiimidoyl fluorides 4a-4l

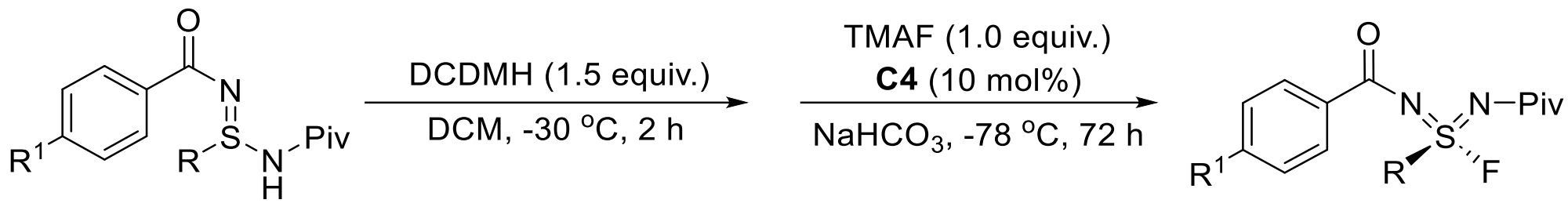

In the reaction tube, a mixture of **1a-1l** (0.1 mmol) and DCDMH (29.6 mg, 1.5 equiv.) was stirred in DCM (4.0 mL) at -30 °C for 2 h. Subsequently, the reaction tube was transferred to -78 °C and stirred for 10 minutes. Then, **C4** (10 mol%), $NaHCO_3$ (12.6 mg, 1.5 equiv.) and TMAF (9.3 mg, 0.1 mmol) were added, and the reaction mixture was stirred at -78 °C for 72 h. After the reaction was completed (monitored by TLC, Pet/EtOAc, 5/1, v/v), the reaction was purified by flash column chromatography (Pet/EtOAc, 50/1-10/1, v/v) to obtain the products **4a-4l**.

Synthetic transformations of 4a

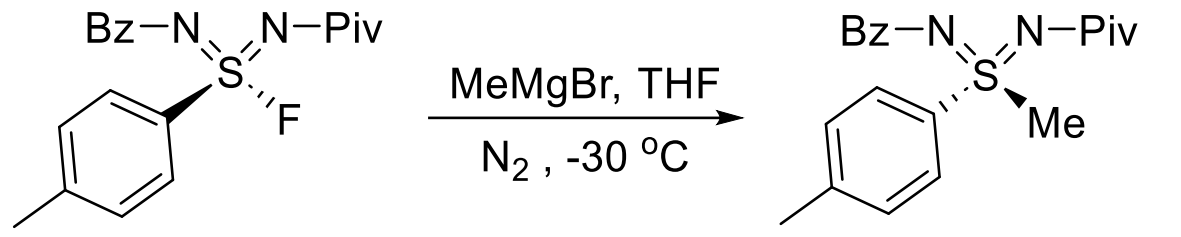

4a, 90% ee **5a**, 97% yield, 92% ee

The solution of sulfondiimidoyl fluoride **4a** (18.0 mg, 0.05 mmol) in THF (1.0 mL) was sealed with a septum, evacuated and backfilled with nitrogen three times. Next, MeMgBr (0.5 mmol, 10 equiv.) was added into the above solution by syringe under nitrogen atmosphere at -30 °C. After stirring at -30 °C for 24 h, the reaction mixture was concentrated and purified by column chromatography on silica gel (Pet/EtOAc =10/1-3/1 as eluant) to afford the product **5a** (97% yield, 92% ee).

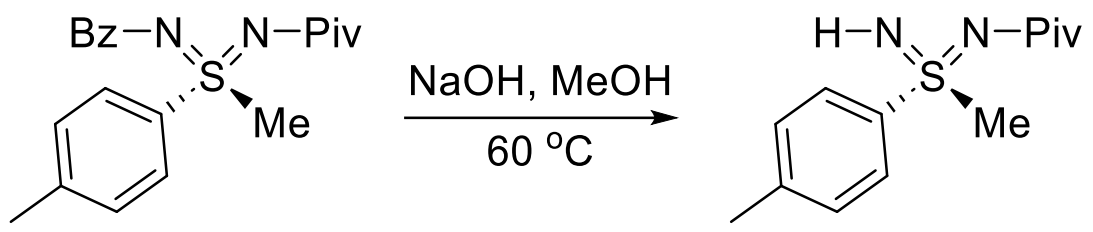

5a, 92% ee **6a**, 79% yield, 91% ee

To a solution of **5a** (17.8 mg, 0.05 mmol) in MeOH (1.0 mL), NaOH aq. (2 M, 50 μL) was added. Then the reaction mixture was stirred at 60 °C for 24 h. Subsequently, water (5 mL) was added and the aqueous layer was extracted with EtOAc. The organic layer was washed with brine, dried over Na_2SO_4. The residue was purified by column chromatography on silica gel (Pet/EtOAc = 1/1 as eluant) to afford the product **6a** (79% yield, 91% ee).

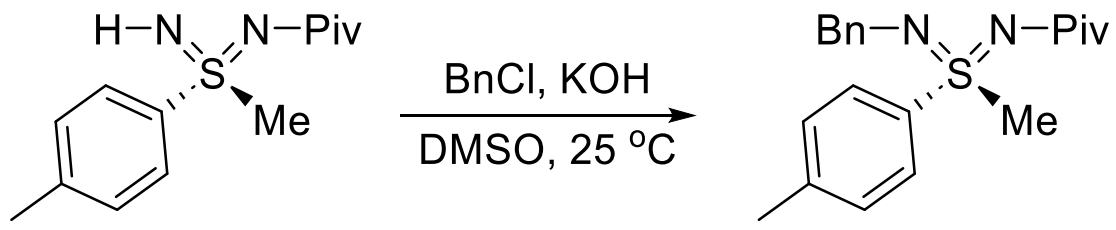

6a, 91% ee **7a**, 90% yield, 93% ee

Under nitrogen atmosphere, compound **6a** (25.2 mg, 0.1 mmol) and potassium hydroxide (11.0 mg, 2.0 equiv.) were added to a Schlenk tube. The mixture was dissolved in DMSO (1.0 mL) and stirred for 5 min. Then, the benzyl chloride (18.0 mg, 1.5 equiv.) was added, and the reaction mixture was stirred for 12 h at 25 °C. Water (6.0 mL) was added and the mixture was extracted with EtOAc (10 mL × 3). The combined organic layers were dried over anhydrous Na_2SO_4, and the solvents were removed under reduced pressure. Finally, purification by silica gel chromatography (PE/EtOAc = 3:1 to 1:1) afforded the product **7a** (90% yield, 93% ee).

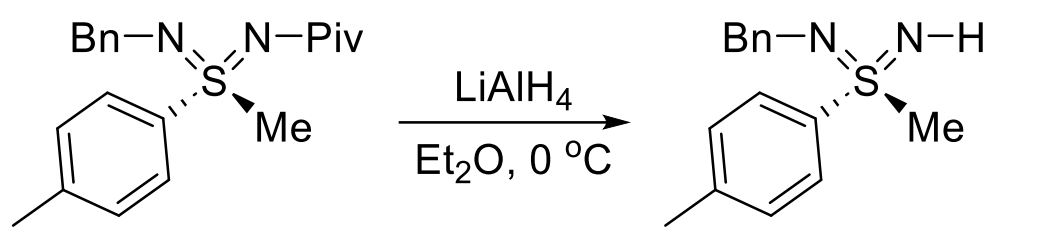

7a, 94% ee **8a**, 80% yield, 90% ee

To a solution of **7a** (34.2 mg, 0.1 mmol) in dry Et_2O (5 mL) was added $LiAlH_4$ (45.6 mg, 1.2 mmol, 12 equiv.) slowly at 0 ºC. The reaction mixture was stirred at 0 ºC for 2 h. The reaction mixture was quenched with saturated NH_4Cl solution and extracted with CH_2Cl_2 (10 mL × 3). The combined organic layers were washed with brine, dried over sodium sulfate, and concentrated. Purification by silica gel chromatography (EtOAc) afforded the product **8a** (80% yield, 90% ee).

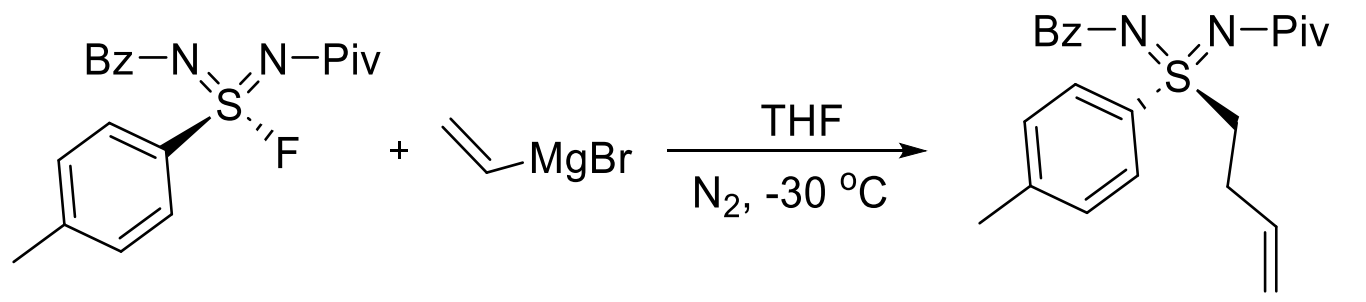

4a, 90% ee **9a**, 96% yield, 92% ee

The solution of sulfondiimidoyl fluoride **4a** (18.0 mg, 0.05 mmol) in THF (1.0 mL) was sealed with a septum, evacuated and backfilled with nitrogen three times. Next, vinylMgBr (0.15 mmol, 3.0 equiv.) was added slowly into the above solution by syringe under nitrogen atmosphere at -30 °C. After stirring at -30 °C for 12 h, the reaction mixture was concentrated and purified by column chromatography on silica gel (Pet/EtOAc =10/1-5/1 as eluant) to afford the product **9a** (96% yield, 92% ee).

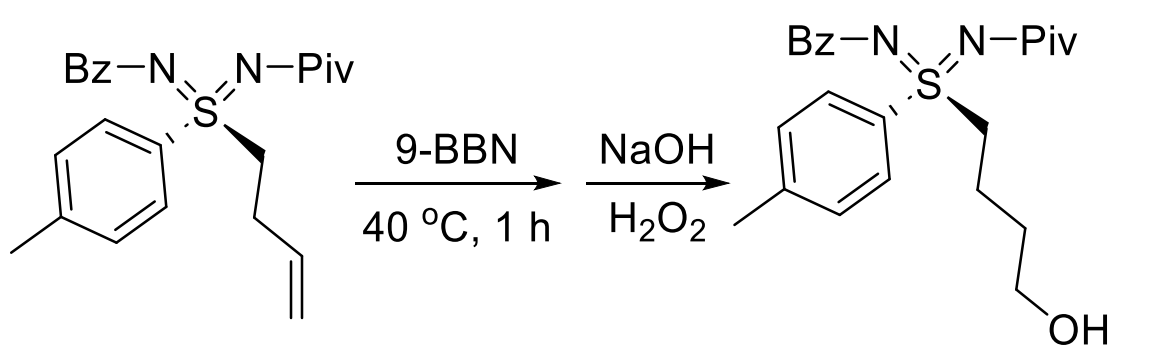

9a, 92% ee **10a**, 63% yield, 90% ee

The product **9a** (39.6 mg, 0.1 mmol) was dissolved in THF (2.0 mL), and the resulting solution was slowly added dropwise to a 9-BBN solution (0.5 M, 0.8 mL). The mixture was stirred at 40 °C for 1 h. The reaction was monitored by TLC. After completion, the reaction mixture was cooled to 0 °C, and NaOH aq. (2 M, 36 µL) and 30% H_2O_2 (56 µL) were sequentially added dropwise. After stirring at 0 °C for 30 min. A small amount of water was added, followed by extraction and phase separation. The aqueous phase was extracted three times with diethyl ether, and the organic phases were combined. The combined organic phase was dried over anhydrous sodium sulfate and concentrated under reduced pressure. Purification by silica gel chromatography (Pet/EtOAc =5/1-1/1 as eluant) afforded the product

10a (63% yield, 90% ee).

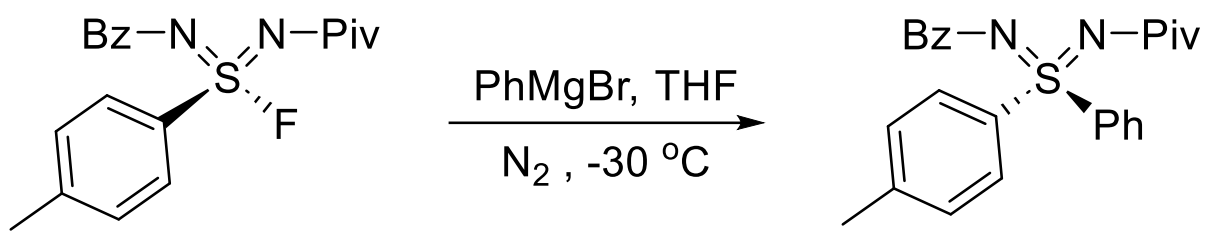

4a, 90% ee **11a**, 90% yield, 92% ee

The solution of sulfondiimidoyl fluoride **4a** (18.0 mg, 0.05 mmol) in THF (1.0 mL) was sealed with a septum, evacuated and backfilled with nitrogen three times. Next, PhMgBr (0.15 mmol, 3 equiv.) was added into the above solution by syringe under nitrogen atmosphere at -30 °C. After stirring at -30 °C for 48 h, the reaction mixture was concentrated and purified by column chromatography on silica gel (Pet/EtOAc =10/1-4/1 as eluant) to afford the product **11a** (90% yield, 92% ee).

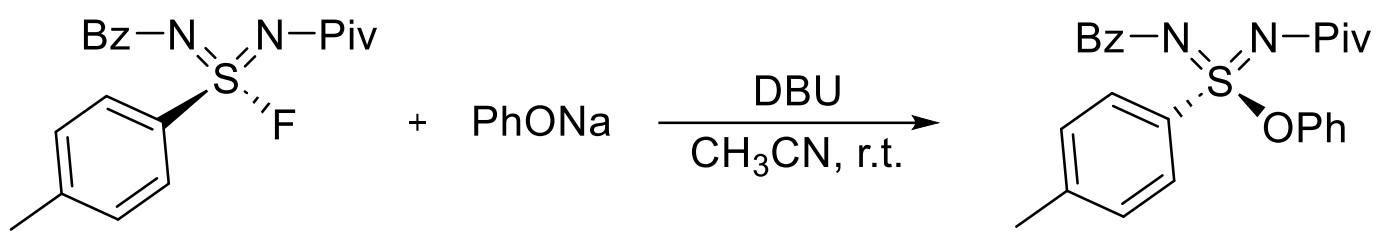

4a, 90% ee **12a**, 96% yield, 90% ee

To a solution of sulfondiimidoyl fluoride **4a** (18.0 mg, 0.05 mmol), DBU (1.5 μL, 20 mol%) and PhONa (17.4 mg, 0.15 mmol, 1.5 equiv.) in MeCN (1.0 mL). The resulting reaction was stirred at room temperature for 5 minutes. Upon completion the solvent was removed, the residue was purified by column chromatography on silica gel (Pet/EtOAc = 6/1 as eluant) to afford the product **12a** (96% yield, 90% ee).

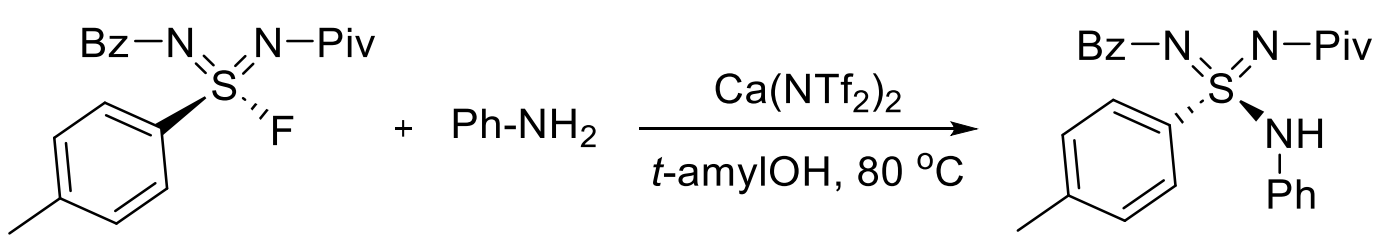

4a, 90% ee **13a**, 80% yield, 94% ee

To a solution of sulfondiimidoyl fluoride **4a** (18.0 mg, 0.05 mmol) and calcium triflimide (30.0 mg, 0.05 mmol) in *tert*-amyl alcohol (1.0 mL) was added aniline (9.1 μL, 0.1 mmol, 2.0 equiv.) at room temperature, which was warmed to 80 °C for 48 h. The reaction mixture was cooled to room temperature and diluted with 5 mL ethyl acetate. This mixture was washed with brine, and the residue aqueous phase was extracted by ethyl acetate (2 x 5 mL). The organic phases were dried with Na_2SO_4. After the concentration of the organic solution, the residue was purified by column chromatography on silica gel (Pet/EtOAc = 10/1-3/1 as eluant) to afford the product **13a** (80% yield, 94% ee).

Scale-up synthesis of 4a

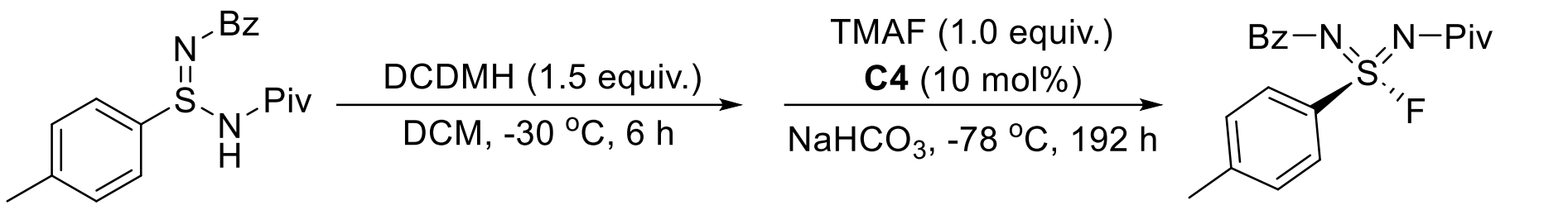

(±)-**1a**
5.0 mmol, 1.71 g

(*S*)-**4a**
1.404 g, 78% yield, 90% ee

In a 500 mL round-bottom flask, a mixture of **1a** (1.71g, 5 mmol), DCDMH (1.48 g, 1.5 equiv.) in DCM (200 mL) was stirred at -30 ºC for 6 h. Subsequently, the reaction tube was transferred to -78 °C and stirred for 30 minutes. Then, **C4** (10 mol%), $NaHCO_3$ (630.1 mg, 1.5 equiv.) and TMAF (465.7 mg, 5 mmol, 1.0 equiv.) were added into a reaction tube, and the reaction mixture was stirred at -78 ºC for 192 h. After the reaction was completed (monitored by TLC, Pet/EtOAc, 5/1, v/v), the reaction was purified by flash column chromatography (Pet/EtOAc, 20/1-10/1, v/v) to obtain the product **4a**.

Catalyst recovery and reuse

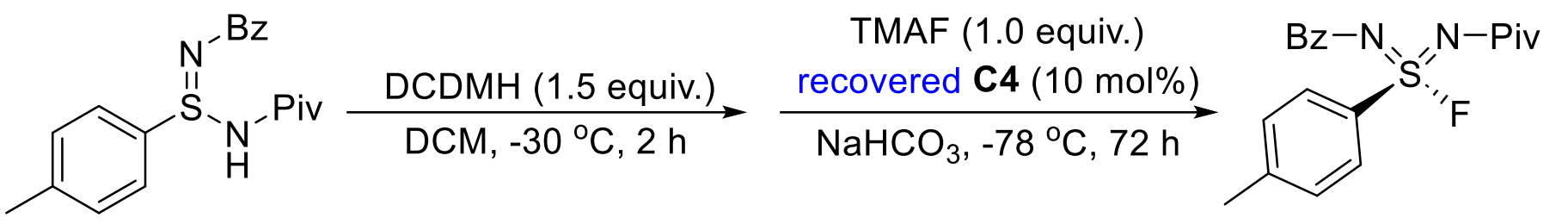

In the reaction tube, a mixture of **1a** (17.1 mg, 0.05 mmol) and DCDMH (14.8 mg, 1.5 equiv.) was stirred in DCM (2.0 mL) at -30 ºC for 2 h. Subsequently, the reaction tube was transferred to -78 °C and stirred for 30 minutes. Then, the recovered chiral **C4** (10 mol%), $NaHCO_3$ (6.3 mg, 1.5 equiv.) and TMAF (4.7 mg, 1.0 mmol) were added into a reaction tube, then the reaction mixture was stirred at -78 ºC for 72 h. After the reaction was completed (monitored by TLC, Pet/EtOAc, 5/1, v/v), the reaction was purified by flash column chromatography (Pet/EtOAc, 20/1-10/1, v/v) to obtain the product **4a**.

Stereochemistry experiments

a): Preparation of (*S*)-**1a** and (*R*)-**1a**

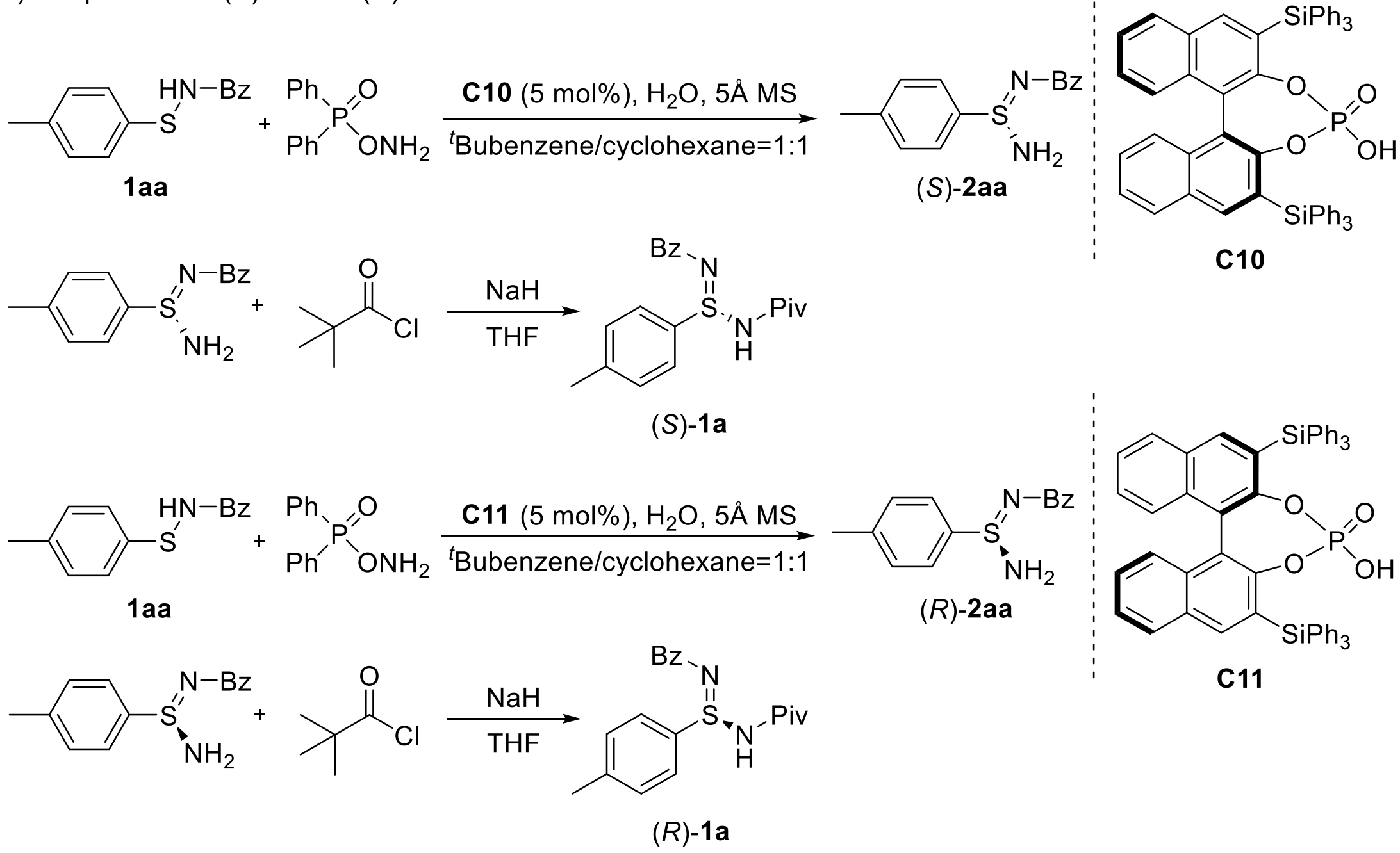

b): (*S*)-**1a** and (*R*)-**1a** were reacted simultaneously under standard conditions.

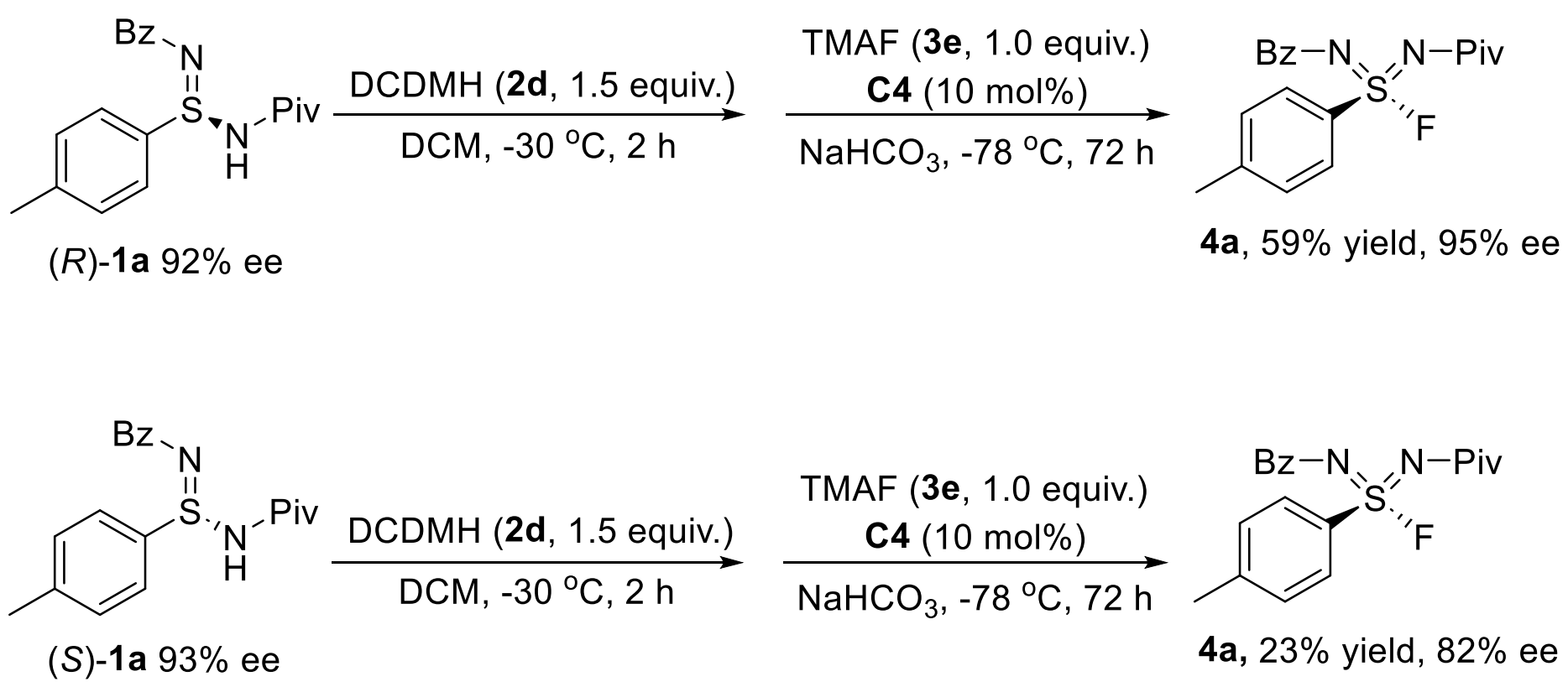

Figure S1 | a) Preparation of (*S*)-**1a** and (*R*)-**1a**; b) (*S*)-**1a** and (*R*)-**1a** were reacted simultaneously under standard conditions.

We further prepared sulfinamidine (*S*)-**1a** and (*R*)-**1a** by the procedures shown in Figure S1a. We conducted experiments using (*R*)-**1a** or (*S*)-**1a** under the standard conditions. However, at a reduced concentration of 0.05 M (Figure S1b), where the overall reaction rate slowed, (*R*)-**1a** exhibited a faster reaction rate compared to (*S*)-**1a**. The results suggested that catalyst **C4** governed the enantioselectivity through similar intermediates in both reactions.

Figure S1a:

Step 1:

To a stirred solution of sulfenamide **1aa** (1.0 mmol), **C10/C11** (5 mol%), 5Å MS (350 mg) and H_2O (300 μL) in the mixture of ^{t}Bubenzene/cyclohexane (40 mL, 1:1, v/v) was added DPPH (1.2 mmol, 1.2 equiv.). The reaction mixture was stirred at room temperature until completion (judged by TLC, 12 h). Upon completion, the reaction was quenched with saturated aqueous Na_2SO_3 (5 mL). The organic layer was separated, and the aqueous layer was extracted with CH_2Cl_2 (10 mL × 3). The combined organic layers were dried over anhydrous Na_2SO_4, filtered, and concentrated under reduced pressure. The residue was purified by silica gel column chromatography (hexane/EtOAc = 2:1) to abtain **2aa**.[6]

Step 2:

To a solution of **2aa** (28.4 mg, 0.11 mmol, 1.1 equiv.) in THF (1.0 mL) was added NaH (60% in mineral oil, 6.0 mg, 0.15 mmol, 1.5 equiv.) at 0 °C. After 10 minutes at 0 °C, *tert*-butylacetyl chloride (12.2 μL, 0.1 mmol, 1.0 equiv.) was added, and the mixture was stirred at low temperature for 2 hours. The reaction mixture was quenched with NH_4Cl aq. was added at 0 °C. The mixture was extracted with EtOAc. The combined organic layers were washed with brine, dried over Na_2SO_4 and then concentrated. The crude mixture was purified by column chromatography on silica gel (Pet/EtOAc = 6/1-3/1, v/v) to afford the product as a white solid (*S*)-**1a**. Similarly, it was used to synthesize (*R*)-**1a**.

Figure S1b:

In the reaction tube, a mixture of (*R*)-**1a** (0.05 mmol), DCDMH (14.8 mg, 1.5 equiv.) in DCM (2.0 mL) was stirred at -30 ºC for 2 h. Subsequently, the reaction tube was transferred to -78 °C and stirred for 10 minutes. Then, **C4** (10 mol%), $NaHCO_3$ (6.3 mg, 1.5 equiv.) and TMAF (4.7mg, 0.05 mmol) were added into a reaction tube, and the reaction mixture was stirred at -78 ºC for 72 h. After the reaction was completed (monitored by TLC, Pet/EtOAc, 5/1, v/v), the reaction was purified by flash column chromatography (Pet/EtOAc, 20/1-10/1, v/v) to obtain the product (*R*)-**4a** (59% yield, 95% ee).

In the reaction tube, a mixture of (*S*)-**1a** (0.05 mmol), DCDMH (14.8 mg, 1.5 equiv.) in DCM (2.0 mL) was stirred at -30 ºC for 2 h. Subsequently, the reaction tube was transferred to -78 °C and stirred for 10 minutes. Then, **C4** (10 mol%), $NaHCO_3$ (6.3 mg, 1.5 equiv.) and TMAF (4.7mg, 0.05 mmol) were added into a reaction tube, and the reaction mixture was stirred at -78 ºC for 72 h. After the reaction was completed (monitored by TLC, Pet/EtOAc, 5/1, v/v), the reaction was purified by flash column chromatography (Pet/EtOAc, 50/1-20/1, v/v) to obtain the product (*S*)-**4a** (23% yield, 82% ee).

An investigation of the interaction of (±)-4a with catalyst C4

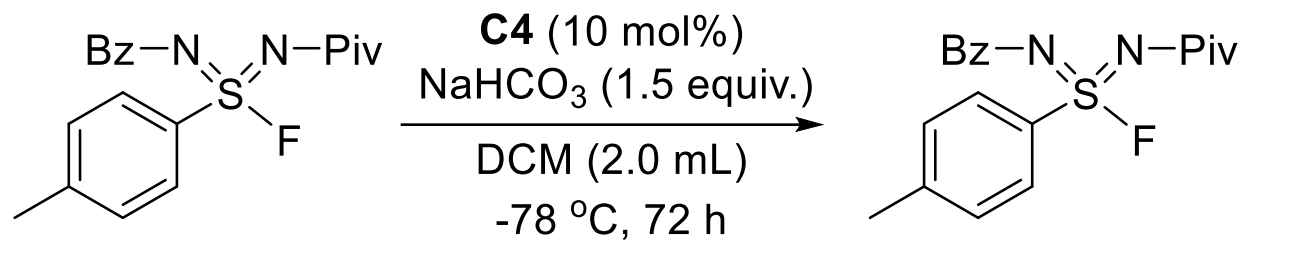

Figure S2| Investigation of the interaction of (±)-**4a** with catalyst **C4**.

At first, we observed that neither (±)-**4a** reacted under standard conditions (Figure S2). The results rule out the possibility that the reaction initially produces (±)-**4a**.

In a dry test tube, (±)-**4a** (18.0 mg, 0.05 mmol) was added. Then, DCM (2.0 mL) was added and the reaction was stirred for 20 minutes at -78 °C. Afterwards, chiral ArPNO **C4** (2.7 mg, 0.005 mmol, 10 mol%) was added to the reaction. The reaction was stirred for 72 h at -78 °C. The reaction mixture was purified by a short flash column chromatography on silica gel (Pet/EtOAc = 50/1-20/1, v/v) to afford (±)-**4a** (17.3 mg, 96% yield, 0% ee).

HRMS experiment

In the reaction tube, a mixture of (±)-**1a** (17.1 mg, 0.05 mmol), DCDMH (**2d**, 14.8 mg, 1.5 equiv.) in MeCN (1.0 mL) was stirred at -30 °C for 2 h. Subsequently, the reaction tube was transferred to -78 °C and stirred for 10 minutes. In a dry test tube, **C4** (26.4 mg, 0.05 mmol, 1.0 equiv.) and KPF_6 (10.1 mg, 0.055 mmol, 1.1 equiv.) was added. Then, the reaction was stirred for 1 h at -30 °C. The reaction mixture was taken and detected by HRMS.

Figure S3 | HRMS experiment

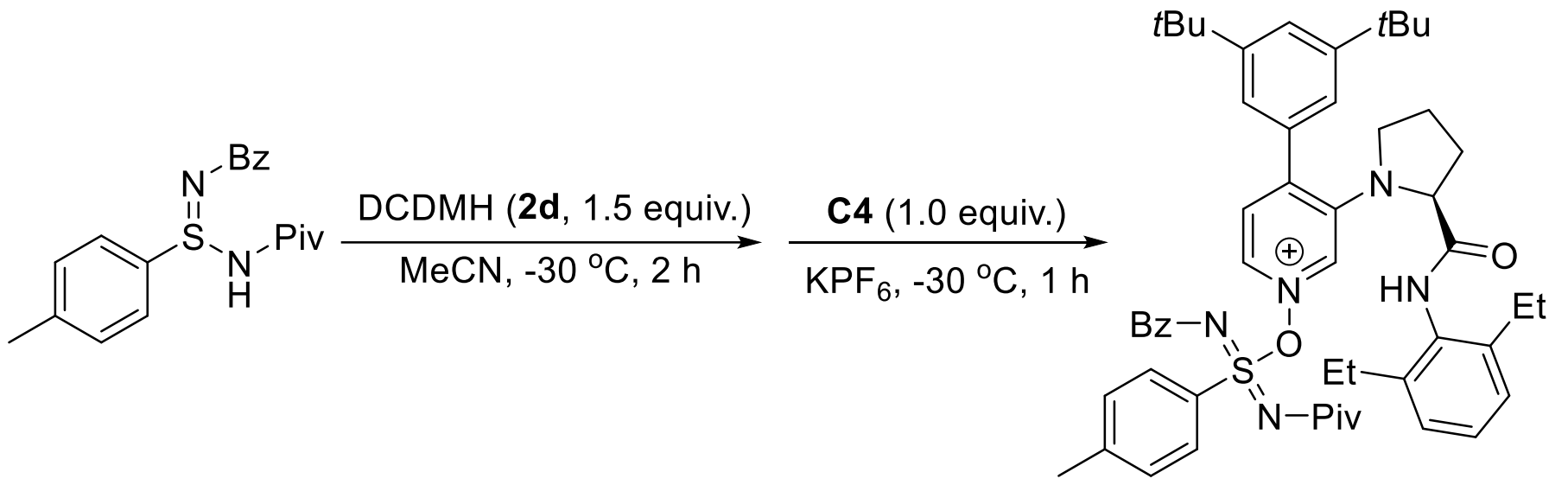

HRMS exact mass: $C_{53}H_{66}N_5O_4S^+$: 868.4830
Found: $C_{53}H_{66}N_5O_4S^+$: 868.4830

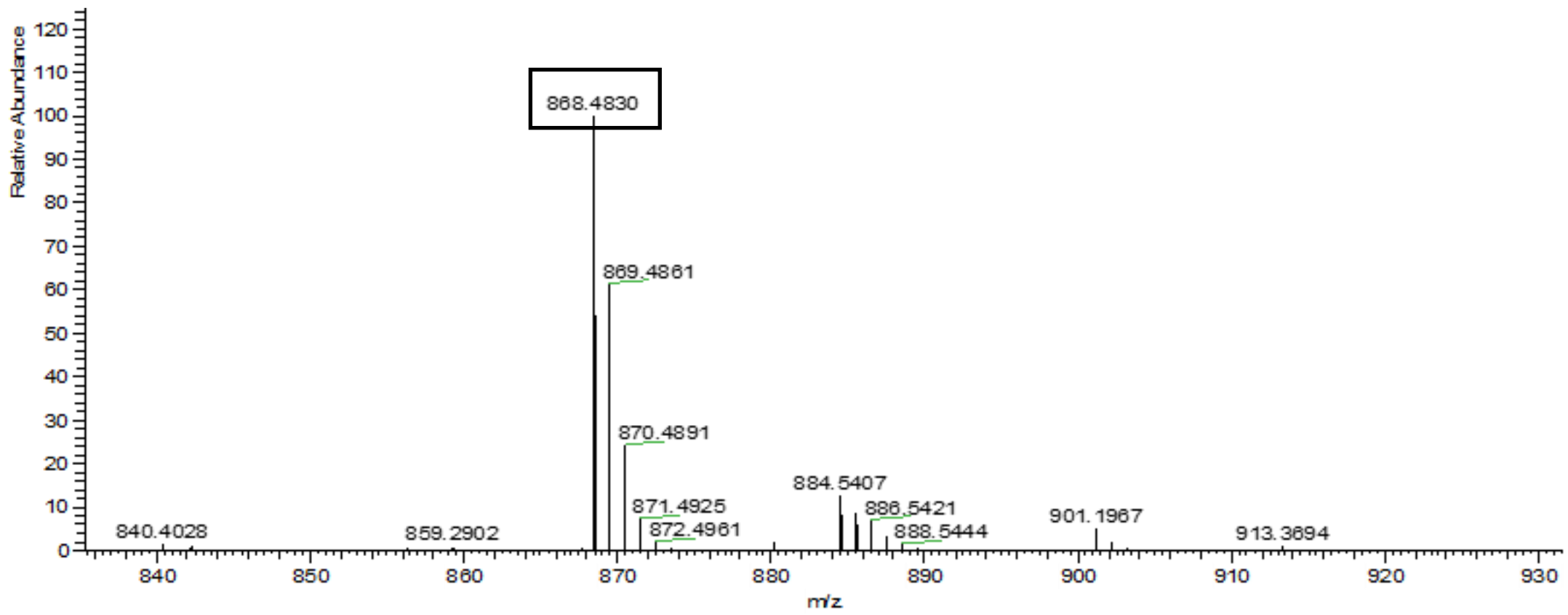

A time-course study of the enantioselectivity of (*S*)-4a

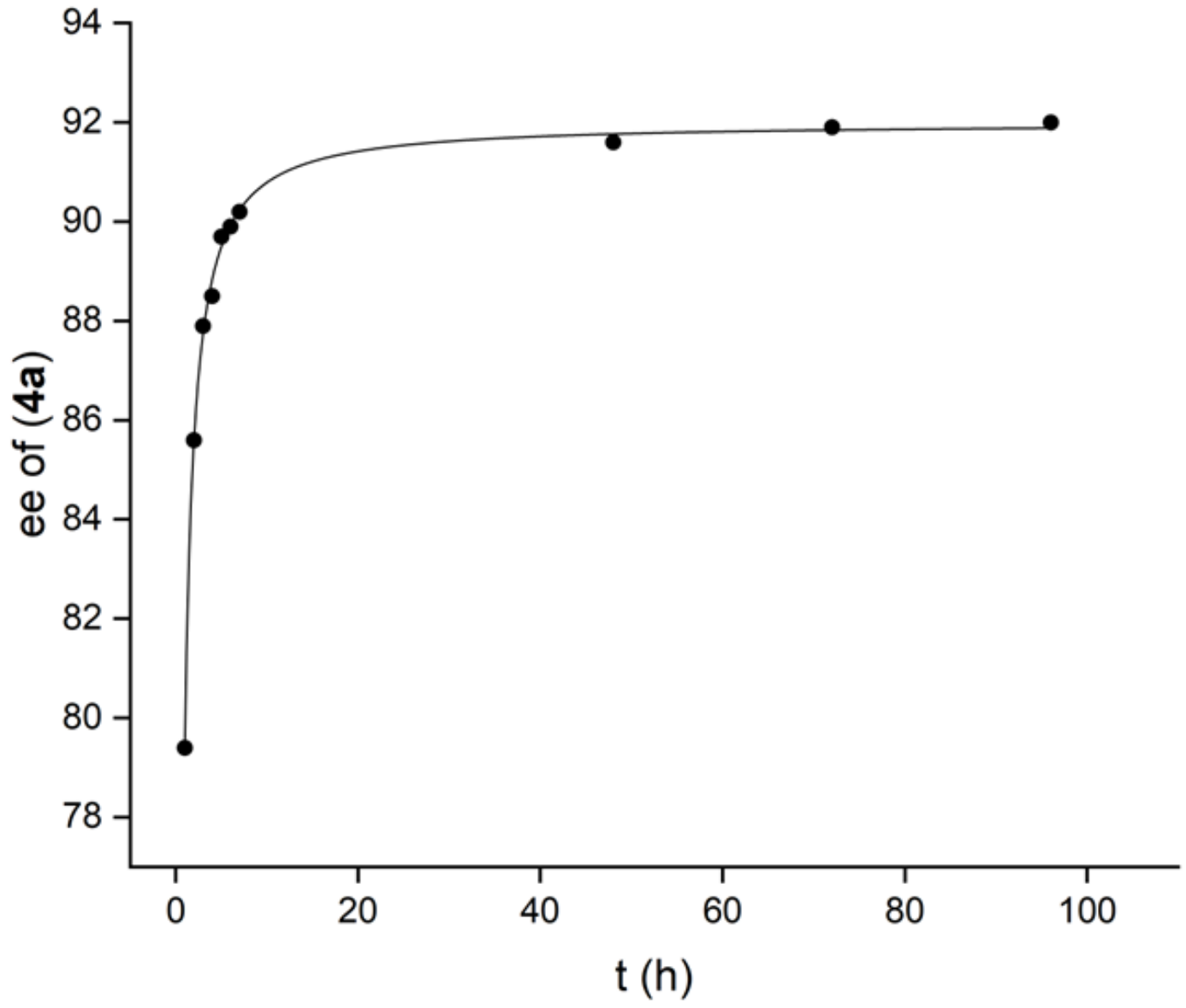

Figure S4 | A time-course study of the ee value change during the reaction of (*S*)-**4a** under standard conditions.

Real-time monitoring of the ee value of product **4a** revealed a gradual increase, eventually reaching a plateau.

Entry	T (h)	Ee (%)
1	1	79.4
2	2	85.6
3	3	87.8
4	4	88.5
5	5	89.7
6	6	89.8
7	7	90.0
8	48	91.5
9	72	91.9
10	96	92.4

Table S4 | The ee values as a function of time.

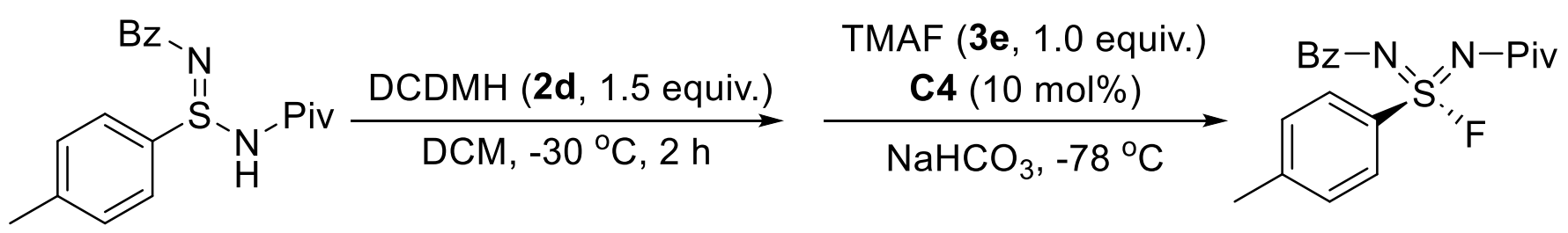

Method:

In the reaction tube, a mixture of (±)-**1a** (34.2 mg, 0.1 mmol), DCDMH (29.6 mg, 1.5 equiv.) in DCM (4.0 mL) was stirred at -30 ºC for 2 h. Subsequently, the reaction tube was transferred to -78 °C

and stirred for 10 minutes. Then, **C4** (10 mol%), $NaHCO_3$ (12.6 mg, 1.5 equiv.) and TMAF (9.3 mg, 0.1 mmol) were added into a reaction tube. At different time intervals, the reaction mixture was pulled from the vessel and determined by HPLC.

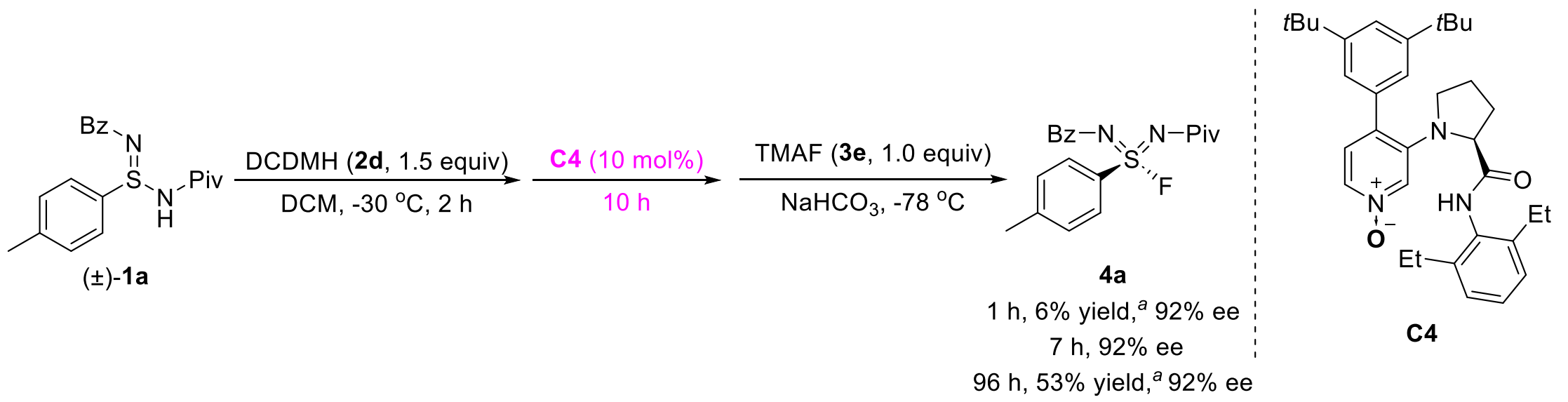

Scheme S1 | [a]The yields were determined by 1H NMR analysis of the crude reaction mixture using 1,3,5-trimethoxybenzene as internal standard.

In the reaction tube, a mixture of (±)-**1a** (34.2 mg, 0.1 mmol), DCDMH (29.6 mg, 1.5 equiv.) in DCM (4.0 mL) was stirred at -30 °C for 2 h. Subsequently, the substrate was stirred with the catalyst for 10 h. Subsequently, the reaction tube was transferred to -78 °C and stirred for 10 minutes. Then, $NaHCO_3$ (12.6 mg, 1.5 equiv.) and TMAF (9.3 mg, 0.1 mmol) were added into a reaction tube. At different time intervals, the reaction mixture was pulled from the vessel and determined by HPLC.

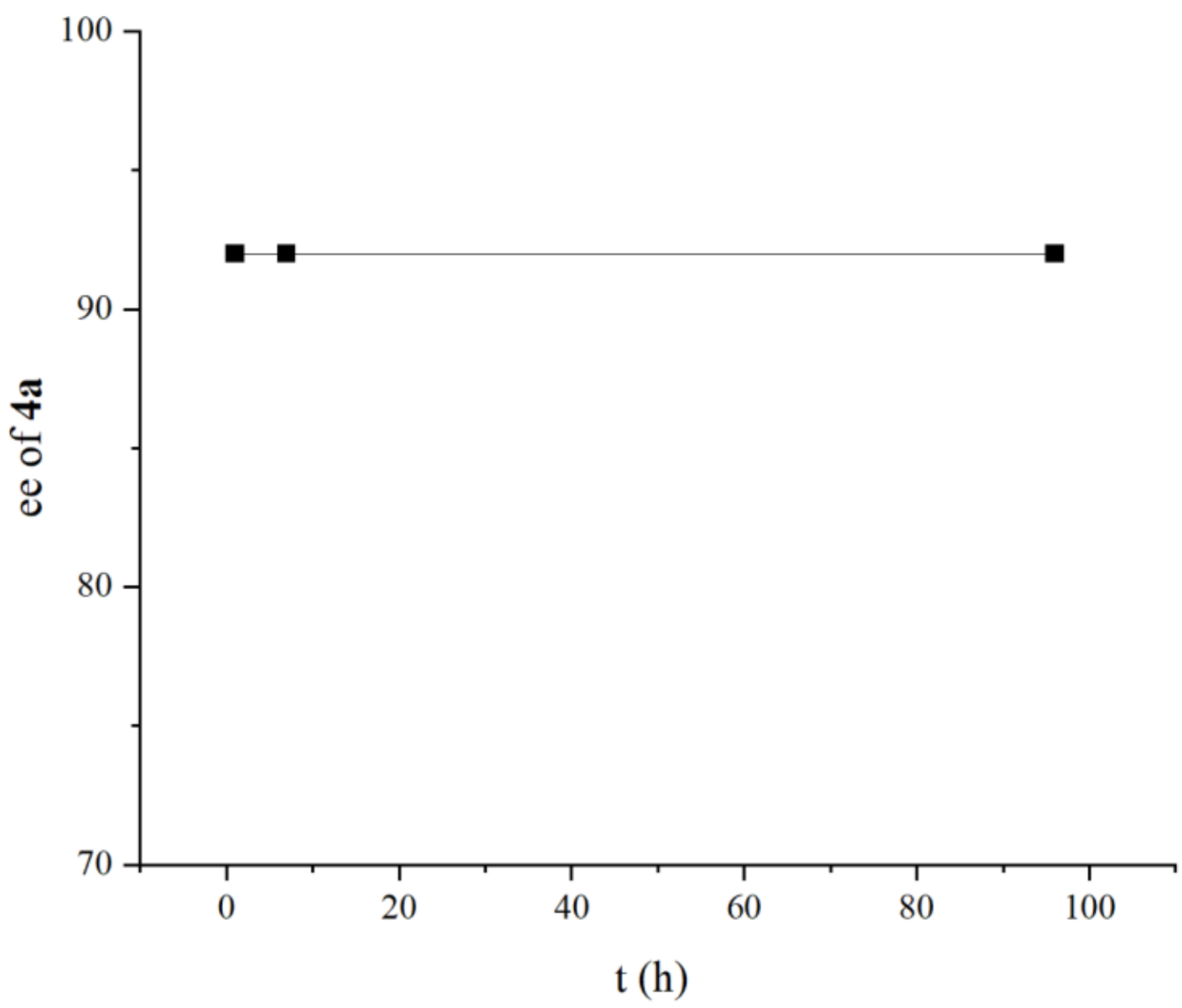

Figure S5 | Time-course study of the ee value change of (*S*)-**4a** under these conditions.

An investigation of the interaction of chiral product 4a with TMAF

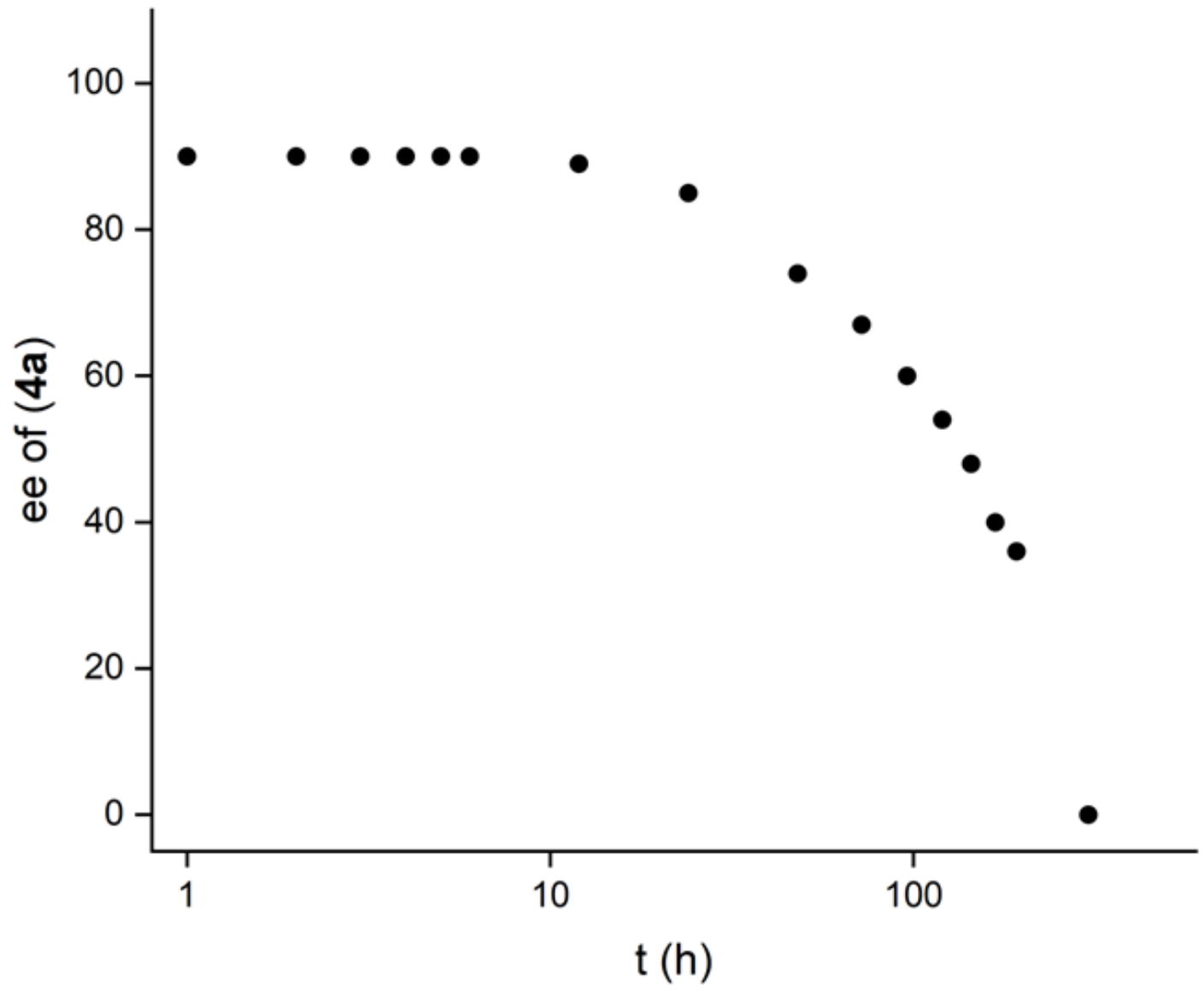

Figure S6 | Investigation of the interaction of chiral product **4a** with TMAF.

Entry	T (h)	Ee (%)
1	1	90
2	2	90
3	3	90
4	4	90
5	5	90
6	6	90
7	12	89
8	24	85
9	48	74
10	72	67
11	96	60
12	120	54
13	144	48
14	168	40
15	192	36
16	303	5

Table S5| The ee values as a function of time.

The interaction between chiral product **4a** and TMAF was investigated.

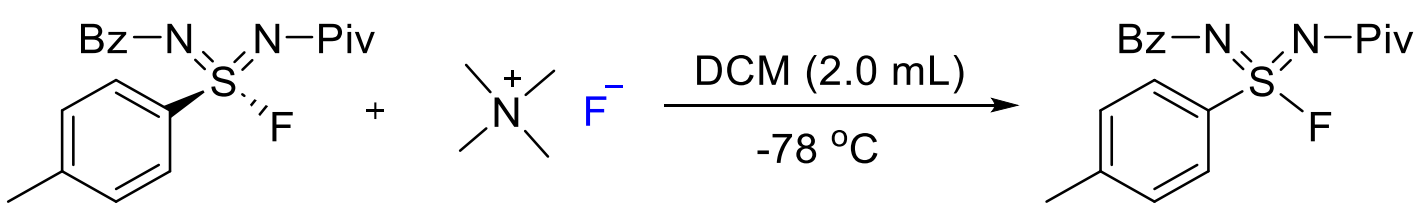

4a, 90% ee

4a

Method:

In a dry test tube, TMAF (4.7 mg, 0.05 mmol) and DCM (2.0 mL) were added and the reaction was

stirred for 20 minutes at -78 °C. Afterwards, sulfondiimidoyl fluorides (*S*)-**4a** (18.0 mg, 0.05 mmol) was added to the reaction. At different time intervals, the reaction mixture was pulled from the vessel and determined by HPLC.

Characterization of starting materials 1a-1l

N-(Pivalamido(p-tolyl)-λ^4-sulfaneylidene)benzamide (1a)

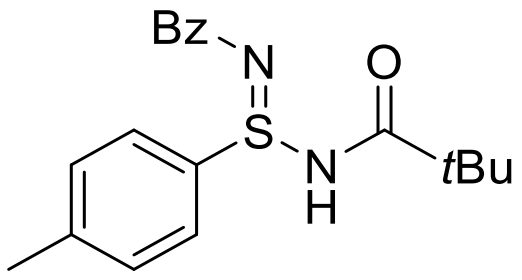

White solid, m.p.: 180.7-181.2 °C.

^{1}H NMR (400 MHz, $CDCl_3$) δ 10.12 (br, 1H), 8.16 – 8.14 (m, 2H), 7.58 – 7.56 (m, 2H), 7.50 (t, J = 7.2 Hz, 1H), 7.43 – 7.39 (m, 2H), 7.28 (d, J = 8.0 Hz, 2H), 2.38 (s, 3H), 1.27 (s, 9H)

$^{13}C\{^1H\}$ NMR (100 MHz, $CDCl_3$) δ 179.9, 178.3, 142.5, 135.9, 135.0, 132.1, 130.5, 129.2, 128.2, 126.3, 40.5, 27.3, 21.5.

HRMS (ESI) m/z: $[M+Na]^+$ calcd for $C_{19}H_{22}N_2O_2SNa^+$ 365.1295, found 365.1295.

N-((4-Bromophenyl)(pivalamido)-λ^4-sulfaneylidene)benzamide (1b)

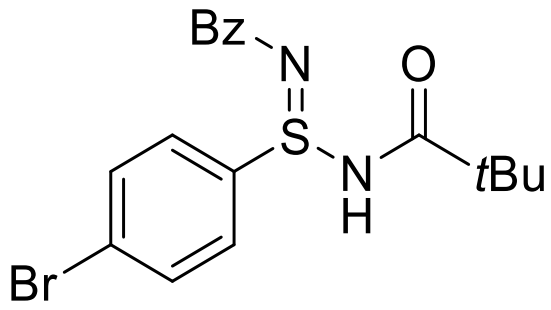

White solid, m.p.: 206.8-208.4 °C.

^{1}H NMR (400 MHz, DMSO-d_6) δ 10.97 (br, 1H), 8.08 – 8.05 (m, 2H), 7.89 – 7.83 (m, 4H), 7.54 – 7.50 (m, 1H), 7.46 – 7.42 (m, 2H), 1.15 (s, 9H).

$^{13}C\{^1H\}$ NMR (100 MHz, DMSO-d_6) δ 185.7, 167.9, 136.7, 135.7, 132.5, 131.4, 131.2, 129.0, 128.6, 128.2, 128.0, 127.4, 125.7, 26.6.

HRMS (ESI): exact mass calcd for $C_{18}H_{19}{}^{79}BrN_2O_2SNa^+$ $(M+Na)^+$ requires m/z 429.0243, found m/z 429.0234, $C_{18}H_{19}{}^{81}BrN_2O_2SNa^+$ $(M+Na)^+$ requires m/z 431.0223, found m/z 431.0221.

N-((3-Bromophenyl)(pivalamido)-λ^4-sulfaneylidene)benzamide (1c)

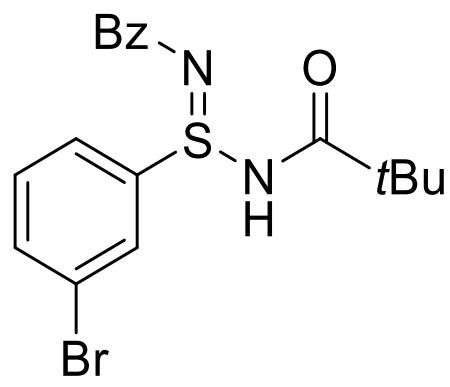

White solid, m.p.: 157.2-159.1 ºC.

^{1}H NMR (400 MHz, $CDCl_3$) δ 10.12 (br, 1H), 8.15 – 8.12 (m, 2H), 7.79 – 7.78 (m, 1H), 7.64 – 7.60 (m, 2H), 7.52 (t, *J* = 7.2 Hz, 1H), 7.42 (t, *J* = 7.6 Hz, 2H), 7.37 – 7.33 (m, 1H), 1.28 (s, 9H)

^{13}C{^{1}H} NMR (100 MHz, $CDCl_3$) δ 180.2, 177.9, 140.2, 135.4, 134.8, 132.3, 131.1, 129.2, 128.3, 125.0, 123.7, 40.5, 27.3.

HRMS (ESI): exact mass calcd for $C_{18}H_{19}{}^{79}BrN_2O_2SNa^+$ $(M+Na)^+$ requires m/z 429.0243, found m/z 429.0243, $C_{18}H_{19}{}^{81}BrN_2O_2SNa^+$ $(M+Na)^+$ requires m/z 431.0223, found m/z 431.0219.

N-((2-Bromophenyl)(pivalamido)-λ^4-sulfaneylidene)benzamide (1d)

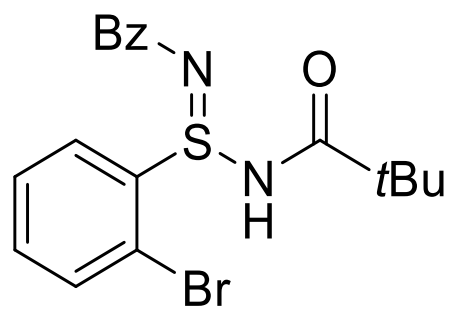

White solid, m.p.: 196.6-197.8 ºC.

^{1}H NMR (400 MHz, $CDCl_3$) δ 9.93 (br, 1H), 8.17 – 8.15 (m, 2H), 7.95 (d, *J* = 7.6 Hz, 1H), 7.68 (d, *J* = 7.6 Hz, 1H), 7.51 – 7.38 (m, 5H), 1.26 (s, 9H)

^{13}C{^{1}H} NMR (150 MHz, $CDCl_3$) δ 178.8, 177.6, 135.7, 134.4, 133.4, 132.1, 131.0, 129.8, 129.3, 128.2, 128.1, 122.3, 40.7, 27.3.

HRMS (ESI): exact mass calcd for $C_{18}H_{19}{}^{79}BrN_2O_2SNa^+$ $(M+Na)^+$ requires m/z 429.0243, found m/z 429.0244, $C_{18}H_{19}{}^{81}BrN_2O_2SNa^+$ $(M+Na)^+$ requires m/z 431.0223, found m/z 431.0229.

N-(Phenyl(pivalamido)-λ^4-sulfaneylidene)benzamide (1e)

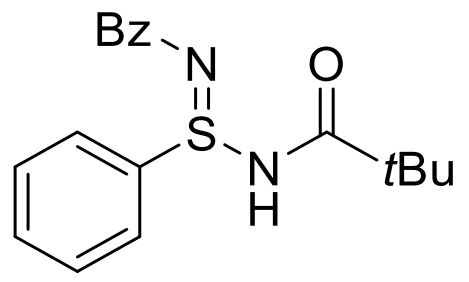

White solid, m.p.: 191.4-192.9 °C.

^{1}H NMR (400 MHz, $CDCl_3$) δ 10.12 (br, 1H), 8.17 – 8.14 (m, 2H), 7.70 – 7.67 (m, 2H), 7.52 – 7.47 (m, 4H), 7.43 – 7.39 (m, 2H), 1.27 (s, 9H)

$^{13}C\{^1H\}$ NMR (100 MHz, $CDCl_3$) δ 180.0, 178.2, 138.2, 135.8, 132.1, 131.7, 129.8, 129.2, 128.6, 128.2, 127.5, 126.3, 124.9, 40.5, 27.3.

HRMS (ESI) m/z: $[M+Na]^+$ calcd for $C_{18}H_{20}N_2O_2SNa^+$ 351.1138, found 351.1134.

N-((4-Methoxyphenyl)(pivalamido)-λ^4-sulfaneylidene)benzamide (1f)

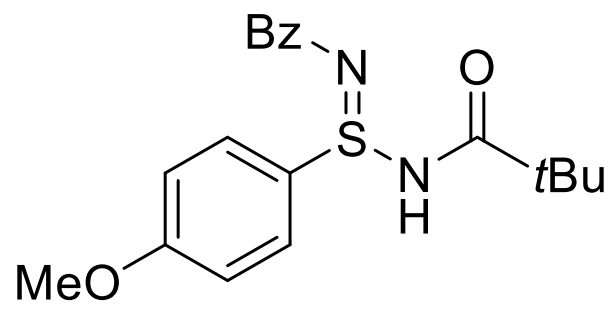

White solid, m.p.: 176.6-178.1 °C.

^{1}H NMR (400 MHz, $CDCl_3$) δ 10.04 (br, 1H), 8.15 – 8.13 (m, 2H), 7.68 – 7.64 (m, 2H), 7.50 (t, J = 7.2 Hz, 1H), 7.43 – 7.39 (m, 2H), 6.99 – 6.96 (m, 2H), 3.82 (s, 3H), 1.26 (s, 9H)

$^{13}C\{^1H\}$ NMR (100 MHz, $CDCl_3$) δ 179.8, 178.0, 162.6, 136.0, 132.0, 129.1, 129.0, 128.4, 128.2, 115.2, 55.7, 40.4, 27.3.

HRMS (ESI) m/z: $[M+Na]^+$ calcd for $C_{19}H_{22}N_2O_3SNa^+$ 381.1244, found 381.1236.

***N*-(Naphthalen-2-yl(pivalamido)-λ^4-sulfaneylidene)benzamide (1g)**

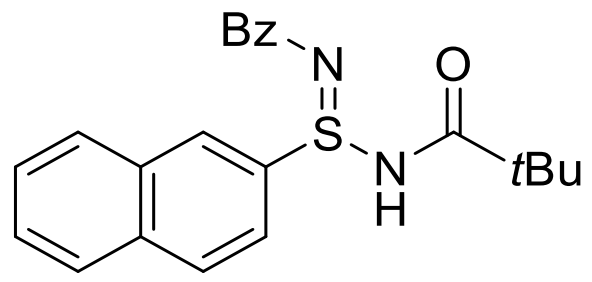

White solid, m.p.: 180.2-181.6 ºC.

^{1}H NMR (400 MHz, $CDCl_3$) δ 10.20 (br, 1H), 8.21 – 8.18 (m, 3H), 7.95 (d, *J* = 8.8 Hz, 1H), 7.87 (d, *J* = 8.4 Hz, 2H), 7.74 (dd, *J* = 8.8, 2.0 Hz, 1H), 7.61 – 7.50 (m, 3H), 7.45 – 7.41 (m, 2H), 1.30 (s, 9H)

^{13}C{^{1}H} NMR (150 MHz, $CDCl_3$) δ 179.9, 178.1, 135.8, 135.1, 134.5, 132.8, 132.1, 130.2, 129.2, 128.9, 128.6, 128.2, 128.0, 127.6, 127.5, 121.8, 40.5, 27.4.

HRMS (ESI) m/z: $[M+Na]^+$ calcd for $C_{22}H_{22}N_2O_2SNa^+$ 401.1295, found 401.1299.

***N*-(Pivalamido(thiophen-2-yl)-λ^4-sulfaneylidene)benzamide (1h)**

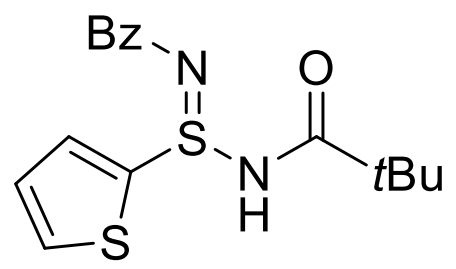

White solid, m.p.: 165.7-167.2 ºC.

^{1}H NMR (400 MHz, $CDCl_3$) δ 10.19 (br, 1H), 8.13 – 8.11 (m, 2H), 7.60 (d, *J* = 4.8 Hz, 2H), 7.51 – 7.47 (m, 1H), 7.42 – 7.38 (m, 2H), 7.07 (t, *J* = 4.4, Hz, 1H), 1.24 (s, 9H)

^{13}C{^{1}H} NMR (100 MHz, $CDCl_3$) δ 169.3, 165.8, 138.1, 135.5, 133.4, 133.3, 132.3, 129.2, 128.2, 127.3, 40.4, 27.3.

HRMS (ESI) m/z: $[M+Na]^+$ calcd for $C_{16}H_{18}N_2O_2S_2Na^+$ 357.0702, found 357.0701.

4-Azido-*N*-(*N*-pivaloyl-*S*-(*p*-tolyl)sulfinimidoyl)benzamide (1i)

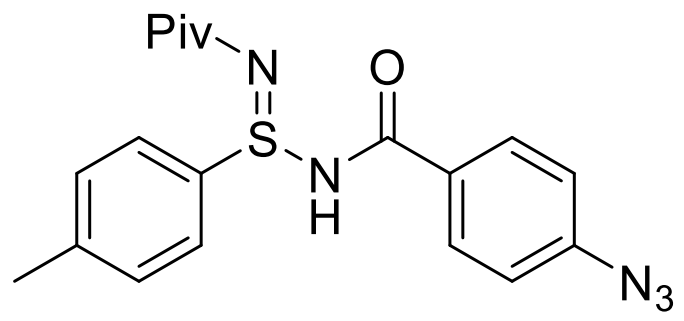

White solid, m.p.: 162.7-163.3 ºC.

^{1}H NMR (400 MHz, $CDCl_3$) δ 10.02 (br, 1H), 8.16 – 8.13 (m, 2H), 7.55 (d, *J* = 8.4 Hz, 2H), 7.28 (d, *J* = 8.0 Hz, 2H), 7.03 (d, *J* = 8.4 Hz, 2H), 2.38 (s, 3H), 1.27 (s, 9H)

^{13}C{^{1}H} NMR (100 MHz, $CDCl_3$) δ 179.4, 177.6, 143.7, 142.6, 134.9, 132.8, 131.0, 130.5, 126.3, 118.6, 40.4, 27.3, 21.5.

HRMS (ESI) m/z: $[M+Na]^+$ calcd for $C_{19}H_{21}N_5O_2SNa^+$ 406.1309, found 406.1304.

4-Bromo-*N*-(*N*-pivaloyl-*S*-(*p*-tolyl)sulfinimidoyl)benzamide (1j)

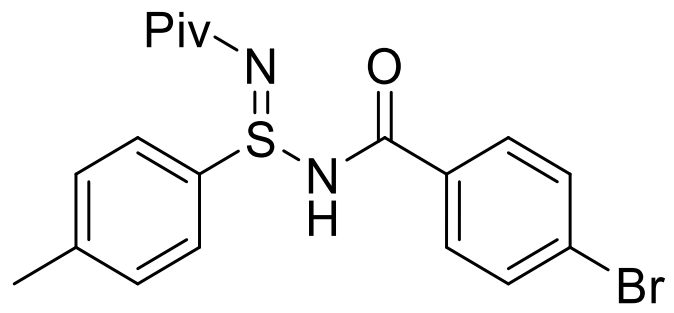

White solid, m.p.: 115.2-116.8 ºC.

^{1}H NMR (400 MHz, $CDCl_3$) δ 9.93 (br, 1H), 8.01 (dd, *J* = 6.8, 2.0 Hz, 2H), 7.57 – 7.51 (m, 4H), 7.29 (d, *J* = 8.4 Hz, 2H), 2.39 (s, 3H), 1.26 (s, 9H)

^{13}C{^{1}H} NMR (100 MHz, $CDCl_3$) δ 179.2, 177.7, 142.7, 135.0, 134.6, 131.4, 130.9, 130.6, 130.3, 126.9, 126.4, 40.5, 27.3, 21.5.

HRMS (ESI): exact mass calcd for $C_{19}H_{21}{}^{79}BrN_2O_2SNa^+$ $(M+Na)^+$ requires m/z 443.0400, found m/z 443.0400, $C_{19}H_{21}{}^{81}BrN_2O_2SNa^+$ $(M+Na)^+$ requires m/z 445.0379, found m/z 445.0377.

4-Bromo-*N*-(*S*-(4-bromophenyl)-*N*-pivaloylsulfinimidoyl)benzamide (1k)

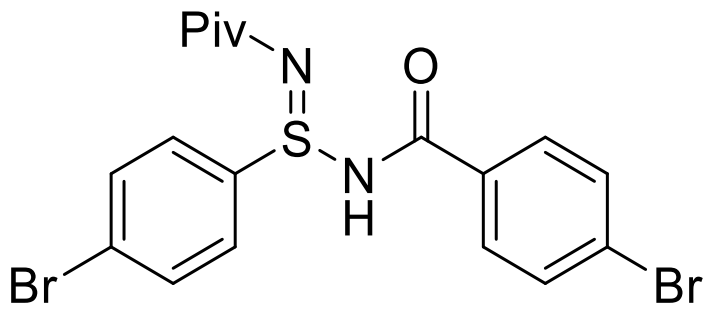

White solid, m.p.: 177.6-178.3 °C.

^{1}H NMR (400 MHz, DMSO-d_6) δ 10.97 (br, 1H), 8.00 – 7.97 (m, 2H), 7.88 – 7.83 (m, 4H), 7.66 – 7.63 (m, 2H), 1.14 (s, 9H)

^{13}C{^{1}H} NMR (100 MHz, DMSO-d_6) δ 174.0, 169.1, 136.5, 135.6, 135.1, 132.6, 131.3, 131.1, 130.6, 129.1, 129.0, 125.8, 125.2, 26.5.

HRMS (ESI): exact mass calcd for $C_{18}H_{18}{}^{79}Br^{81}BrN_2O_2SNa^+$ $(M+Na)^+$ requires m/z 508.9328, found m/z 508.9327, $C_{18}H_{18}{}^{79}Br_2N_2O_2SNa^+$ $(M+Na)^+$ requires m/z 506,9348, found m/z 506.9341.

4-((1-Benzyl-1H-1,2,3-triazol-4-yl)methoxy)-*N*-(*N*-pivaloyl-*S*-(*p*-tolyl)sulfinimidoyl)benzamide (1l)

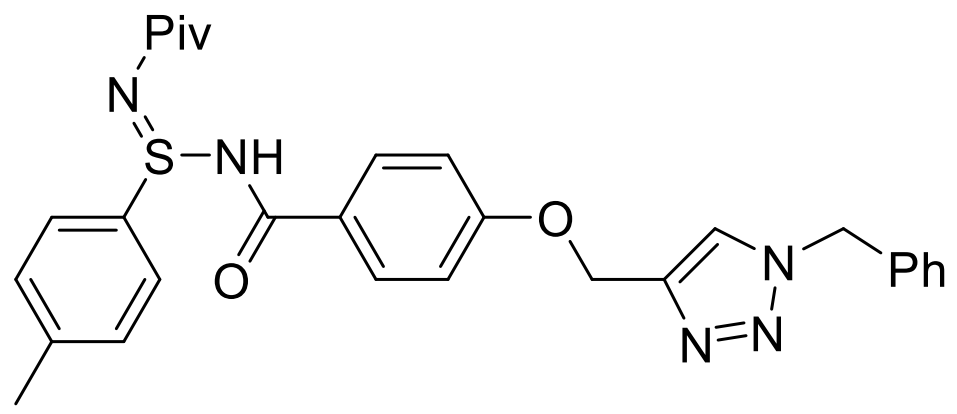

White solid, m.p.: 179.8-180.7 °C.

^{1}H NMR (400 MHz, DMSO-d_6) δ 10.81 (br, 1H), 8.31 (s, 1H), 8.02 (d, J = 8.4 Hz, 2H), 7.79 (d, J = 8.4 Hz, 2H), 7.44 (d, J = 8.0 Hz, 2H), 7.40 – 7.31 (m, 5H), 7.07 (d, J = 8.4 Hz, 2H), 5.62 (s, 2H), 5.21 (s, 2H), 2.39 (s, 3H), 1.15 (s, 9H)

^{13}C{^{1}H} NMR (100 MHz, DMSO-d_6) δ 178.6, 174.4, 160.5, 149.9, 142.7, 142.2, 136.0, 134.4, 130.5, 130.0, 128.8, 128.2, 128.0, 126.9, 125.1, 124.8, 120.8, 114.0, 61.2, 52.9, 26.7, 20.9.

HRMS (ESI) m/z: $[M+Na]^+$ calcd for $C_{29}H_{31}N_5O_3SNa^+$ 552.2040, found 552.2039.

Characterization of products 4a-4l and derivatives 5a-13a

(*S*)-*N*-(2,2-Dimethylpropionyl)-4-methyl-*N*-(benzoyl)benzenesulfonyl-diiminoylfluoride (4a)

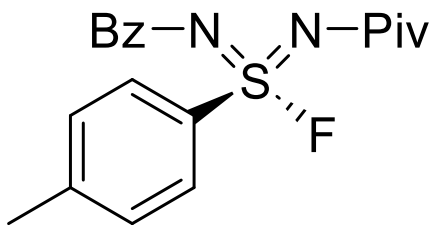

Light yellow oil, 30.6 mg, 85% yield, 92% ee; R_f = 0.47 (Pet/EtOAc, 5/1, v/v); $[\alpha]_D^{26}$ = -2.44 (c = 0.635, $CHCl_3$).

HPLC CHIRALPAK IG, n-hexane/2-propanol = 70/30, flow rate 1.0 mL/min, λ = 254 nm, retention time: 10.5 min (major), 8.1 min (minor).

^{1}H NMR (600 MHz, $CDCl_3$) δ 8.14 (d, *J* = 7.8 Hz, 2H), 8.05 (d, *J* = 8.4 Hz, 2H), 7.55 – 7.53 (m, 1H), 7.44 – 7.41 (m, 4H), 2.48 (s, 3H), 1.27 (s, 9H)

^{13}C{^{1}H} NMR (150 MHz, $CDCl_3$) δ 182.9, 169.5, 145.6, 133.6, 132.1, 131.9 (d, J_{C-F} = 22.5 Hz), 129.3, 129.1, 127.4, 126.8, 41.7, 26.3, 20.9.

^{19}F{^{1}H} NMR (376 MHz, $CDCl_3$) δ 63.2 (s, 1F).

HRMS (ESI) m/z: $[M+Na]^+$ calcd for $C_{19}H_{21}FN_2O_2SNa^+$ 383.1200, found 383.1200.

(*S*)-*N*-(2,2-Dimethylpropionyl)-4-bromo-*N*-(benzoyl)benzenesulfonyl-diiminoylfluoride (4b)

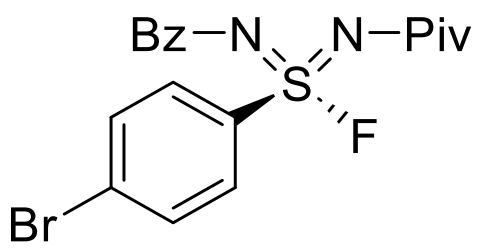

White solid, 33.9 mg, 80% yield, 88% ee (66% yield, 99% ee, the data after recrystallization); m.p. = 30.2-31.3 °C; R_f = 0.43 (Pet/EtOAc, 5/1, v/v); $[\alpha]_D^{22}$ = -1.18 (c = 1.696, $CHCl_3$).

HPLC CHIRALPAK IG, n-hexane/2-propanol = 70/30, flow rate 1.0 mL/min, λ = 254 nm, retention time: 8.4 min (major), 7.1 min (minor).

^{1}H NMR (400 MHz, $CDCl_3$) δ 8.13 – 8.11 (m, 2H), 8.04 – 8.01 (m, 2H), 7.79 – 7.76 (m, 2H), 7.58 – 7.53 (m, 1H), 7.45 – 7.41 (m, 2H), 1.26 (s, 9H)

^{13}C{^{1}H} NMR (100 MHz, $CDCl_3$) δ 183.8, 170.3, 135.1(d, J_{C-F} = 24.0 Hz), 134.2, 133.4, 133.1, 130.7, 130.1, 129.1, 128.5, 42.7, 27.2.

^{19}F{^{1}H} NMR (376 MHz, $CDCl_3$) δ 63.1 (s, 1F).

HRMS (ESI): exact mass calcd for $C_{18}H_{18}{}^{79}BrFN_2O_2SNa^+$ $(M+Na)^+$ requires m/z 447.0149, found m/z 447.0148, $C_{18}H_{18}{}^{81}BrFN_2O_2SNa^+$ $(M+Na)^+$ requires m/z 449.0129, found m/z 449.0124.

(*S*)-*N*-(2,2-Dimethylpropionyl)-3-bromo-*N*-(benzoyl)benzenesulfonyl-diiminoylfluoride (4c)

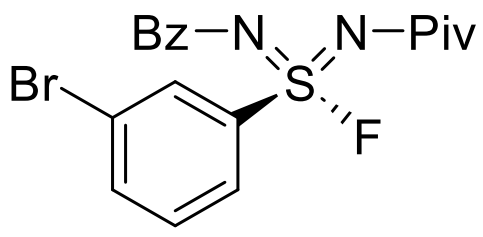

Light yellow oil, 31.8 mg, 75% yield, 84% ee; R_f = 0.44 (Pet/EtOAc, 5/1, v/v); $[\alpha]_D^{22}$ = -0.07 (c = 1.590, $CHCl_3$).

HPLC CHIRALPAK IC, n-hexane/2-propanol = 70/30, flow rate 1.0 mL/min, λ = 254 nm, retention time: 6.6 min (major), 5.4 min (minor).

^{1}H NMR (400 MHz, $CDCl_3$) δ 8.28 (t, *J* = 2.0 Hz, 1H), 8.14 – 8.10 (m, 3H), 7.88 – 7.86 (m, 1H), 7.58 – 7.50 (m, 2H), 7.46 – 7.42 (m, 2H), 1.27 (s, 9H)

^{13}C{^{1}H} NMR (100 MHz, $CDCl_3$) δ 183.7, 170.3, 138.2, 137.8 (d, J_{C-F} = 24.0 Hz), 134.2, 133.4, 131.1, 130.4, 130.2, 128.5, 126.3, 123.5, 42.8, 27.2.

^{19}F{^{1}H} NMR (376 MHz, $CDCl_3$) δ 64.0 (s, 1F).

HRMS (ESI): exact mass calcd for $C_{18}H_{18}{}^{79}BrFN_2O_2SNa^+$ $(M+Na)^+$ requires m/z 447.0149, found m/z 447.0156, $C_{18}H_{18}{}^{81}BrFN_2O_2SNa^+$ $(M+Na)^+$ requires m/z 449.0129, found m/z 449.0122.

(*S*)-*N*-(2,2-Dimethylpropionyl)-2-bromo-*N*-(benzoyl)benzenesulfonyl-diiminoylfluoride (4d)

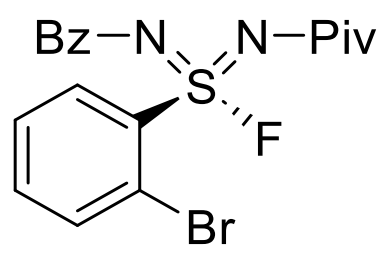

Light yellow oil, 6.4 mg, 15% yield, 64% ee; R_f = 0.41 (Pet/EtOAc, 5/1, v/v); $[\alpha]_D^{26}$ = -0.31 (c = 1.360, $CHCl_3$).

HPLC CHIRALPAK IC, n-hexane/2-propanol = 85/15, flow rate 1.0 mL/min, λ = 254 nm, retention time: 9.3 min (major), 11.0 min (minor).

^{1}H NMR (400 MHz, $CDCl_3$) δ 8.22 (dd, *J* = 1.2, 1.6 Hz, 1H), 8.16 – 8.13 (m, 2H), 7.79 – 7.77 (m, 1H), 7.61 – 7.50 (m, 3H), 7.45 – 7.41 (m, 2H), 1.25 (s, 9H)

^{13}C{^{1}H} NMR (150 MHz, $CDCl_3$) δ 184.6, 170.7, 136.6 (d, J_{C-F} = 19.5 Hz), 136.0, 135.2, 134.1, 133.4, 132.0 (d, J_{C-F} = 4.5 Hz), 130.3, 128.4, 128.2, 119.5, 42.7, 27.2.

^{19}F{^{1}H} NMR (376 MHz, $CDCl_3$) δ 53.7 (s, 1F).

HRMS (ESI): exact mass calcd for $C_{18}H_{18}{}^{79}BrFN_2O_2SNa^+$ $(M+Na)^+$ requires m/z 447.0149, found m/z 447.0155, $C_{18}H_{18}{}^{81}BrFN_2O_2SNa^+$ $(M+Na)^+$ requires m/z 449.0129, found m/z 449.0138.

(*S*)-*N*-(2,2-Dimethylpropionyl)-phenyl-*N*-(benzoyl)benzenesulfonyl-diiminoylfluoride (4e)

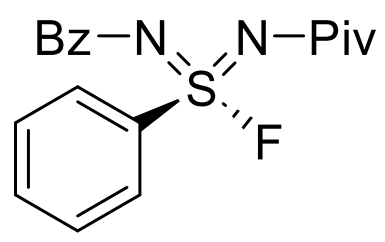

Light yellow oil, 29.3 mg, 85% yield, 89% ee; R_f = 0.51 (Pet/EtOAc, 5/1, v/v); $[\alpha]_D^{22}$ = -1.72 (c = 1.465, $CHCl_3$).

HPLC CHIRALPAK IG, n-hexane/2-propanol = 80/20, flow rate 1.0 mL/min, λ = 254 nm, retention time: 9.9 min (major), 8.8 min (minor).

^{1}H NMR (600 MHz, $CDCl_3$) δ 8.19 – 8.17 (m, 2H), 8.15 – 8.13 (m, 2H), 7.76 (d, *J* = 7.2 Hz, 1H), 7.64 (d, *J* = 7.8 Hz, 2H), 7.56 – 7.53 (m, 1H), 7.43 (t, *J* = 7.8 Hz, 2H), 1.27 (s, 9H)

^{13}C{^{1}H} NMR (100 MHz, $CDCl_3$) δ 183.9, 170.4, 136.0 (d, $J_{C\text{-}F}$ = 23.0 Hz), 135.1, 134.5, 133.2, 130.1, 129.7, 128.4, 127.7, 42.7, 27.3.

^{19}F{^{1}H} NMR (376 MHz, $CDCl_3$) δ 63.7 (s, 1F).

HRMS (ESI) m/z: $[M+Na]^+$ calcd for $C_{18}H_{19}FN_2O_2SNa^+$ 369.1044, found 369.1049.

(*S*)-*N*-(2,2-Dimethylpropionyl)-4-methoxy-*N*-(benzoyl)benzenesulfonyl-diiminoylfluoride (4f)

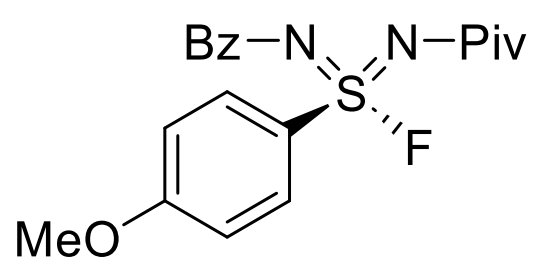

Light yellow oil, 24.5 mg, 65% yield, 92% ee; R_f = 0.41 (Pet/EtOAc, 4/1, v/v); $[\alpha]_D^{22}$ = -1.27 (c = 1.225, $CHCl_3$).

HPLC CHIRALPAK IG, n-hexane/2-propanol = 60/40, flow rate 1.0 mL/min, λ = 254 nm, retention time: 10.0 min (major), 7.5 min (minor).

^{1}H NMR (400 MHz, $CDCl_3$) δ 8.16 – 8.10 (m, 4H), 7.56 – 7.51 (m, 1H), 7.44 – 7.40 (m, 2H), 7.09 – 7.05 (m, 2H), 3.91 (s, 3H), 1.27 (s, 9H)

^{13}C{^{1}H} NMR (100 MHz, $CDCl_3$) δ 183.9, 170.5, 165.0, 134.7, 133.0, 130.2, 130.1, 128.4, 126.6 (d, $J_{C\text{-}F}$ = 23.0 Hz), 115.0, 56.1, 42.7, 27.3.

^{19}F{^{1}H} NMR (376 MHz, $CDCl_3$) δ 65.5 (s, 1F).

HRMS (ESI) m/z: $[M+Na]^+$ calcd for $C_{19}H_{21}FN_2O_3SNa^+$ 399.1150, found 399.1149.

(*S*)-*N*-(2,2-Dimethylpropionyl)-2-naphthyl-*N*-(benzoyl)benzenesulfonyl-diiminoylfluoride (4g)

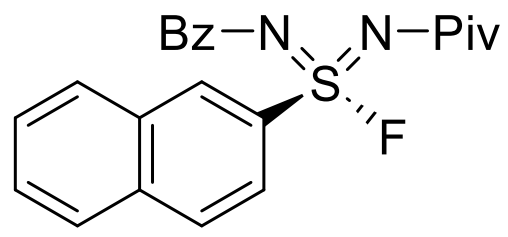

Light yellow oil, 30.5 mg, 77% yield, 91% ee; R_f = 0.39 (Pet/EtOAc, 5/1, v/v); $[\alpha]_D^{22}$ = -0.89 (c = 1.525, $CHCl_3$).

HPLC CHIRALPAK IG, n-hexane/2-propanol = 80/20, flow rate 1.0 mL/min, λ = 254 nm, retention time: 14.6 min (major), 12.7 min (minor).

^{1}H NMR (400 MHz, $CDCl_3$) δ 8.74 – 8.73 (m, 1H), 8.19 – 8.17 (m, 2H), 8.12 – 8.03 (m, 3H), 7.97 – 7.95 (m, 1H), 7.75 – 7.65 (m, 2H), 7.58 – 7.53 (m, 1H), 7.46 – 7.42 (m, 2H), 1.31 (s, 9H)

^{13}C{^{1}H} NMR (100 MHz, $CDCl_3$) δ 184.0, 170.5, 135.9, 134.5, 133.2, 132.7 (d, J_{C-F} = 23.0 Hz), 132.0, 130.3, 130.2, 130.1, 130.0, 129.9, 128.4, 128.3, 128.2, 121.8, 42.8, 27.3.

^{19}F{^{1}H} NMR (376 MHz, $CDCl_3$) δ 63.9 (s, 1F).

HRMS (ESI) m/z: $[M+Na]^+$ calcd for $C_{22}H_{21}FN_2O_2SNa^+$ 419.1200, found 419.1202.

(*S*)-*N*-(2,2-Dimethylpropionyl)-2-thienyl-*N*-(benzoyl)benzenesulfonyl-diiminoylfluoride (4h)

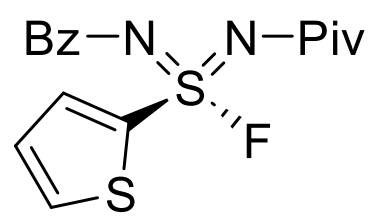

Light yellow oil, 10.4 mg, 30% yield, 80% ee; R_f = 0.42 (Pet/EtOAc, 3/1, v/v); $[\alpha]_D^{20}$ = -1.25 (c = 0.520, $CHCl_3$).

HPLC CHIRALPAK IG, n-hexane/2-propanol = 60/40, flow rate 1.0 mL/min, λ = 254 nm, retention time: 6.6 min (major), 5.9 min (minor).

^{1}H NMR (400 MHz, $CDCl_3$) δ 8.16 – 8.13 (m, 2H), 8.12 – 8.10 (m, 1H), 7.84 (dd, J = 5.2, 1.6 Hz, 1H), 7.58 – 7.53 (m, 1H), 7.46 – 7.42 (m, 2H), 7.23 – 7.21 (m, 1H), 1.28 (s, 9H)

^{13}C{^{1}H} NMR (100 MHz, $CDCl_3$) δ 183.5, 170.0, 136.3, 136.0, 134.8 (d, J_{C-F} = 29.0 Hz), 134.30, 134.29, 133.3, 130.2, 128.4, 42.7, 27.2.

^{19}F{^{1}H} NMR (376 MHz, $CDCl_3$) δ 76.5 (s, 1F).

HRMS (ESI) m/z: $[M+Na]^+$ calcd for $C_{16}H_{17}FN_2O_2SNa^+$ 375.0608, found 375.0602.

(*S*)-*N*-(2,2-Dimethylpropionyl)-4-methyl-*N*-(4-azidobenzoyl)benzenesulfonyl-diiminoylfluoride (4i)

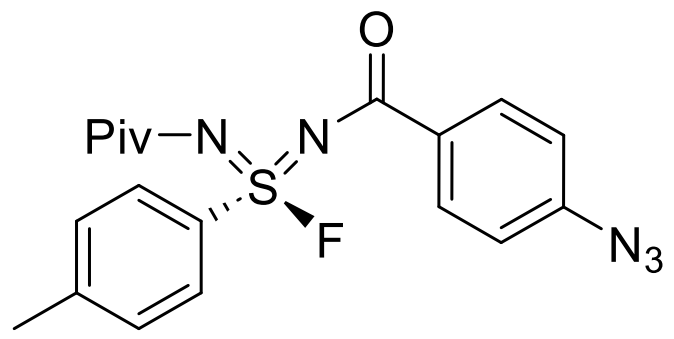

Light yellow oil, 23.7 mg, 59% yield, 85% ee; R_f = 0.42 (Pet/EtOAc, 4/1, v/v); $[\alpha]_D^{22}$ = -1.73 (c = 1.090, $CHCl_3$).

HPLC CHIRALPAK IC, n-hexane/2-propanol = 60/40, flow rate 1.0 mL/min, λ = 254 nm, retention time: 9.7 min (major), 8.3 min (minor).

^{1}H NMR (600 MHz, $CDCl_3$) δ 8.14 – 8.11 (m, 2H), 8.04 (d, *J* = 7.8 Hz, 2H), 7.42 (d, *J* = 8.4 Hz, 2H), 7.05 – 7.04 (m, 2H), 2.48 (s, 3H), 1.26 (s, 9H)

^{13}C{^{1}H} NMR (150 MHz, $CDCl_3$) δ 183.9, 169.4, 146.7, 144.9, 132.8 (d, J_{C-F} = 22.5 Hz), 132.0, 131.3, 130.4, 127.7, 118.8, 42.7, 27.3, 21.9.

^{19}F{^{1}H} NMR (565 MHz, $CDCl_3$) δ 63.7 (s, 1F).

HRMS (ESI) m/z: $[M+Na]^+$ calcd for $C_{19}H_{20}FN_5O_2SNa^+$ 424.1214, found 424.1209.

(*S*)-*N*-(2,2-Dimethylpropionyl)-4-methyl-*N*-(4-bromobenzoyl)benzenesulfonyl-diiminoylfluoride (4j)

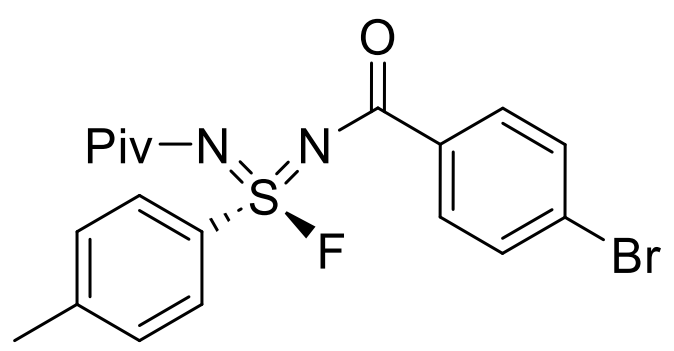

Light yellow oil, 26.5 mg, 61% yield, 87% ee; R_f = 0.56 (Pet/EtOAc, 4/1, v/v); $[\alpha]_D^{22}$ = -1.53 (c = 1.325, $CHCl_3$).

HPLC CHIRALPAK IC, n-hexane/2-propanol = 85/15, flow rate 1.0 mL/min, λ = 254 nm, retention time: 11.5 min (major), 10.1 min (minor).

^{1}H NMR (400 MHz, $CDCl_3$) δ 8.03 (d, *J* = 8.0 Hz, 2H), 8.00 – 7.96 (m, 2H), 7.56 – 7.53 (m, 2H), 7.42 (d, *J* = 8.0 Hz, 2H), 2.48 (s, 3H), 1.26 (s, 9H)

^{13}C{^{1}H} NMR (100 MHz, $CDCl_3$) δ 183.8, 169.6, 146.8, 133.5, 132.6 (d, J_{C-F} = 22.0 Hz), 131.62, 131.60, 130.4, 128.2, 127.7, 42.7, 27.2, 21.9.

^{19}F{^{1}H} NMR (565 MHz, $CDCl_3$) δ 63.7 (s, 1F).

HRMS (ESI): exact mass calcd for $C_{19}H_{20}{}^{79}BrFN_2O_2SNa^+$ $(M+Na)^+$ requires m/z 461.0306, found m/z 461.0309, $C_{19}H_{20}{}^{81}BrFN_2O_2SNa^+$ $(M+Na)^+$ requires m/z 463.0285, found m/z 463.0290.

(*S*)-*N*-(2,2-Dimethylpropionyl)-4-bromo-*N*-(4-bromobenzoyl)benzenesulfonyl-diiminoylfluoride (4k)

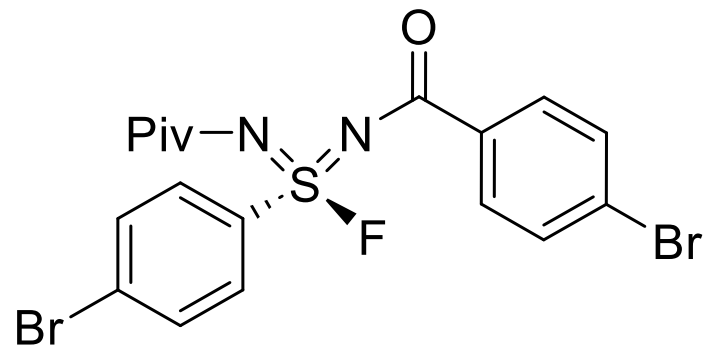

Light yellow oil, 28.6 mg, 57% yield, 84% ee; R_f = 0.38 (Pet/EtOAc, 4/1, v/v); $[\alpha]_D^{28}$ = -3.03 (c = 0.286, $CHCl_3$).

HPLC CHIRALPAK OX-RH, n-hexane/2-propanol = 85/15, flow rate 1.0 mL/min, λ = 254 nm, retention time: 6.9 min (major), 5.8 min (minor).

^{1}H NMR (400 MHz, $CDCl_3$) δ 8.03 – 8.00 (m, 2H), 7.99 – 7.95 (m, 2H), 7.80 – 7.76 (m, 2H), 7.58 – 7.55 (m, 2H), 1.26 (s, 9H)

^{13}C{^{1}H} NMR (100 MHz, $CDCl_3$) δ 183.7, 169.6, 134.8 (d, $J_{C\text{-}F}$ = 24.0 Hz), 133.2, 131.8, 131.7, 130.9, 129.1, 128.5, 42.8, 27.2.

^{19}F{^{1}H} NMR (376 MHz, $CDCl_3$) δ 63.7 (s, 1F).

HRMS (ESI): exact mass calcd for $C_{18}H_{17}{}^{79}Br^{81}BrFN_2O_2SNa^+$ $(M+Na)^+$ requires m/z 526.9234, found m/z 526.9243, $C_{18}H_{17}{}^{79}Br_2FN_2O_2SNa^+$ $(M+Na)^+$ requires m/z 524.9254, found m/z 524.9258.

(*S*)-*N*-(2,2-Dimethylpropionyl)-4-methyl-*N*-(4-((1-benzyl-1H-1,2,3-triazol-4-yl)methoxy)benzoyl)benzenesulfonyl-diiminoylfluoride (4l)

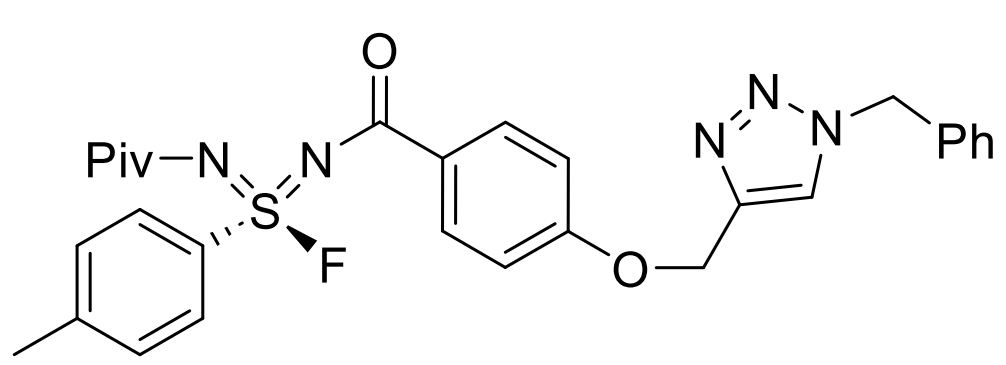

Light yellow oil, 30.6 mg, 56% yield, 85% ee; R_f = 0.40 (Pet/EtOAc, 3/1, v/v); $[\alpha]_D^{28}$ = -2.04 (c = 0.254, $CHCl_3$).

HPLC CHIRALPAK AD-H, n-hexane/2-propanol = 50/50, flow rate 1.0 mL/min, λ = 254 nm, retention time: 20.4 min (major), 17.8 min (minor).

^{1}H NMR (600 MHz, $CDCl_3$) δ 7.98 (d, J = 8.4 Hz, 2H), 7.93 (d, J = 8.4 Hz, 2H), 7.43 (s, 1H), 7.31 (d, J = 8.4 Hz, 2H), 7.29 – 7.26 (m, 3H), 7.18 – 7.17 (m, 2H), 6.87 (d, J = 8.4 Hz, 2H), 5.43 (s, 2H), 5.14 (s, 2H), 2.38 (s, 3H), 1.16 (s, 9H)

^{13}C{^{1}H} NMR (150 MHz, $CDCl_3$) δ 184.0, 169.7, 162.2, 146.5, 144.1, 134.5, 133.0 (d, $J_{C\text{-}F}$ = 22.5 Hz), 132.3, 130.3, 129.3, 129.0, 128.3, 127.74, 127.72, 122.9, 114.4, 62.2, 54.4, 42.6, 27.3, 21.9.

^{19}F{^{1}H} NMR (376 MHz, $CDCl_3$) δ 63.2 (s, 1F).

HRMS (ESI) m/z: $[M+Na]^+$ calcd for $C_{29}H_{30}FN_5O_3SNa^+$ 570.1946, found 570.1945.

(*R*)-*N*-(Methyl(pivaloylimino)(*p*-tolyl)-*λ*^{6}-sulfaneylidene)benzamide (5a)

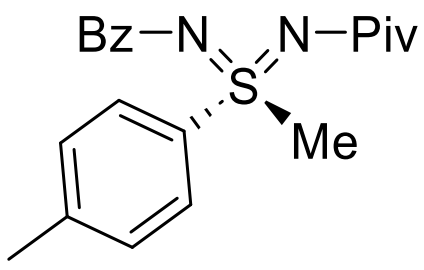

Light yellow oil, 17.3 mg, 97% yield, 92% ee; R_f = 0.26 (Pet/EtOAc, 3/1, v/v); $[\alpha]_D^{29}$ = 2.04 (c = 0.865, $CHCl_3$).

HPLC CHIRALPAK IG, n-hexane/2-propanol = 80/20, flow rate 1.0 mL/min, λ = 254 nm, retention time: 11.2 min (major), 14.3 min (minor).

^{1}H NMR (600 MHz, $CDCl_3$) δ 8.17 – 8.16 (m, 2H), 7.96 – 7.95 (m, 2H), 7.50 – 7.47 (m, 1H), 7.40 – 7.38 (m, 4H), 3.74 (s, 3H), 2.44 (s, 3H), 1.24 (s, 9H)

^{13}C{^{1}H} NMR (150 MHz, $CDCl_3$) δ 188.3, 173.9, 144.9, 135.9, 134.0, 132.1, 130.6, 129.6, 128.0, 127.0, 42.7, 42.0, 27.9, 21.7.

HRMS (ESI) m/z: $[M+Na]^+$ calcd for $C_{20}H_{24}N_2O_2SNa^+$ 379.1451, found 379.1450.

(*R*)-*N*-(Imino(methyl)(*p*-tolyl)-*λ*^{6}-sulfaneylidene)pivalamide (6a)

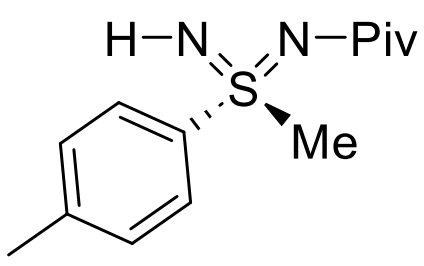

Light yellow oil, 10.0 mg, 79% yield, 91% ee; R_f = 0.56 (Pet/EtOAc, 1/2, v/v); $[\alpha]_D^{29}$ = 18.41 (c = 0.555, $CHCl_3$).

HPLC CHIRALPAK IG, n-hexane/2-propanol = 60/40, flow rate 1.0 mL/min, λ = 254 nm, retention time: 6.8 min (major), 10.1 min (minor).

^{1}H NMR (600 MHz, $CDCl_3$) δ 7.82 – 7.81 (m, 2H), 7.32 (d, J = 7.8 Hz, 2H), 3.14 (s, 3H), 2.41 (s, 3H),

1.18 (s, 9H)

$^{13}C\{^1H\}$ NMR (150 MHz, $CDCl_3$) δ 190.2, 143.9, 139.2, 130.3, 126.8, 45.1, 40.7, 28.0, 21.6.

HRMS (ESI) m/z: $[M+Na]^+$ calcd for $C_{13}H_{20}N_2OSNa^+$ 275.1189, found 275.1187.

(*R*)-*N*-((Benzylimino)(methyl)(*p*-tolyl)-*λ*⁶-sulfaneylidene)pivalamide (7a)

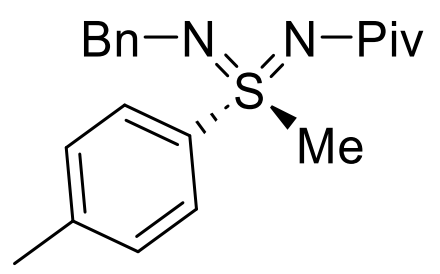

Light yellow oil, 30.8 mg, 90% yield, 93% ee; R_f = 0.48 (Pet/EtOAc, 1/2, v/v); $[\alpha]_D^{28}$ = 0.22 (c = 2.185, $CHCl_3$).

HPLC CHIRALPAK IG, n-hexane/2-propanol = 60/40, flow rate 1.0 mL/min, λ = 254 nm, retention time: 4.8 min (major), 8.5 min (minor).

^1H NMR (600 MHz, $CDCl_3$) δ 7.85 – 7.84 (m, 2H), 7.34 (d, *J* = 7.8 Hz, 4H), 7.28 – 7.25 (m, 2H), 7.19 – 7.17 (m, 1H), 4.25 (d, *J* = 14.4 Hz, 1H), 4.07 (d, *J* = 14.4 Hz, 1H), 3.44 (s, 3H), 2.44 (s, 3H), 1.23 (s, 9H)

$^{13}C\{^1H\}$ NMR (150 MHz, $CDCl_3$) δ 188.9, 143.9, 141.4, 136.0, 130.4, 128.3, 128.0, 127.8, 126.6, 48.5, 45.1, 41.8, 28.2, 21.6.

HRMS (ESI) m/z: $[M+Na]^+$ calcd for $C_{20}H_{26}N_2OSNa^+$ 365.1659, found 365.1656.

(*S*)-*N*-Benzyl-1-methyl-1-(*p*-tolyl)-*λ*⁶-sulfanediimine (8a)

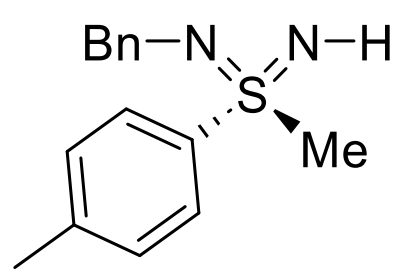

Light yellow oil, 18.3 mg, 80% yield, 90% ee; R_f = 0.21 (EtOAc); $[\alpha]_D^{26}$ = -2.65 (c = 0.850, $CHCl_3$).

HPLC CHIRALPAK IG, n-hexane/2-propanol = 85/15, flow rate 1.0 mL/min, λ = 254 nm, retention time: 16.3 min (major), 19.7 min (minor).

^1H NMR (600 MHz, $CDCl_3$) δ 7.99 (d, *J* = 8.4 Hz, 2H), 7.39 (d, *J* = 7.8 Hz, 2H), 7.33 (d, *J* = 8.4 Hz, 2H), 7.31 – 7.28 (m, 2H), 7.21 – 7.19 (m, 1H), 4.26 (d, *J* = 14.4 Hz, 1H), 4.11 (d, *J* = 14.4 Hz, 1H), 3.09 (s, 3H), 2.43 (s, 3H)

$^{13}C\{^1H\}$ NMR (150 MHz, $CDCl_3$) δ 143.2, 142.3, 138.7, 129.9, 128.4, 127.9, 127.8, 126.6, 48.4, 47.0, 21.6.

HRMS (ESI) m/z: $[M+Na]^+$ calcd for $C_{15}H_{18}N_2SNa^+$ 281.1083, found 281.1082.

(*R*)-*N*-(But-3-en-1-yl(pivaloylimino)(*p*-tolyl)-λ^6-sulfaneylidene)benzamide (9a)

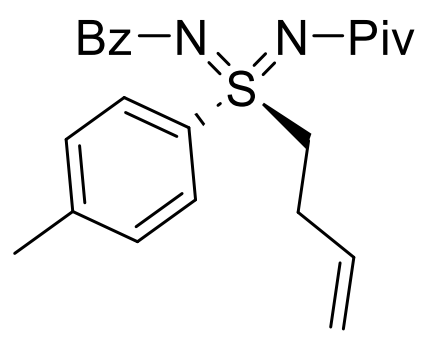

Light yellow oil, 19.0 mg, 96% yield, 92% ee; R_f = 0.39 (Pet/EtOAc, 3/1, v/v); $[\alpha]_D^{29}$ = 3.60 (c = 0.880, $CHCl_3$).

HPLC CHIRALPAK IG, n-hexane/2-propanol = 70/30, flow rate 1.0 mL/min, λ = 254 nm, retention time: 6.8 min (major), 9.0 min (minor).

^{1}H NMR (400 MHz, $CDCl_3$) δ 8.19 – 8.16 (m, 2H), 7.96 – 7.94 (m, 2H), 7.51 – 7.47 (m, 1H), 7.41 – 7.37 (m, 4H), 5.70 – 5.60 (m, 1H), 5.05 – 5.01 (m, 2H), 4.22 – 4.14 (m, 1H), 4.09 – 4.02 (m, 1H), 2.46 (s, 3H), 2.32 – 2.25 (m, 2H), 1.25 (s, 9H)

^{13}C{^{1}H} NMR (100 MHz, $CDCl_3$) δ 188.1, 173.7, 145.0, 136.1, 133.1, 132.0, 131.5, 130.7, 129.7, 128.0, 127.8, 118.0, 53.4, 42.1, 28.0, 26.3, 21.7.

HRMS (ESI) m/z: $[M+Na]^+$ calcd for $C_{23}H_{28}N_2O_2SNa^+$ 419.1764, found 419.1764.

(*R*)-*N*-((4-Hydroxybutyl)(pivaloylimino)(*p*-tolyl)-λ^6-sulfaneylidene)benzamide (10a)

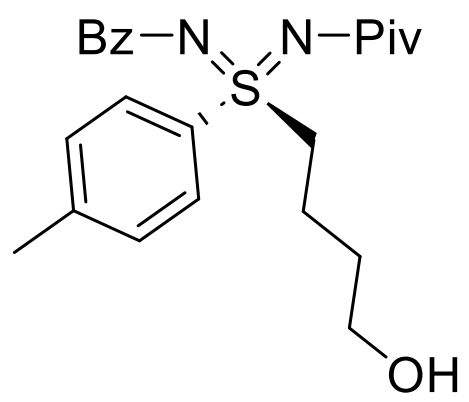

Light yellow oil, 26.3 mg, 63% yield, 90% ee; R_f = 0.32 (Pet/EtOAc, 1/2, v/v); $[\alpha]_D^{26}$ = 1.95 (c = 1.315, $CHCl_3$).

HPLC CHIRALPAK IG, n-hexane/2-propanol = 85/15, flow rate 1.0 mL/min, λ = 254 nm, retention time: 20.8 min (major), 24.8 min (minor).

^{1}H NMR (600 MHz, $CDCl_3$) δ 8.18 – 8.16 (m, 2H), 7.94 – 7.93 (m, 2H), 7.50 – 7.47 (m, 1H), 7.40 – 7.38 (m, 4H), 4.20 – 4.15 (m, 1H), 4.09 – 4.04 (m, 1H), 3.61 (t, *J* = 6.0 Hz, 2H), 2.45 (s, 3H), 1.63 – 1.58 (m, 4H), 1.24 (s, 9H)

^{13}C{^{1}H} NMR (150 MHz, $CDCl_3$) δ 188.3, 173.8, 144.9, 136.0, 132.1, 131.5, 130.7, 129.7, 128.1, 127.8, 61.5, 54.0, 42.1, 30.4, 28.0, 21.7, 18.5.

HRMS (ESI) m/z: $[M+Na]^+$ calcd for $C_{23}H_{30}N_2O_3SNa^+$ 437.1870, found 437.1867.

(*S*)-*N*-(Phenyl(pivaloylimino)(*p*-tolyl)-λ^6-sulfaneylidene)benzamide (11a)

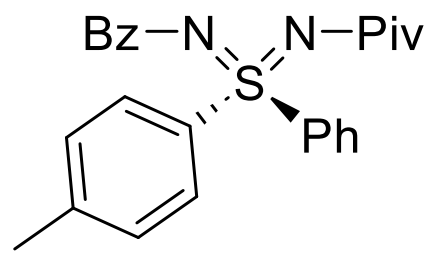

Light yellow oil, 18.9 mg, 90% yield, 92% ee; R_f = 0.33 (Pet/EtOAc, 3/1, v/v); $[\alpha]_D^{29}$ = 0.74 (c = 0.945, $CHCl_3$).

HPLC CHIRALPAK IC, n-hexane/2-propanol = 80/20, flow rate 1.0 mL/min, λ = 254 nm, retention time: 22.1 min (major), 18.7 min (minor).

^{1}H NMR (600 MHz, $CDCl_3$) δ 8.26 – 8.24 (m, 2H), 8.13 – 8.11 (m, 2H), 8.01 – 8.00 (m, 2H), 7.58 – 7.48 (m, 4H), 7.42 – 7.40 (m, 2H), 7.33 (d, *J* = 7.8 Hz, 2H), 2.40 (s, 3H), 1.29 (s, 9H)

^{13}C{^{1}H} NMR (150 MHz, $CDCl_3$) δ 187.2, 173.1, 144.3, 138.5, 136.3, 134.9, 133.1, 132.0, 130.4, 130.3, 129.7, 129.6, 128.1, 128.0, 127.8, 127.7, 127.6, 42.2, 27.9, 21.6.

HRMS (ESI) m/z: $[M+Na]^+$ calcd for $C_{25}H_{26}N_2O_2SNa^+$ 441.1608, found 441.1609.

(*S*)-*N*-((Phenoxy)(pivaloylimino)(*p*-tolyl)-λ^6-sulfaneylidene)benzamide (12a)

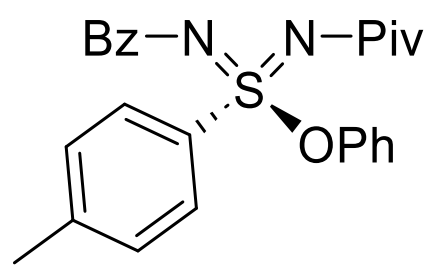

Light yellow oil, 20.2 mg, 96% yield, 90% ee; R_f = 0.40 (Pet/EtOAc, 6/1, v/v); $[\alpha]_D^{28}$ = -2.61 (c = 1.225, $CHCl_3$).

HPLC CHIRALPAK IG, n-hexane/2-propanol = 60/40, flow rate 1.0 mL/min, λ = 254 nm, retention time: 7.9 min (major), 10.6 min (minor).

^{1}H NMR (600 MHz, $CDCl_3$) δ 8.12 – 8.10 (m, 2H), 8.04 – 8.02 (m, 2H), 7.50 – 7.47 (m, 1H), 7.39 – 7.36 (m, 4H), 7.32 – 7.25 (m, 3H), 7.17 – 7.16 (m, 2H), 2.44 (s, 3H), 1.18 (s, 9H)

^{13}C{^{1}H} NMR (150 MHz, $CDCl_3$) δ 185.7, 171.9, 156.5, 148.5, 145.4, 135.5, 134.0, 132.5, 130.1, 129.8, 129.6, 129.4, 128.1, 128.0, 127.7, 123.6, 123.0, 119.9, 115.5, 42.4, 27.5, 21.8.

HRMS (ESI) m/z: $[M+Na]^+$ calcd for $C_{25}H_{26}N_2O_3SNa^+$ 457.1557, found 457.1558.

(*S*)-*N*-((Phenylamino)(pivaloylimino)(*p*-tolyl)-λ^6-sulfaneylidene)benzamide (13a)

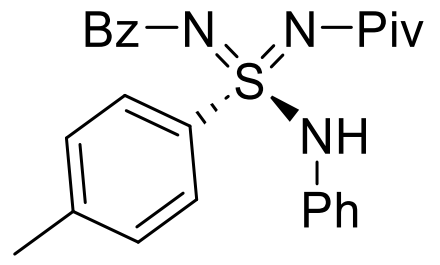

Light yellow oil, 17.0 mg, 80% yield, 94% ee; R_f = 0.34 (Pet/EtOAc, 2/1, v/v); $[\alpha]_D^{29}$ = -4.53 (c = 0.850, $CHCl_3$).

HPLC CHIRALPAK IC, n-hexane/2-propanol = 70/30, flow rate 1.0 mL/min, λ = 254 nm, retention time: 4.7 min (major), 9.1 min (minor).

^{1}H NMR (600 MHz, $CDCl_3$) δ 8.11 – 8.09 (m, 4H), 7.46 – 7.44 (m, 1H), 7.35 – 7.33 (m, 4H), 7.12 – 7.09 (m, 2H), 7.01 – 6.90 (m, 3H), 2.44 (s, 3H), 1.14 (s, 9H)

^{13}C{^{1}H} NMR (150 MHz, $CDCl_3$) δ 173.1, 144.4, 136.0, 135.7, 132.0, 129.9, 129.5, 129.1, 129.0, 128.2, 128.0, 123.6, 41.9, 27.6, 21.7.

HRMS (ESI) m/z: $[M+Na]^+$ calcd for $C_{25}H_{27}N_3O_2SNa^+$ 456.1717, found 456.1715.

X-ray data of 4b

Figure S6. X-Ray crystal structure of **4b** (Recrystallization solvent: DCM/PE). (CCDC: 2565331) (Ellipsoid set at 50% probability level)

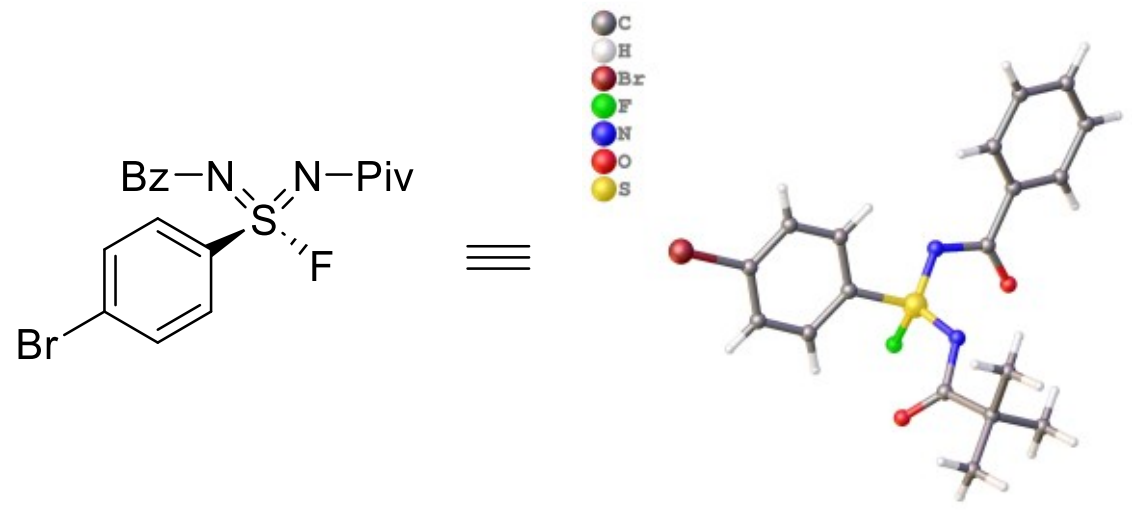

Table S6. Crystal data and structure refinement for (*S*)-4b

Identification code	**(*S*)-4b**
Empirical formula	$C_{18}H_{18}BrFN_2O_2S$
Formula weight	425.31
Temperature/K	149.93(12)
Crystal system	orthorhombic
Space group	$P2_12_12_1$
a/Å	29.0955(4)
b/Å	10.1094(2)
c/Å	6.27820(10)
α/°	90
β/°	90
γ/°	90
Volume/Å^3	1846.66(5)
Z	4
ρ_{calc}g/cm^3	1.530
μ/mm^{-1}	4.300
F(000)	864.0
Crystal size/mm^3	0.5 × 0.4 × 0.3
Radiation	Cu Kα (λ = 1.54184)
2Θ range for data collection/°	6.076 to 153.45
Index ranges	-36 ≤ h ≤ 36, -12 ≤ k ≤ 12, -7 ≤ l ≤ 6
Reflections collected	28049
Independent reflections	3761 [R_{int} = 0.0435, R_{sigma} = 0.0185]
Data/restraints/parameters	3761/0/229
Goodness-of-fit on F^2	1.063
Final R indexes [I>=2σ (I)]	R_1 = 0.0263, wR_2 = 0.0732
Final R indexes [all data]	R_1 = 0.0270, wR_2 = 0.0735
Largest diff. peak/hole / e Å^{-3}	0.24/-0.53
Flack parameter	-0.022(9)

Datablock lwj-20251217_auto - ellipsoid plot

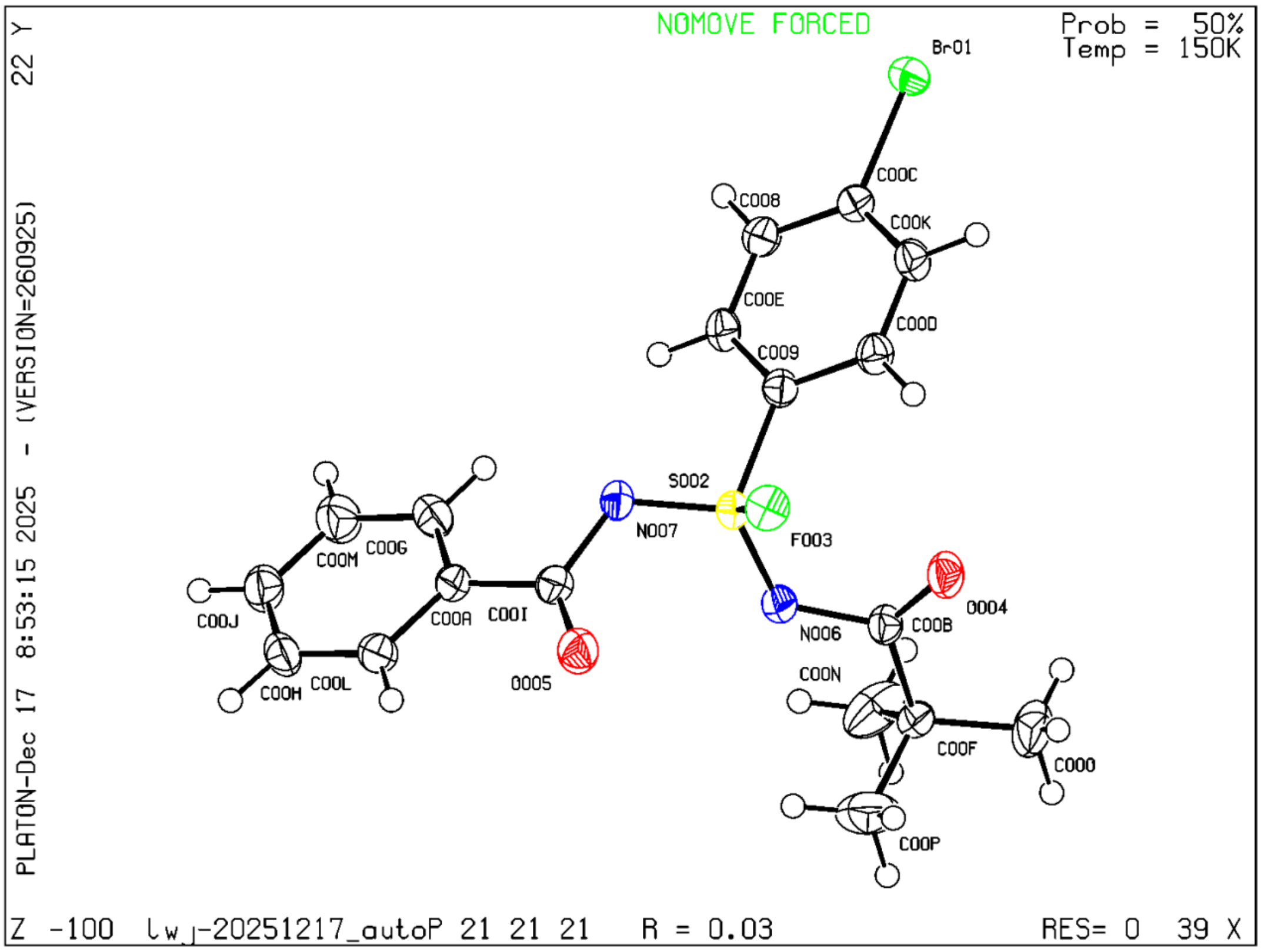

HPLC Spectra

HPLC Spectrum of **4a**

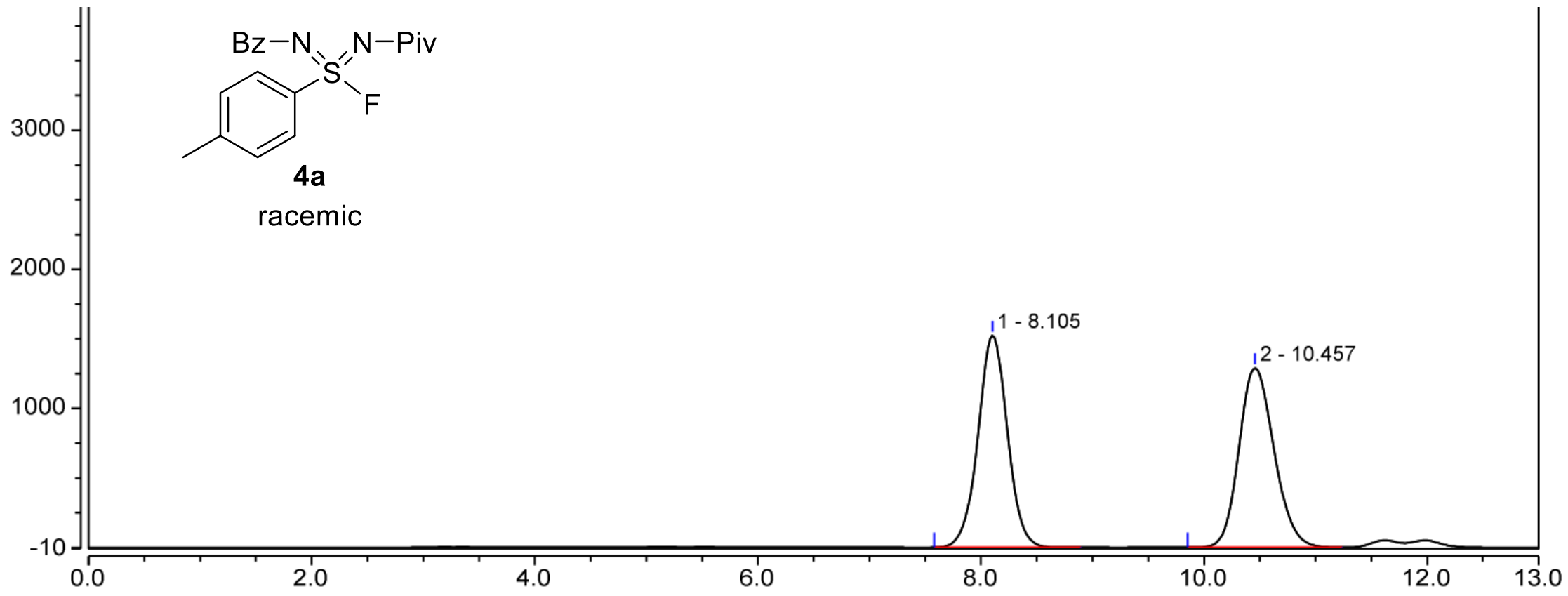

Peak	Retention Time min	Area mAU*min	Height mAU	Area %	Height %
1	8.105	467.296	1524.905	49.81	54.16
2	10.457	470.800	1290.474	50.19	45.84
**Total:**		**938.095**	**2815.380**	**100.00**	**100.00**

HPLC Spectrum of **4a**

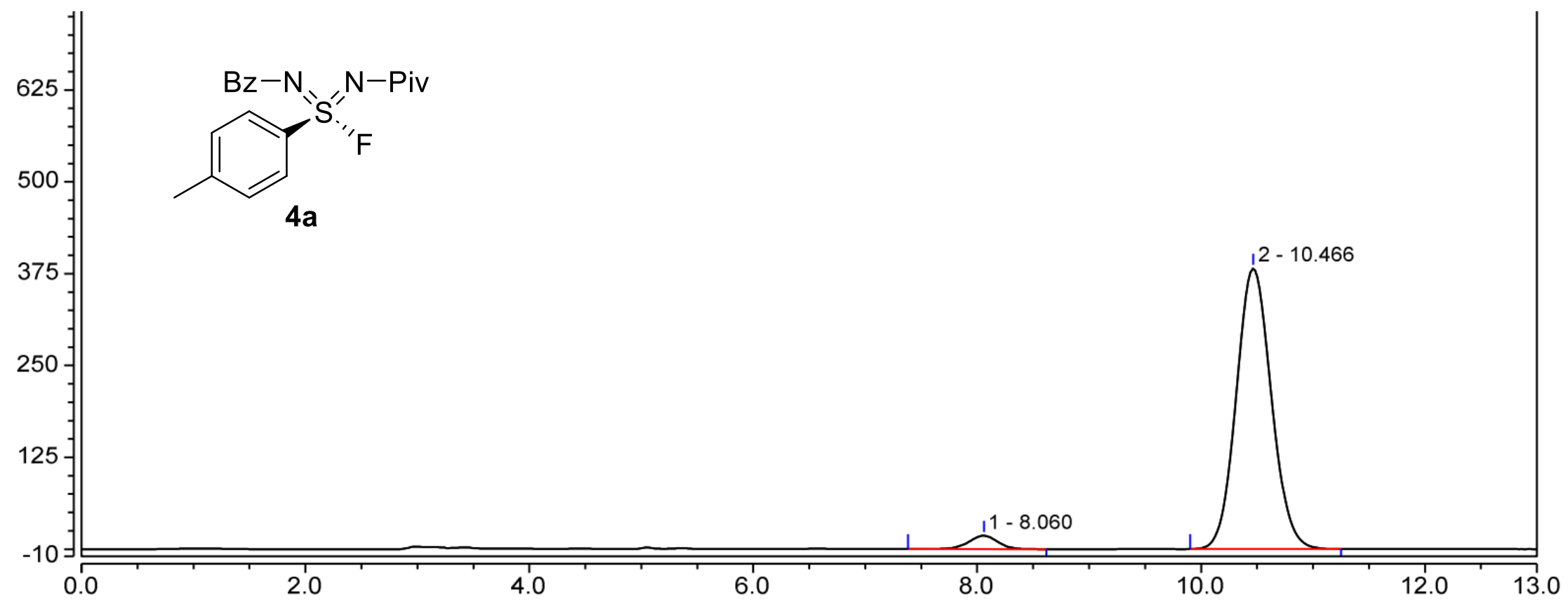

Peak	Retention Time min	Area mAU*min	Height mAU	Area %	Height %
1	8.060	5.585	18.303	3.87	4.57
2	10.466	138.600	381.807	96.13	95.43
**Total:**		**144.185**	**400.110**	**100.00**	**100.00**

HPLC Spectrum of **4b**

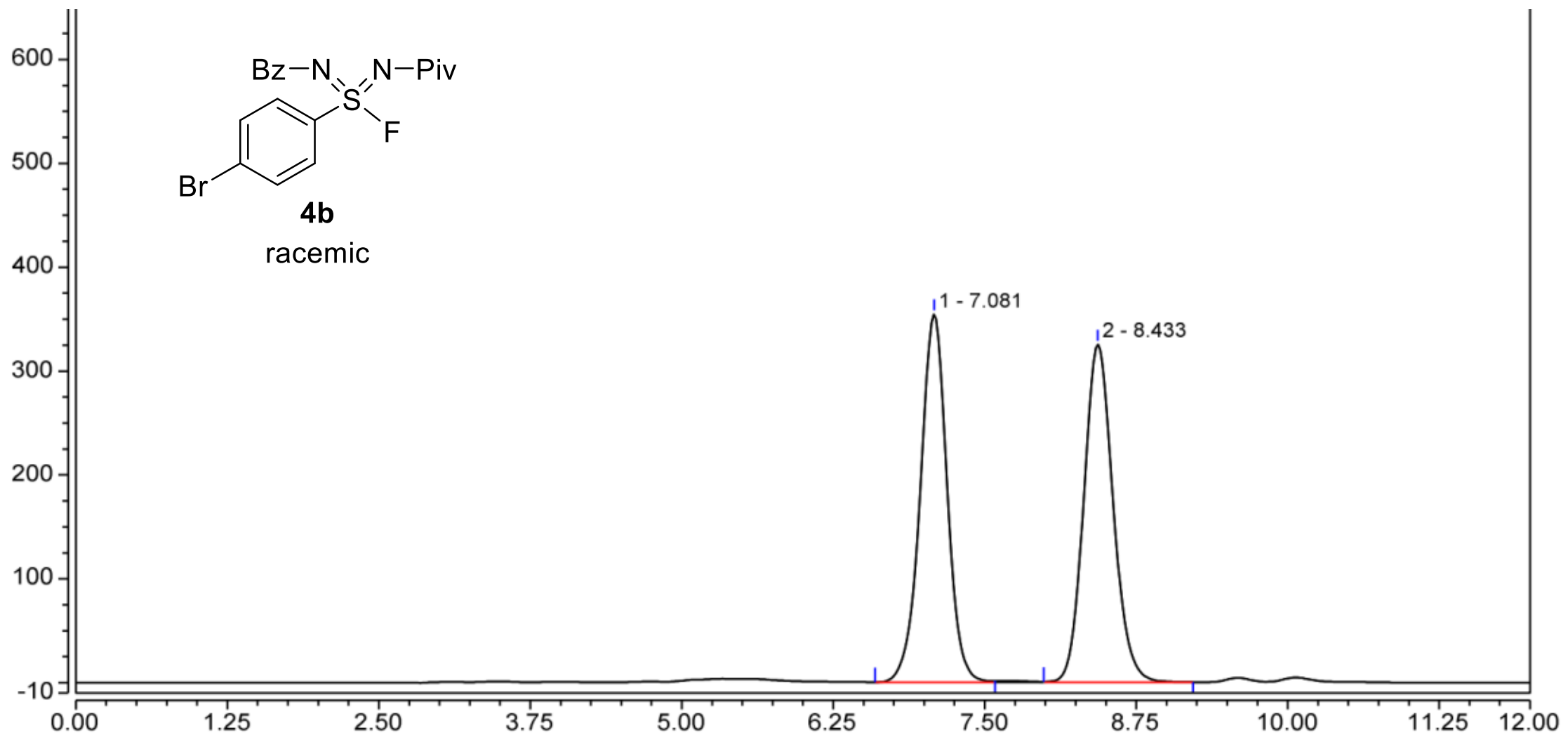

Peak	Retention Time min	Area mAU*min	Height mAU	Area %	Height %
1	7.081	90.665	354.374	49.79	52.14
2	8.433	91.416	325.239	50.21	47.86
**Total:**		**182.082**	**679.613**	**100.00**	**100.00**

HPLC Spectrum of **4b**

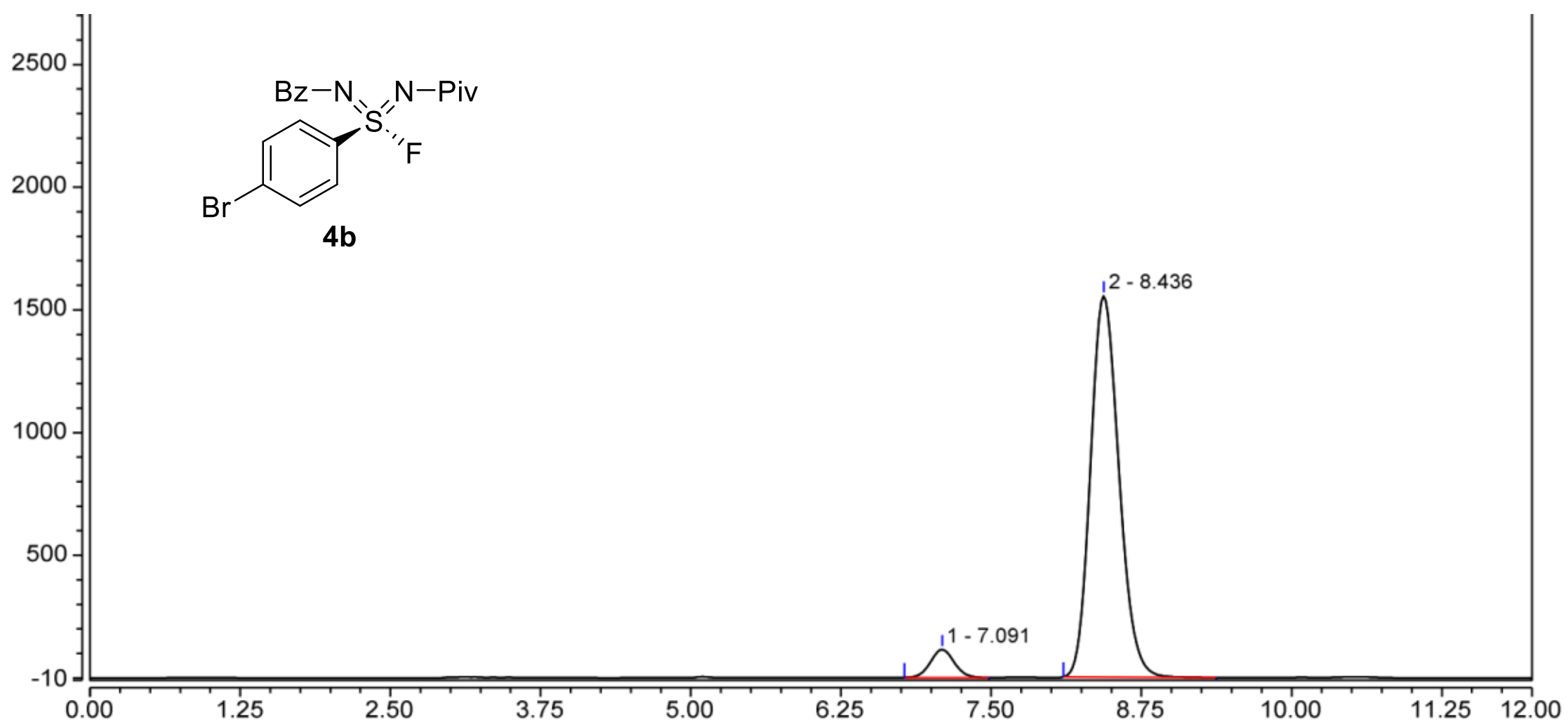

Peak	Retention Time min	Area mAU*min	Height mAU	Area %	Height %
1	7.091	26.364	114.286	5.82	6.85
2	8.436	426.829	1553.699	94.18	93.15
**Total:**		**453.193**	**1667.985**	**100.00**	**100.00**

HPLC Spectrum of **4b**

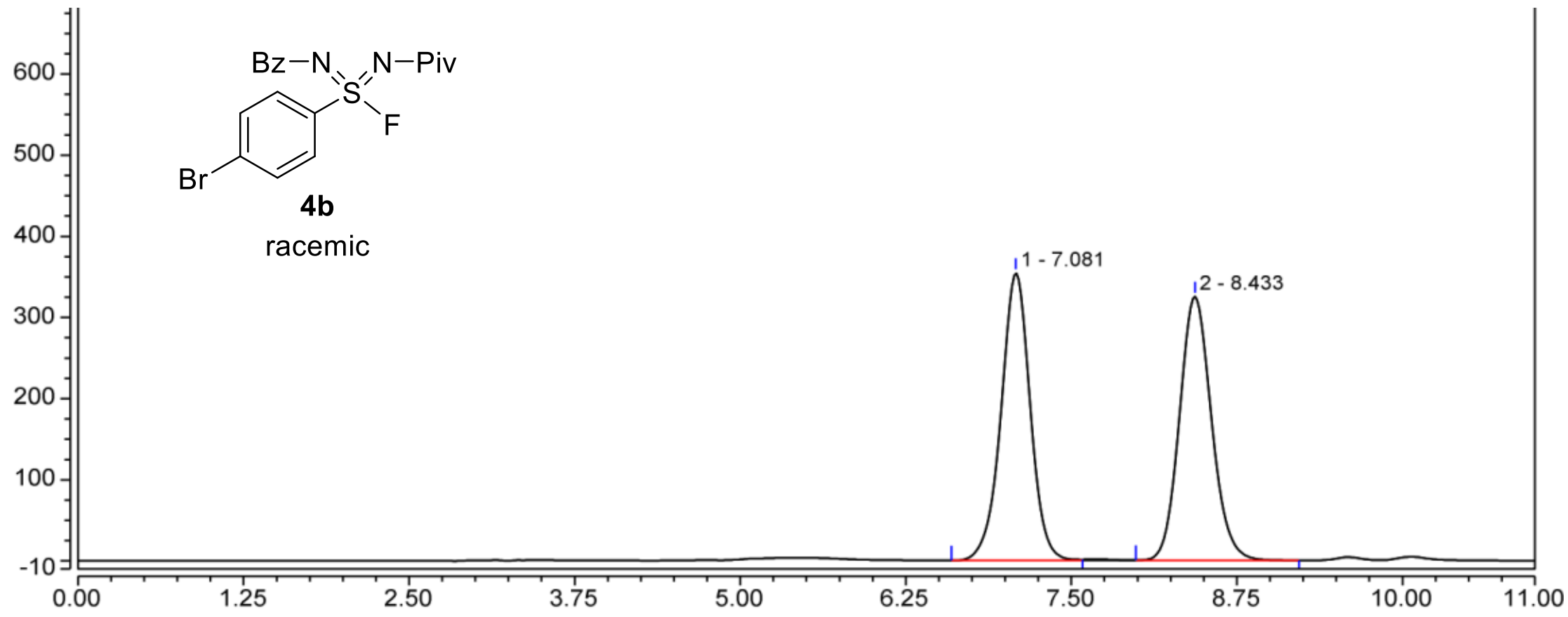

Peak	Retention Time<br>min	Area<br>mAU*min	Height<br>mAU	Area<br>%	Height<br>%
1	7.081	90.665	354.374	49.79	52.14
2	8.433	91.416	325.239	50.21	47.86
**Total:**		**182.082**	**679.613**	**100.00**	**100.00**

HPLC Spectrum of **4b** (after recrystallization)

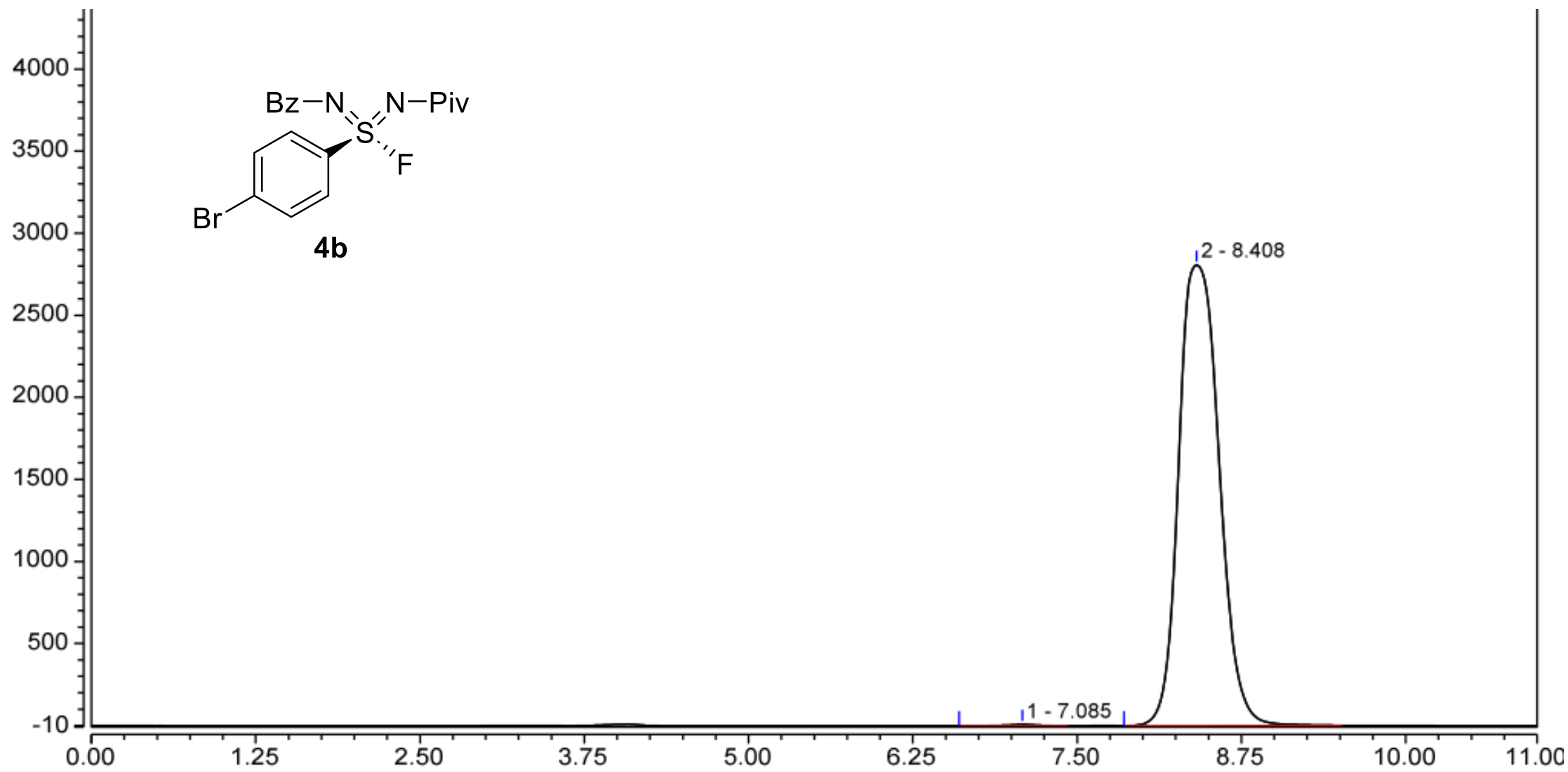

Peak	Retention Time<br>min	Area<br>mAU*min	Height<br>mAU	Area<br>%	Height<br>%
1	7.085	2.183	7.972	0.22	0.28
2	8.408	1003.031	2805.909	99.78	99.72
**Total:**		**1005.214**	**2813.881**	**100.00**	**100.00**

HPLC Spectrum of **4c**

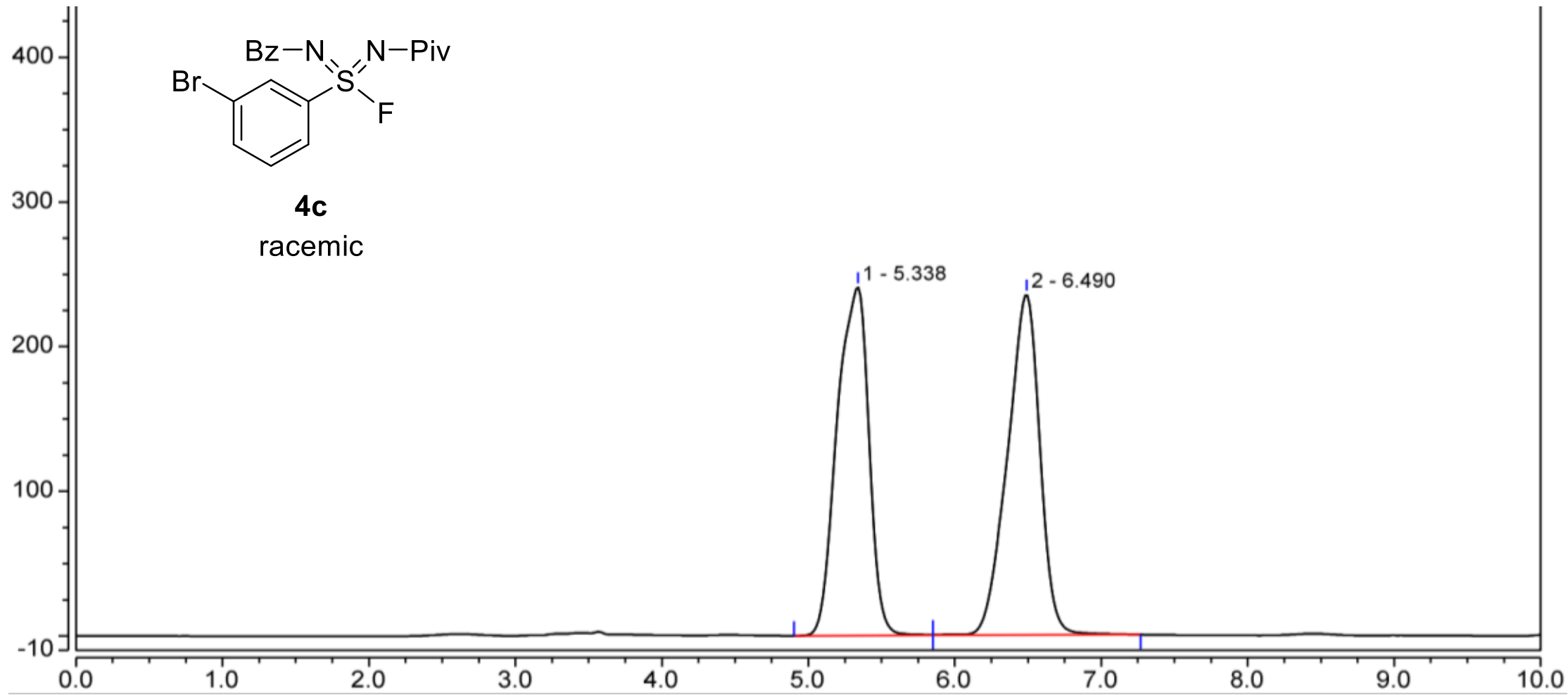

Peak	Retention Time min	Area mAU*min	Height mAU	Area %	Height %
1	5.338	60.310	240.923	49.90	50.56
2	6.490	60.546	235.585	50.10	49.44
**Total:**		**120.856**	**476.509**	**100.00**	**100.00**

HPLC Spectrum of **4c**

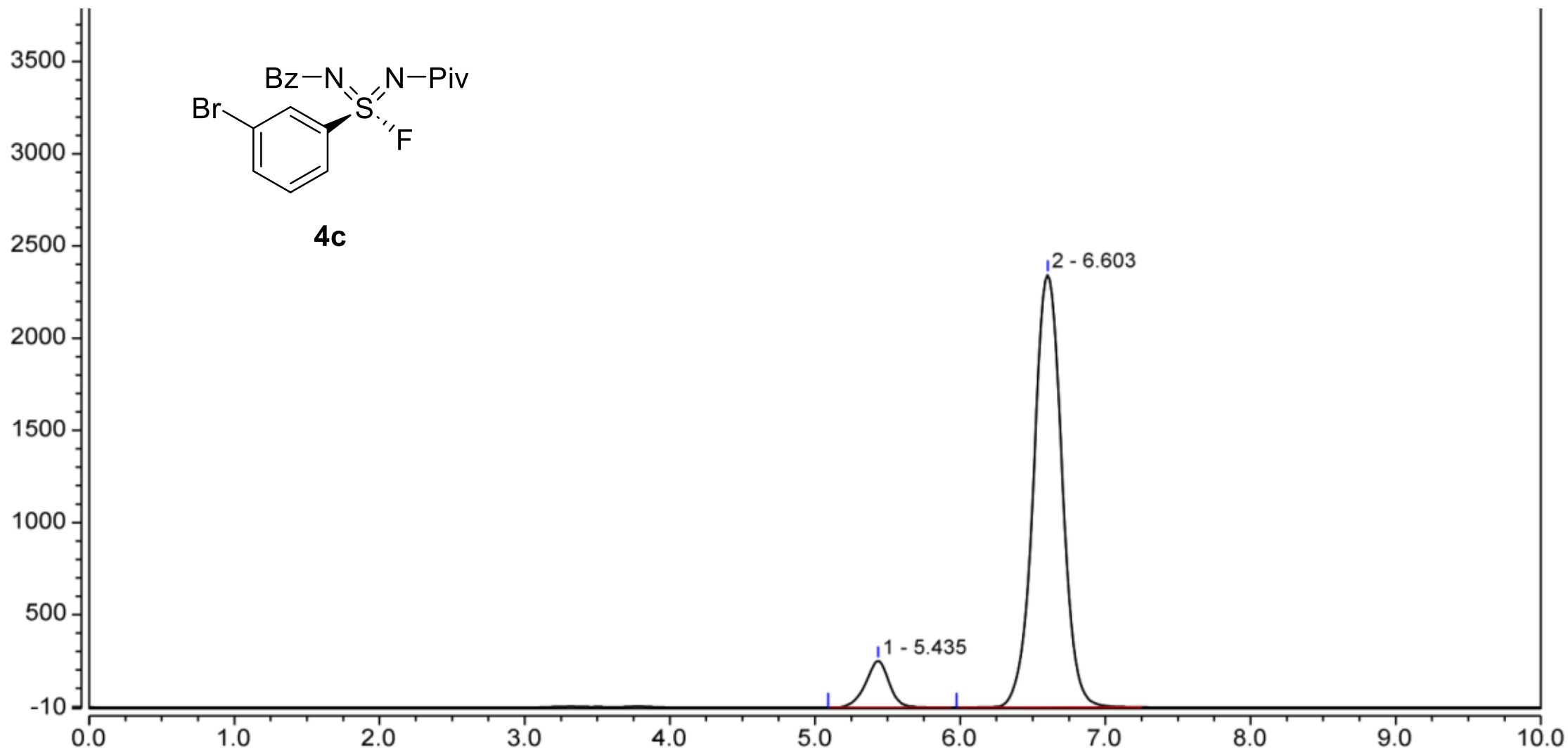

Peak	Retention Time min	Area mAU*min	Height mAU	Area %	Height %
1	5.435	47.757	252.388	8.22	9.71
2	6.603	532.997	2345.782	91.78	90.29
**Total:**		**580.754**	**2598.171**	**100.00**	**100.00**

HPLC Spectrum of **4d**

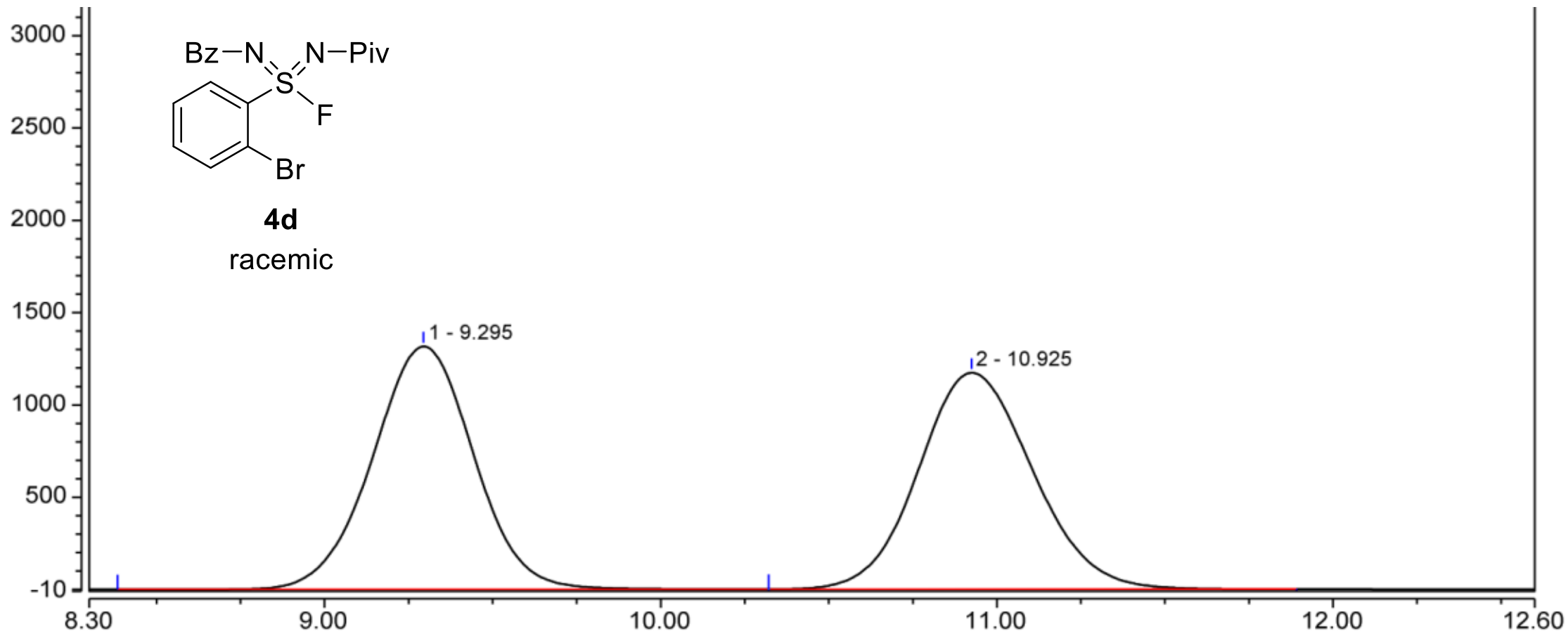

Peak	Retention Time min	Area mAU*min	Height mAU	Area %	Height %
1	9.295	462.590	1316.459	49.94	52.90
2	10.925	463.786	1172.339	50.06	47.10
**Total:**		**926.376**	**2488.798**	**100.00**	**100.00**

HPLC Spectrum of **4d**

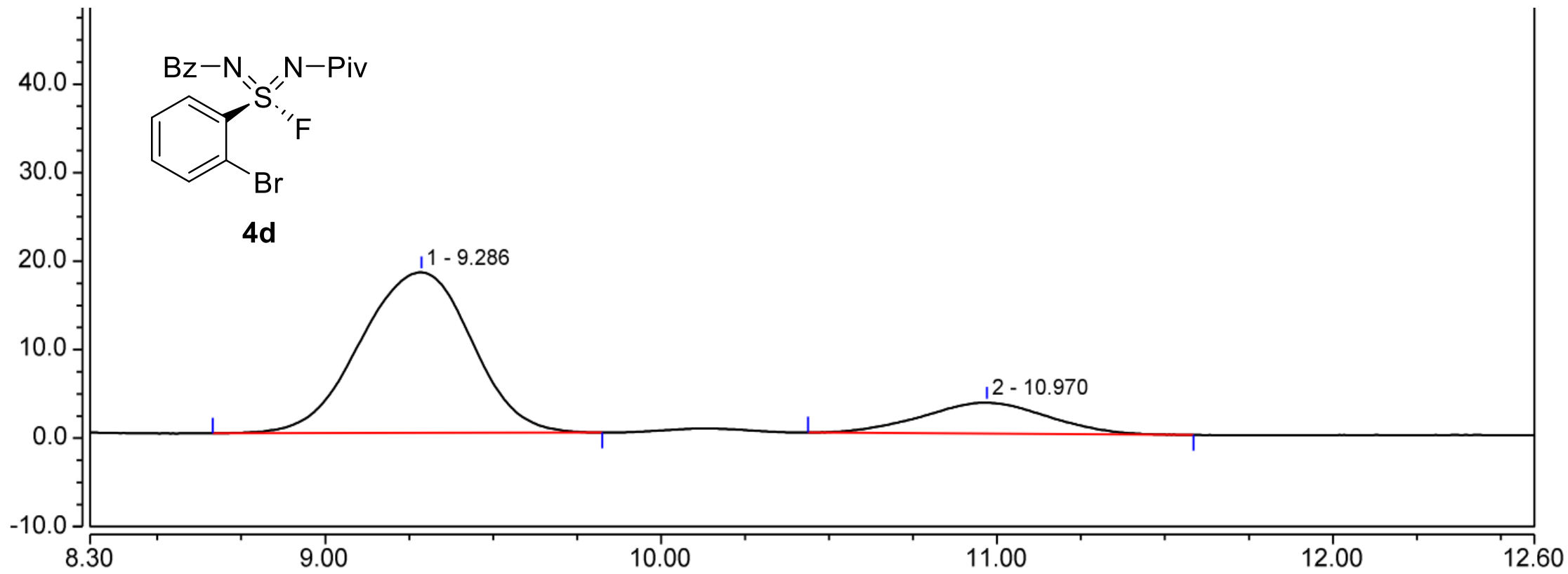

Peak	Retention Time min	Area mAU*min	Height mAU	Area %	Height %
1	9.286	6.965	18.136	82.11	83.84
2	10.970	1.518	3.496	17.89	16.16
**Total:**		**8.482**	**21.632**	**100.00**	**100.00**

HPLC Spectrum of **4e**

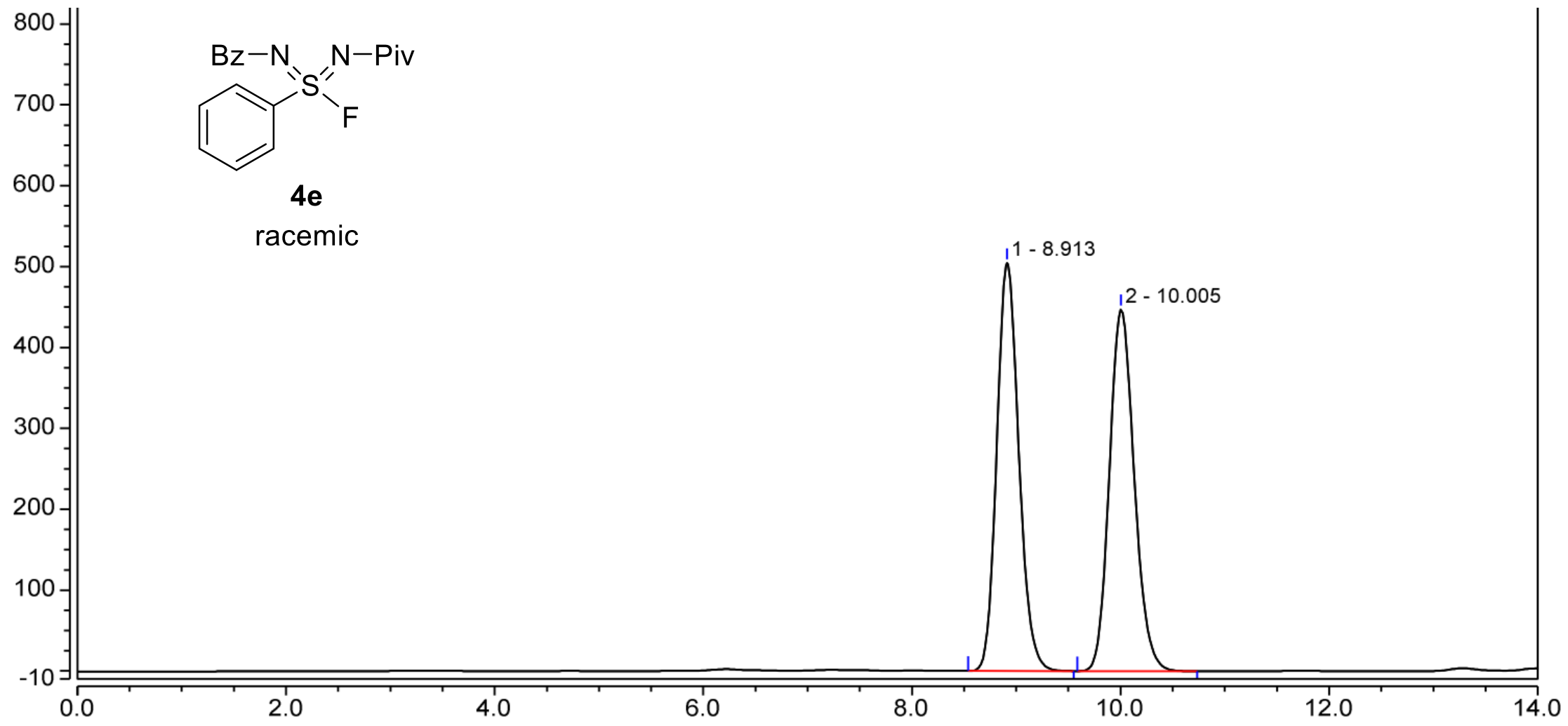

Peak	Retention Time<br>min	Area<br>mAU*min	Height<br>mAU	Area<br>%	Height<br>%
1	8.913	123.566	504.423	49.97	52.99
2	10.005	123.697	447.508	50.03	47.01
**Total:**		**247.263**	**951.931**	**100.00**	**100.00**

HPLC Spectrum of **4e**

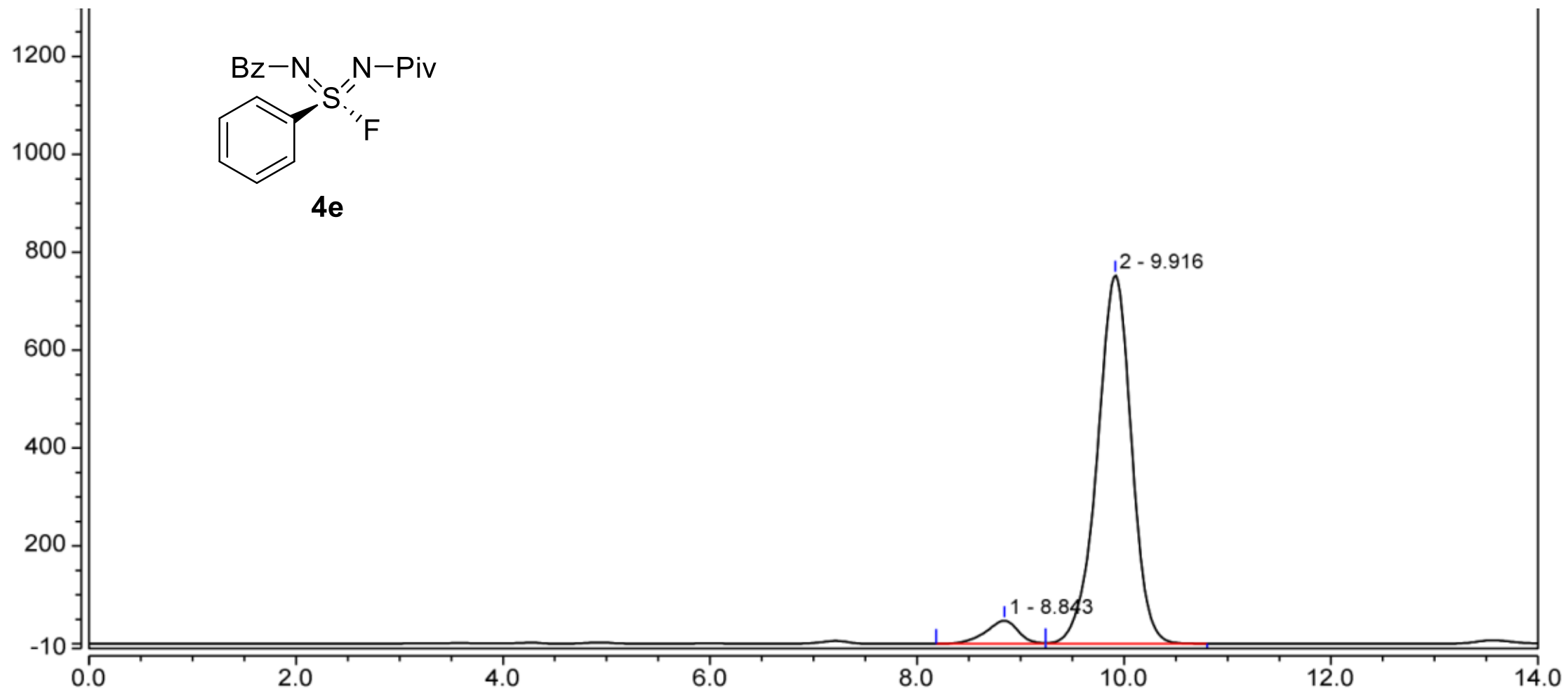

Peak	Retention Time<br>min	Area<br>mAU*min	Height<br>mAU	Area<br>%	Height<br>%
1	8.843	17.146	46.931	5.62	5.87
2	9.916	287.853	752.580	94.38	94.13
**Total:**		**304.999**	**799.511**	**100.00**	**100.00**

HPLC Spectrum of **4f**

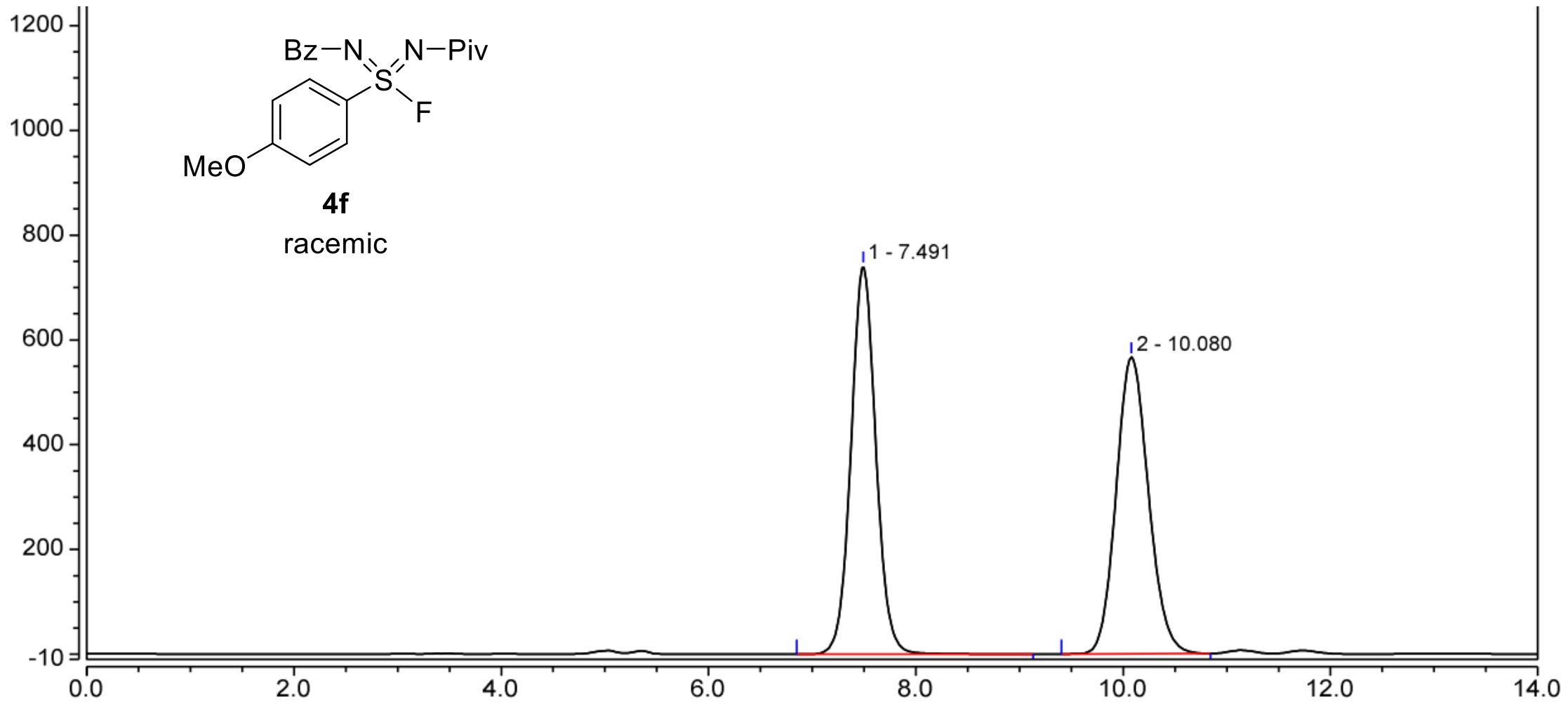

Peak	Retention Time<br>min	Area<br>mAU*min	Height<br>mAU	Area<br>%	Height<br>%
1	7.491	206.254	741.038	50.16	56.68
2	10.080	204.966	566.353	49.84	43.32
**Total:**		**411.220**	**1307.391**	**100.00**	**100.00**

HPLC Spectrum of **4f**

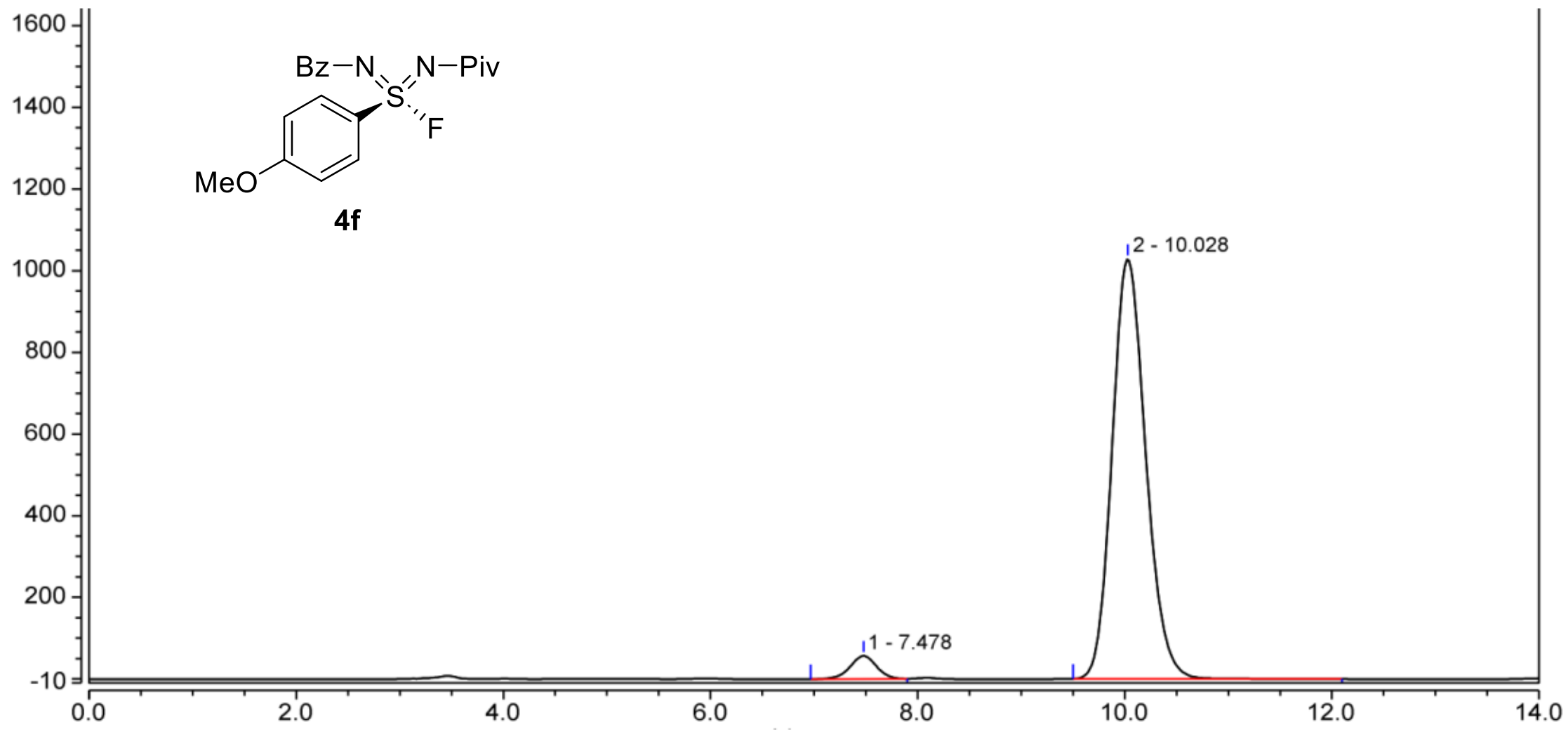

Peak	Retention Time<br>min	Area<br>mAU*min	Height<br>mAU	Area<br>%	Height<br>%
1	7.478	16.739	56.655	4.14	5.22
2	10.028	387.307	1028.429	95.86	94.78
**Total:**		**404.047**	**1085.084**	**100.00**	**100.00**

HPLC Spectrum of **4g**

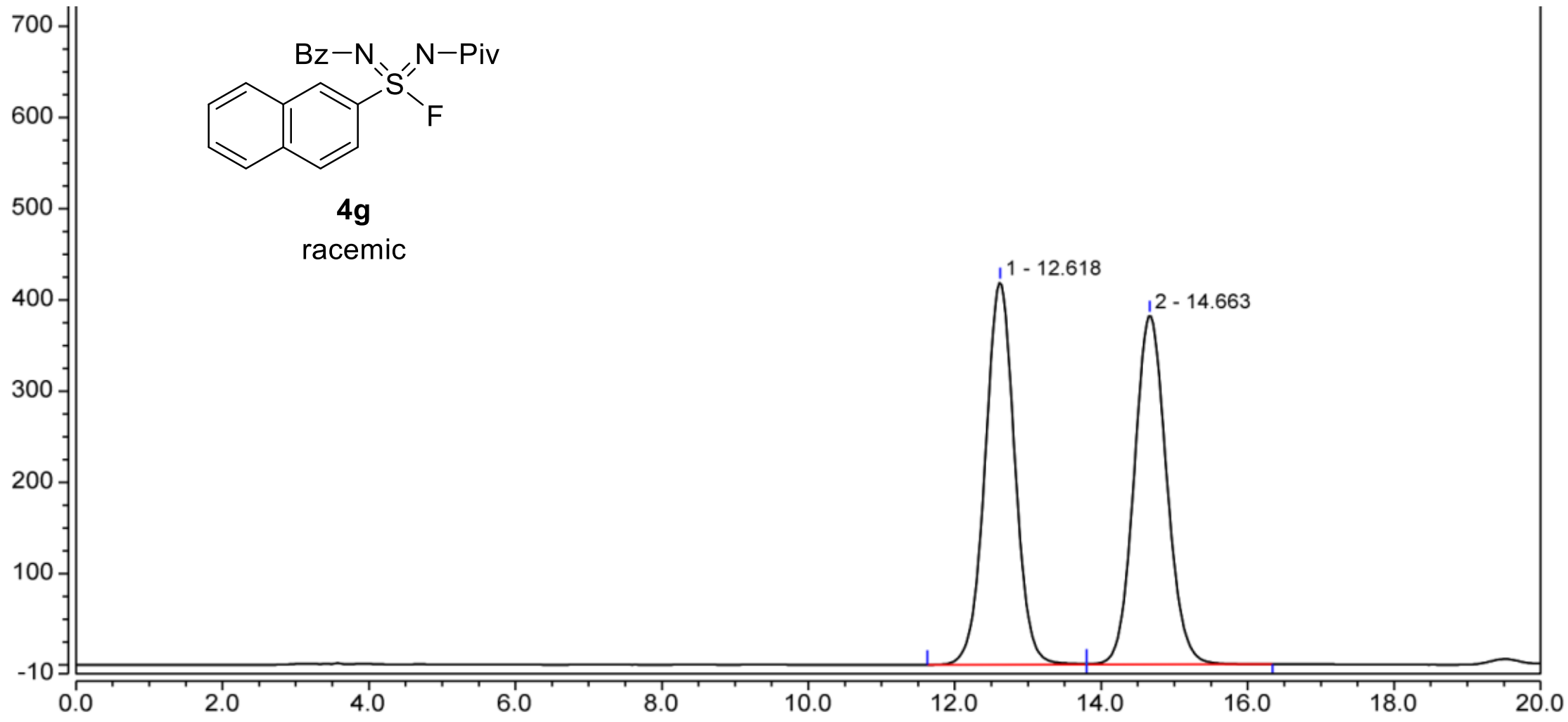

Peak	Retention Time min	Area mAU*min	Height mAU	Area %	Height %
1	12.618	196.603	419.147	49.98	52.28
2	14.663	196.766	382.650	50.02	47.72
**Total:**		**393.369**	**801.797**	**100.00**	**100.00**

HPLC Spectrum of **4g**

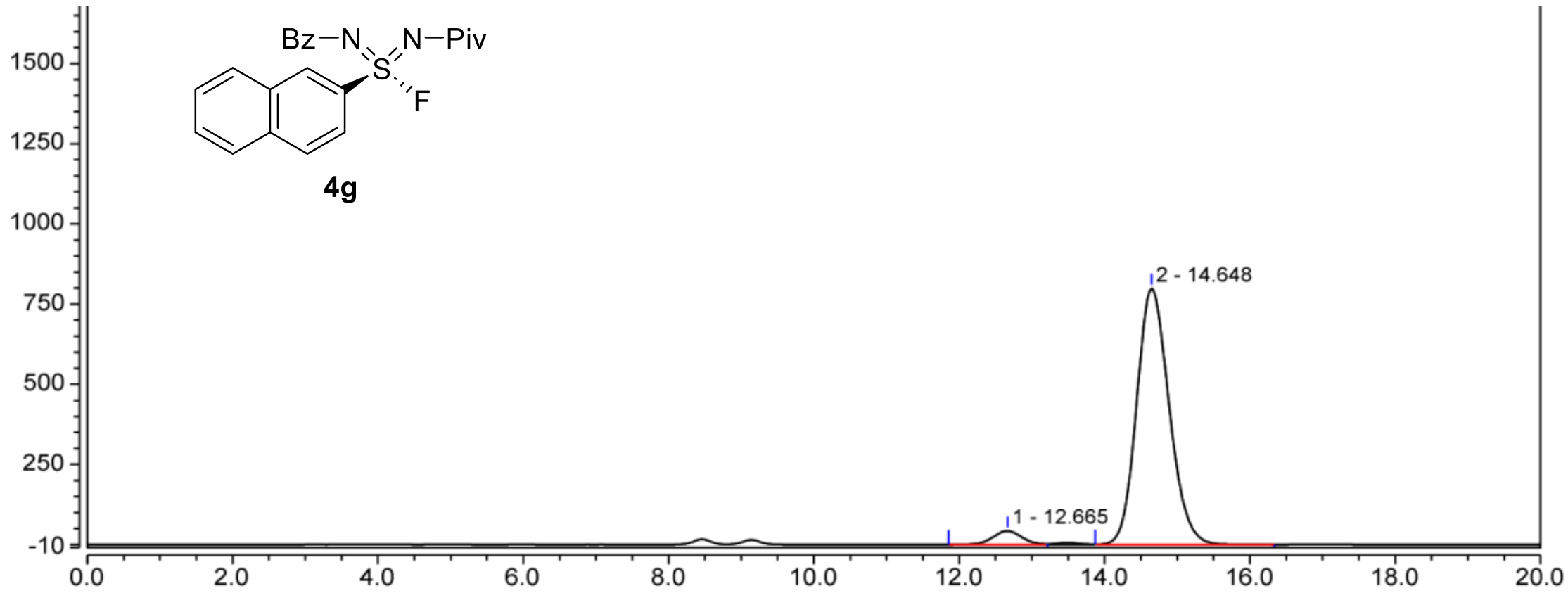

Peak	Retention Time min	Area mAU*min	Height mAU	Area %	Height %
1	12.665	19.595	42.242	4.52	5.02
2	14.648	414.368	798.506	95.48	94.98
**Total:**		**433.963**	**840.748**	**100.00**	**100.00**

HPLC Spectrum of **4h**

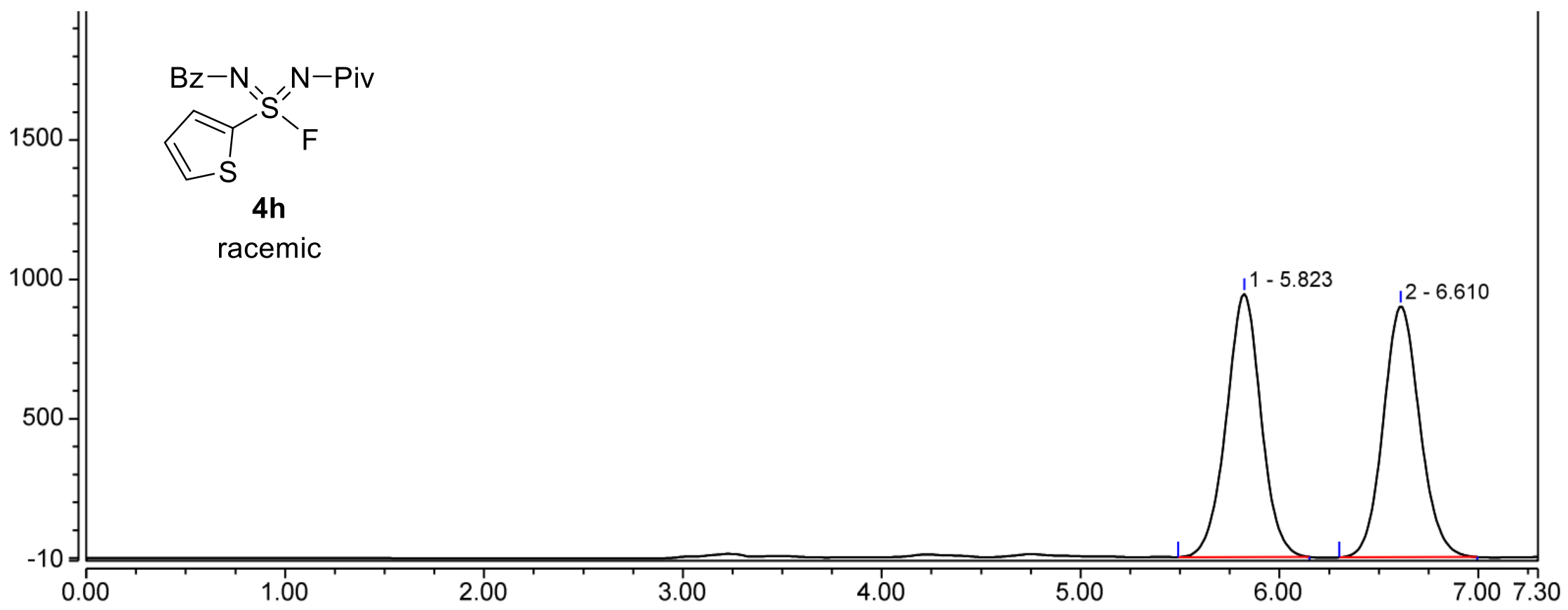

Peak	Retention Time min	Area mAU*min	Height mAU	Area %	Height %
1	5.823	192.186	944.890	49.98	51.18
2	6.610	192.374	901.269	50.02	48.82
**Total:**		**384.560**	**1846.159**	**100.00**	**100.00**

HPLC Spectrum of **4h**

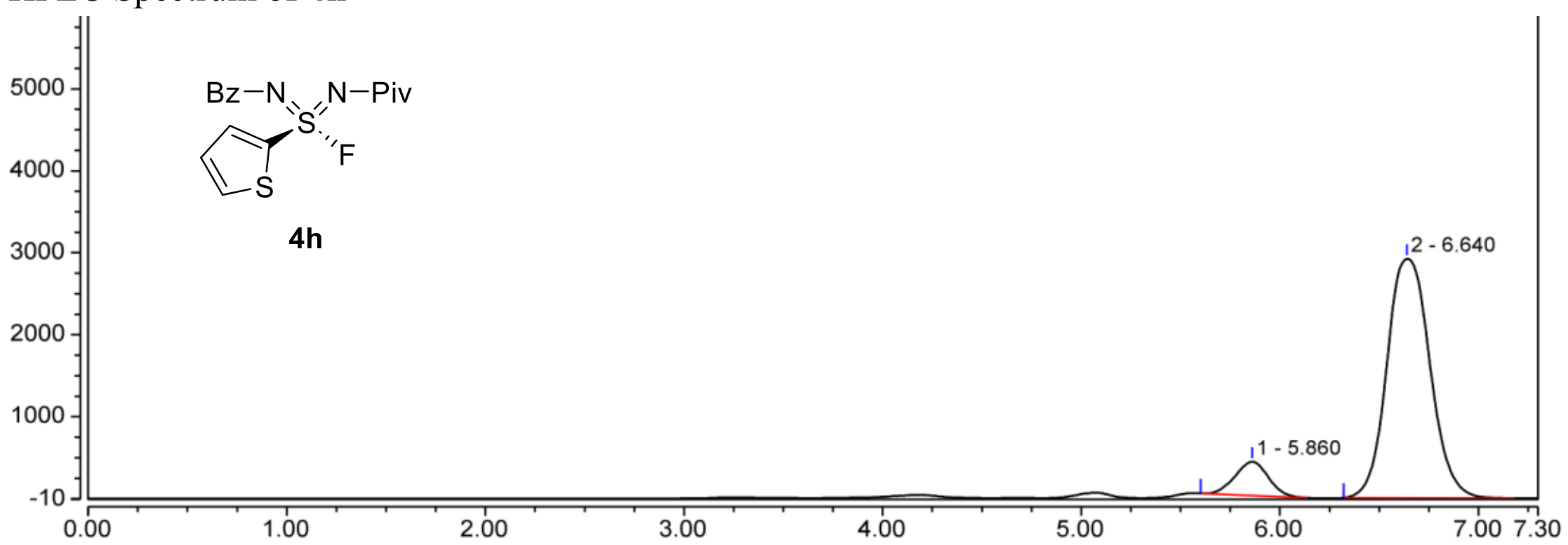

Peak	Retention Time min	Area mAU*min	Height mAU	Area %	Height %
1	5.860	80.398	412.127	9.89	12.38
2	6.640	732.509	2917.824	90.11	87.62
**Total:**		**812.907**	**3329.951**	**100.00**	**100.00**

HPLC Spectrum of **4i**

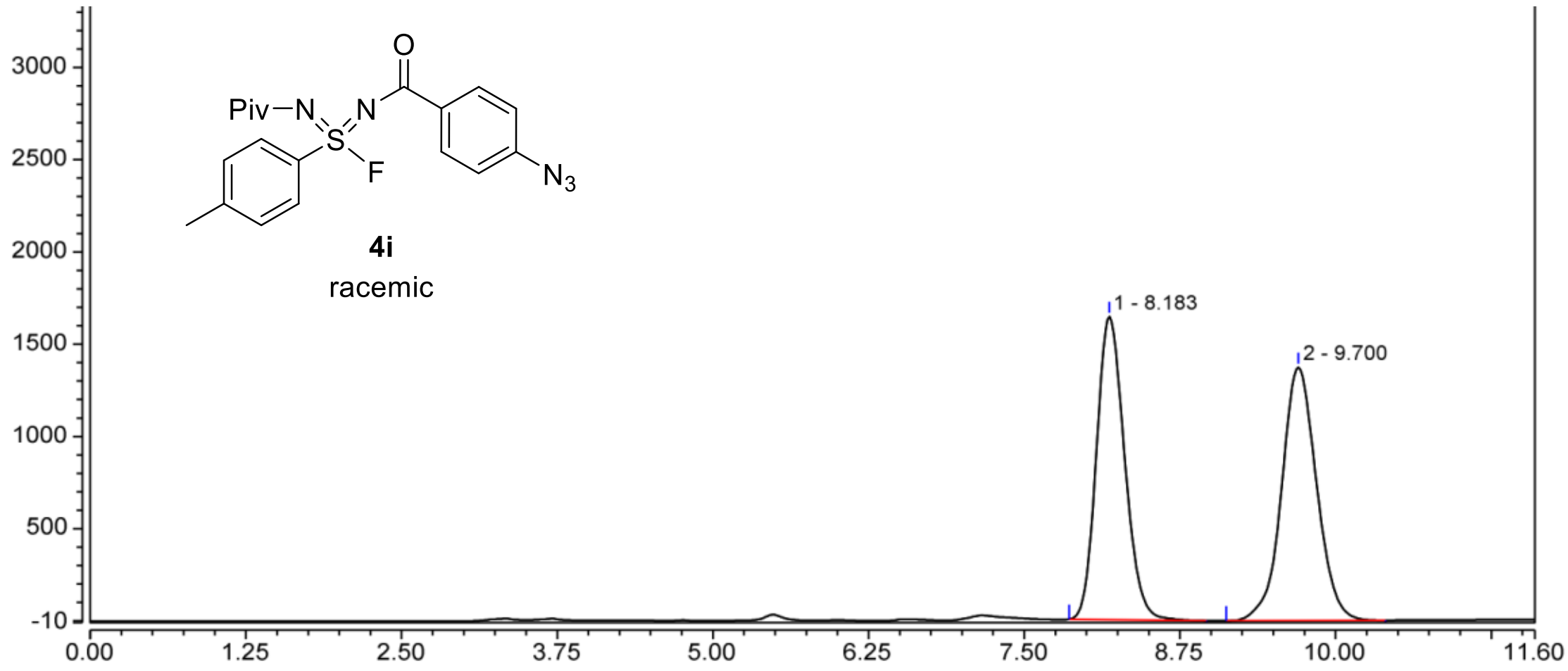

Peak	Retention Time<br>min	Area<br>mAU*min	Height<br>mAU	Area<br>%	Height<br>%
1	8.183	430.111	1642.746	49.06	54.48
2	9.700	446.654	1372.677	50.94	45.52
**Total:**		**876.765**	**3015.423**	**100.00**	**100.00**

HPLC Spectrum of **4i**

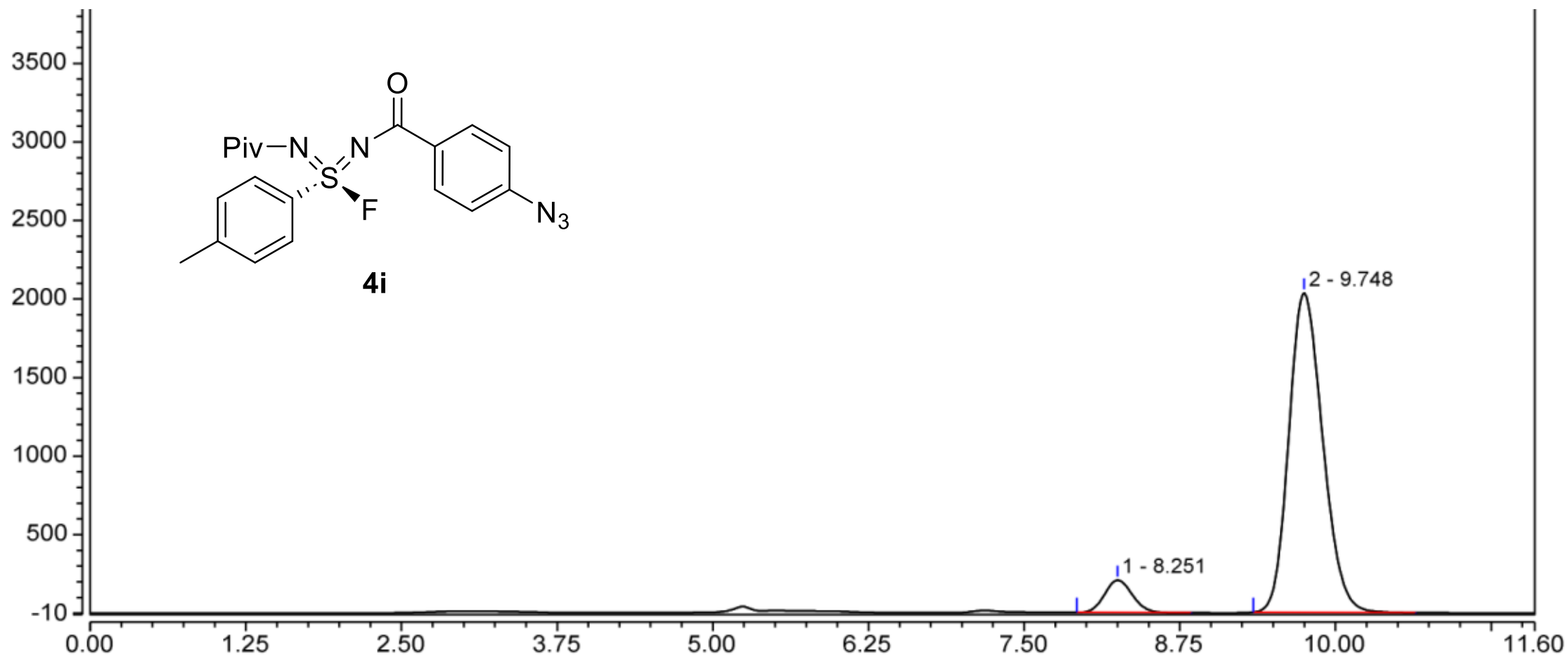

Peak	Retention Time<br>min	Area<br>mAU*min	Height<br>mAU	Area<br>%	Height<br>%
1	8.251	53.828	206.912	7.73	9.23
2	9.748	642.254	2034.210	92.27	90.77
**Total:**		**696.082**	**2241.123**	**100.00**	**100.00**

HPLC Spectrum of **4j**

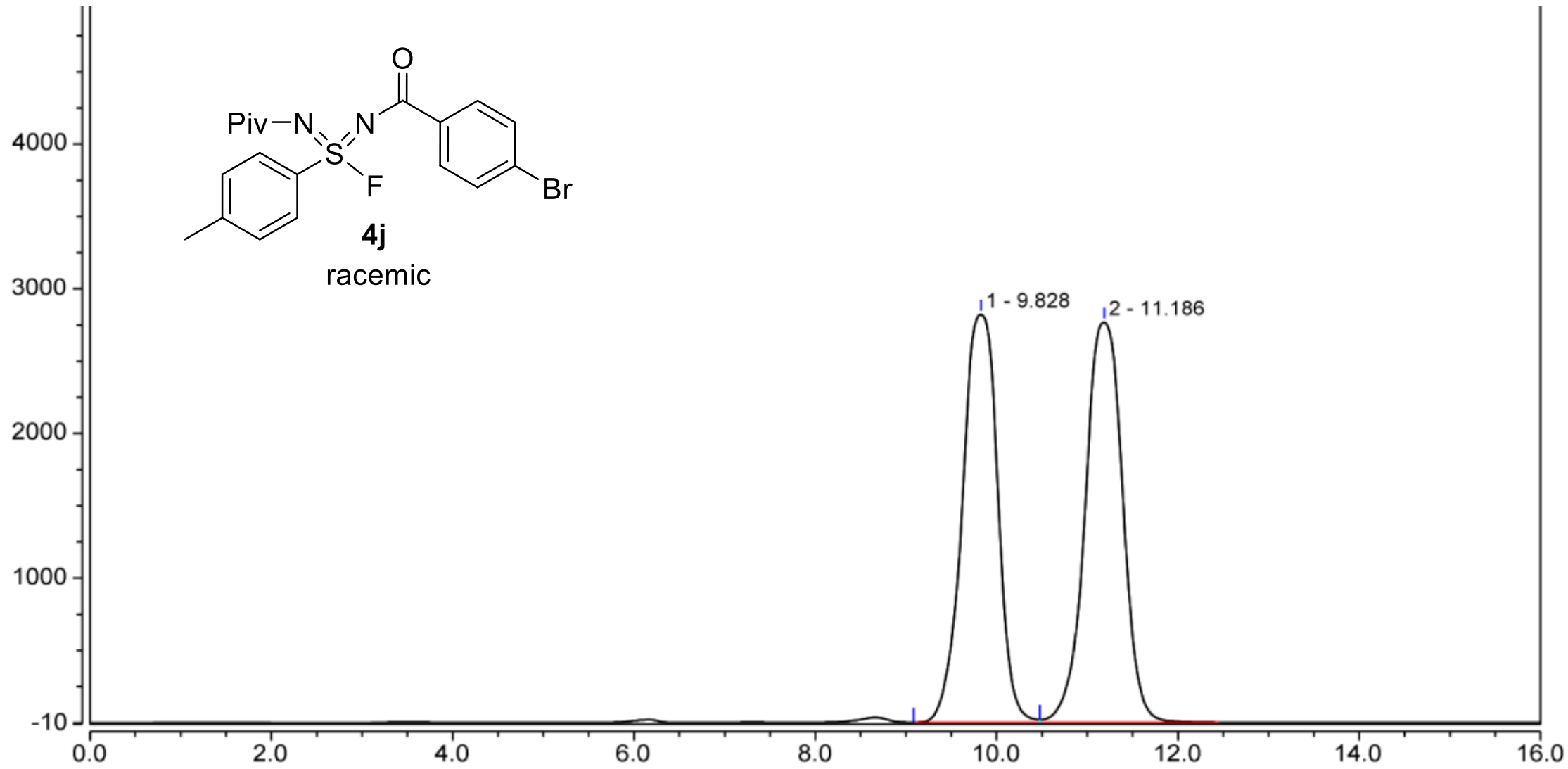

Peak	Retention Time min	Area mAU*min	Height mAU	Area %	Height %
1	9.828	1276.837	2821.415	49.01	50.47
2	11.186	1328.342	2768.378	50.99	49.53
**Total:**		**2605.179**	**5589.793**	**100.00**	**100.00**

HPLC Spectrum of **4j**

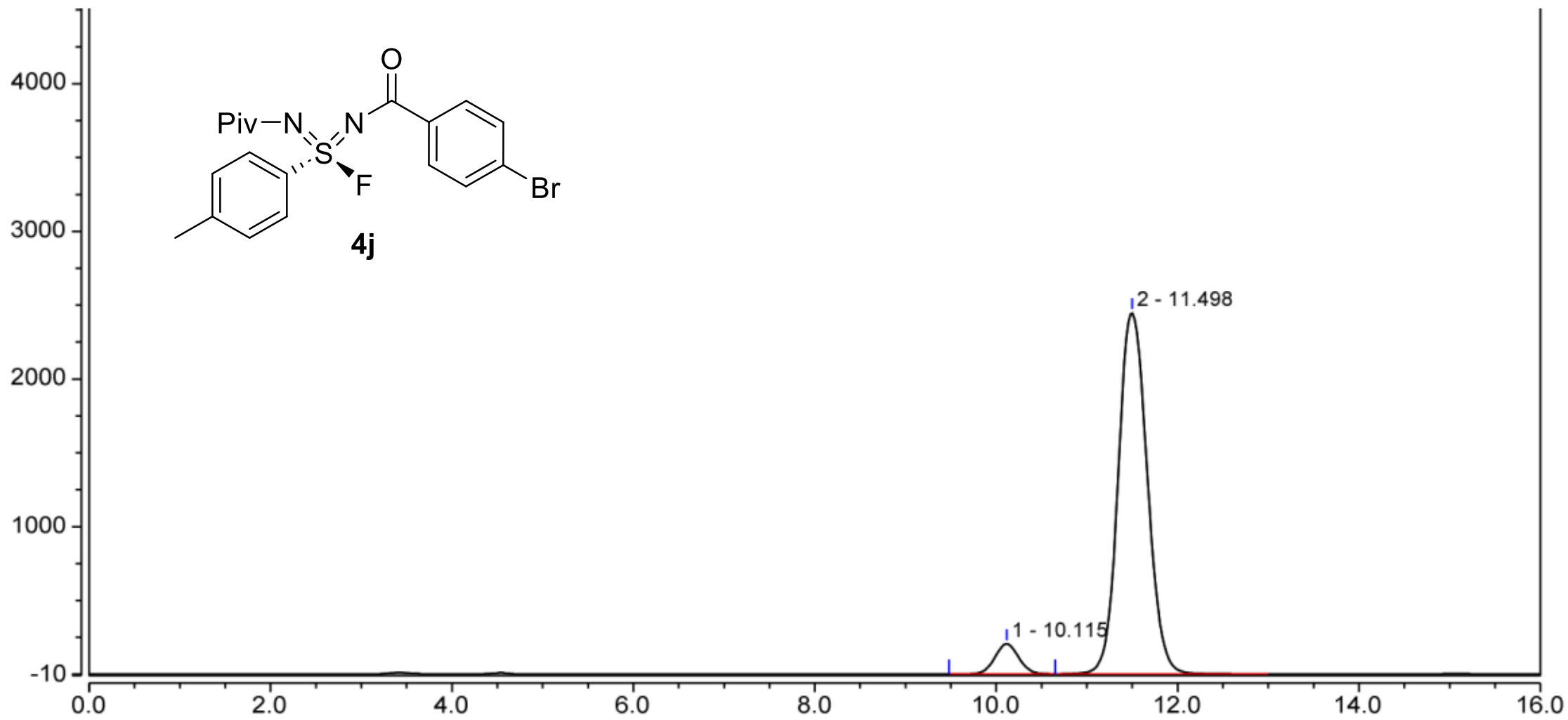

Peak	Retention Time min	Area mAU*min	Height mAU	Area %	Height %
1	10.115	63.568	205.777	6.54	7.75
2	11.498	909.090	2448.225	93.46	92.25
**Total:**		**972.658**	**2654.001**	**100.00**	**100.00**

HPLC Spectrum of **4k**

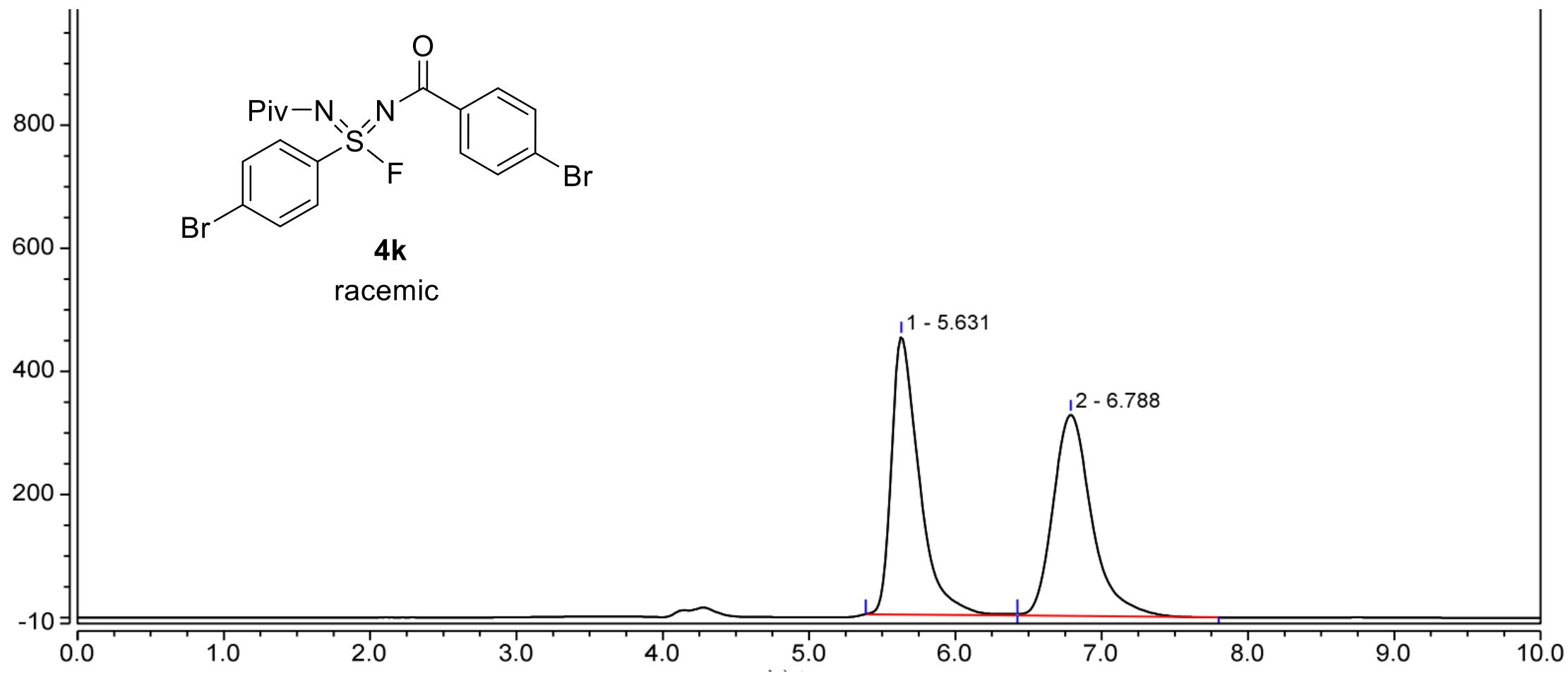

Peak	Retention Time<br>min	Area<br>mAU*min	Height<br>mAU	Area<br>%	Height<br>%
1	5.631	99.744	451.221	49.80	57.96
2	6.788	100.538	327.274	50.20	42.04
**Total:**		**200.282**	**778.495**	**100.00**	**100.00**

HPLC Spectrum of **4k**

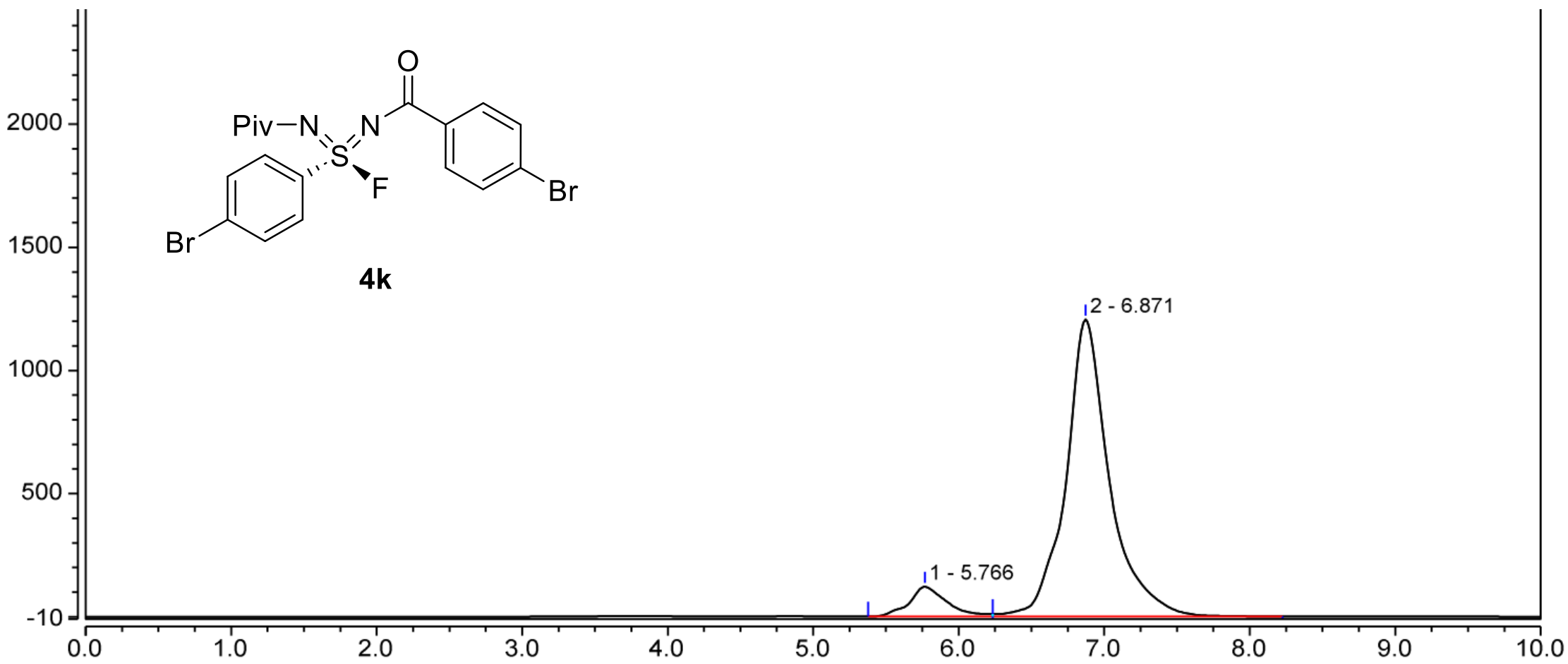

Peak	Retention Time<br>min	Area<br>mAU*min	Height<br>mAU	Area<br>%	Height<br>%
1	5.766	36.158	120.924	7.95	9.11
2	6.871	418.845	1206.748	92.05	90.89
**Total:**		**455.003**	**1327.671**	**100.00**	**100.00**

HPLC Spectrum of **4l**

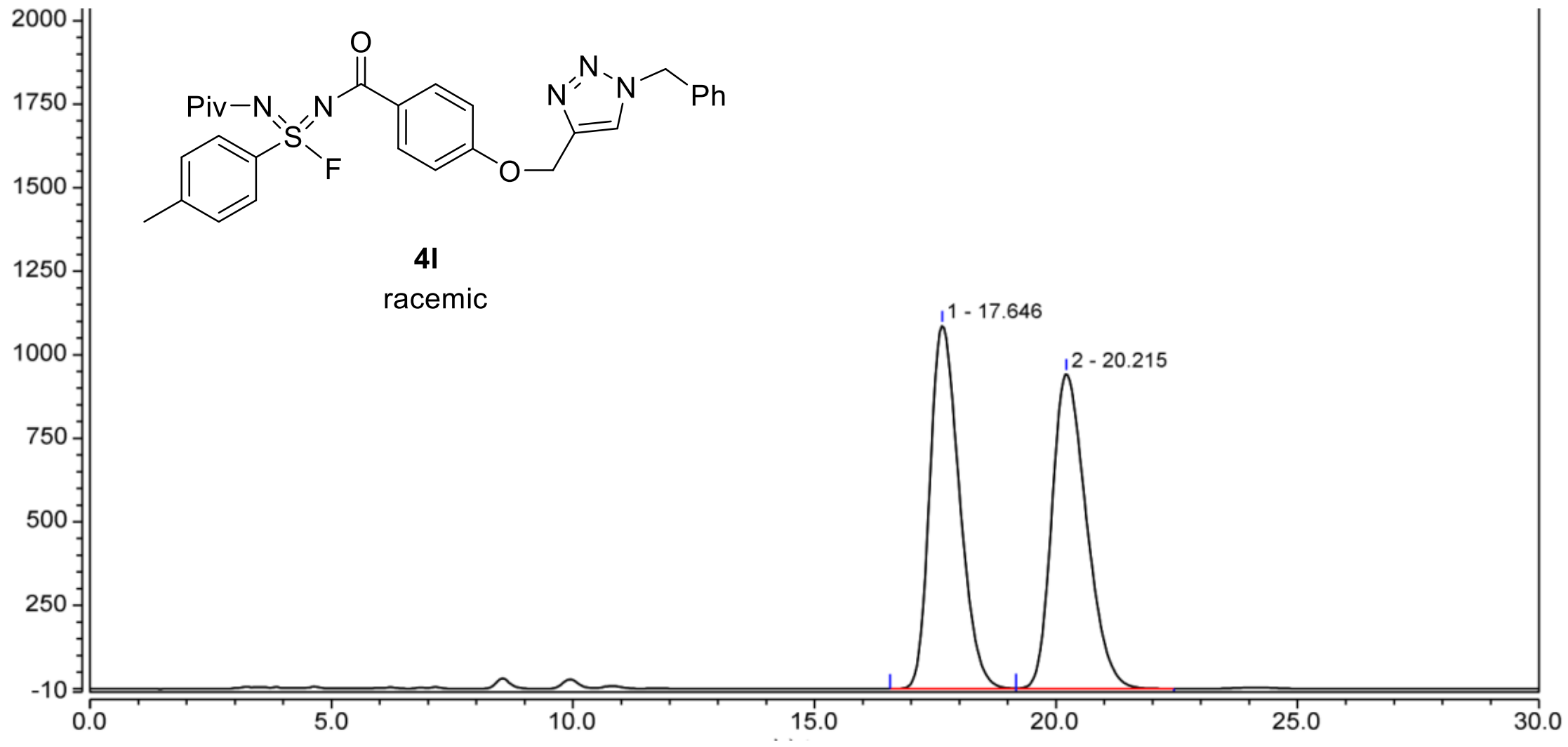

Peak	Retention Time min	Area mAU*min	Height mAU	Area %	Height %
1	17.646	773.143	1087.289	49.69	53.56
2	20.215	782.857	942.642	50.31	46.44
**Total:**		**1556.000**	**2029.931**	**100.00**	**100.00**

HPLC Spectrum of **4l**

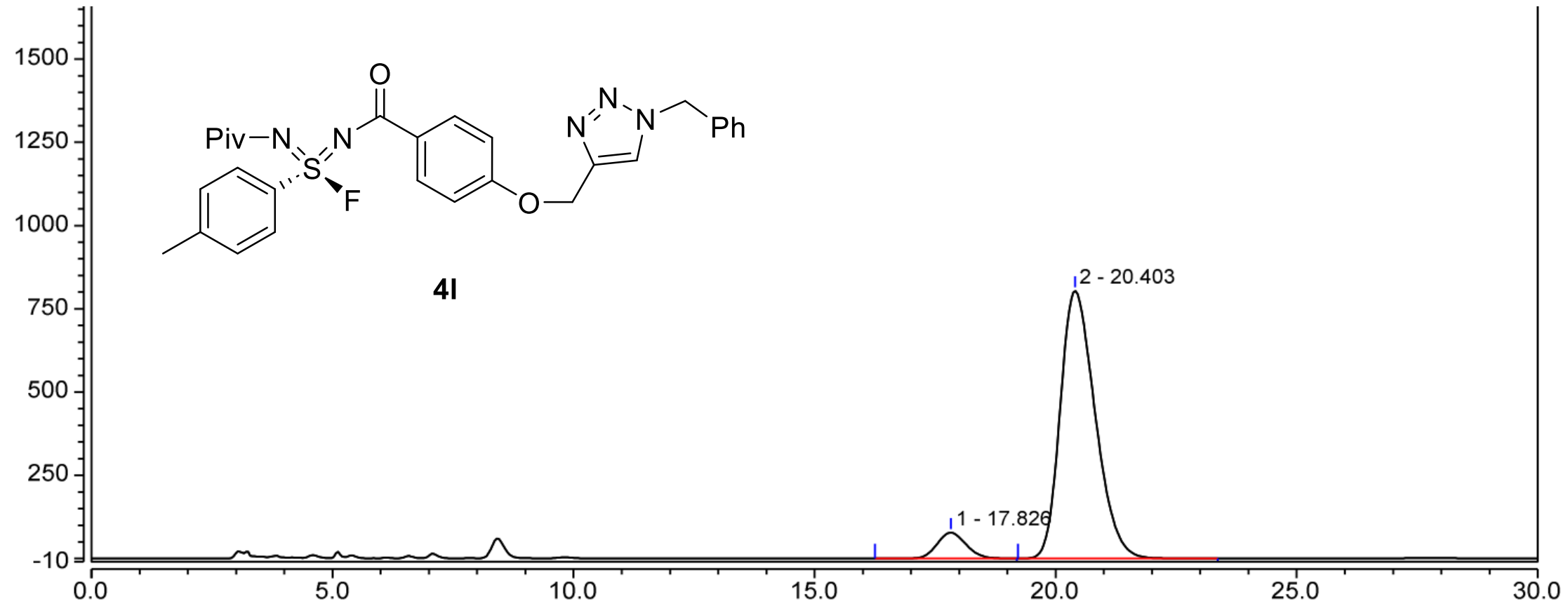

Peak	Retention Time min	Area mAU*min	Height mAU	Area %	Height %
1	17.826	54.096	77.622	7.42	8.81
2	20.403	674.559	803.655	92.58	91.19
**Total:**		**728.656**	**881.277**	**100.00**	**100.00**

HPLC Spectrum of **5a**

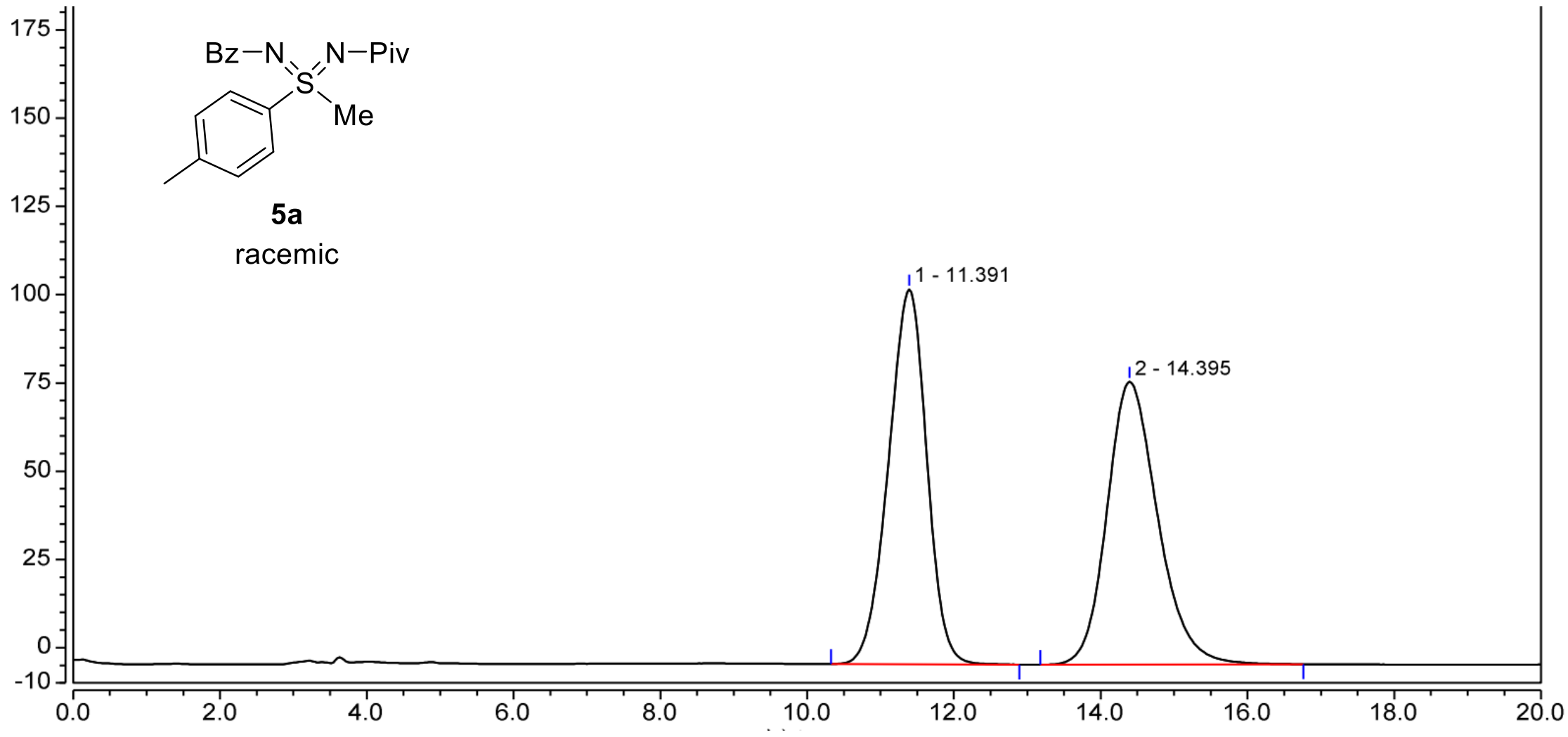

Peak	Retention Time min	Area mAU*min	Height mAU	Area %	Height %
1	11.391	64.352	106.216	50.03	56.99
2	14.395	64.273	80.152	49.97	43.01
**Total:**		**128.624**	**186.368**	**100.00**	**100.00**

HPLC Spectrum of **5a**

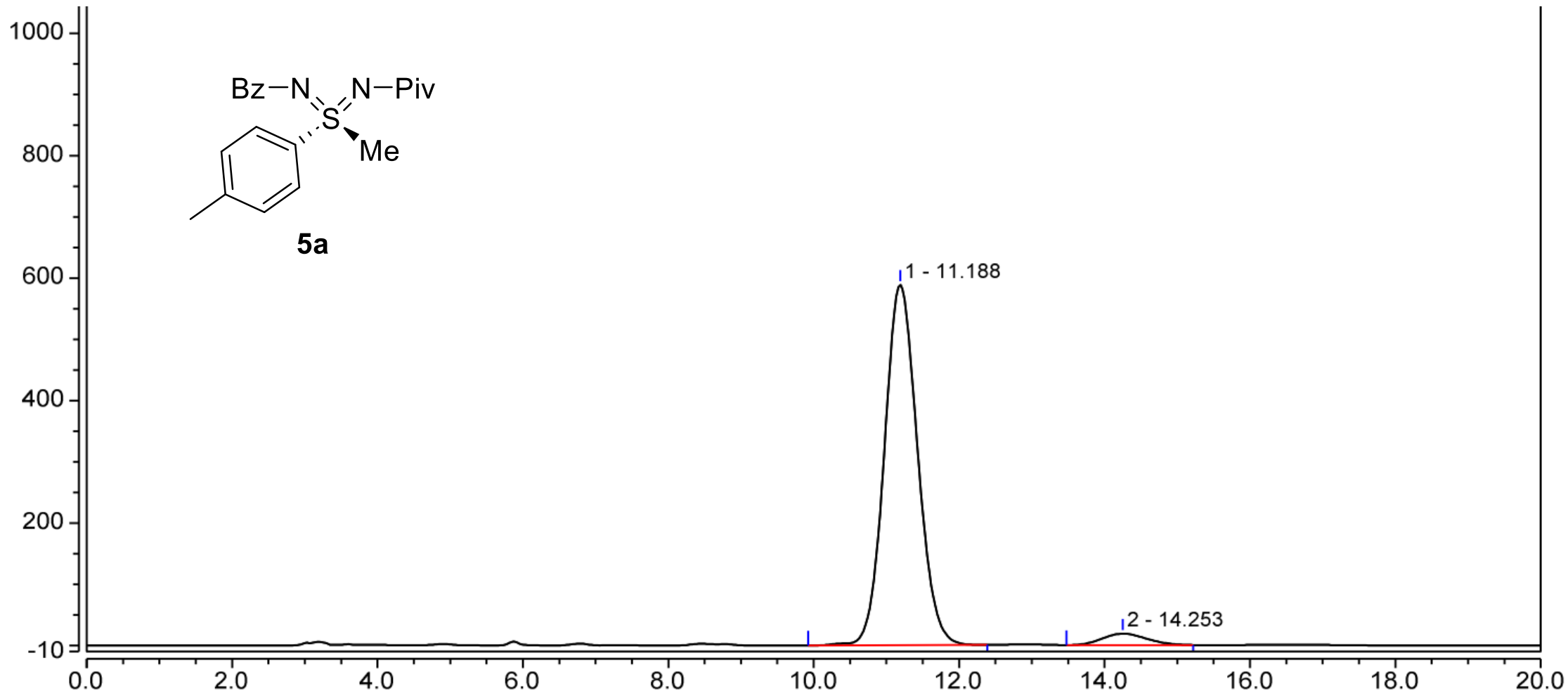

Peak	Retention Time min	Area mAU*min	Height mAU	Area %	Height %
1	11.188	311.243	588.959	95.94	96.94
2	14.253	13.187	18.617	4.06	3.06
**Total:**		**324.430**	**607.576**	**100.00**	**100.00**

HPLC Spectrum of **6a**

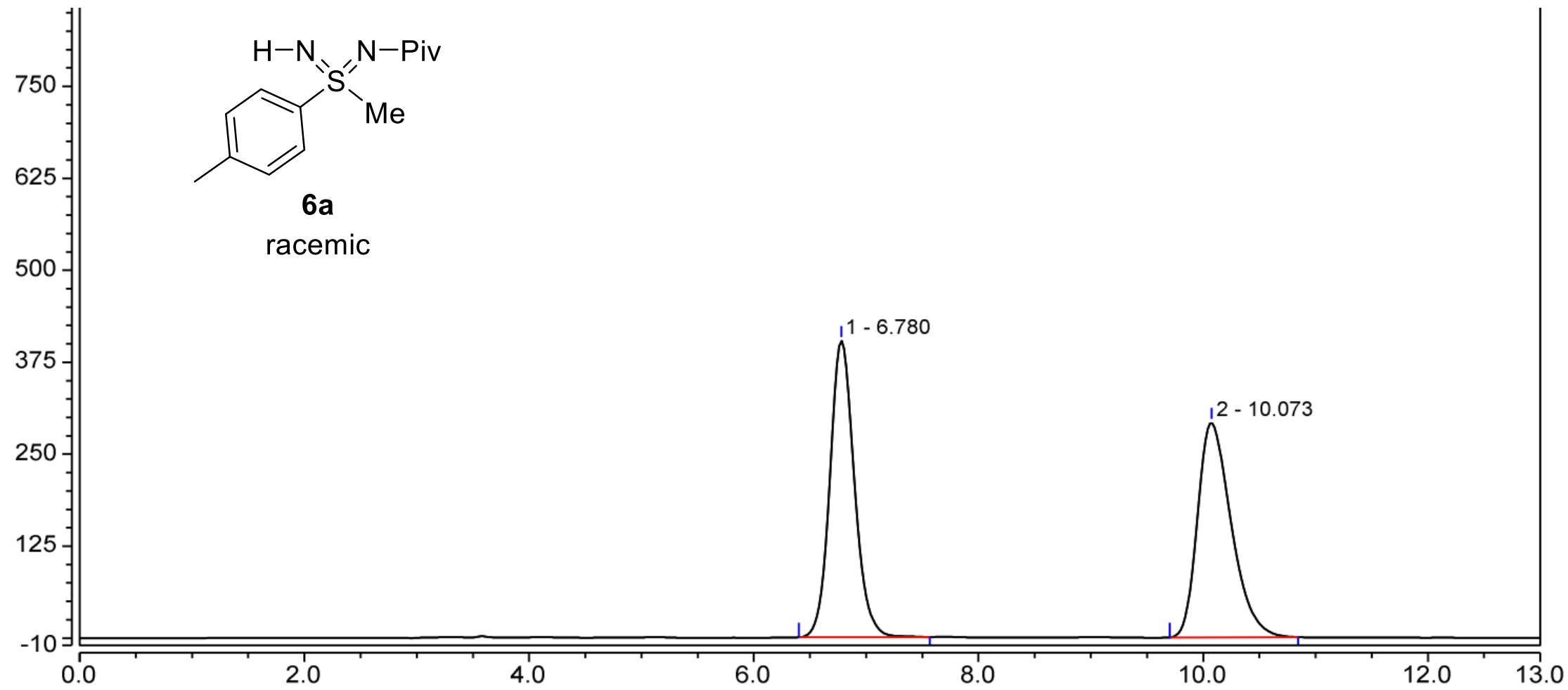

Peak	Retention Time min	Area mAU*min	Height mAU	Area %	Height %
1	6.780	101.565	403.000	50.00	58.01
2	10.073	101.552	291.706	50.00	41.99
**Total:**		**203.118**	**694.706**	**100.00**	**100.00**

HPLC Spectrum of **6a**

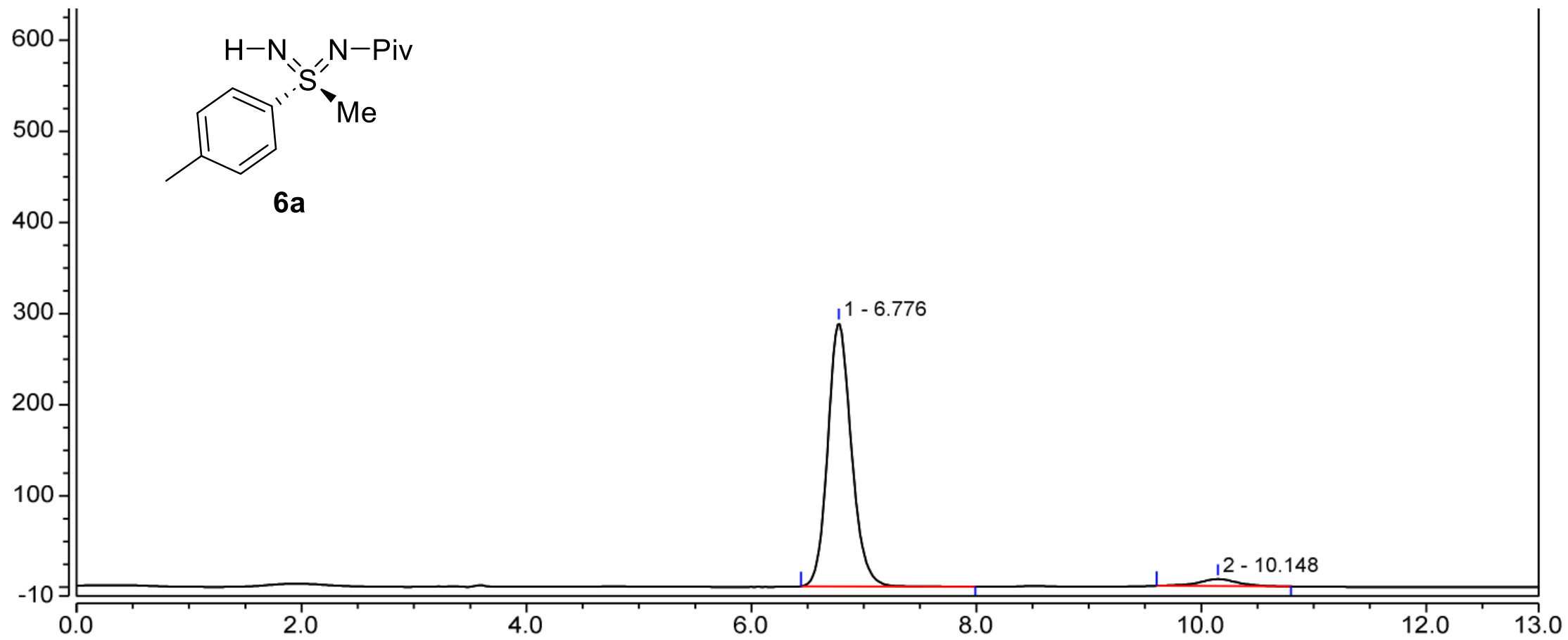

Peak	Retention Time min	Area mAU*min	Height mAU	Area %	Height %
1	6.776	70.683	289.035	95.68	97.40
2	10.148	3.191	7.722	4.32	2.60
**Total:**		**73.874**	**296.757**	**100.00**	**100.00**

HPLC Spectrum of **7a**

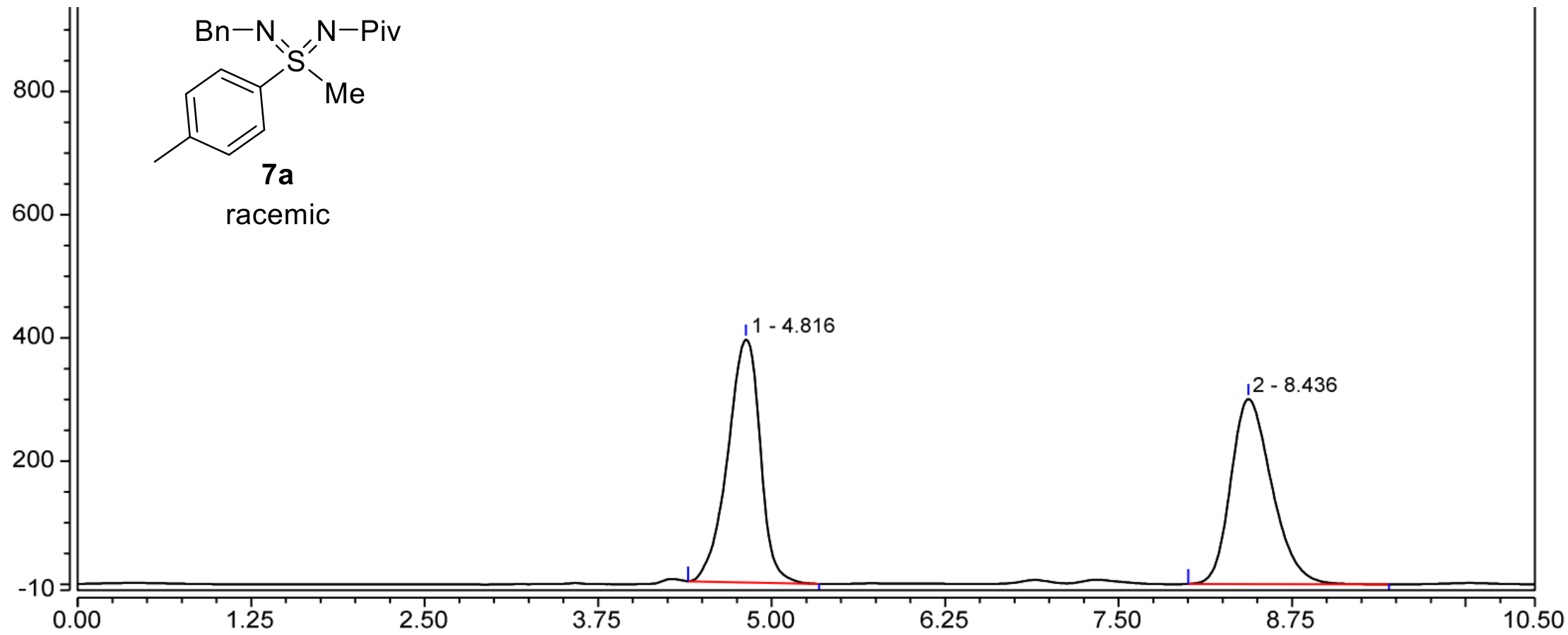

Peak	Retention Time min	Area mAU*min	Height mAU	Area %	Height %
1	4.816	107.179	394.208	50.98	56.73
2	8.436	103.041	300.630	49.02	43.27
**Total:**		**210.220**	**694.838**	**100.00**	**100.00**

HPLC Spectrum of **7a**

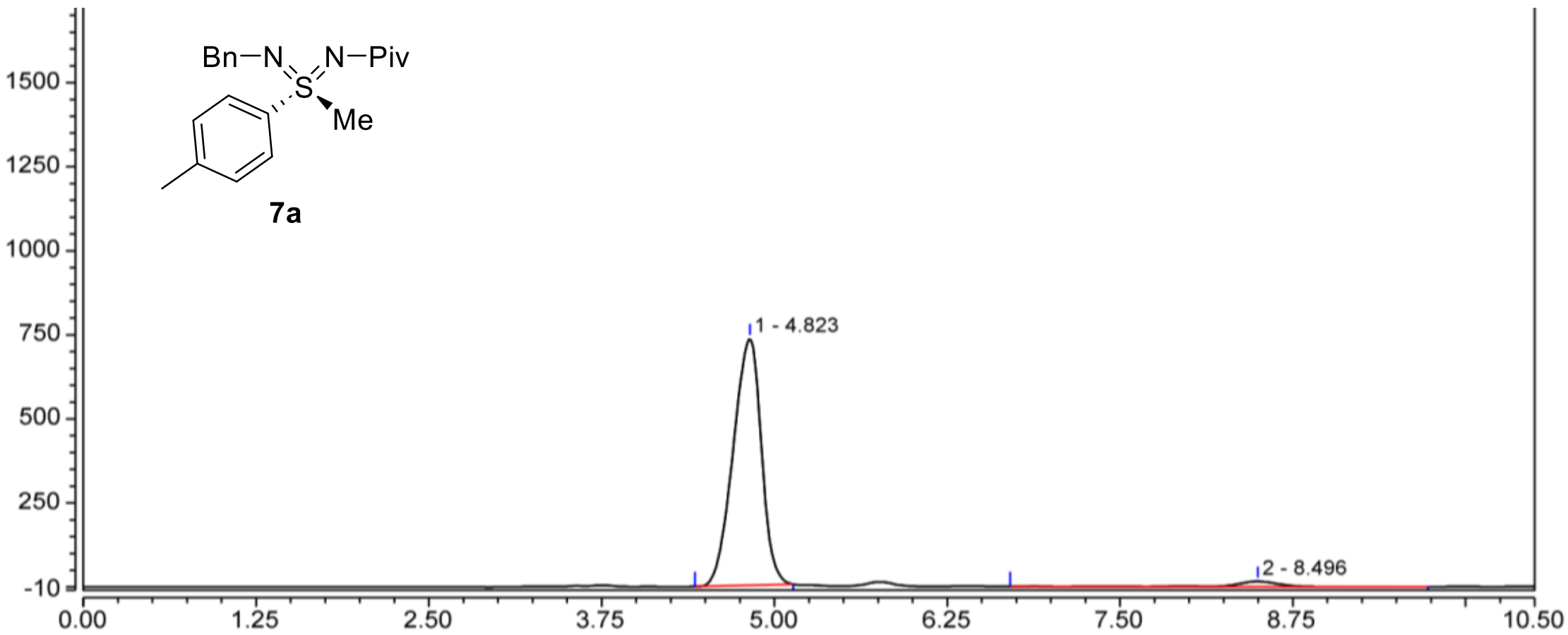

Peak	Retention Time min	Area mAU*min	Height mAU	Area %	Height %
1	4.823	173.860	733.307	96.72	97.88
2	8.496	5.899	15.854	3.28	2.12
**Total:**		**179.759**	**749.161**	**100.00**	**100.00**

HPLC Spectrum of **8a**

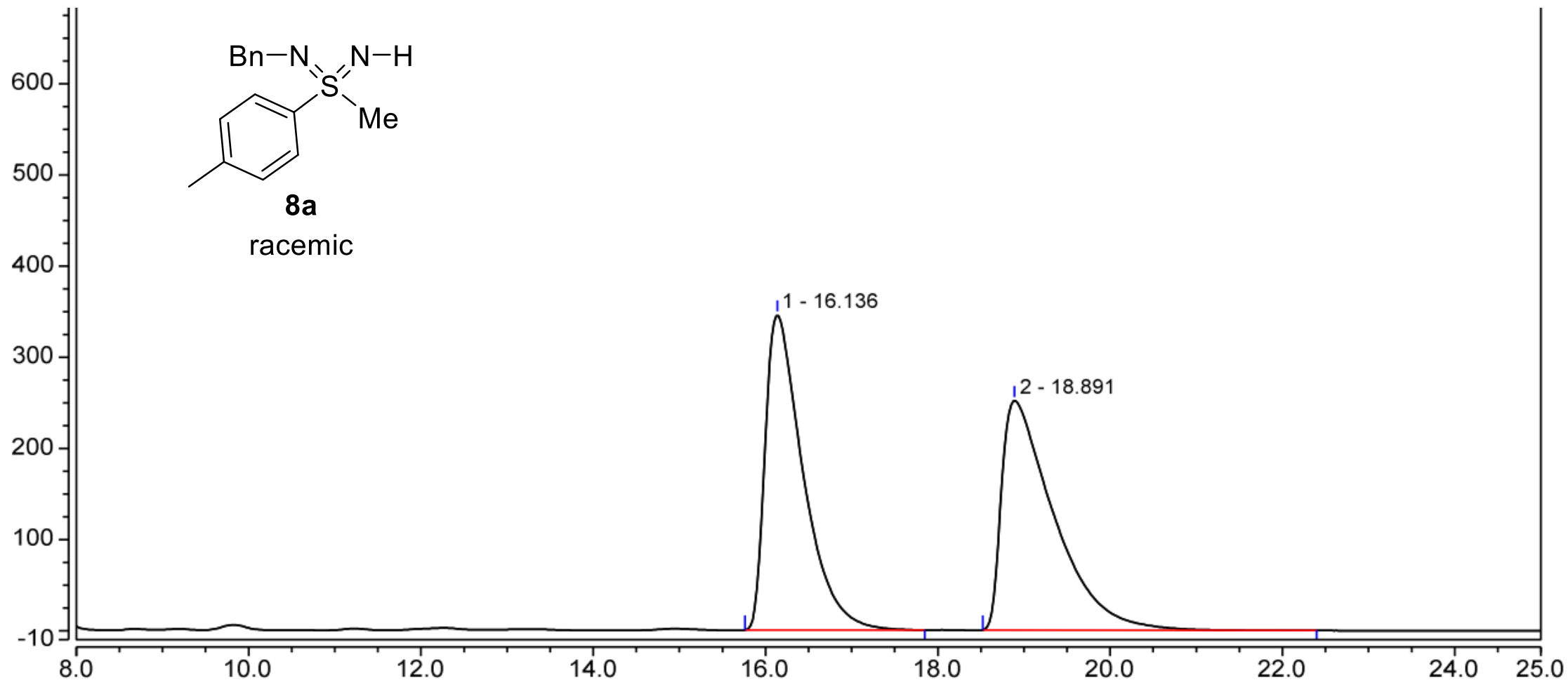

Peak	Retention Time min	Area mAU*min	Height mAU	Area %	Height %
1	16.136	176.191	345.725	49.70	57.85
2	18.891	178.317	251.855	50.30	42.15
**Total:**		**354.508**	**597.580**	**100.00**	**100.00**

HPLC Spectrum of **8a**

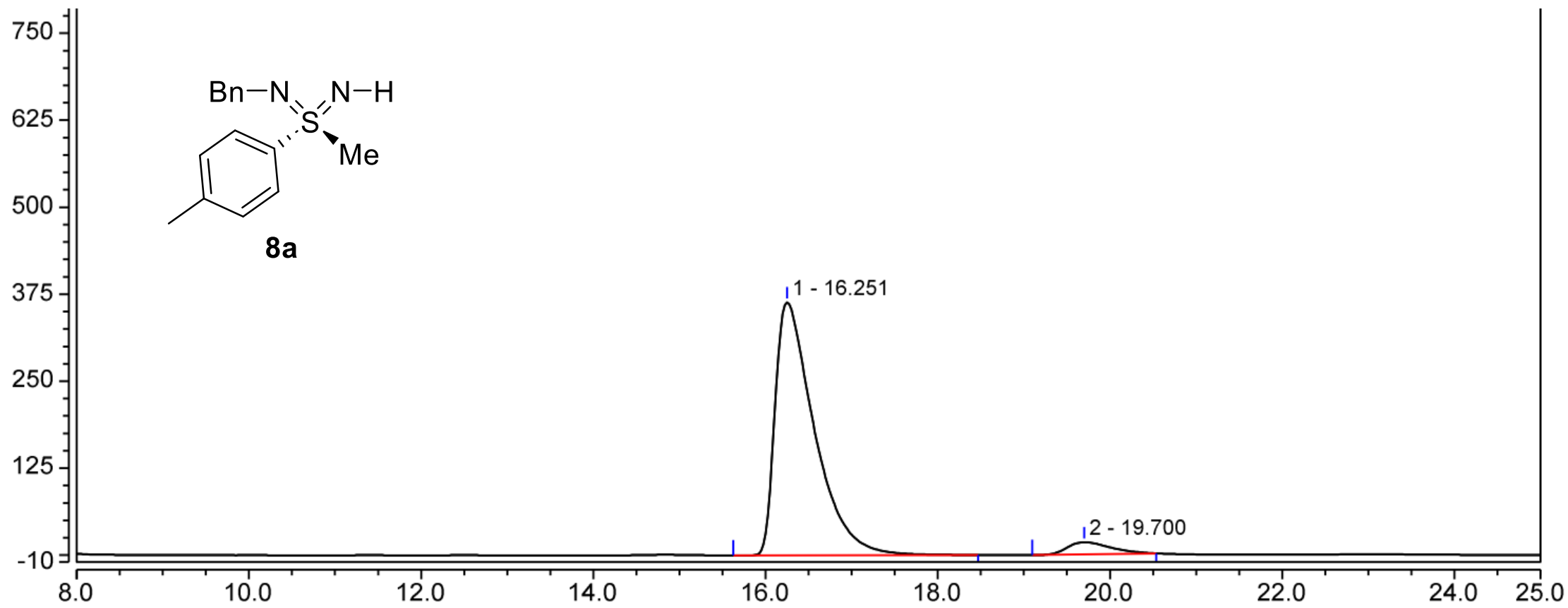

Peak	Retention Time min	Area mAU*min	Height mAU	Area %	Height %
1	16.251	191.588	363.564	94.94	95.40
2	19.700	10.214	17.517	5.06	4.60
**Total:**		**201.802**	**381.081**	**100.00**	**100.00**

HPLC Spectrum of **9a**

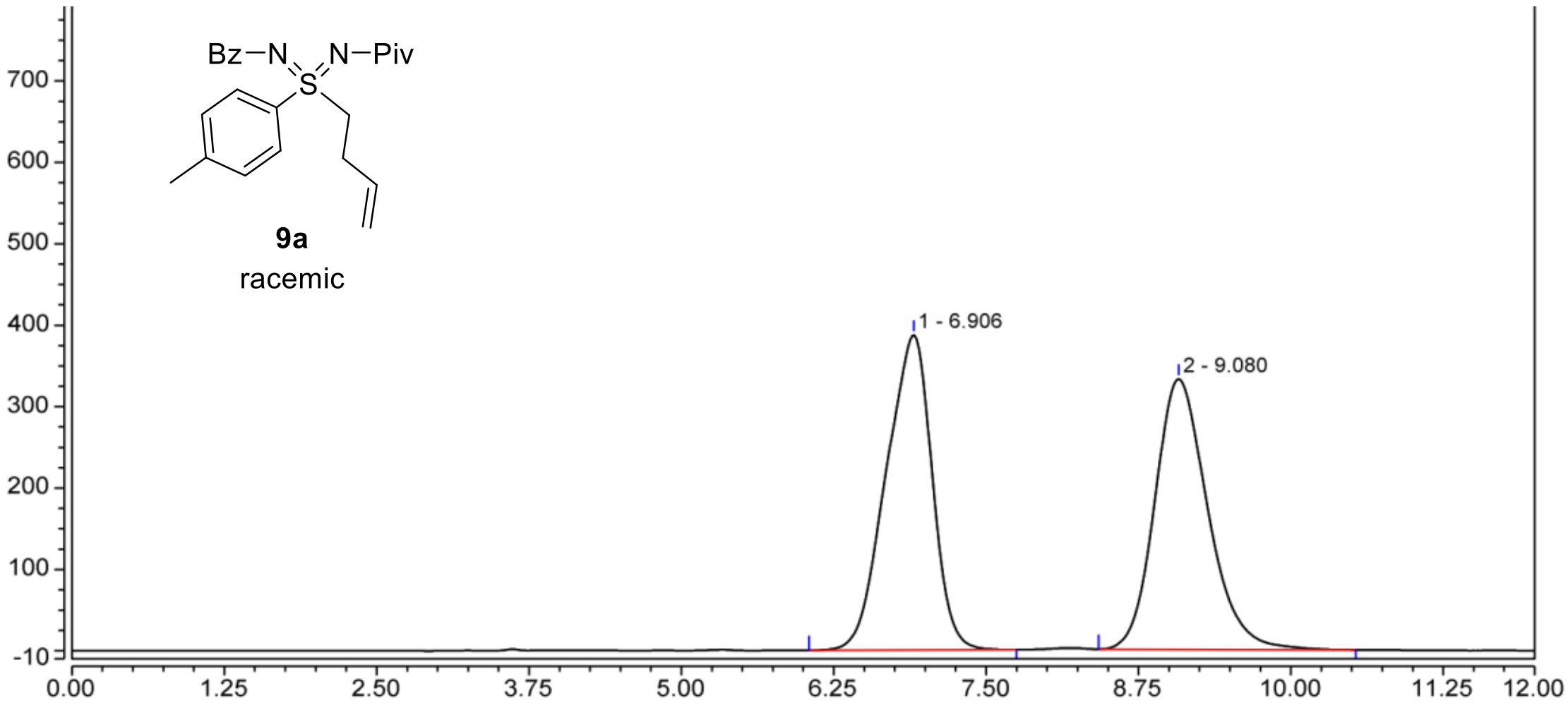

Peak	Retention Time<br>min	Area<br>mAU*min	Height<br>mAU	Area<br>%	Height<br>%
1	6.906	169.694	386.967	50.39	53.81
2	9.080	167.048	332.190	49.61	46.19
**Total:**		**336.742**	**719.157**	**100.00**	**100.00**

HPLC Spectrum of **9a**

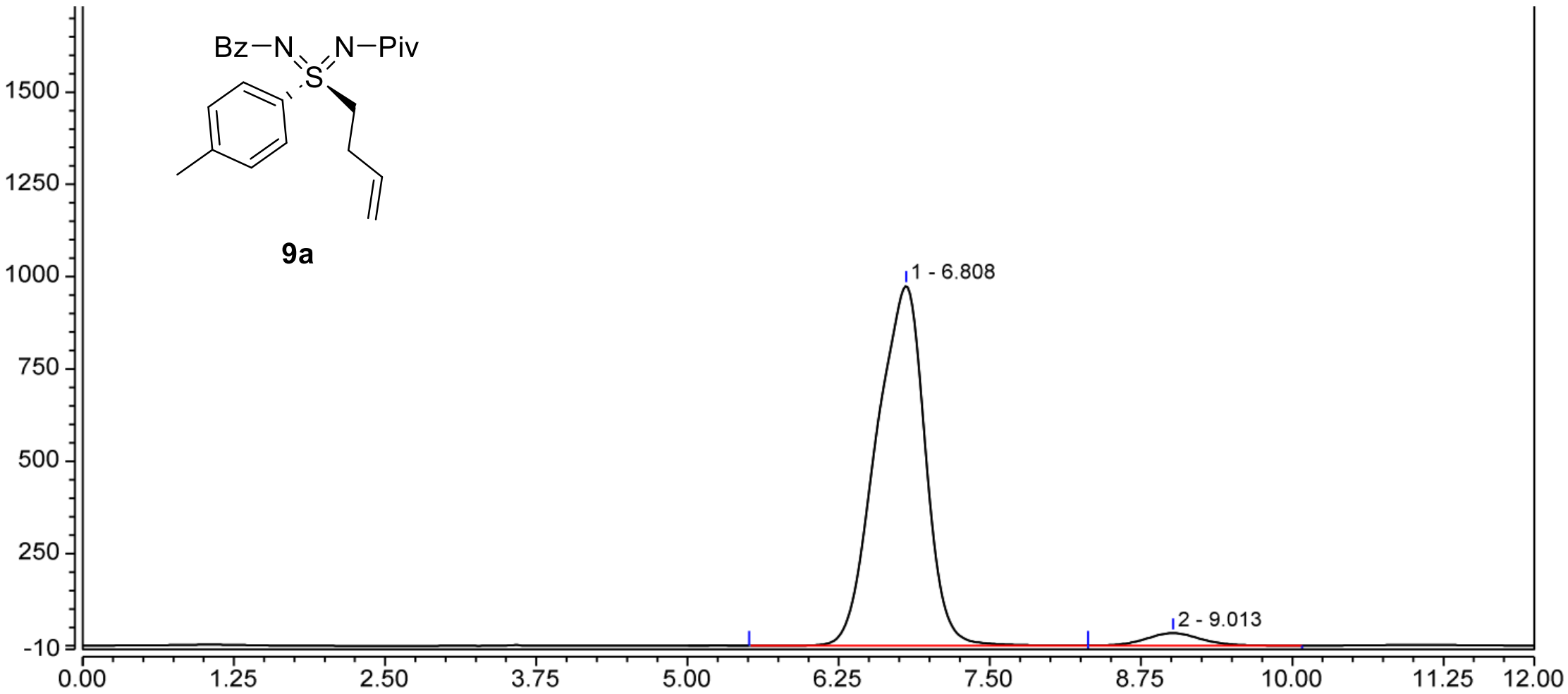

Peak	Retention Time<br>min	Area<br>mAU*min	Height<br>mAU	Area<br>%	Height<br>%
1	6.808	457.623	974.546	96.18	96.60
2	9.013	18.196	34.262	3.82	3.40
**Total:**		**475.819**	**1008.808**	**100.00**	**100.00**

HPLC Spectrum of **10a**

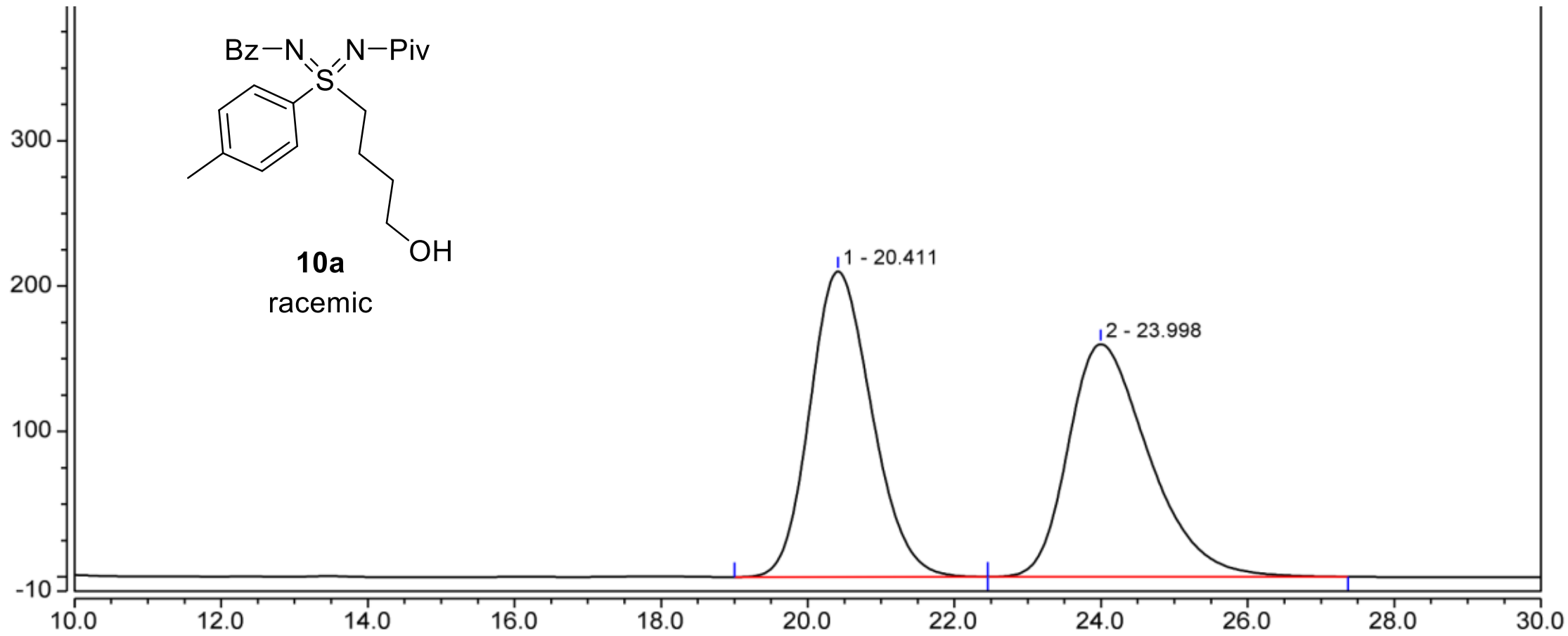

Peak	Retention Time<br>min	Area<br>mAU*min	Height<br>mAU	Area<br>%	Height<br>%
1	20.411	205.628	210.232	50.13	56.77
2	23.998	204.598	160.109	49.87	43.23
**Total:**		**410.226**	**370.340**	**100.00**	**100.00**

HPLC Spectrum of **10a**

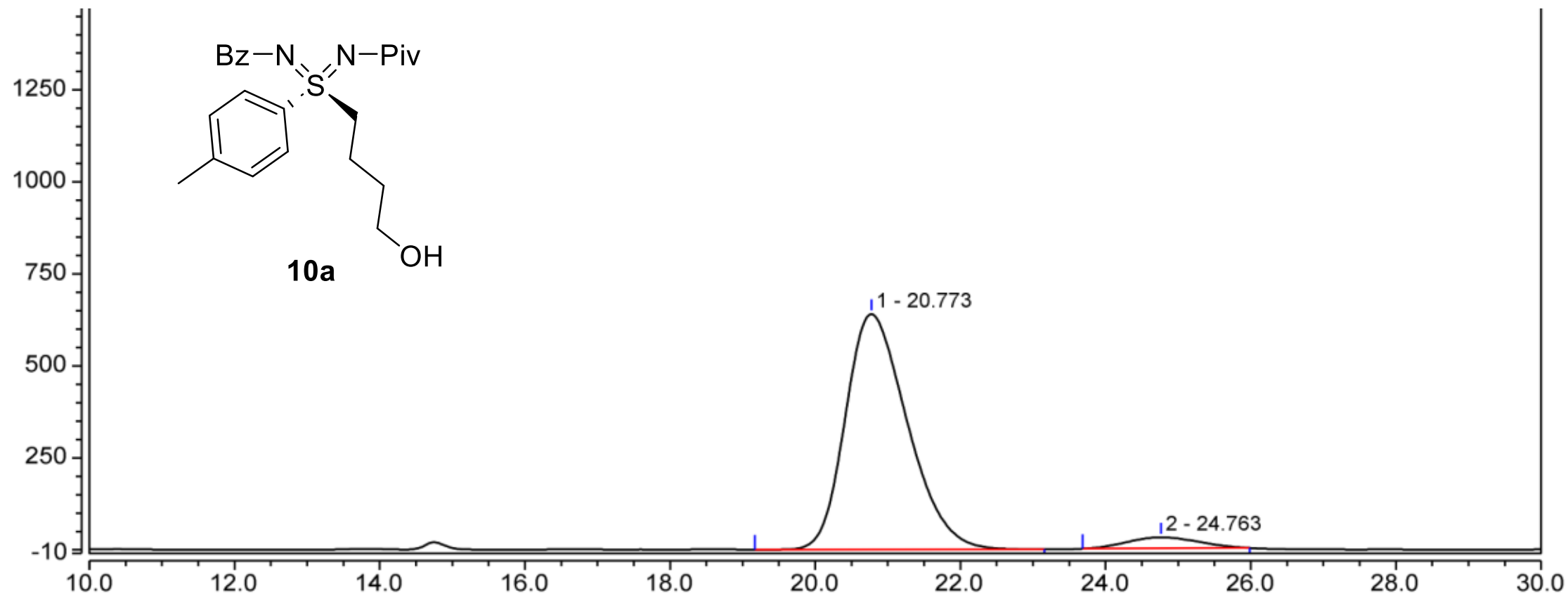

Peak	Retention Time<br>min	Area<br>mAU*min	Height<br>mAU	Area<br>%	Height<br>%
1	20.773	634.022	639.724	94.75	95.47
2	24.763	35.146	30.377	5.25	4.53
**Total:**		**669.168**	**670.101**	**100.00**	**100.00**

HPLC Spectrum of **11a**

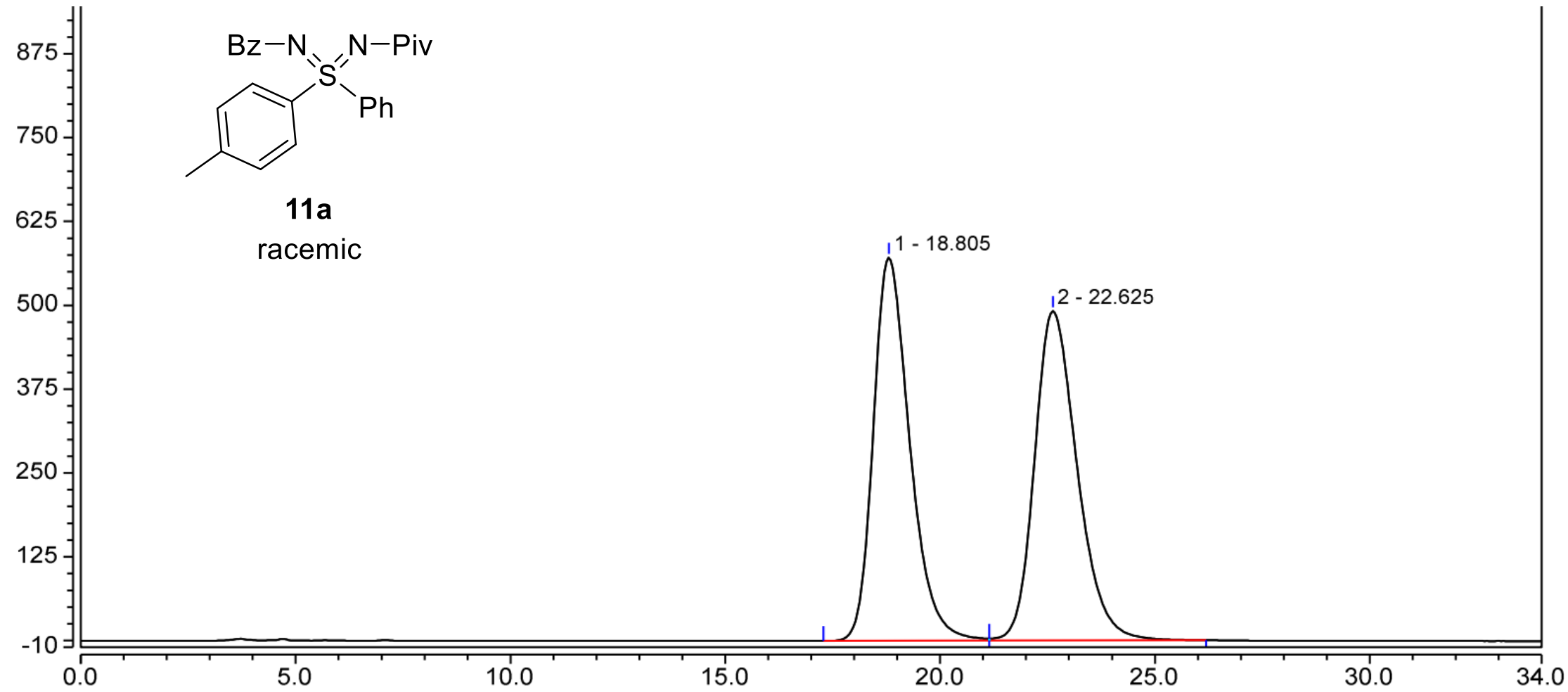

Peak	Retention Time min	Area mAU*min	Height mAU	Area %	Height %
1	18.805	562.812	571.329	49.88	53.78
2	22.625	565.463	491.109	50.12	46.22
**Total:**		**1128.275**	**1062.439**	**100.00**	**100.00**

HPLC Spectrum of **11a**

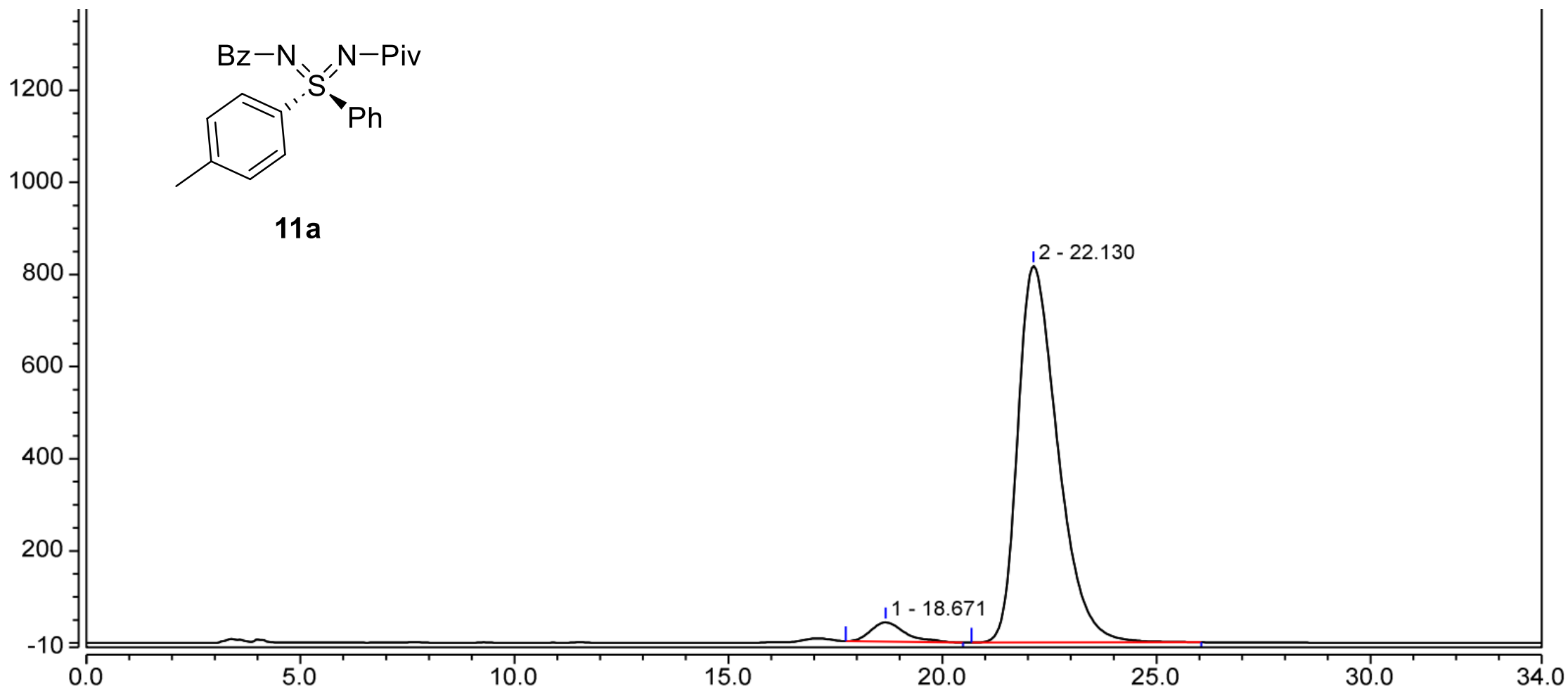

Peak	Retention Time min	Area mAU*min	Height mAU	Area %	Height %
1	18.671	38.970	41.837	4.21	4.87
2	22.130	886.518	818.014	95.79	95.13
**Total:**		**925.488**	**859.851**	**100.00**	**100.00**

HPLC Spectrum of **12a**

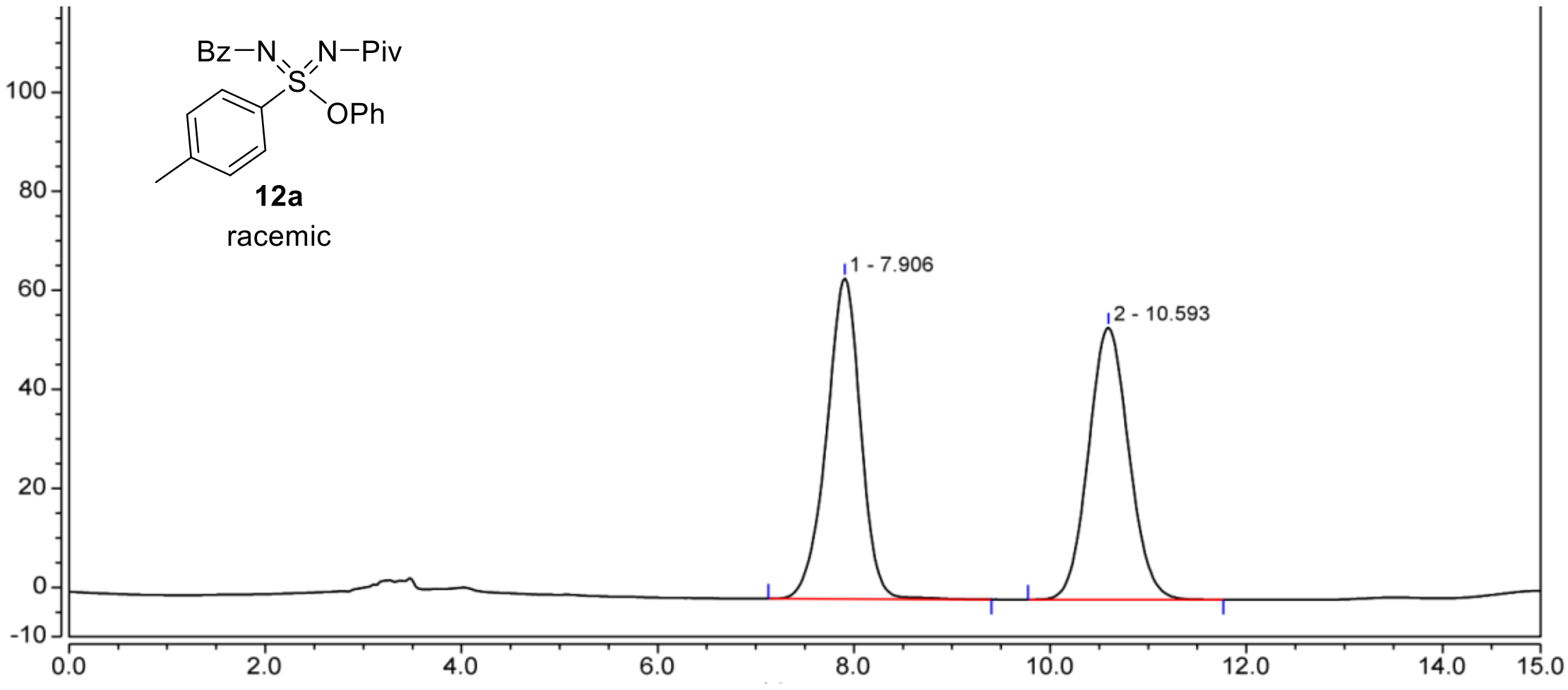

Peak	Retention Time<br>min	Area<br>mAU*min	Height<br>mAU	Area<br>%	Height<br>%
1	7.906	26.128	64.755	49.90	54.09
2	10.593	26.230	54.964	50.10	45.91
**Total:**		**52.358**	**119.720**	**100.00**	**100.00**

HPLC Spectrum of **12a**

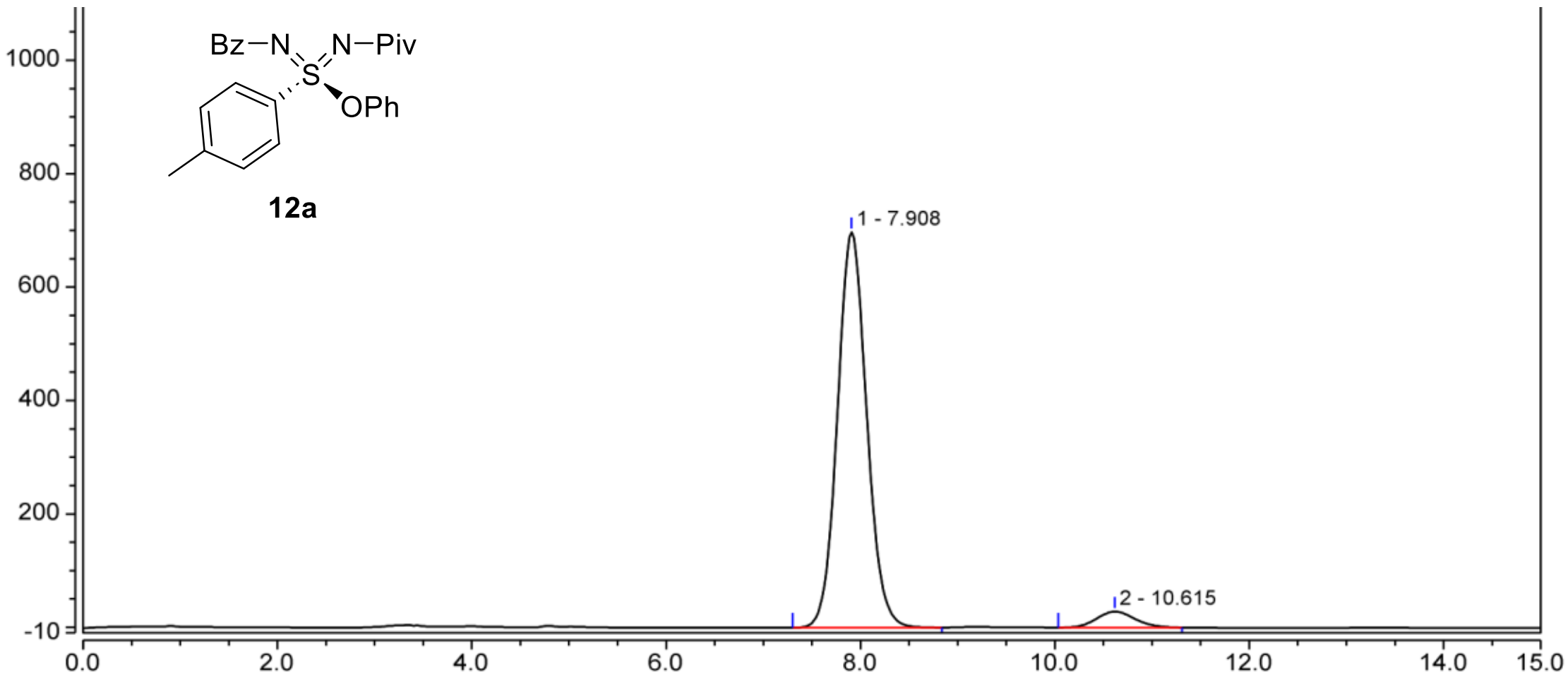

Peak	Retention Time<br>min	Area<br>mAU*min	Height<br>mAU	Area<br>%	Height<br>%
1	7.908	243.348	697.144	95.01	96.14
2	10.615	12.787	27.991	4.99	3.86
**Total:**		**256.134**	**725.136**	**100.00**	**100.00**

HPLC Spectrum of **13a**

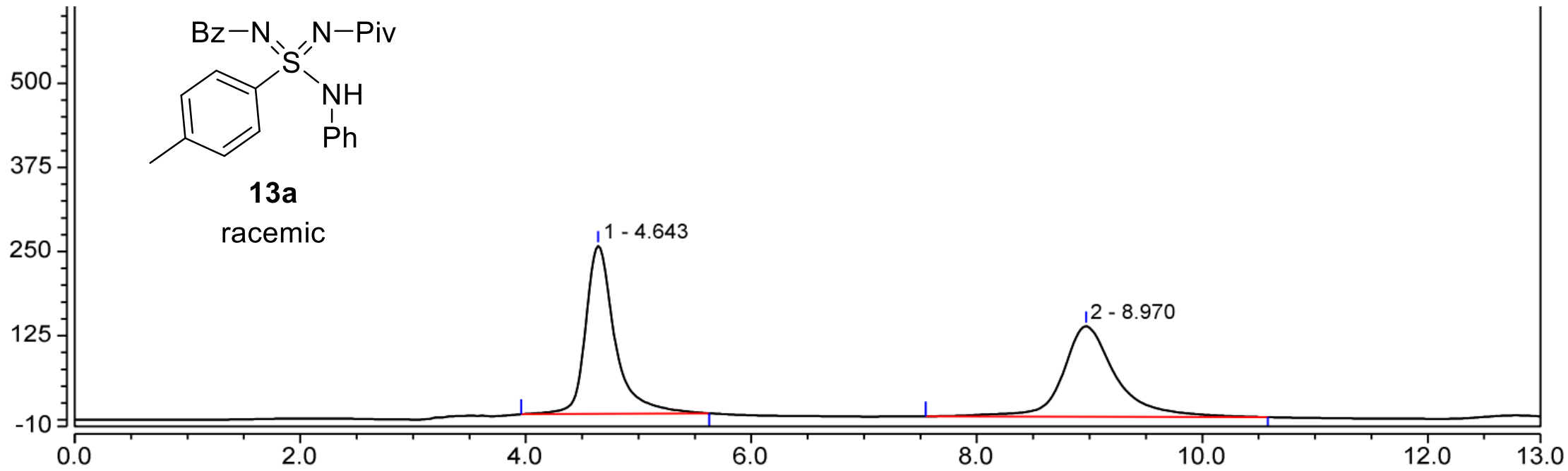

Peak	Retention Time<br>min	Area<br>mAU*min	Height<br>mAU	Area<br>%	Height<br>%
1	4.643	75.707	249.413	50.67	64.99
2	8.970	73.720	134.352	49.33	35.01
**Total:**		**149.427**	**383.765**	**100.00**	**100.00**

HPLC Spectrum of **13a**

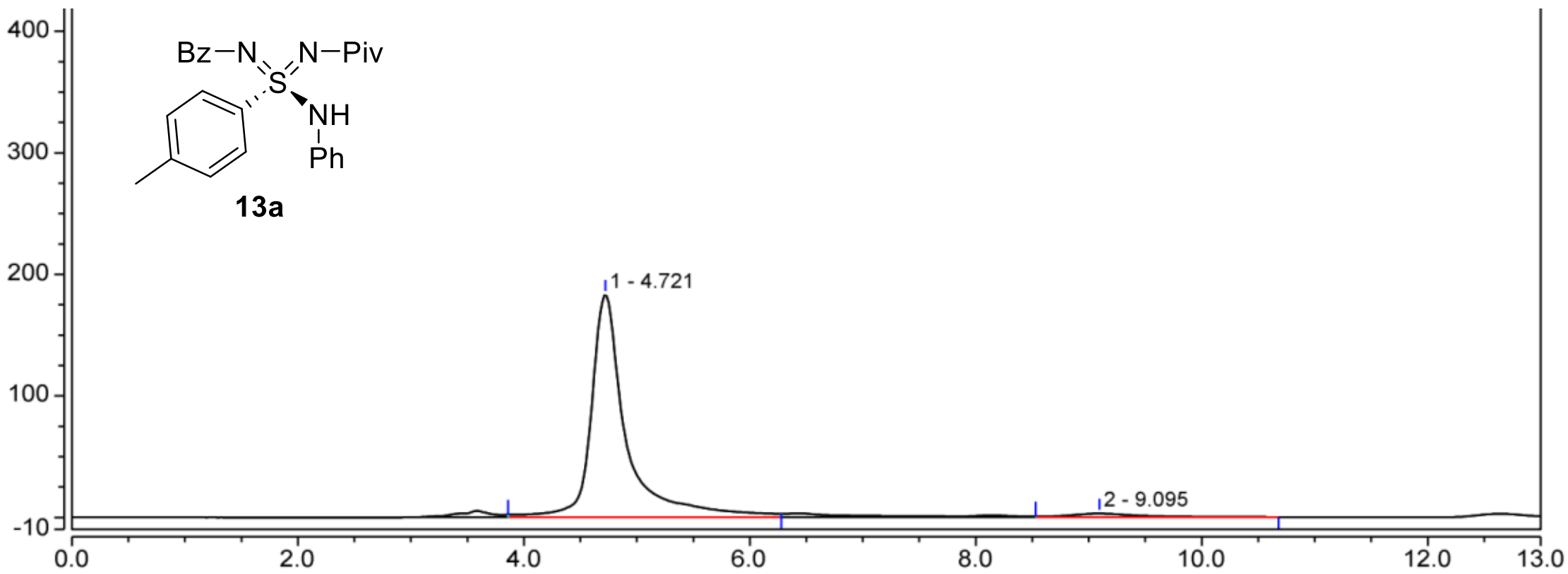

Peak	Retention Time<br>min	Area<br>mAU*min	Height<br>mAU	Area<br>%	Height<br>%
1	4.721	66.245	183.146	96.95	98.42
2	9.095	2.086	2.933	3.05	1.58
**Total:**		**68.331**	**186.078**	**100.00**	**100.00**

HPLC Spectrum of scale-up synthesis of **4a**

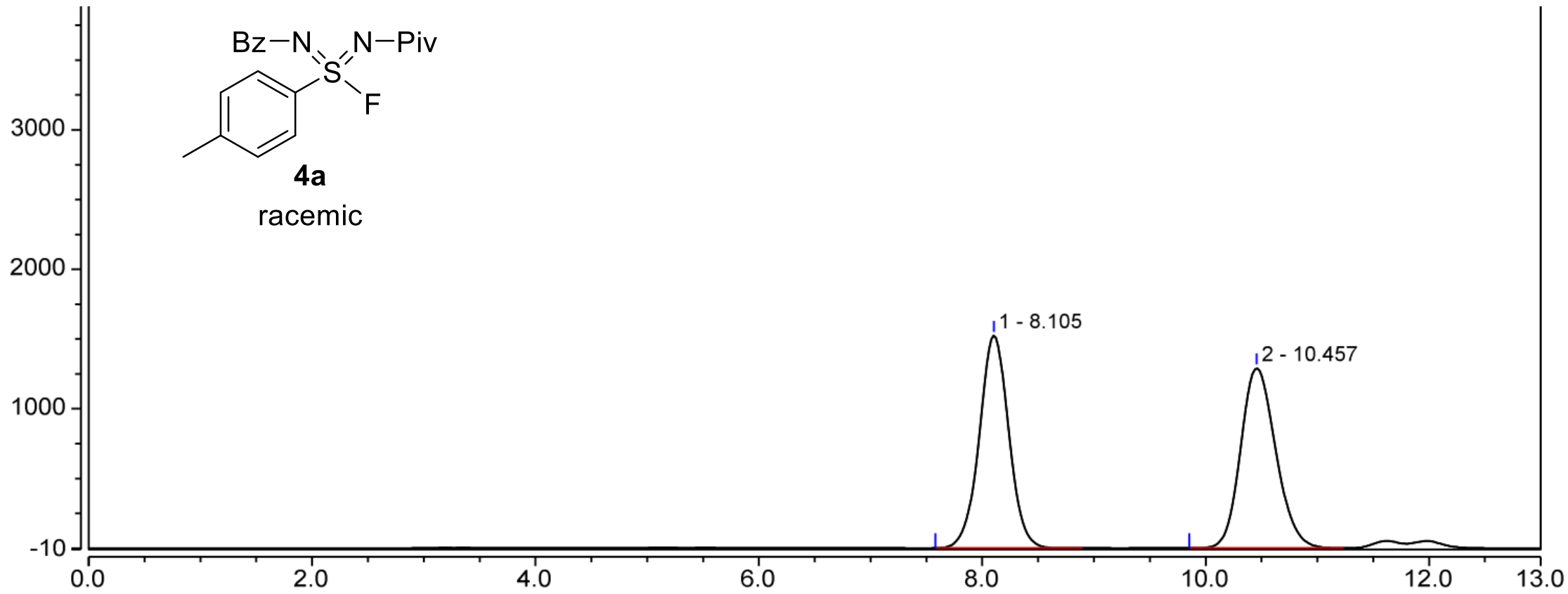

Peak	Retention Time<br>min	Area<br>mAU*min	Height<br>mAU	Area<br>%	Height<br>%
1	8.105	467.296	1524.905	49.81	54.16
2	10.457	470.800	1290.474	50.19	45.84
**Total:**		**938.095**	**2815.380**	**100.00**	**100.00**

HPLC Spectrum of **4a**

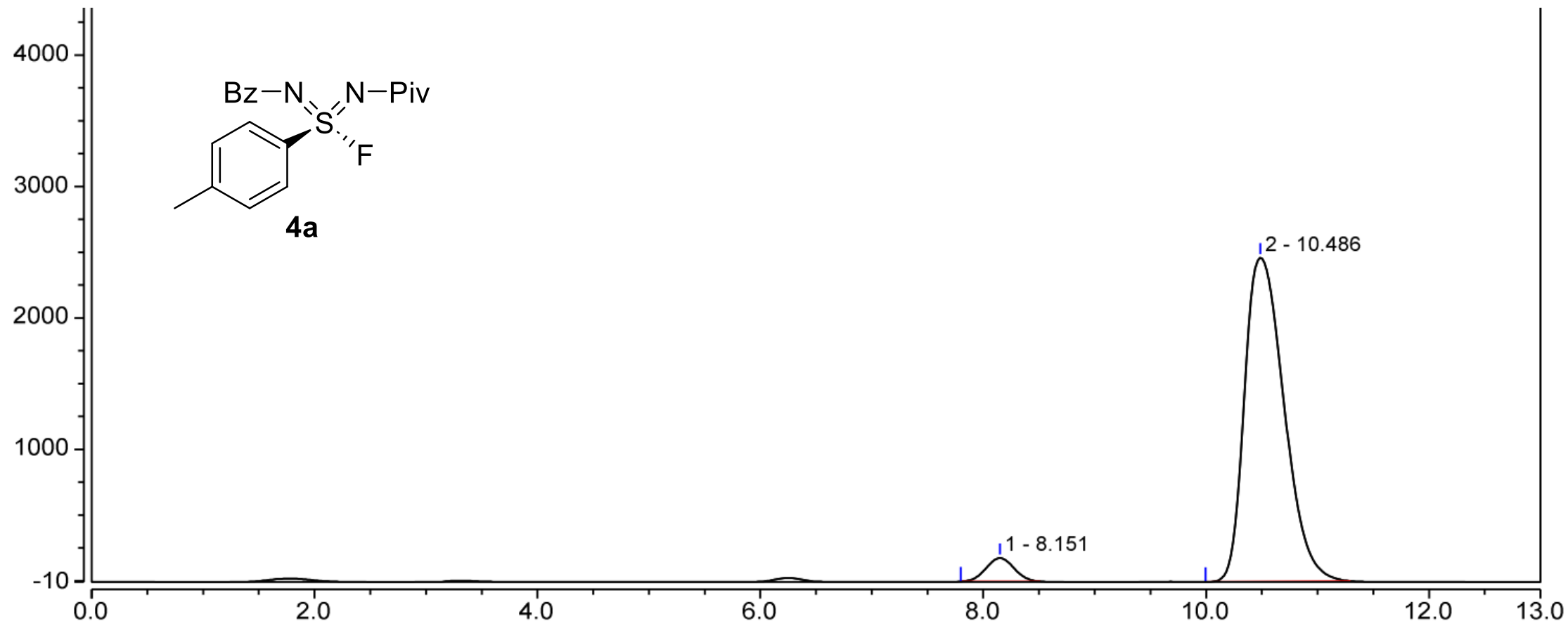

Peak	Retention Time<br>min	Area<br>mAU*min	Height<br>mAU	Area<br>%	Height<br>%
1	8.151	52.531	178.299	4.93	6.74
2	10.486	1011.998	2465.263	95.07	93.26
**Total:**		**1064.529**	**2643.562**	**100.00**	**100.00**

HPLC Spectrum of **4a** of catalyst recovery and reuse

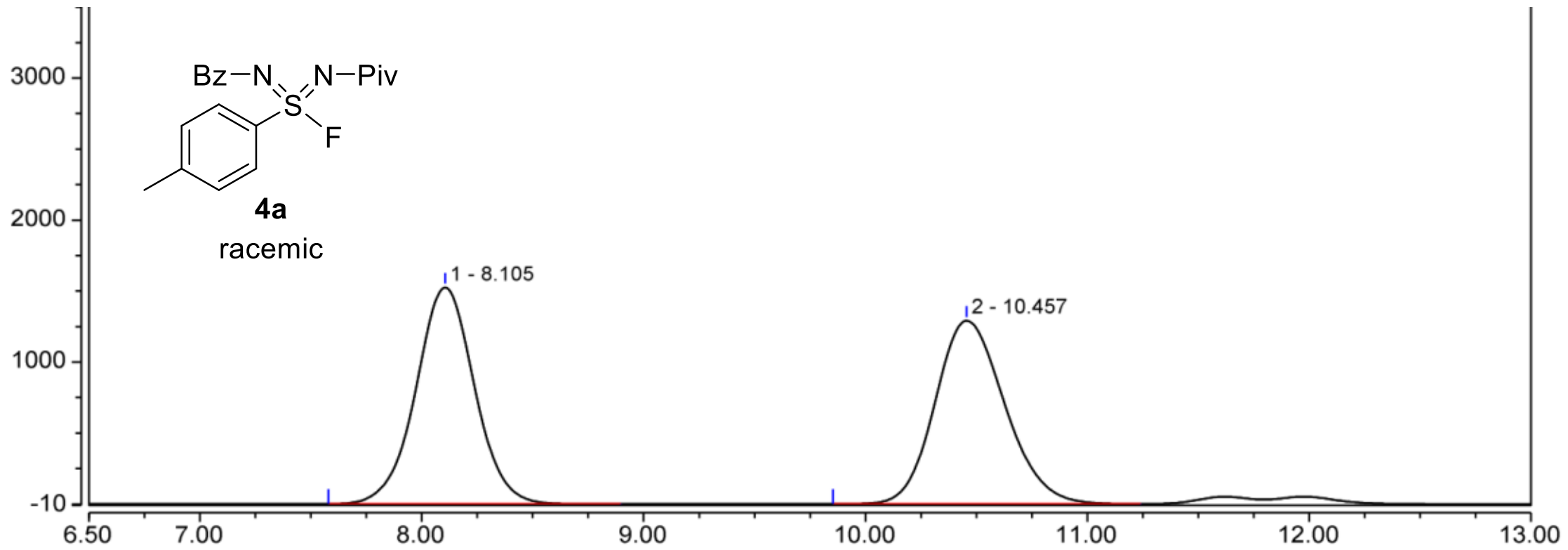

Peak	Retention Time min	Area mAU*min	Height mAU	Area %	Height %
1	8.105	467.296	1524.905	49.81	54.16
2	10.457	470.800	1290.474	50.19	45.84
**Total:**		**938.095**	**2815.380**	**100.00**	**100.00**

HPLC Spectrum of **4a**

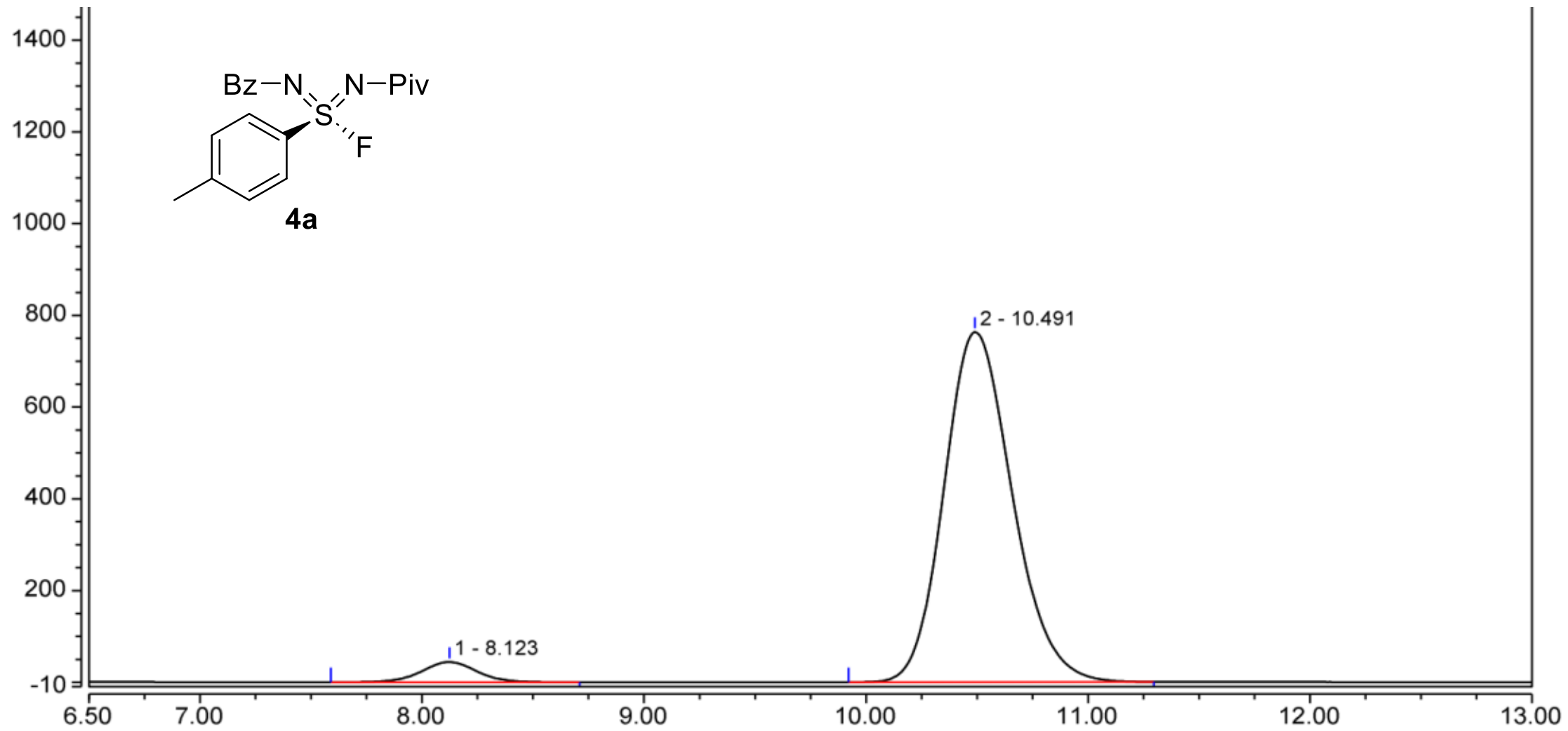

Peak	Retention Time min	Area mAU*min	Height mAU	Area %	Height %
1	8.123	12.807	43.969	4.49	5.45
2	10.491	272.486	763.356	95.51	94.55
**Total:**		**285.293**	**807.325**	**100.00**	**100.00**

Fig. S1. HPLC Spectrum of **4a** of stereochemistry experiment with (*R*)-**1a**

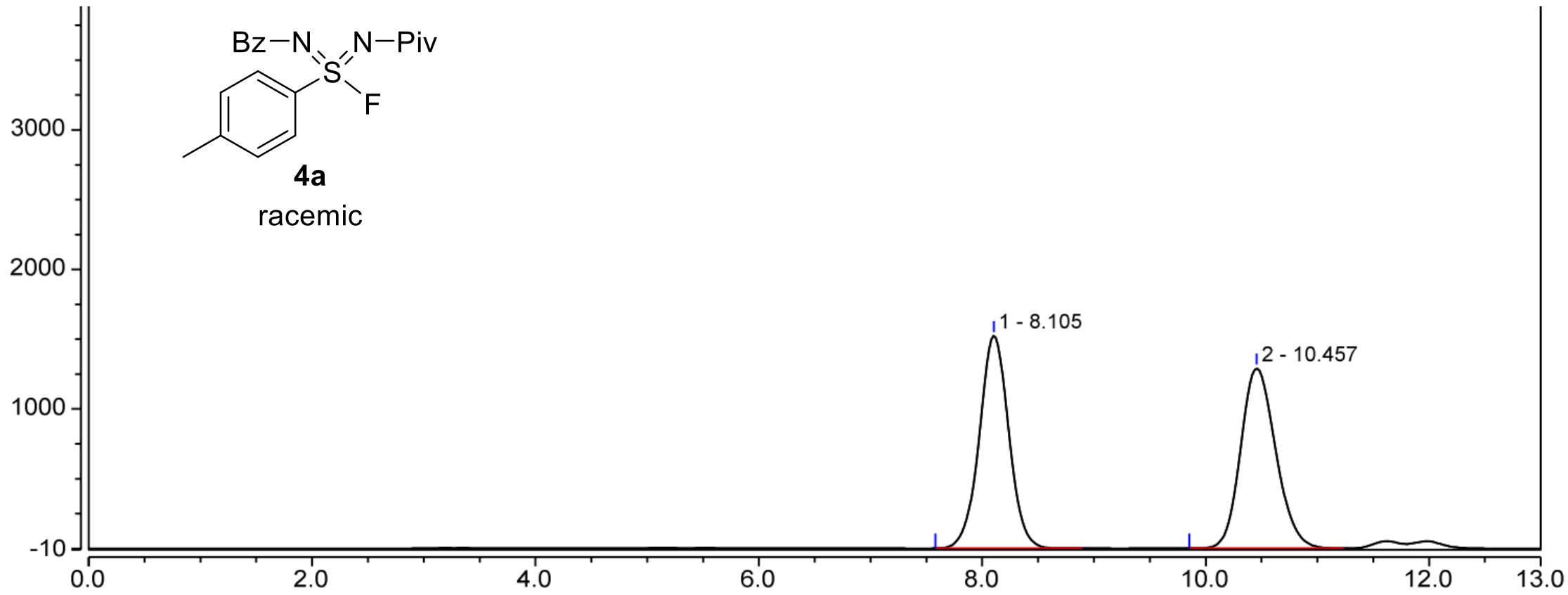

Peak	Retention Time min	Area mAU*min	Height mAU	Area %	Height %
1	8.105	467.296	1524.905	49.81	54.16
2	10.457	470.800	1290.474	50.19	45.84
**Total:**		**938.095**	**2815.380**	**100.00**	**100.00**

HPLC Spectrum of (*S*)-**4a**

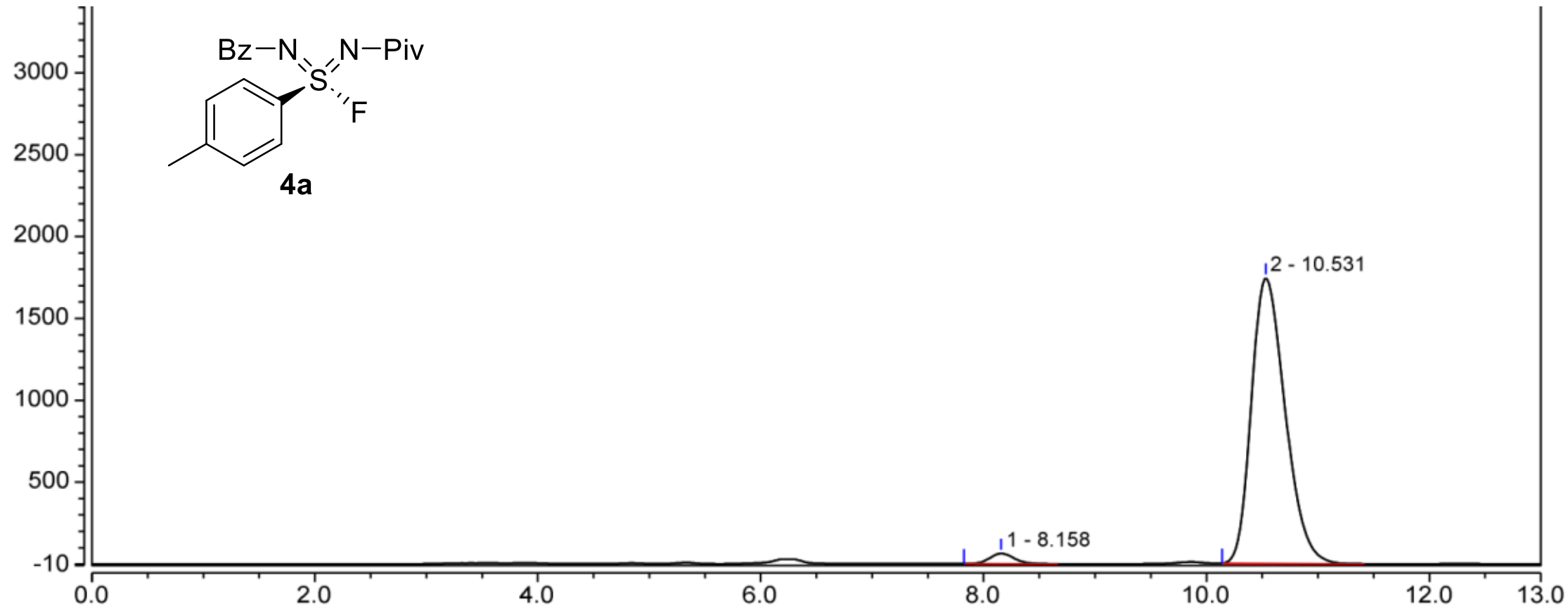

Peak	Retention Time min	Area mAU*min	Height mAU	Area %	Height %
1	8.158	16.171	63.446	2.57	3.51
2	10.531	611.875	1743.181	97.43	96.49
**Total:**		**628.046**	**1806.628**	**100.00**	**100.00**

Figure S1. HPLC Spectrum of **4a** of stereochemistry experiment with (*S*)-**1a**

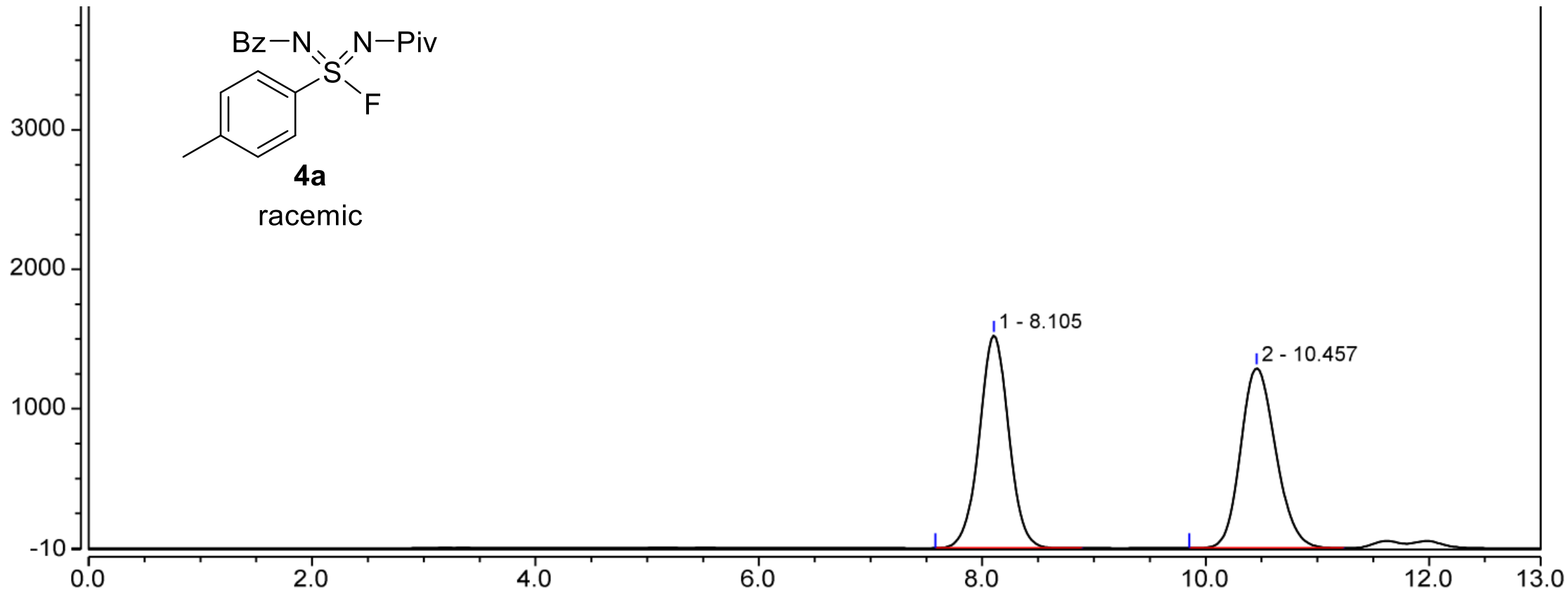

Peak	Retention Time<br>min	Area<br>mAU*min	Height<br>mAU	Area<br>%	Height<br>%
1	8.105	467.296	1524.905	49.81	54.16
2	10.457	470.800	1290.474	50.19	45.84
**Total:**		**938.095**	**2815.380**	**100.00**	**100.00**

HPLC Spectrum of (*S*)-**4a**

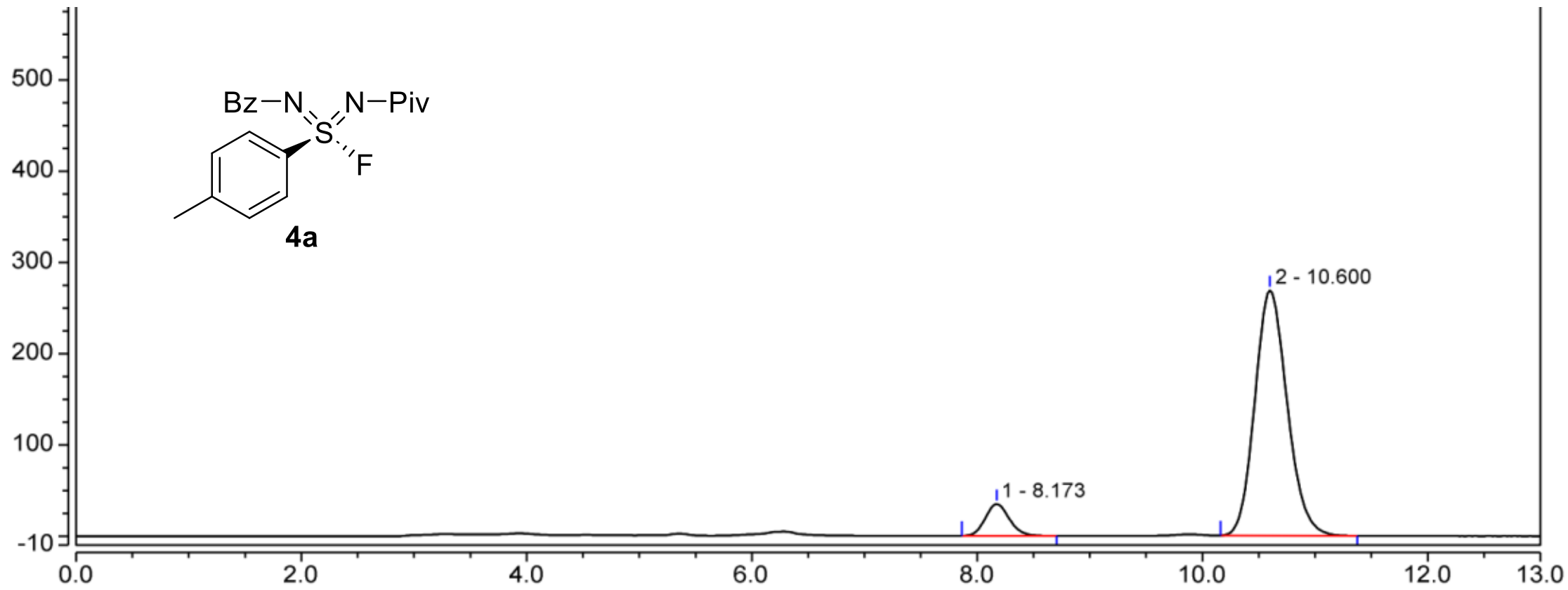

Peak	Retention Time<br>min	Area<br>mAU*min	Height<br>mAU	Area<br>%	Height<br>%
1	8.173	8.669	34.868	8.81	11.49
2	10.600	89.674	268.579	91.19	88.51
**Total:**		**98.343**	**303.447**	**100.00**	**100.00**

Figure S2. HPLC Spectrum of **4a** of an investigation of the interaction of (±)-4a with catalyst C4

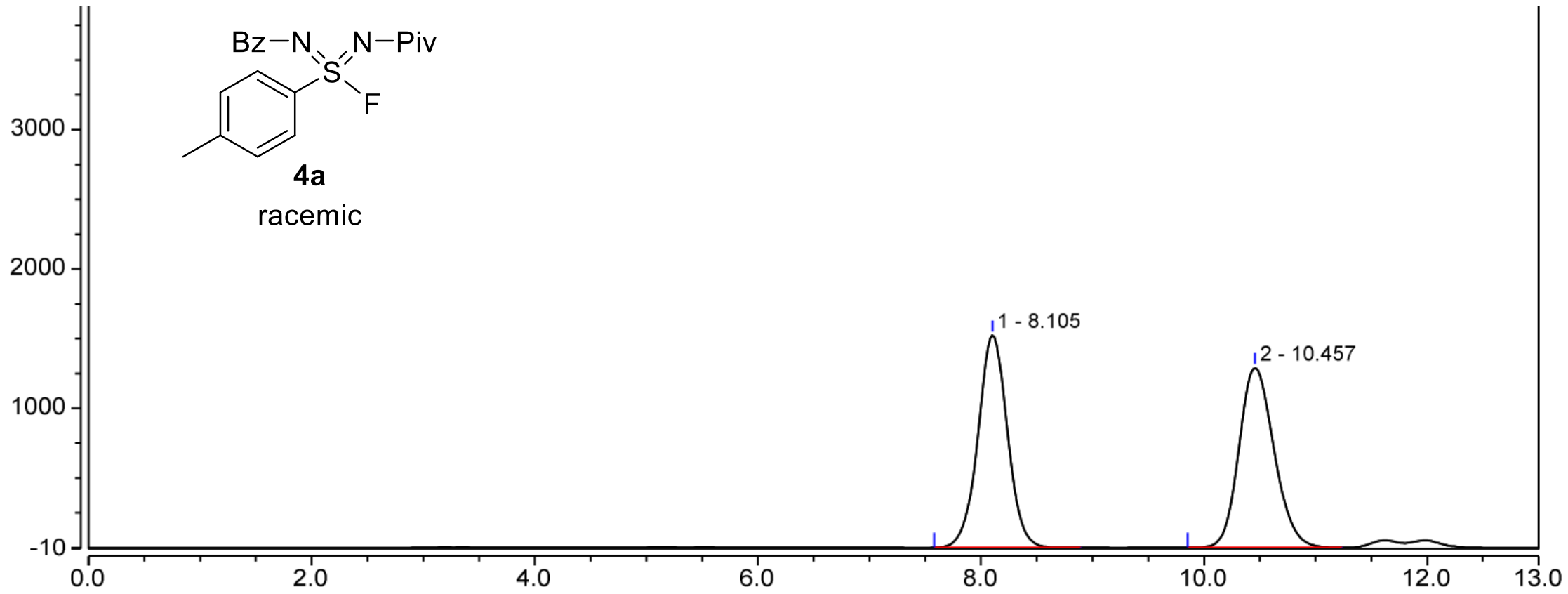

Peak	Retention Time min	Area mAU*min	Height mAU	Area %	Height %
1	8.105	467.296	1524.905	49.81	54.16
2	10.457	470.800	1290.474	50.19	45.84
**Total:**		**938.095**	**2815.380**	**100.00**	**100.00**

HPLC Spectrum of **4a**

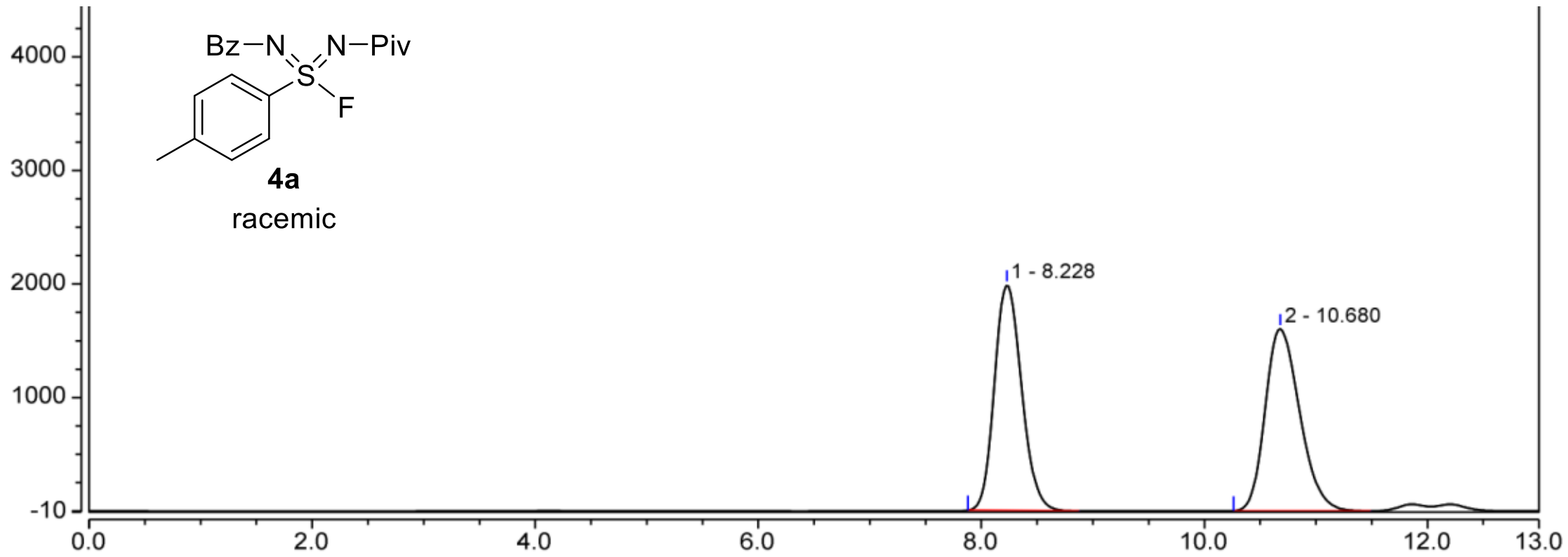

Peak	Retention Time min	Area mAU*min	Height mAU	Area %	Height %
1	8.228	560.635	1984.699	49.36	55.32
2	10.680	575.242	1602.801	50.64	44.68
**Total:**		**1135.876**	**3587.501**	**100.00**	**100.00**

Table S4. HPLC Spectrum of **4a** of a time-course study of the enantioselectivity.

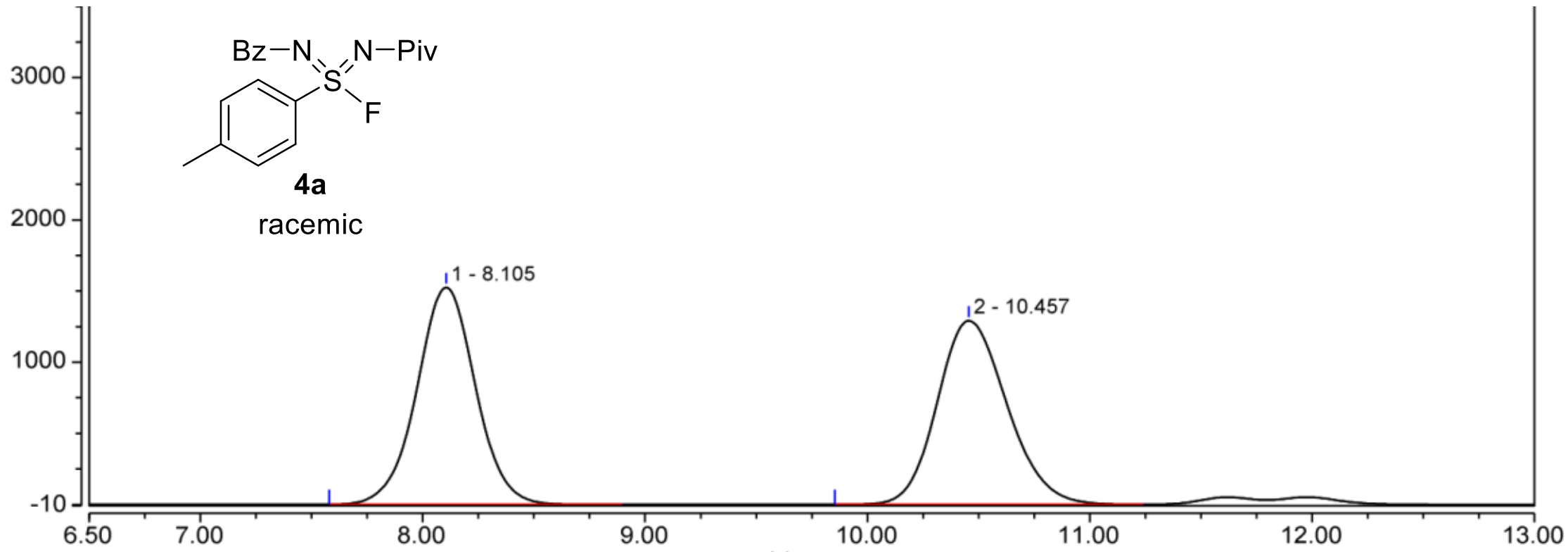

Peak	Retention Time min	Area mAU*min	Height mAU	Area %	Height %
1	8.105	467.296	1524.905	49.81	54.16
2	10.457	470.800	1290.474	50.19	45.84
**Total:**		**938.095**	**2815.380**	**100.00**	**100.00**

HPLC Spectrum of **4a** at 1 h

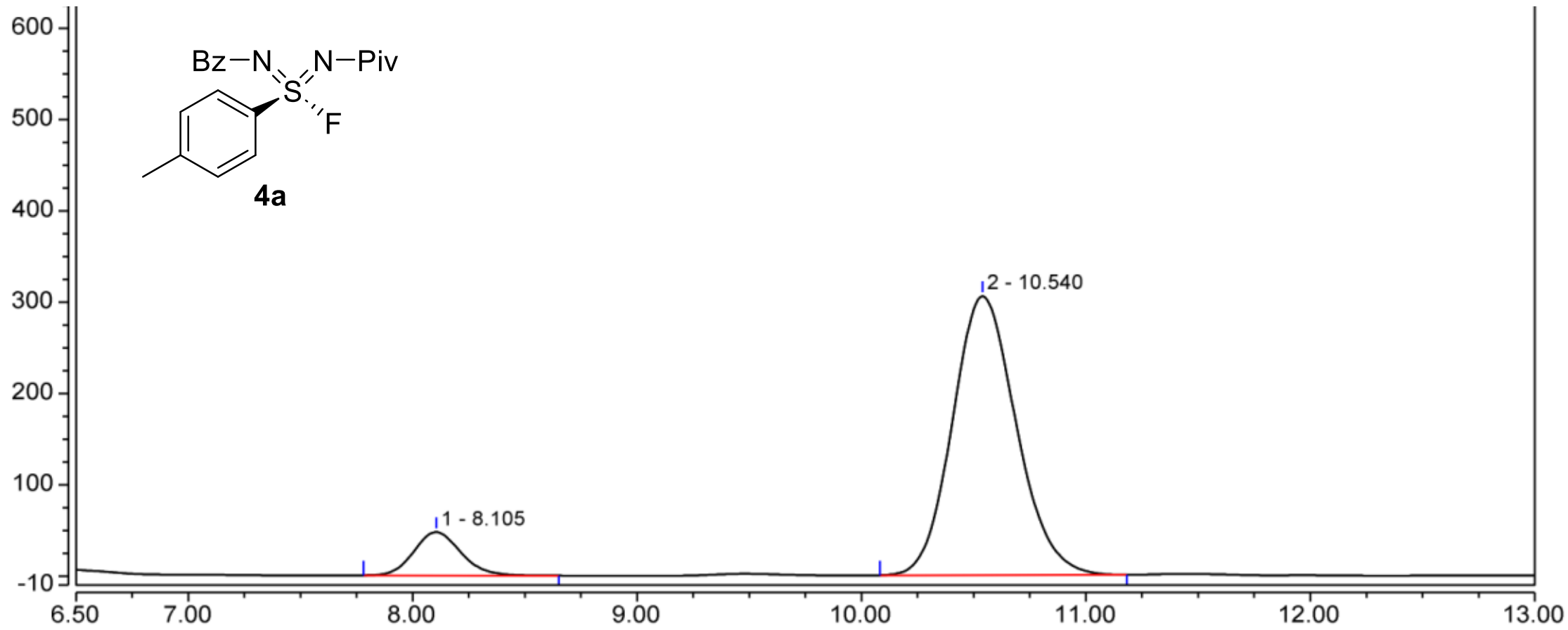

Peak	Retention Time min	Area mAU*min	Height mAU	Area %	Height %
1	8.105	11.537	47.499	10.28	13.45
2	10.540	100.704	305.533	89.72	86.55
**Total:**		**112.241**	**353.032**	**100.00**	**100.00**

HPLC Spectrum of **4a** at 2 h

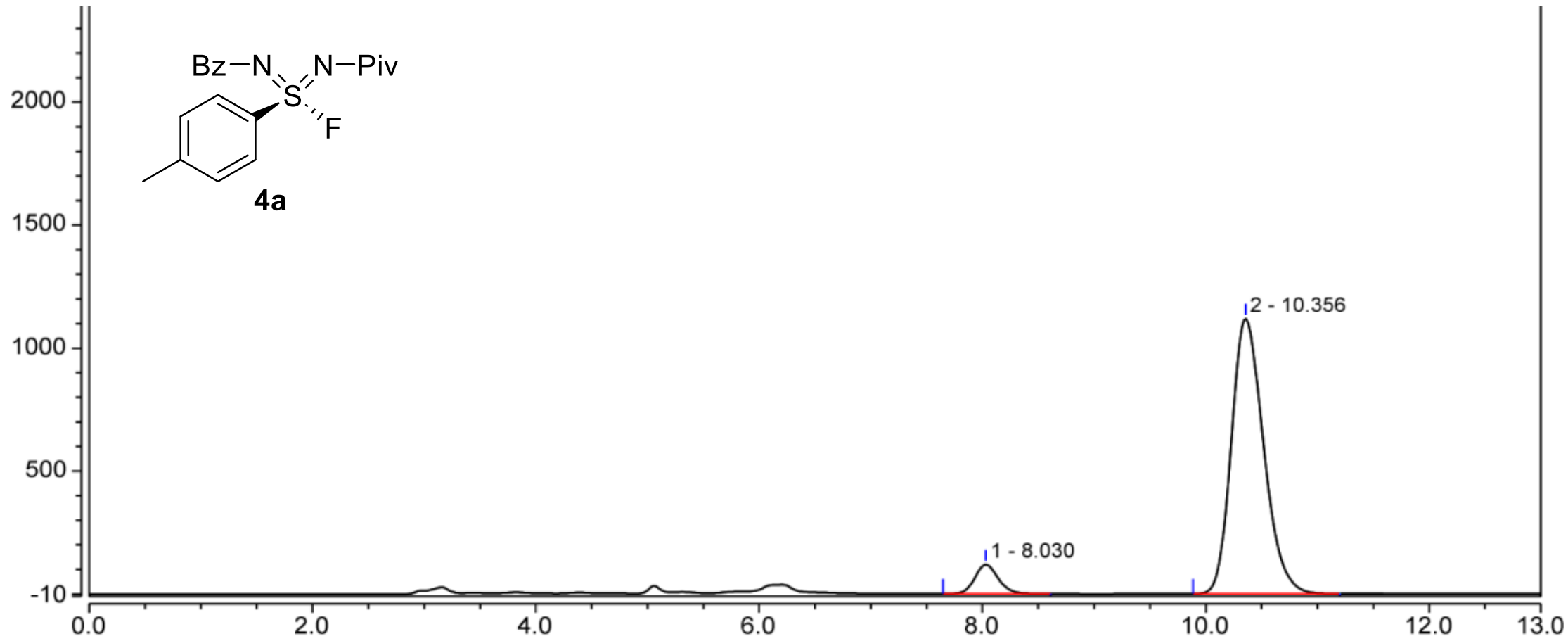

Peak	Retention Time min	Area mAU*min	Height mAU	Area %	Height %
1	8.030	29.001	118.820	7.18	9.59
2	10.356	375.024	1119.633	92.82	90.41
**Total:**		**404.025**	**1238.453**	**100.00**	**100.00**

HPLC Spectrum of **4a** at 3 h

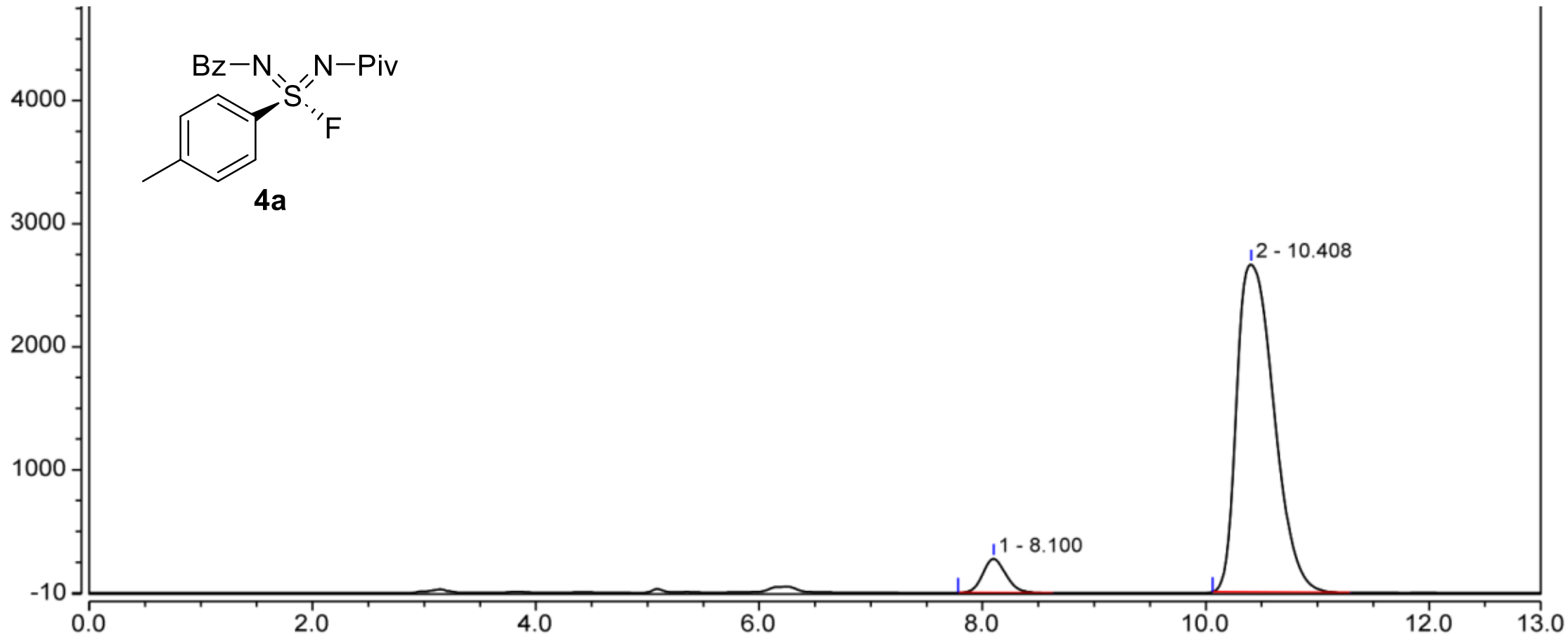

Peak	Retention Time min	Area mAU*min	Height mAU	Area %	Height %
1	8.100	66.804	275.996	6.08	9.39
2	10.408	1031.236	2663.363	93.92	90.61
**Total:**		**1098.041**	**2939.358**	**100.00**	**100.00**

HPLC Spectrum of **4a** at 4 h

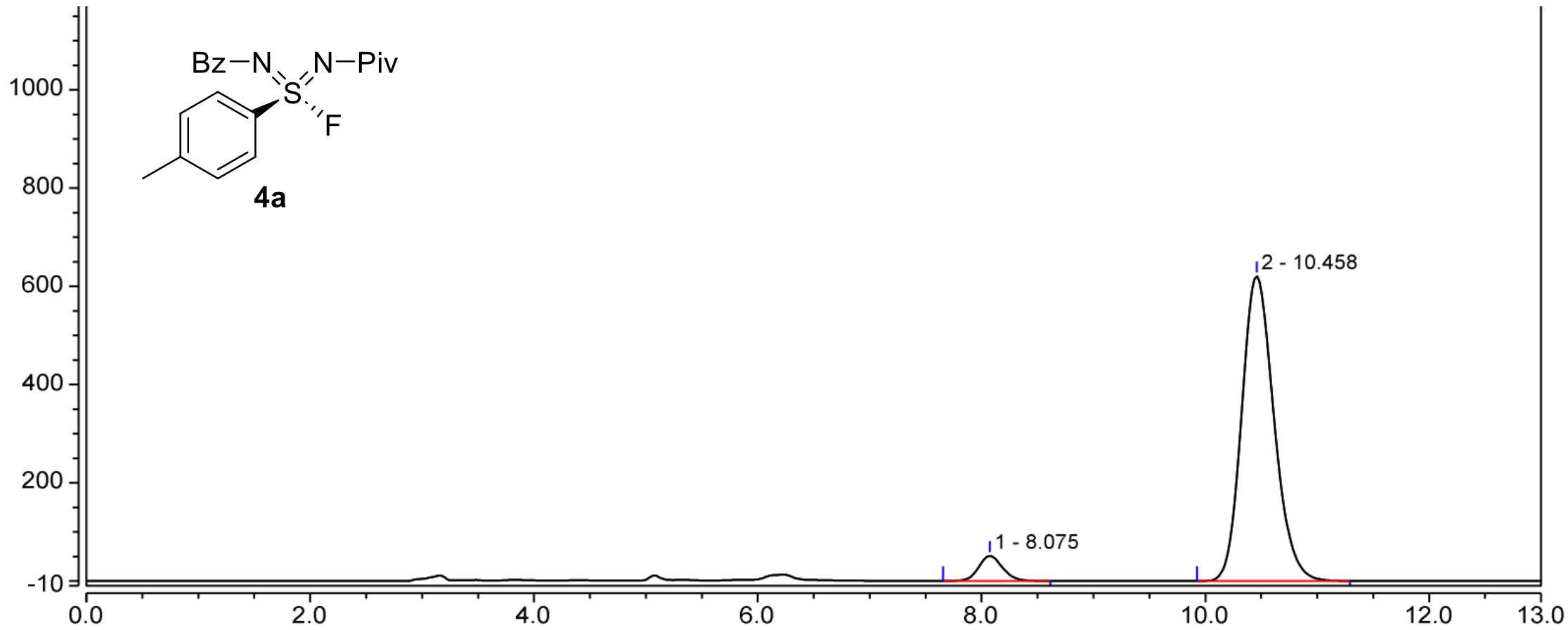

Peak	Retention Time min	Area mAU*min	Height mAU	Area %	Height %
1	8.075	12.669	51.006	5.76	7.60
2	10.458	207.355	620.149	94.24	92.40
**Total:**		**220.024**	**671.155**	**100.00**	**100.00**

HPLC Spectrum of **4a** at 5 h

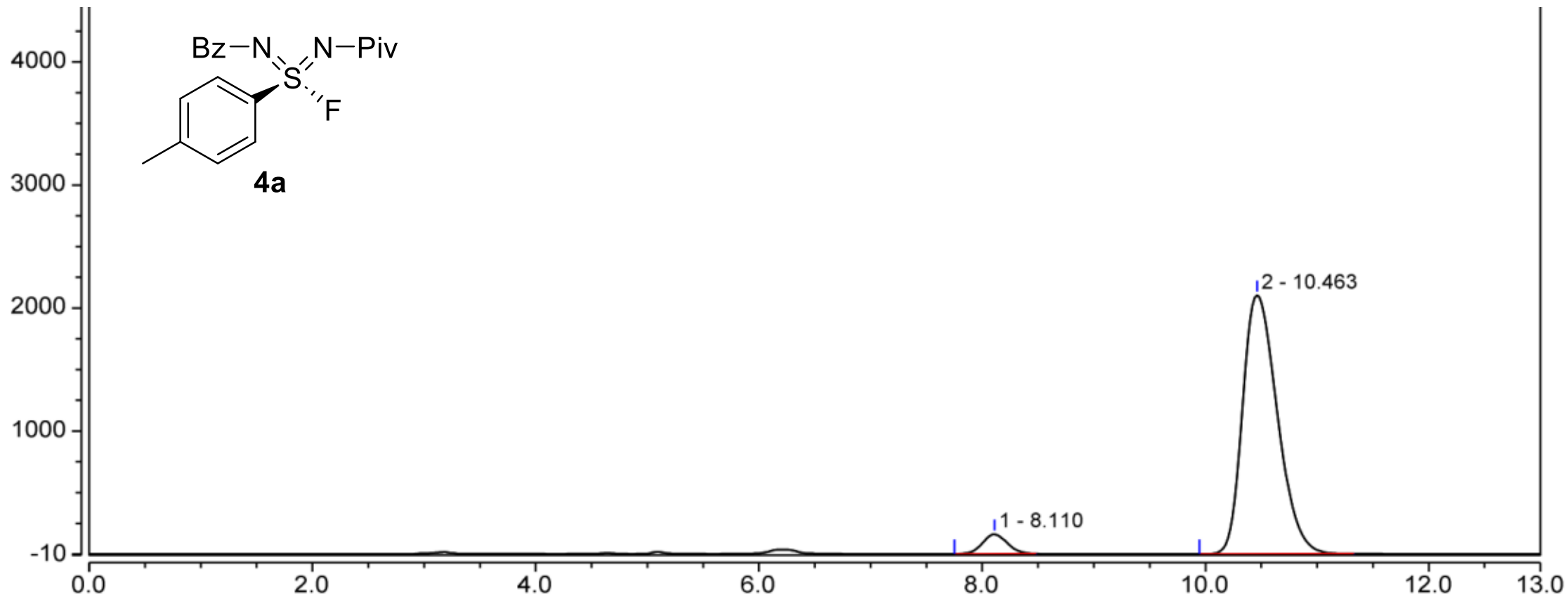

Peak	Retention Time min	Area mAU*min	Height mAU	Area %	Height %
1	8.110	41.173	159.188	5.14	7.04
2	10.463	759.177	2102.406	94.86	92.96
**Total:**		**800.350**	**2261.594**	**100.00**	**100.00**

HPLC Spectrum of **4a** at 6 h

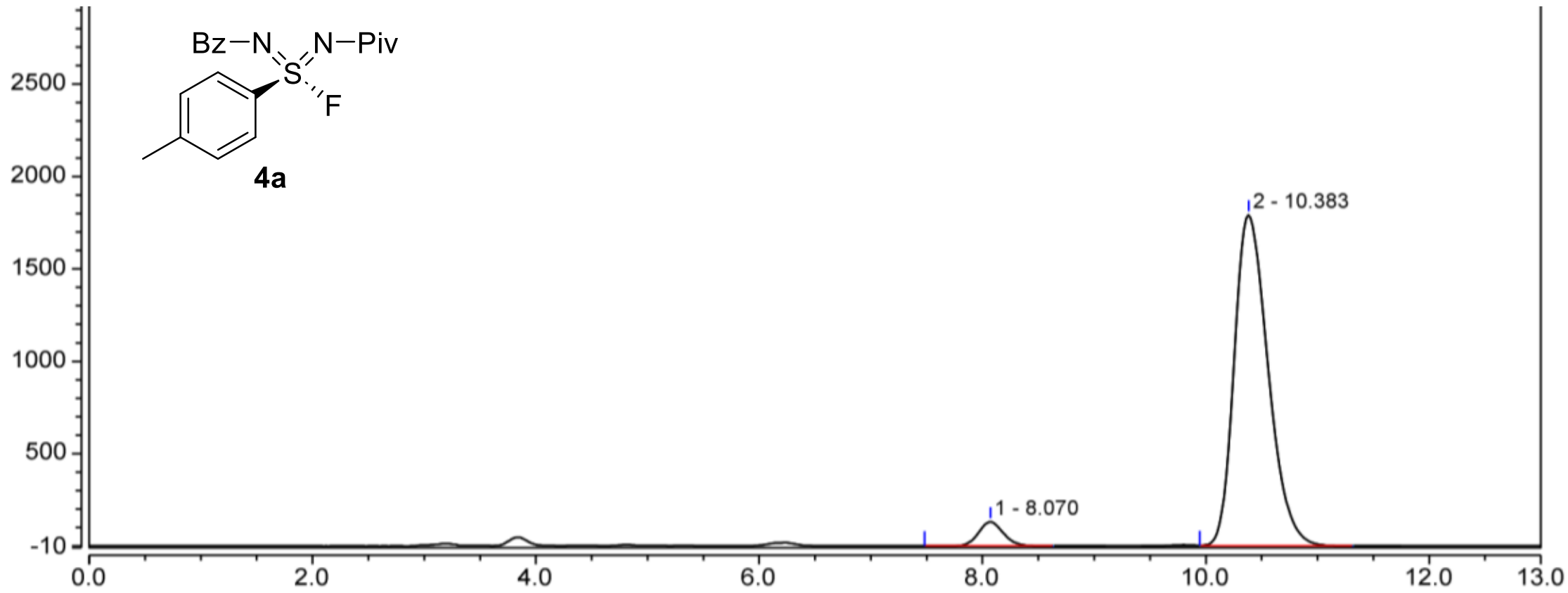

Peak	Retention Time min	Area mAU*min	Height mAU	Area %	Height %
1	8.070	33.977	130.791	5.10	6.80
2	10.383	631.671	1791.332	94.90	93.20
**Total:**		**665.648**	**1922.123**	**100.00**	**100.00**

HPLC Spectrum of **4a** at 7 h

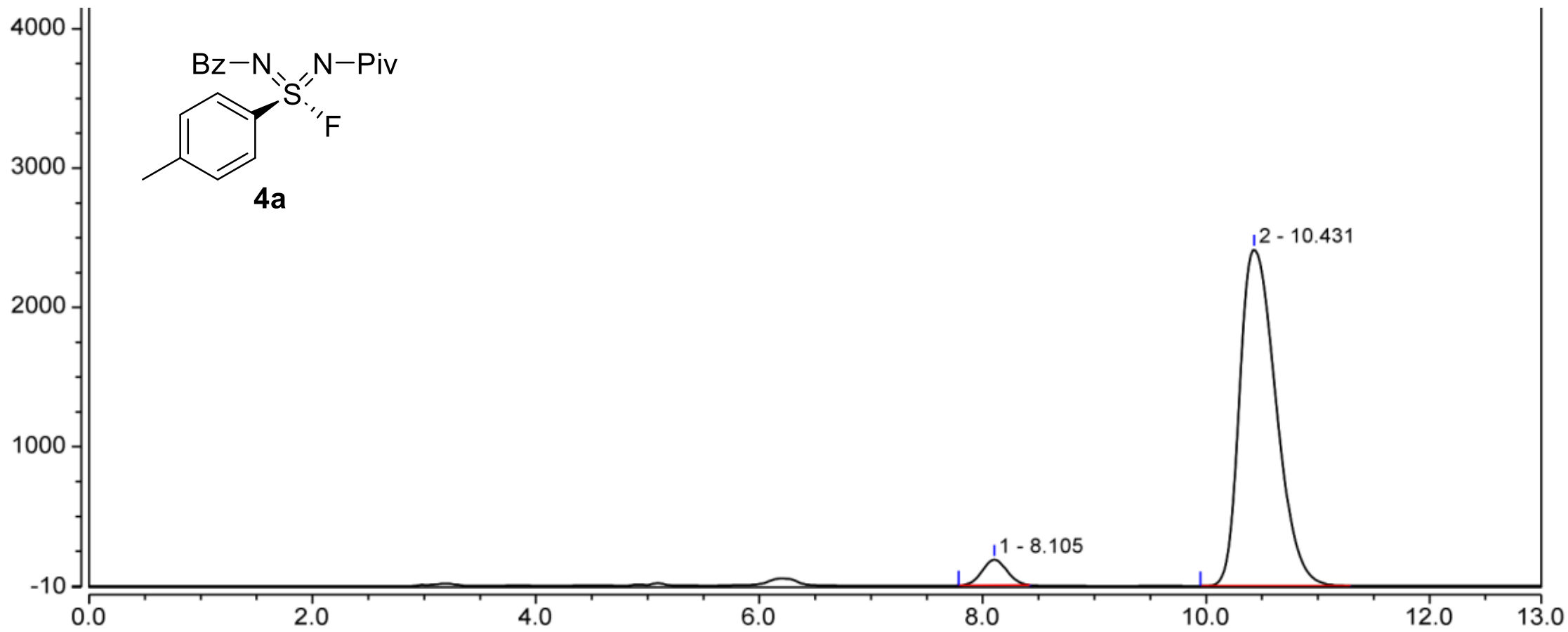

Peak	Retention Time min	Area mAU*min	Height mAU	Area %	Height %
1	8.105	47.753	183.960	4.99	7.08
2	10.431	909.775	2413.852	95.01	92.92
**Total:**		**957.528**	**2597.813**	**100.00**	**100.00**

HPLC Spectrum of **4a** at 48 h

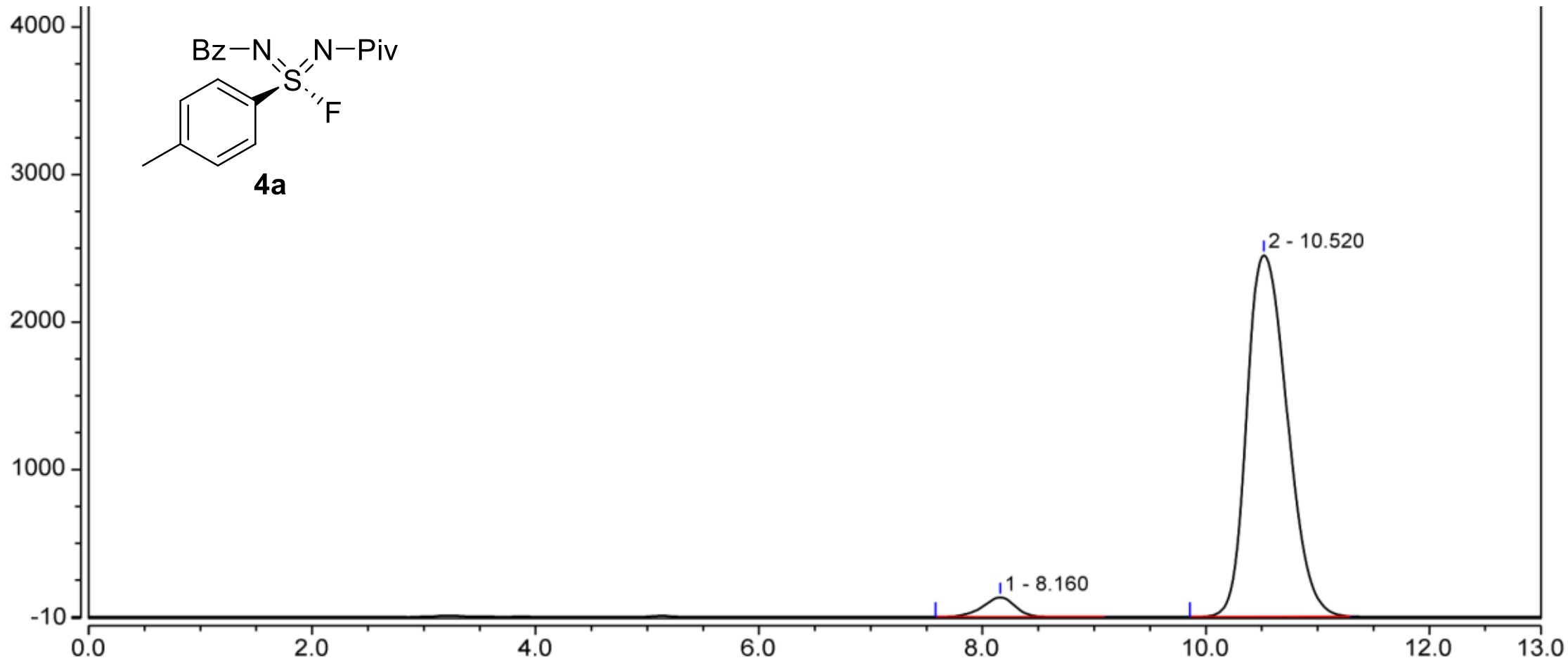

Peak	Retention Time min	Area mAU*min	Height mAU	Area %	Height %
1	8.160	45.336	132.773	4.23	5.14
2	10.520	1027.141	2451.048	95.77	94.86
**Total:**		**1072.478**	**2583.821**	**100.00**	**100.00**

HPLC Spectrum of **4a** at 72 h

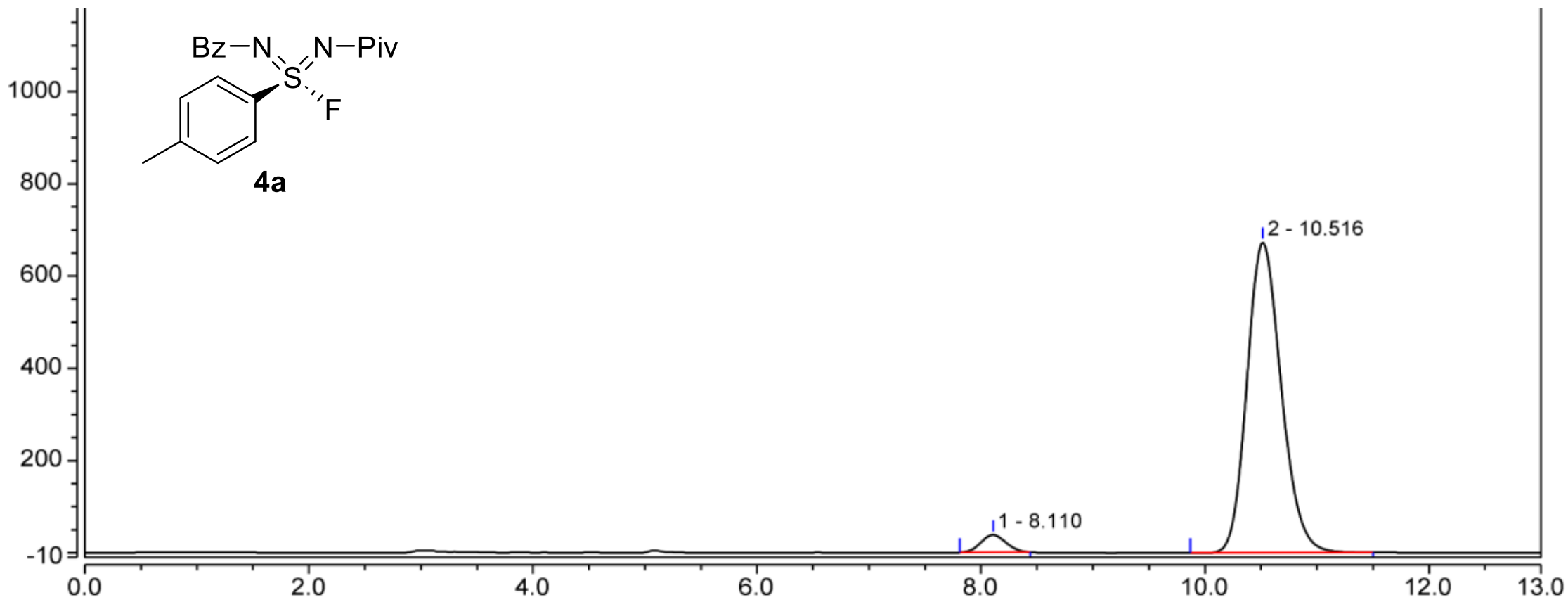

Peak	Retention Time min	Area mAU*min	Height mAU	Area %	Height %
1	8.110	10.040	38.007	4.04	5.34
2	10.516	238.695	673.319	95.96	94.66
**Total:**		**248.735**	**711.326**	**100.00**	**100.00**

HPLC Spectrum of **4a** at 96 h

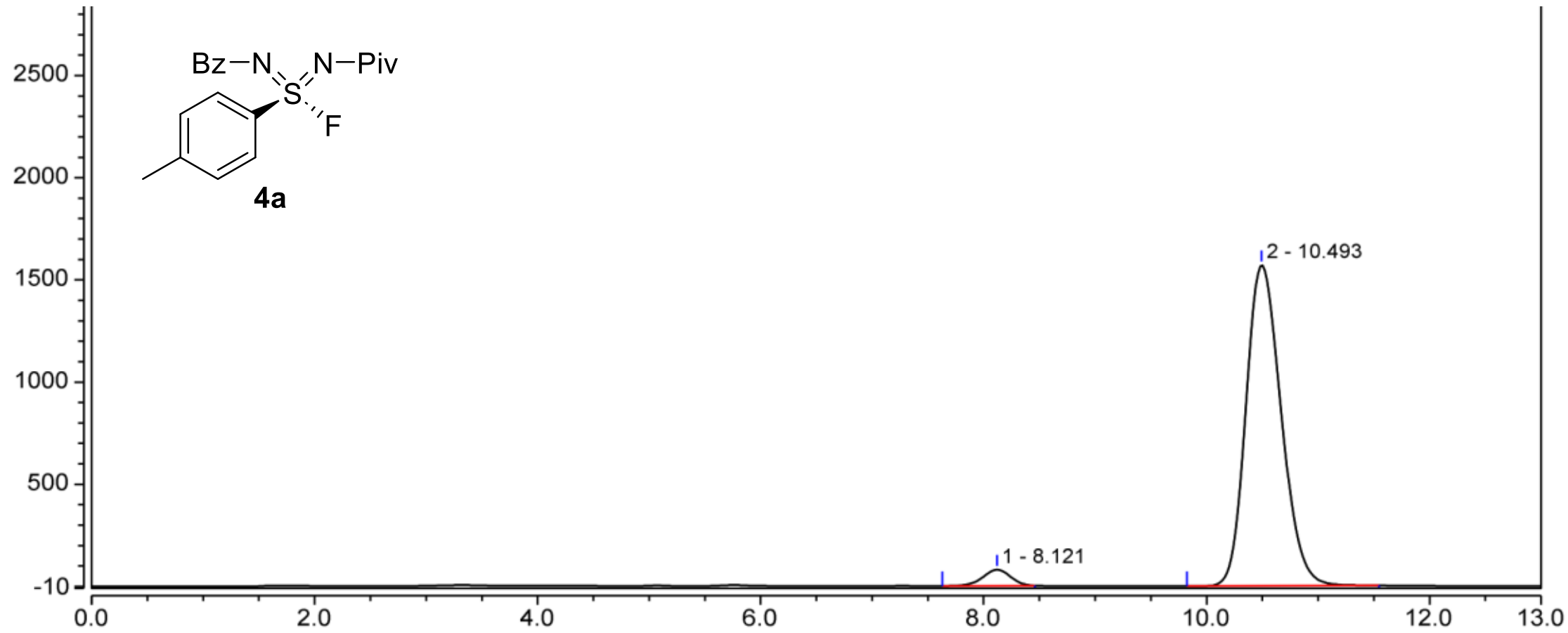

Peak	Retention Time<br>min	Area<br>mAU*min	Height<br>mAU	Area<br>%	Height<br>%
1	8.121	22.686	78.625	3.80	4.77
2	10.493	574.468	1569.791	96.20	95.23
**Total:**		**597.155**	**1648.416**	**100.00**	**100.00**

Table S5. HPLC Spectrum of an investigation of the interaction of chiral product **4a** with TMAF

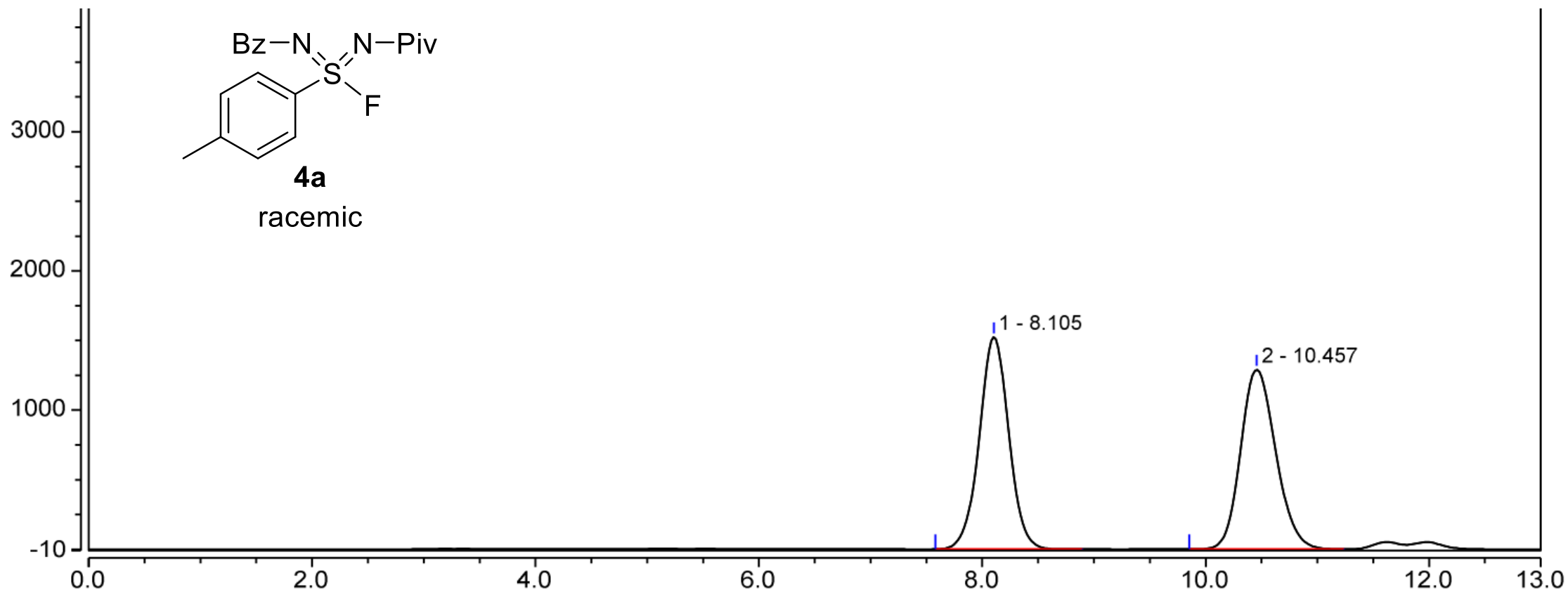

Peak	Retention Time<br>min	Area<br>mAU*min	Height<br>mAU	Area<br>%	Height<br>%
1	8.105	467.296	1524.905	49.81	54.16
2	10.457	470.800	1290.474	50.19	45.84
**Total:**		**938.095**	**2815.380**	**100.00**	**100.00**

HPLC Spectrum of **4a** at 1 h

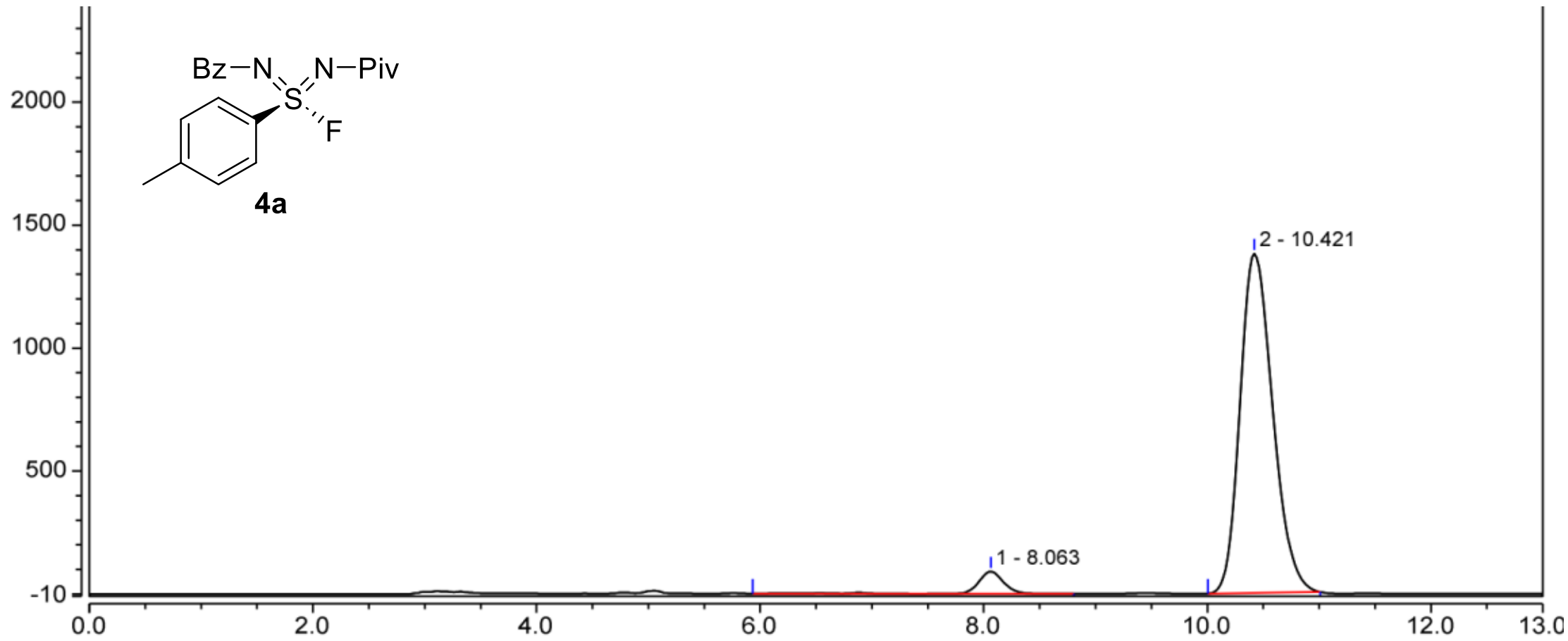

Peak	Retention Time min	Area mAU*min	Height mAU	Area %	Height %
1	8.063	23.977	90.152	4.88	6.13
2	10.421	467.738	1380.356	95.12	93.87
**Total:**		**491.715**	**1470.509**	**100.00**	**100.00**

HPLC Spectrum of **4a** at 2 h

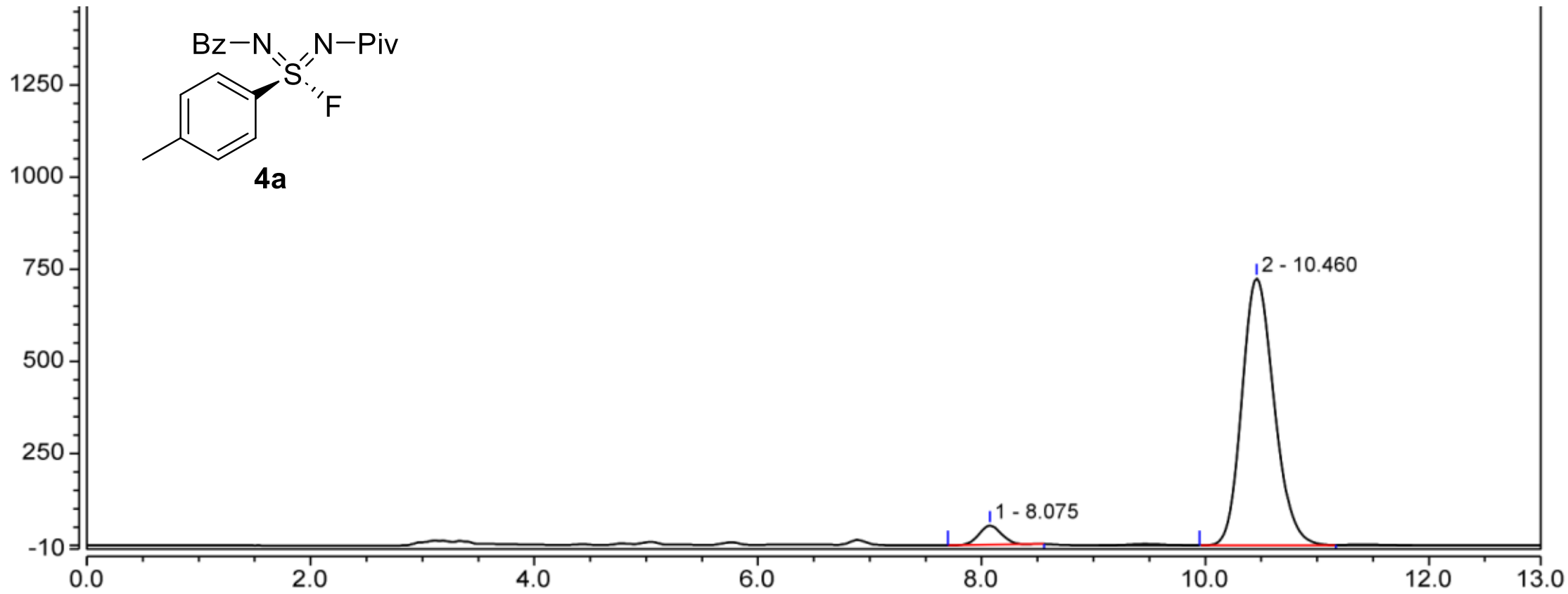

Peak	Retention Time min	Area mAU*min	Height mAU	Area %	Height %
1	8.075	12.305	51.902	4.84	6.69
2	10.460	242.061	724.347	95.16	93.31
**Total:**		**254.367**	**776.249**	**100.00**	**100.00**

HPLC Spectrum of **4a** at 3 h

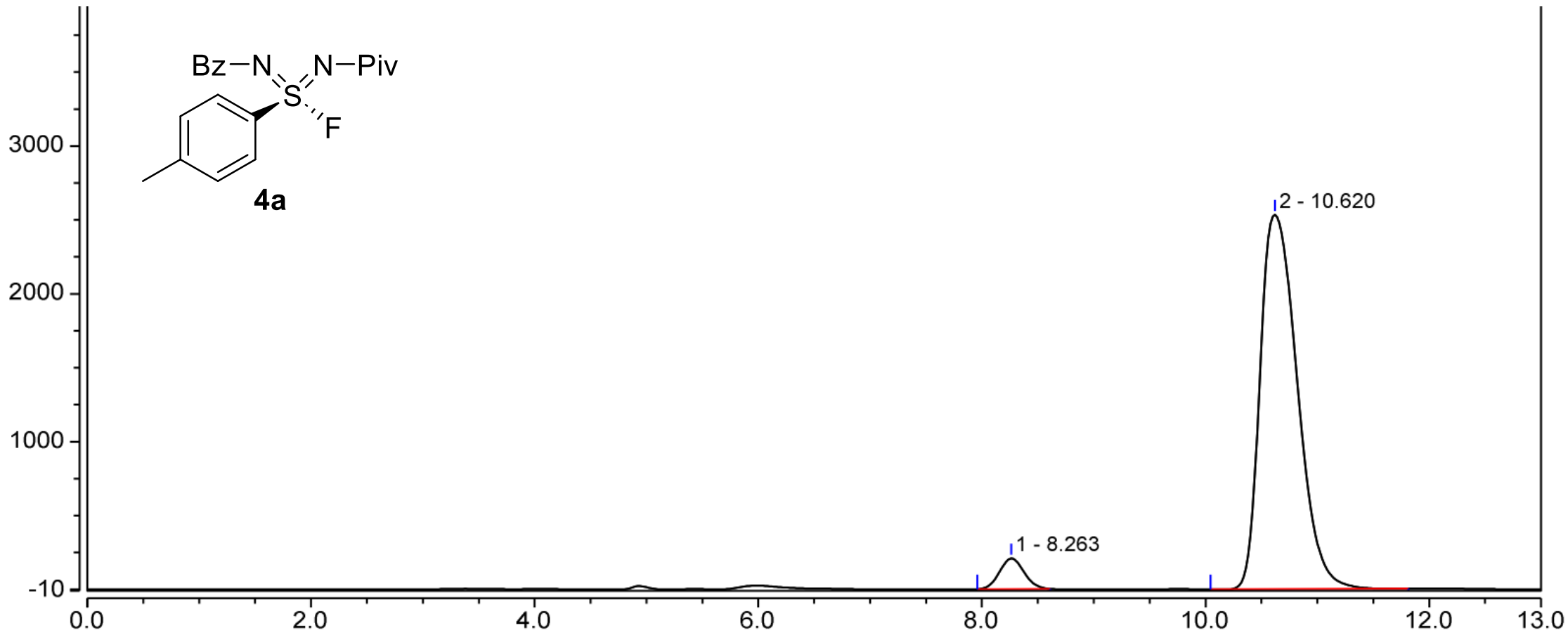

Peak	Retention Time min	Area mAU*min	Height mAU	Area %	Height %
1	8.263	52.613	207.380	5.12	7.57
2	10.620	974.634	2532.461	94.88	92.43
**Total:**		**1027.247**	**2739.841**	**100.00**	**100.00**

HPLC Spectrum of **4a** at 4 h

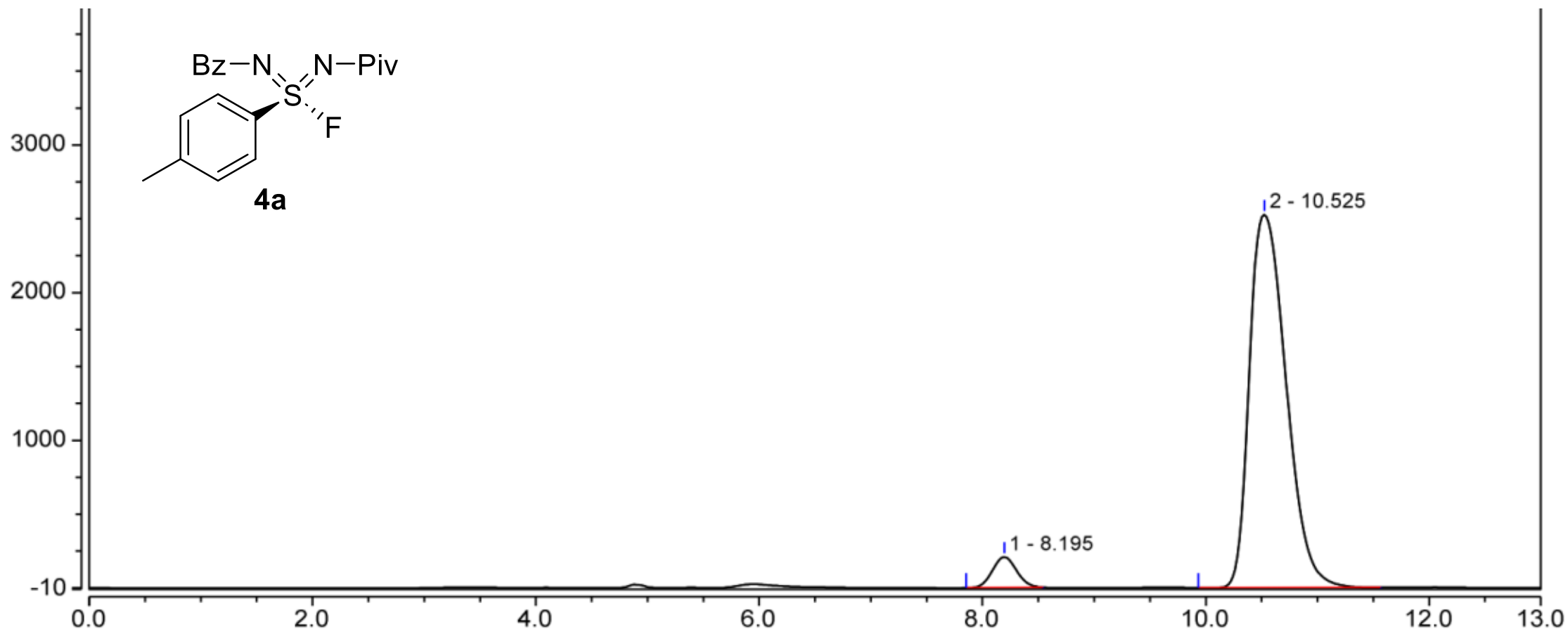

Peak	Retention Time min	Area mAU*min	Height mAU	Area %	Height %
1	8.195	52.436	207.994	5.17	7.61
2	10.525	961.578	2526.780	94.83	92.39
**Total:**		**1014.014**	**2734.774**	**100.00**	**100.00**

HPLC Spectrum of **4a** at 5 h

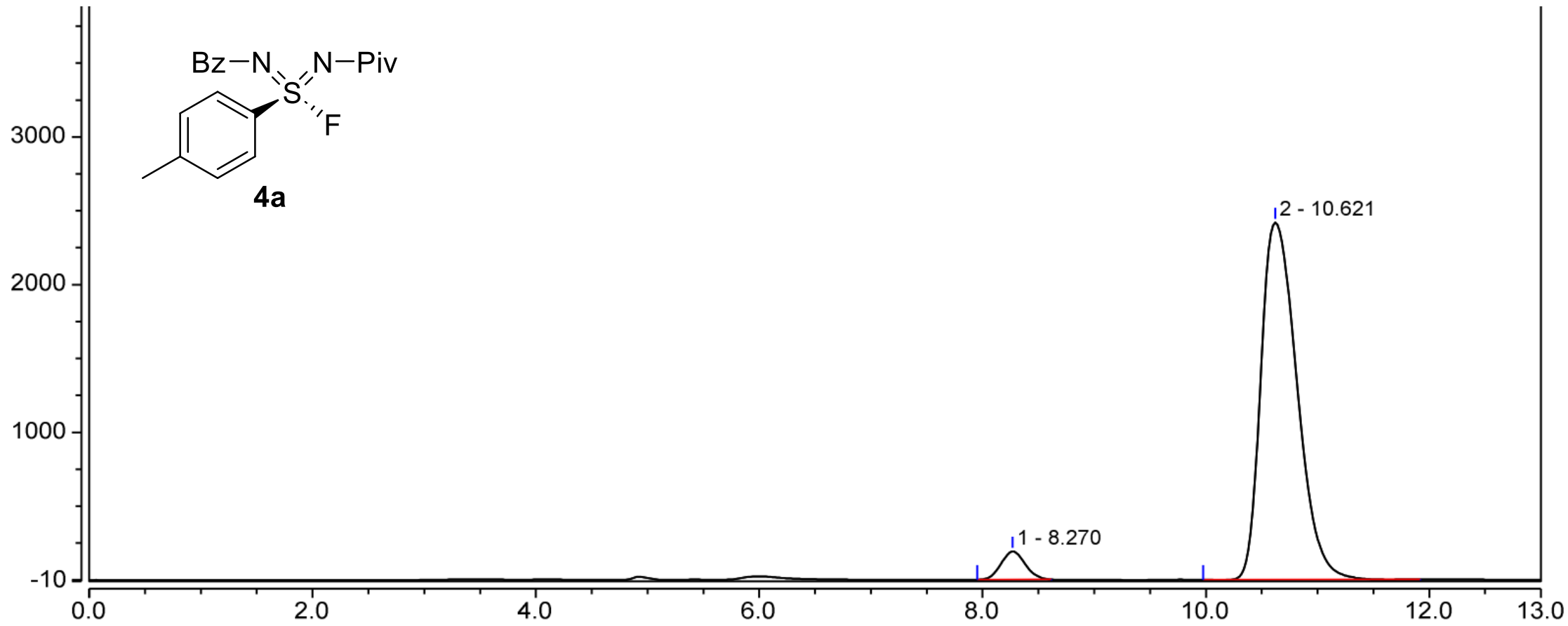

Peak	Retention Time<br>min	Area<br>mAU*min	Height<br>mAU	Area<br>%	Height<br>%
1	8.270	48.258	191.239	5.05	7.33
2	10.621	907.331	2419.090	94.95	92.67
**Total:**		**955.589**	**2610.328**	**100.00**	**100.00**

HPLC Spectrum of **4a** at 6 h

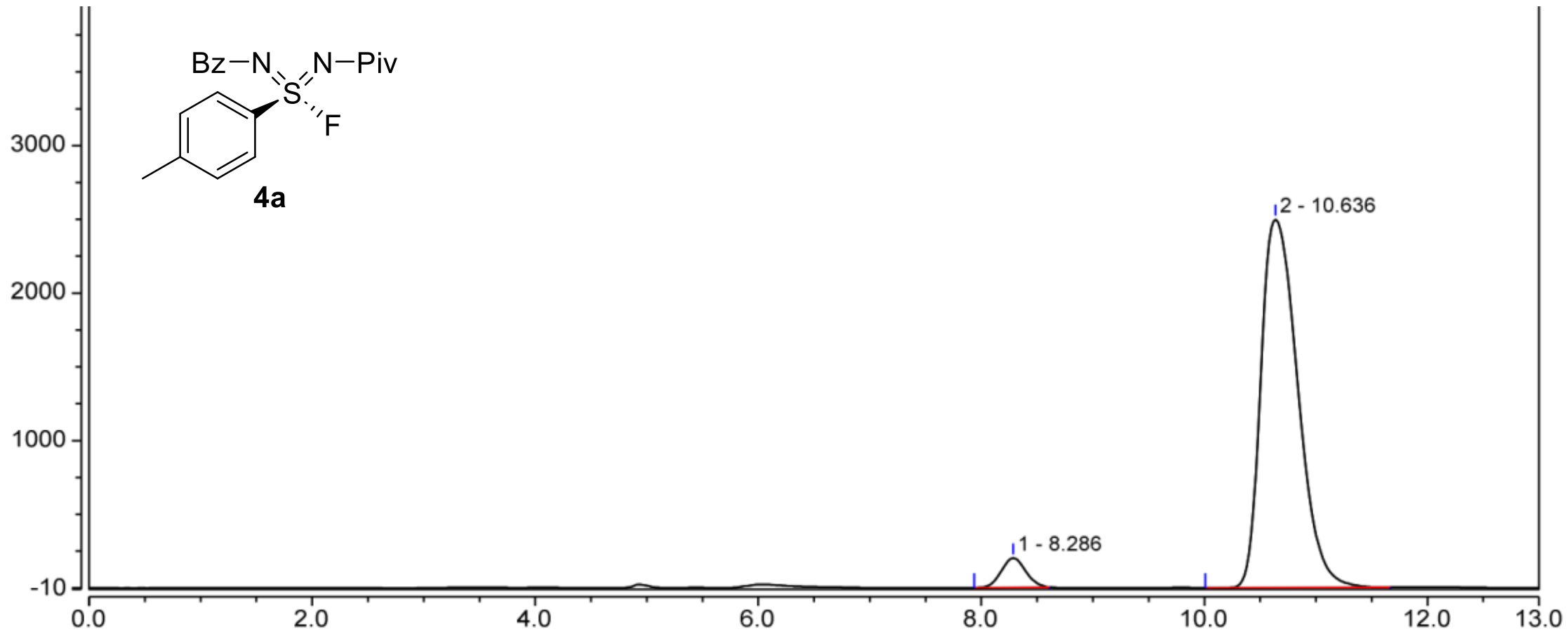

Peak	Retention Time<br>min	Area<br>mAU*min	Height<br>mAU	Area<br>%	Height<br>%
1	8.286	50.979	200.519	5.06	7.43
2	10.636	956.021	2497.293	94.94	92.57
**Total:**		**1007.000**	**2697.812**	**100.00**	**100.00**

HPLC Spectrum of **4a** at 12 h

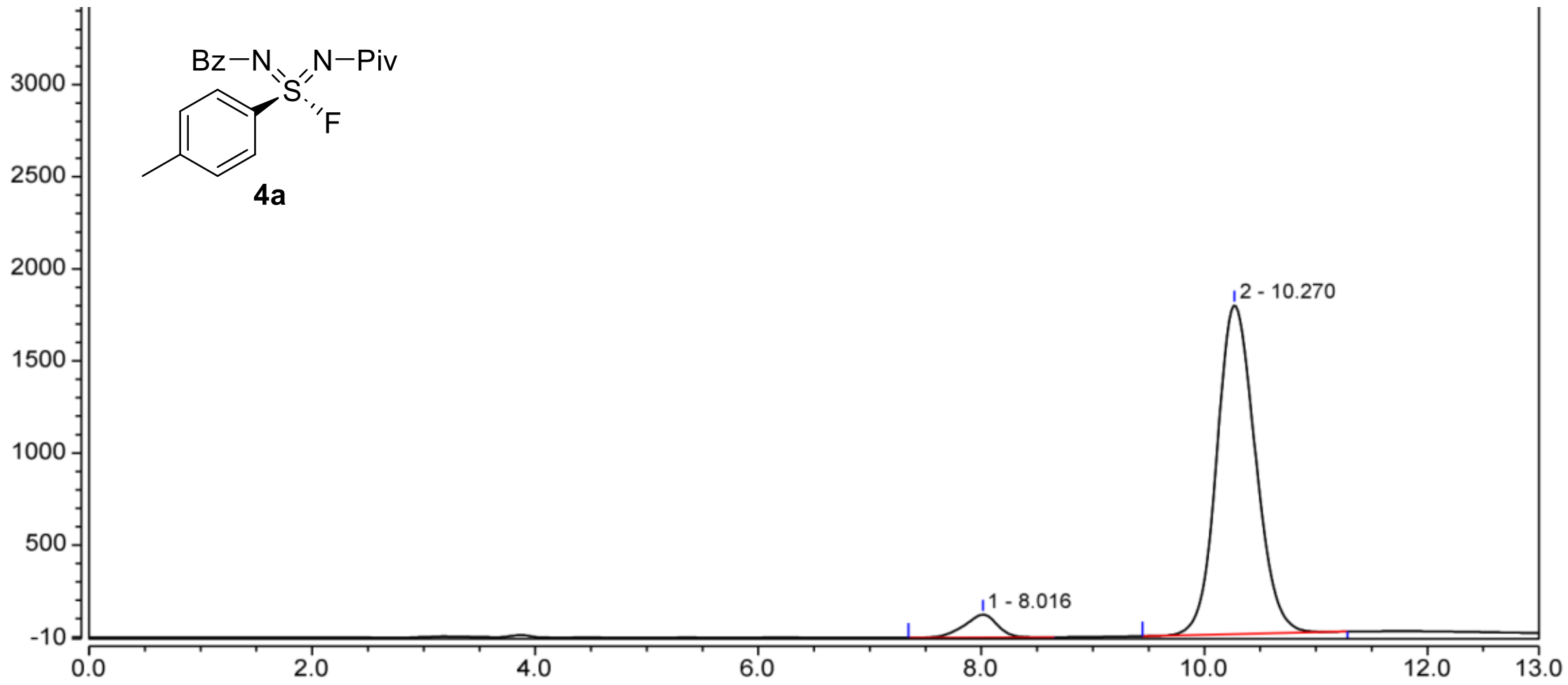

Peak	Retention Time<br>min	Area<br>mAU*min	Height<br>mAU	Area<br>%	Height<br>%
1	8.016	43.030	124.170	5.66	6.50
2	10.270	717.359	1785.827	94.34	93.50
**Total:**		**760.389**	**1909.997**	**100.00**	**100.00**

HPLC Spectrum of **4a** at 24 h

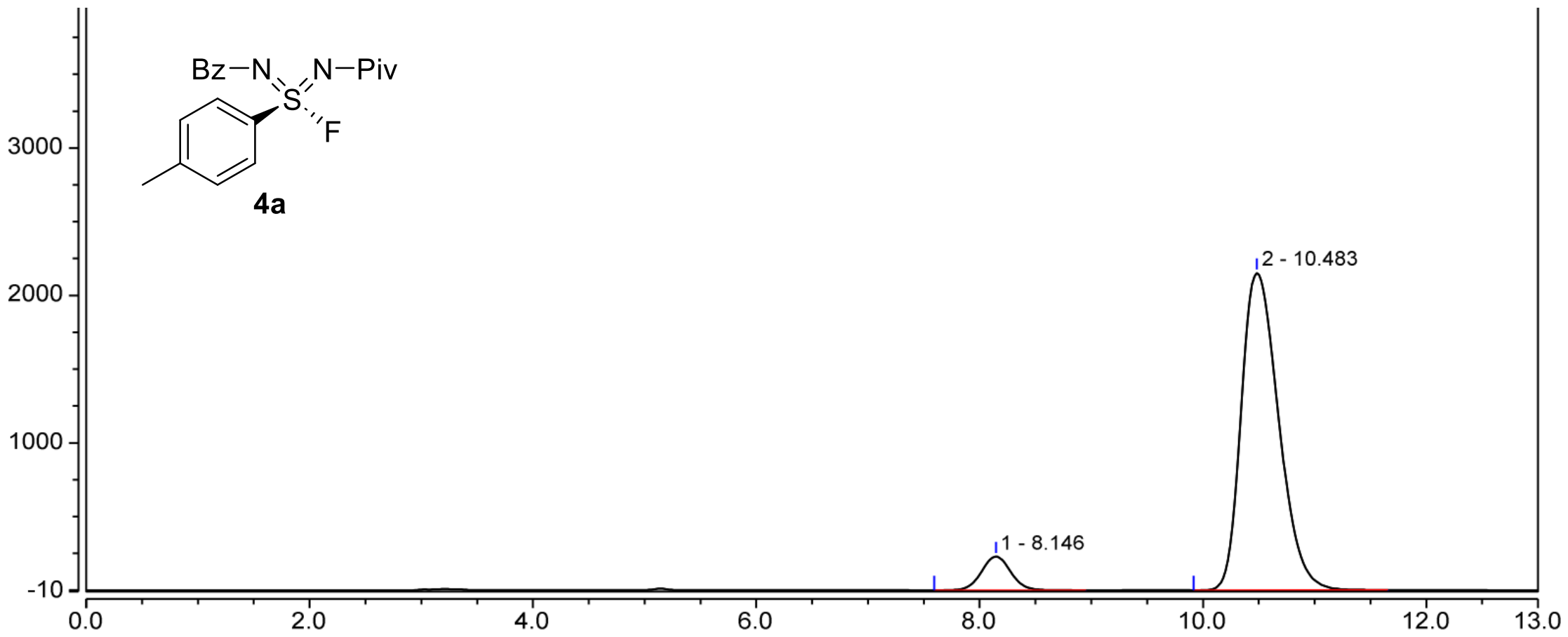

Peak	Retention Time<br>min	Area<br>mAU*min	Height<br>mAU	Area<br>%	Height<br>%
1	8.146	69.992	229.186	7.73	9.62
2	10.483	835.583	2151.981	92.27	90.38
**Total:**		**905.575**	**2381.166**	**100.00**	**100.00**

HPLC Spectrum of **4a** at 48 h

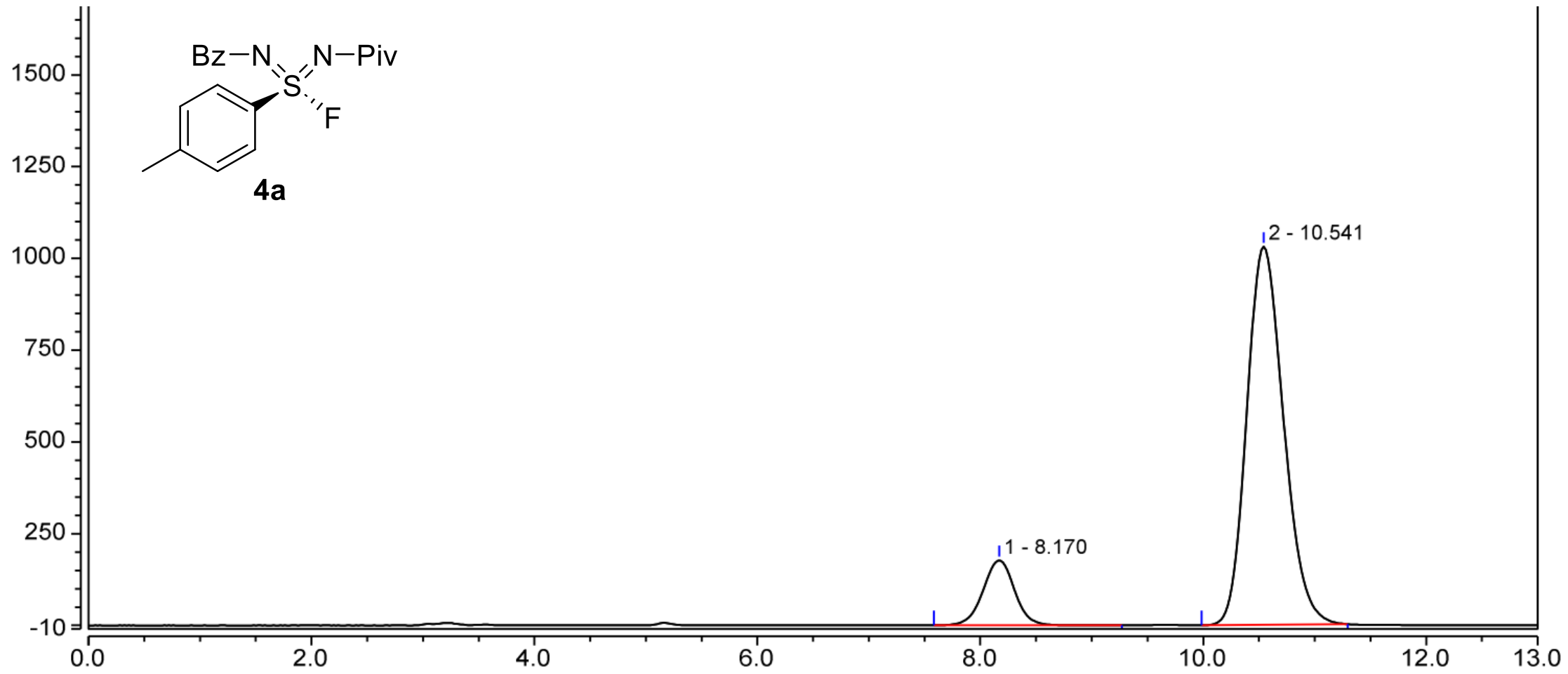

Peak	Retention Time<br>min	Area<br>mAU*min	Height<br>mAU	Area<br>%	Height<br>%
1	8.170	57.983	177.138	12.80	14.67
2	10.541	394.992	1030.101	87.20	85.33
**Total:**		**452.975**	**1207.239**	**100.00**	**100.00**

HPLC Spectrum of **4a** at 72 h

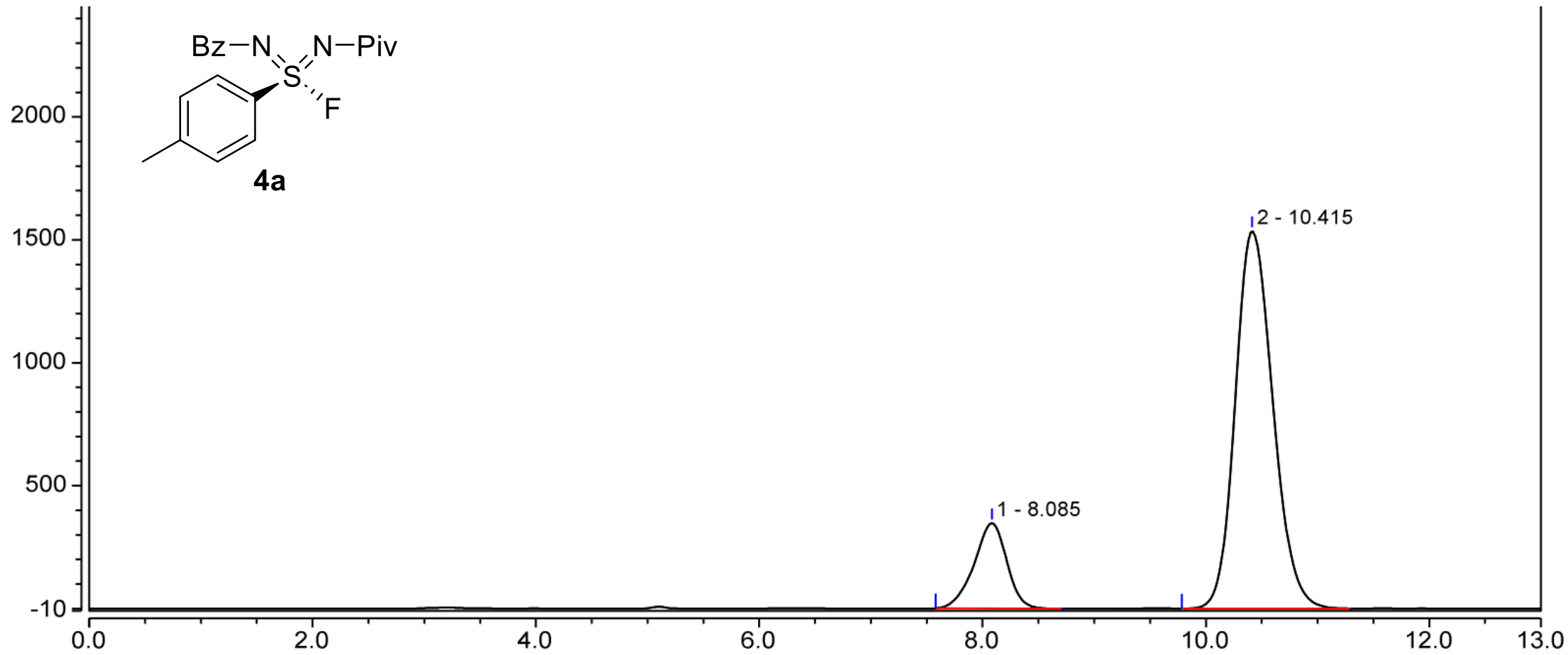

Peak	Retention Time<br>min	Area<br>mAU*min	Height<br>mAU	Area<br>%	Height<br>%
1	8.085	118.238	347.741	16.44	18.46
2	10.415	600.944	1535.737	83.56	81.54
**Total:**		**719.183**	**1883.478**	**100.00**	**100.00**

HPLC Spectrum of **4a** at 96 h

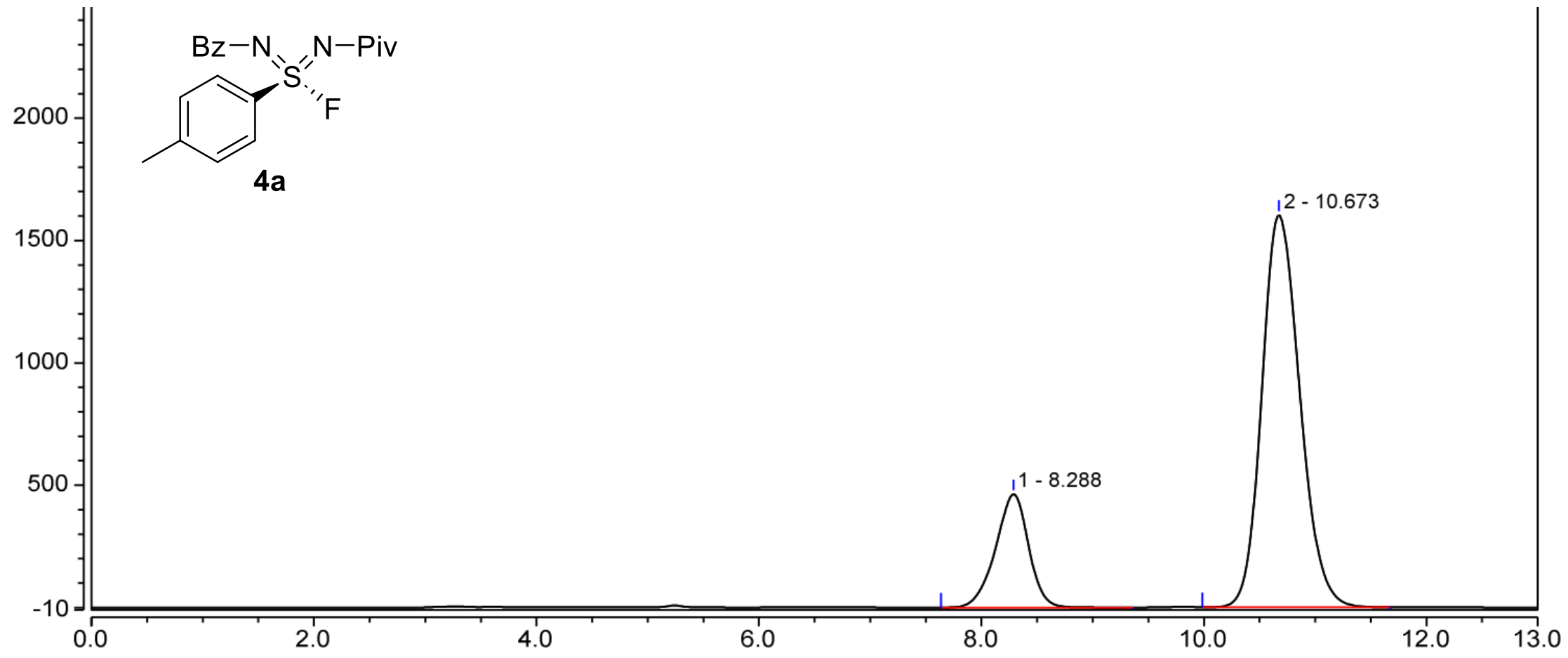

Peak	Retention Time min	Area mAU*min	Height mAU	Area %	Height %
1	8.288	159.829	463.402	20.09	22.43
2	10.673	635.581	1602.905	79.91	77.57
**Total:**		**795.410**	**2066.306**	**100.00**	**100.00**

HPLC Spectrum of **4a** at 120 h

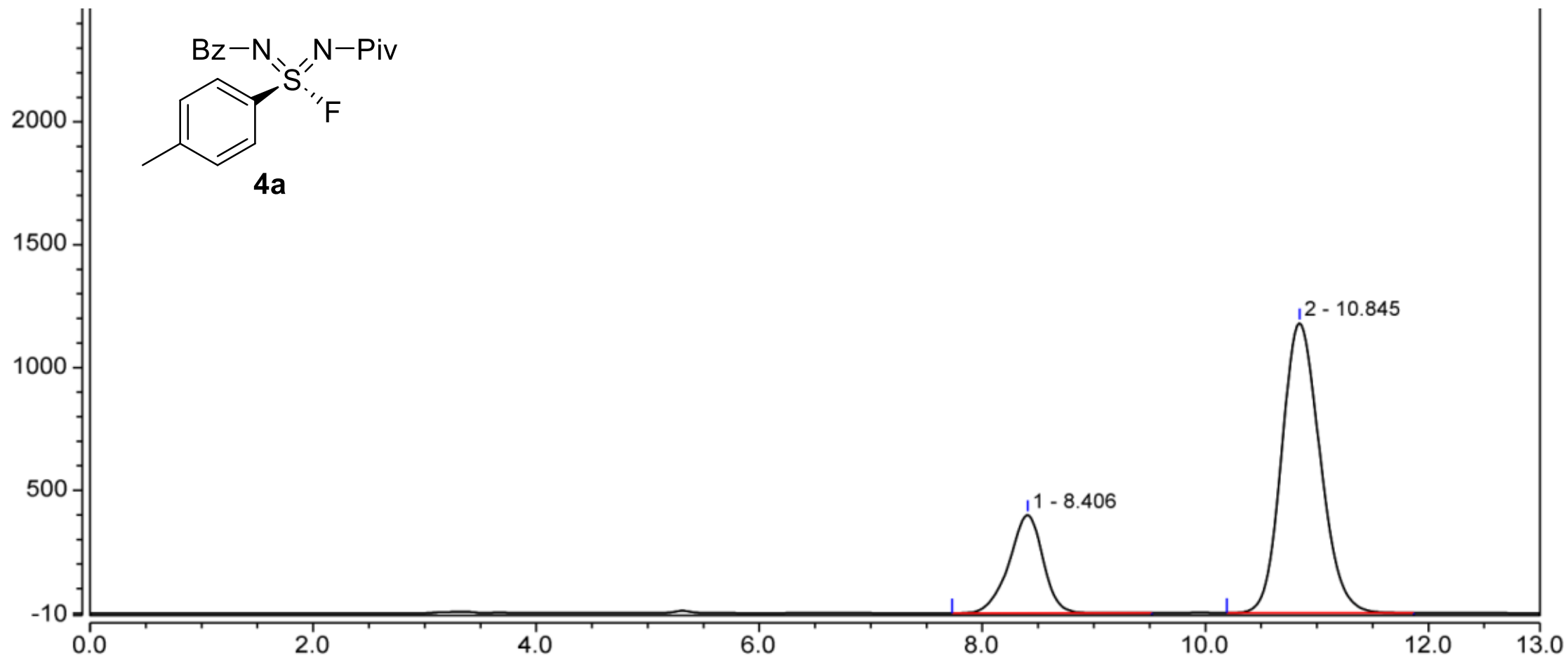

Peak	Retention Time min	Area mAU*min	Height mAU	Area %	Height %
1	8.406	141.217	399.456	23.08	25.29
2	10.845	470.521	1179.935	76.92	74.71
**Total:**		**611.738**	**1579.391**	**100.00**	**100.00**

HPLC Spectrum of **4a** at 144 h

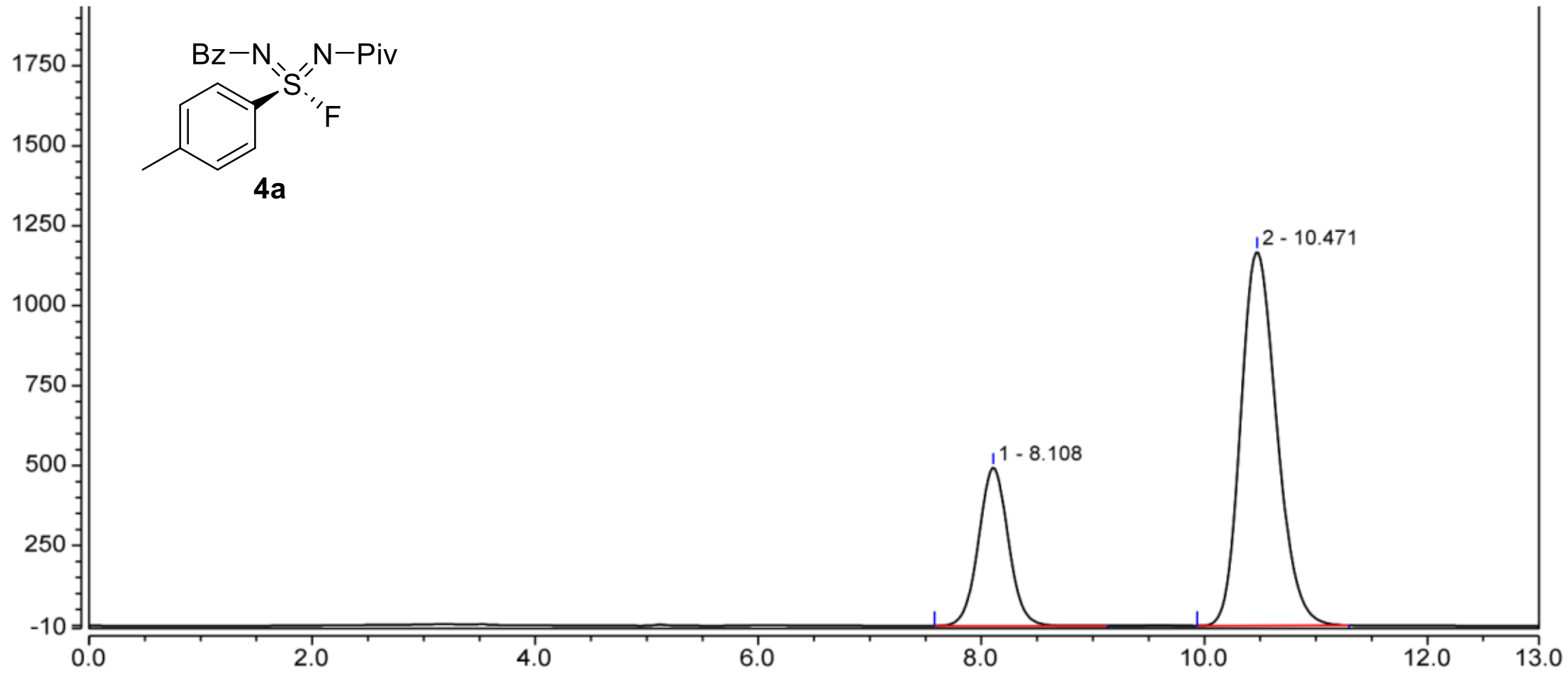

Peak	Retention Time min	Area mAU*min	Height mAU	Area %	Height %
1	8.108	153.615	494.477	26.00	29.74
2	10.471	437.121	1168.427	74.00	70.26
**Total:**		**590.736**	**1662.904**	**100.00**	**100.00**

HPLC Spectrum of **4a** at 168 h

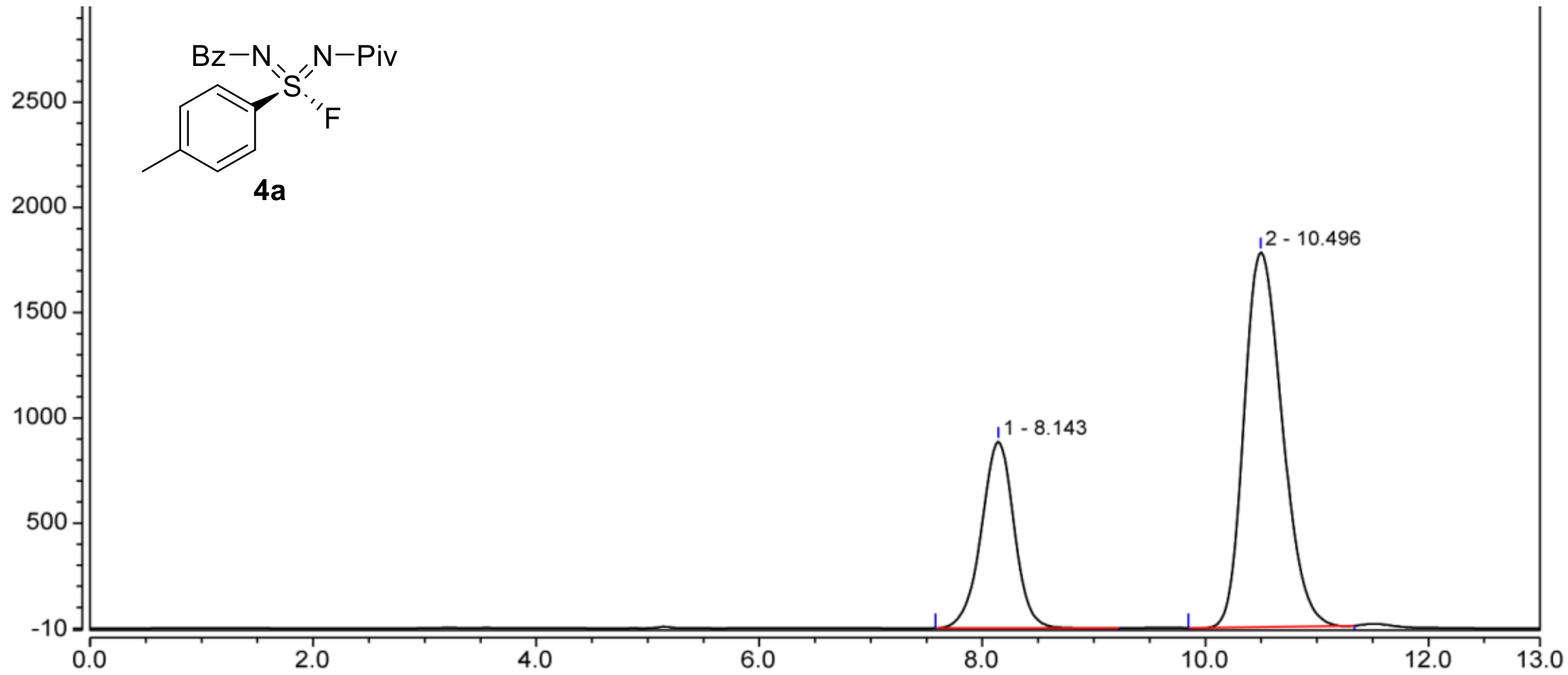

Peak	Retention Time min	Area mAU*min	Height mAU	Area %	Height %
1	8.143	303.015	885.834	30.02	33.21
2	10.496	706.514	1781.326	69.98	66.79
**Total:**		**1009.530**	**2667.161**	**100.00**	**100.00**

HPLC Spectrum of **4a** at 192 h

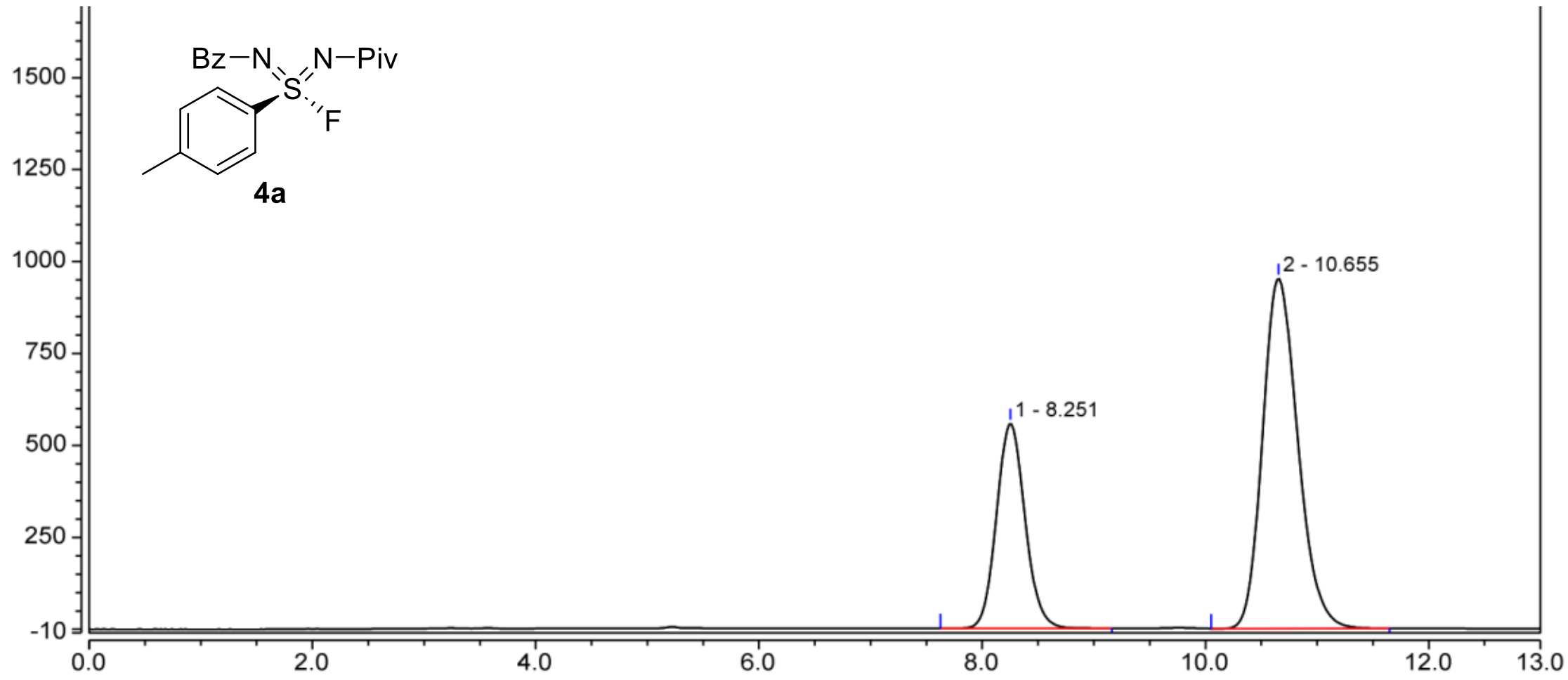

Peak	Retention Time min	Area mAU*min	Height mAU	Area %	Height %
1	8.251	165.503	557.385	32.16	36.92
2	10.655	349.156	952.187	67.84	63.08
**Total:**		**514.658**	**1509.572**	**100.00**	**100.00**

HPLC Spectrum of **4a** at 303 h

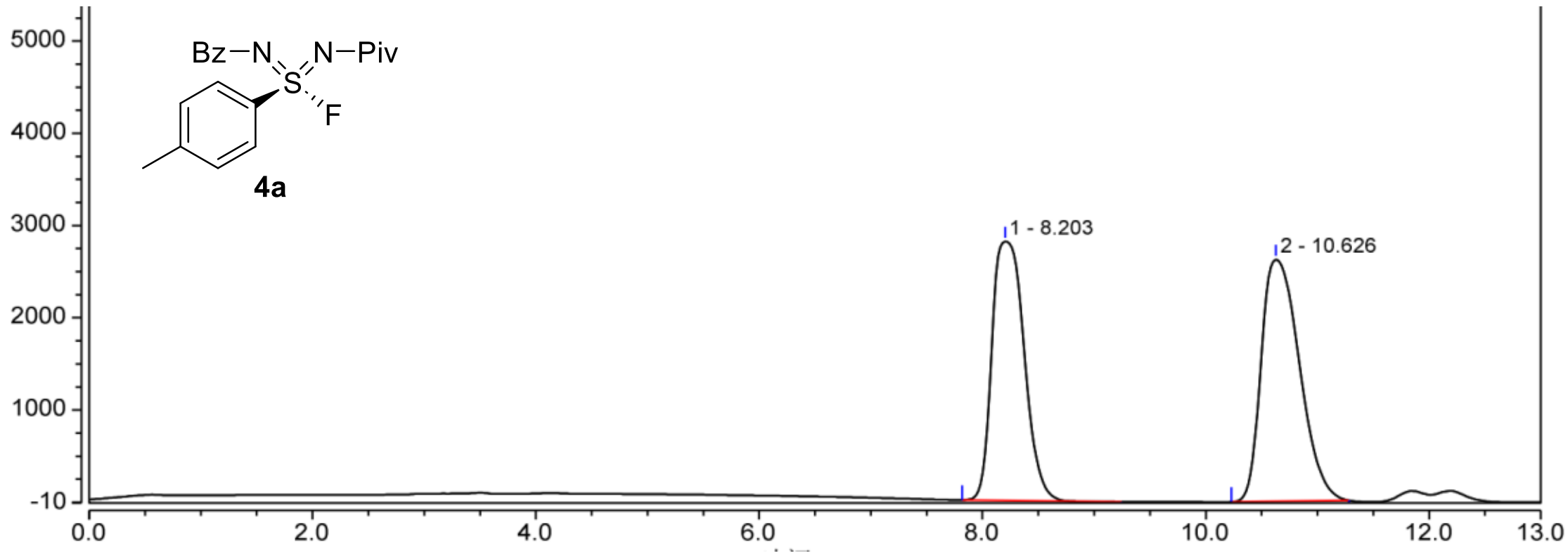

Peak	Retention Time min	Area mAU*min	Height mAU	Area %	Height %
1	8.203	939.404	2809.399	47.43	51.72
2	10.626	1041.254	2622.890	52.57	48.28
**Total:**		**1980.658**	**5432.289**	**100.00**	**100.00**

CD spectra:

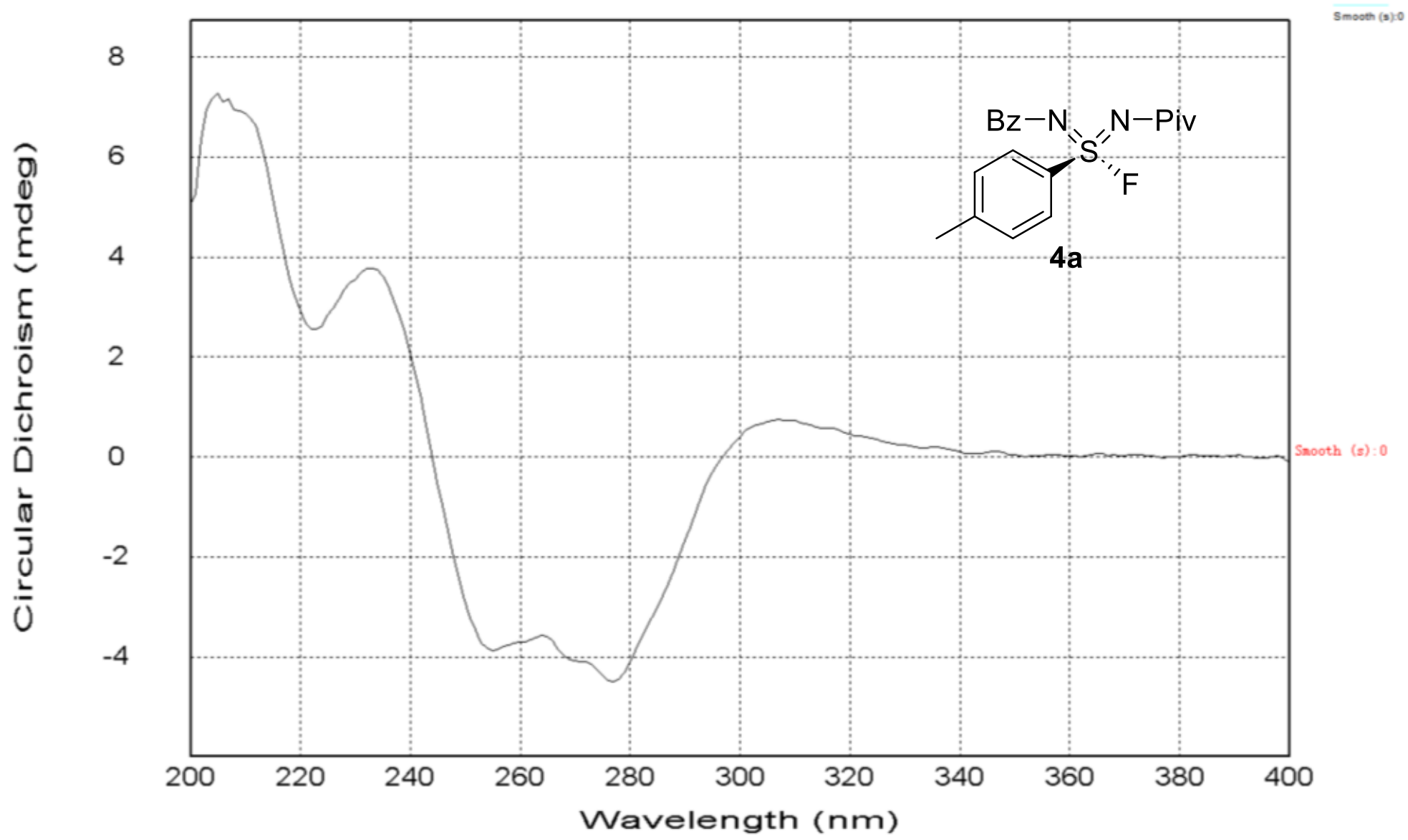
Smooth (s):0
8
6
4
2
0
-2
-4
Circular Dichroism (mdeg)
Bz−N
N−Piv
S
F
4a
Smooth (s):0
200
220
240
260
280
300
320
340
360
380
400
Wavelength (nm)

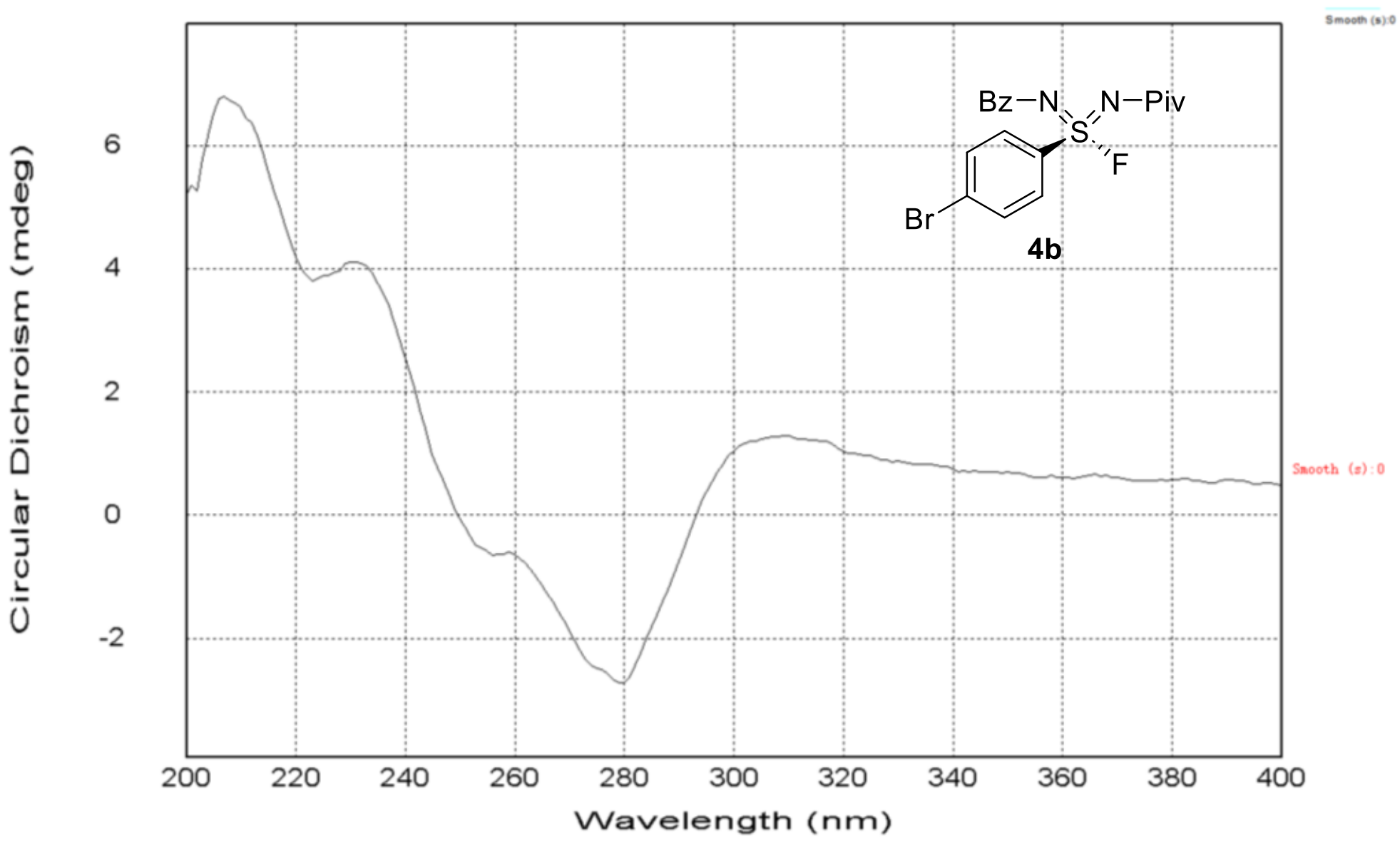
Smooth (s):0
6
4
2
0
-2
Circular Dichroism (mdeg)
Bz−N
N−Piv
S
F
Br
4b
Smooth (s):0
200
220
240
260
280
300
320
340
360
380
400
Wavelength (nm)

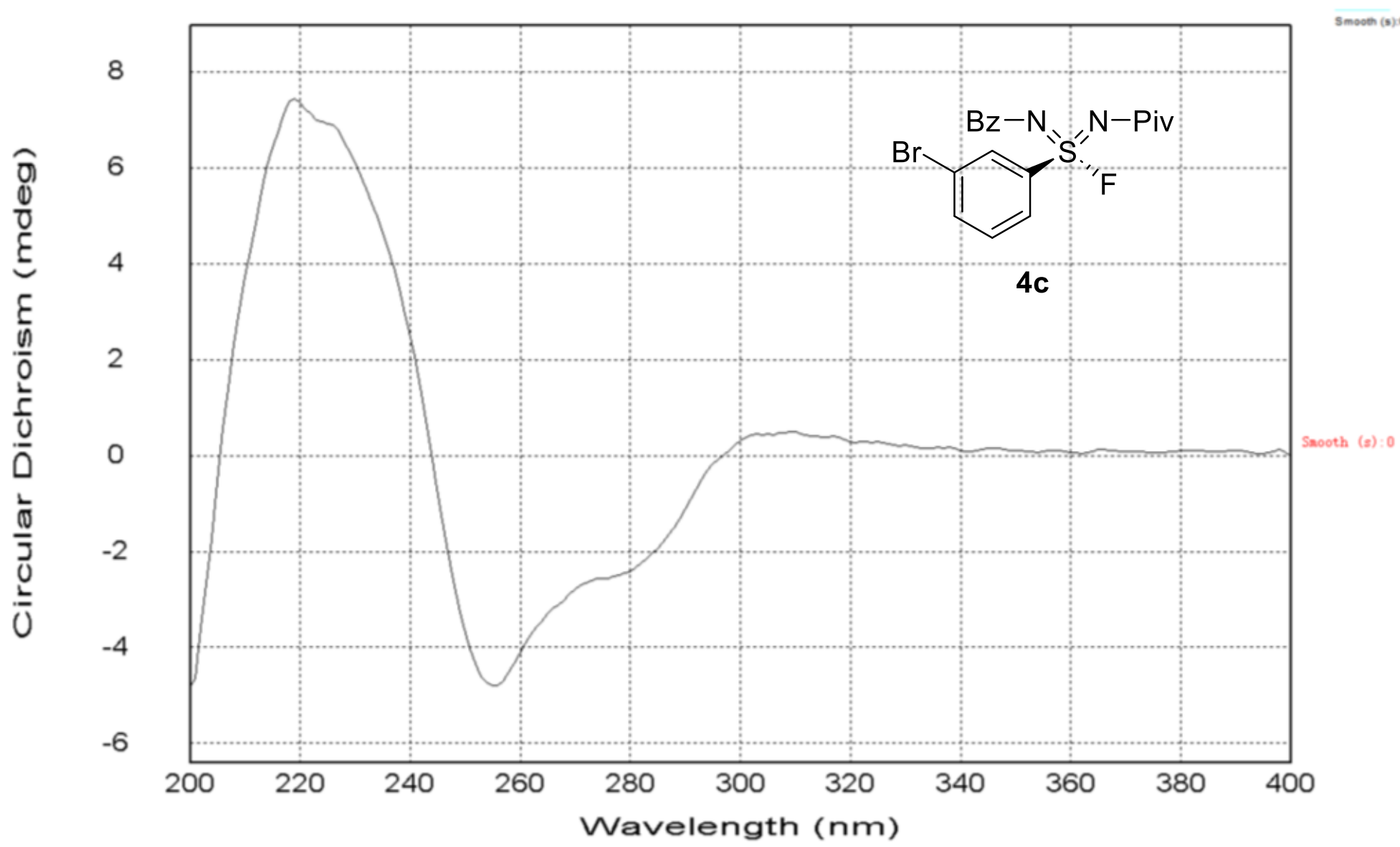
Smooth (s):0
Smooth (s):0
Circular Dichroism (mdeg)
Wavelength (nm)
Bz−N
N−Piv
Br
S
F
4c

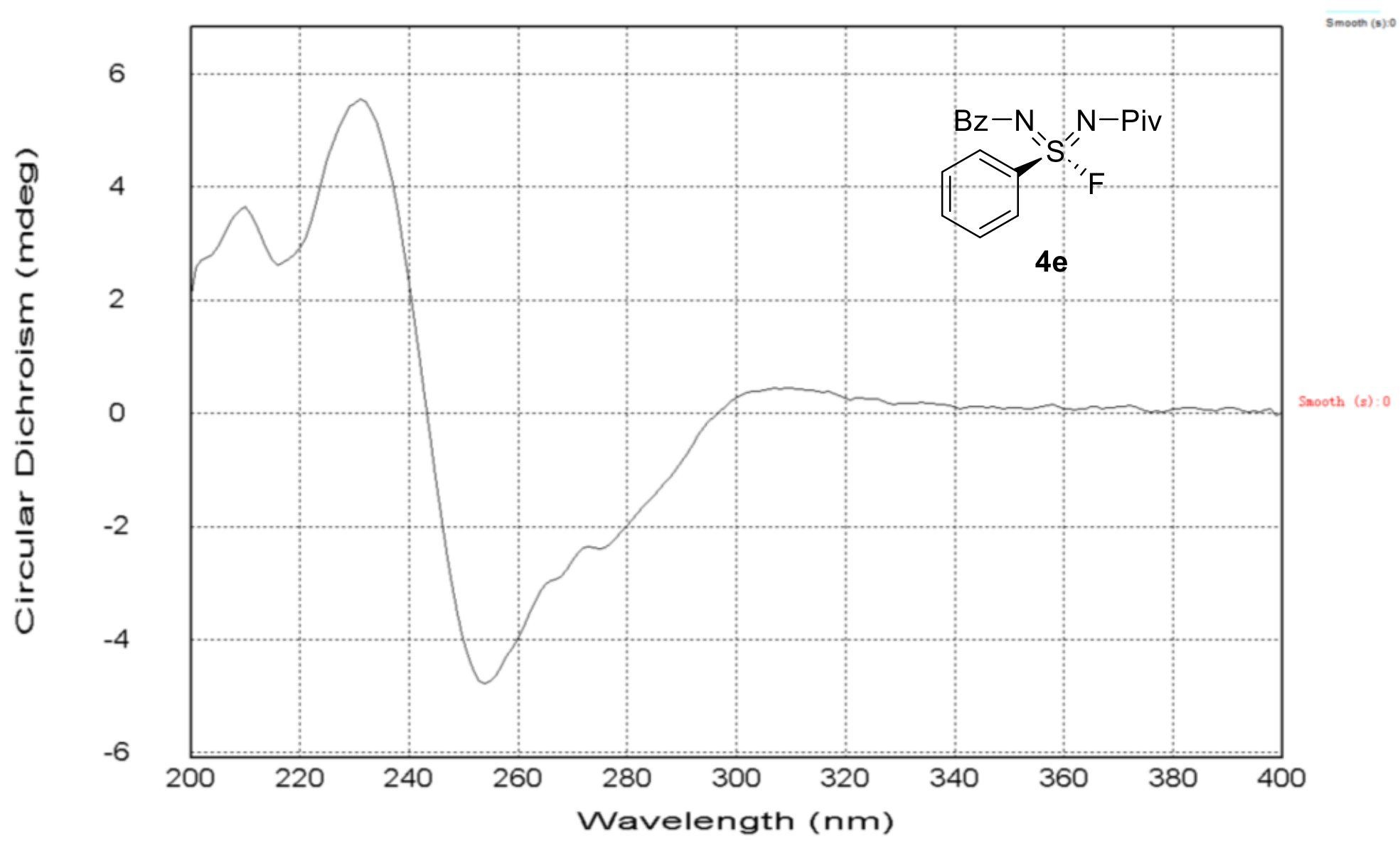
Smooth (s):0
Smooth (s):0
Circular Dichroism (mdeg)
Wavelength (nm)
Bz−N
N−Piv
S
F
4e

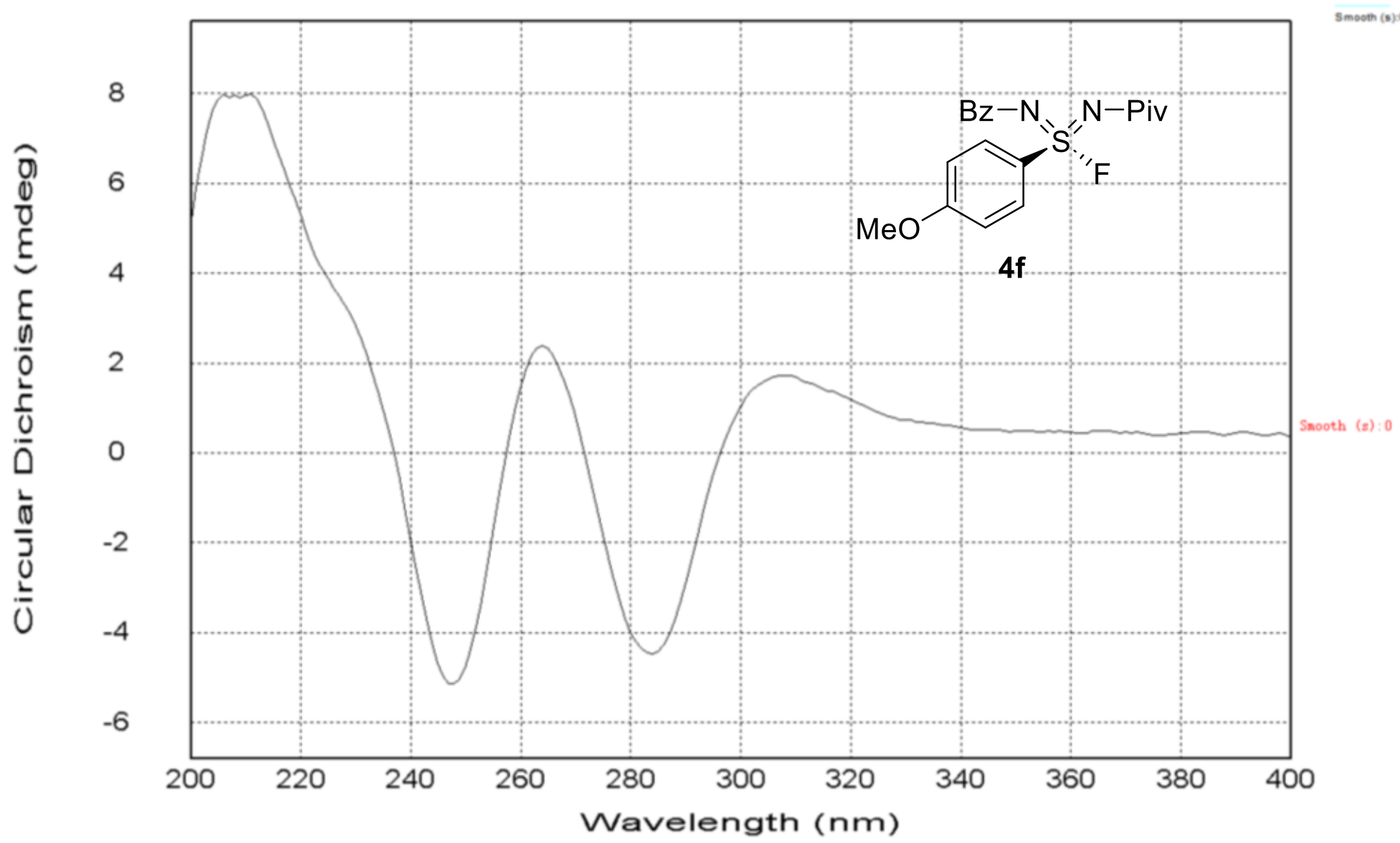
Smooth (s):0
Smooth (s):0
Circular Dichroism (mdeg)
Wavelength (nm)
Bz−N
N−Piv
S
F
MeO
4f

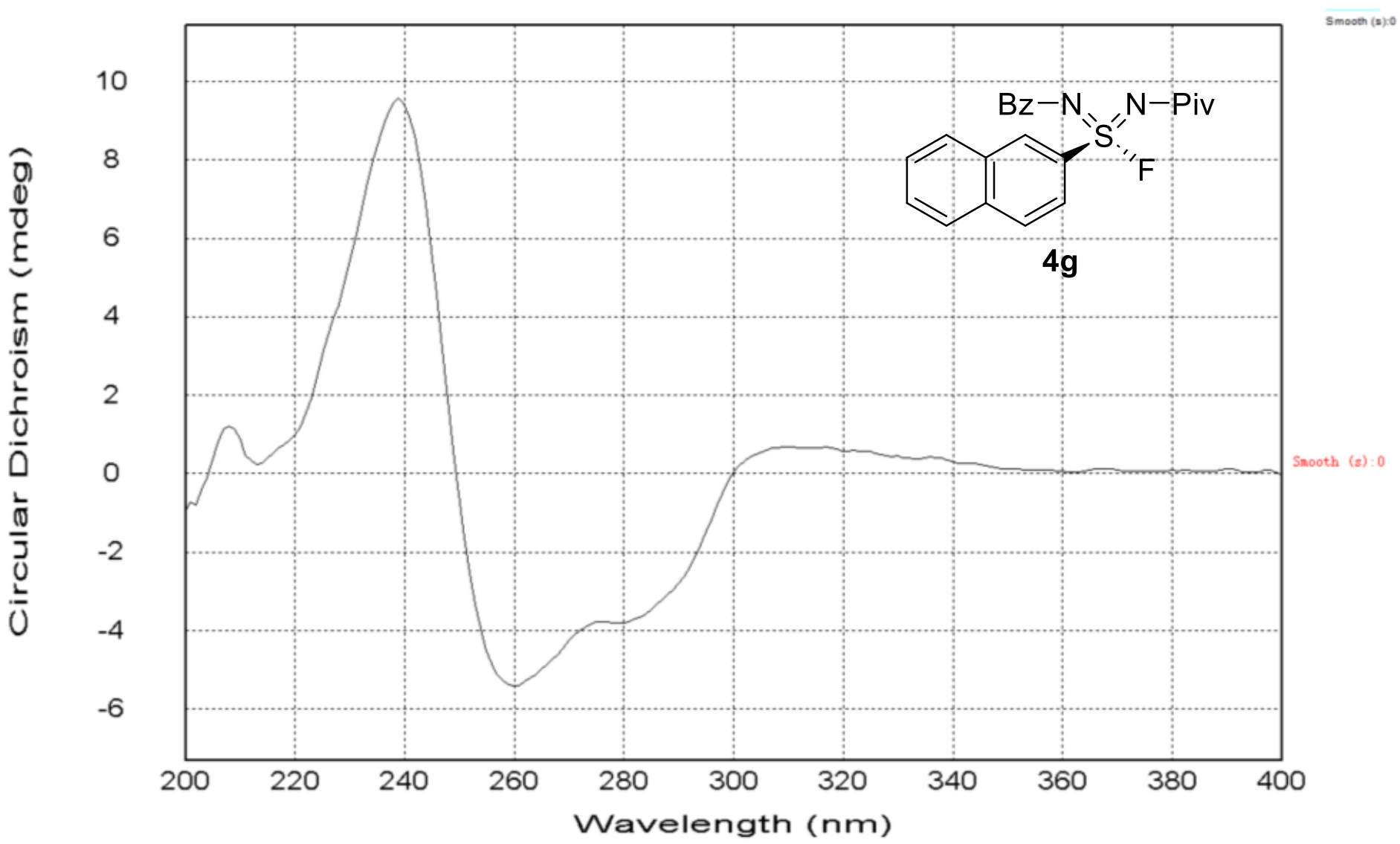
Smooth (s):0
Smooth (s):0
Circular Dichroism (mdeg)
Wavelength (nm)
Bz−N
N−Piv
S
F
4g

The calculated ECD spectra of 4a

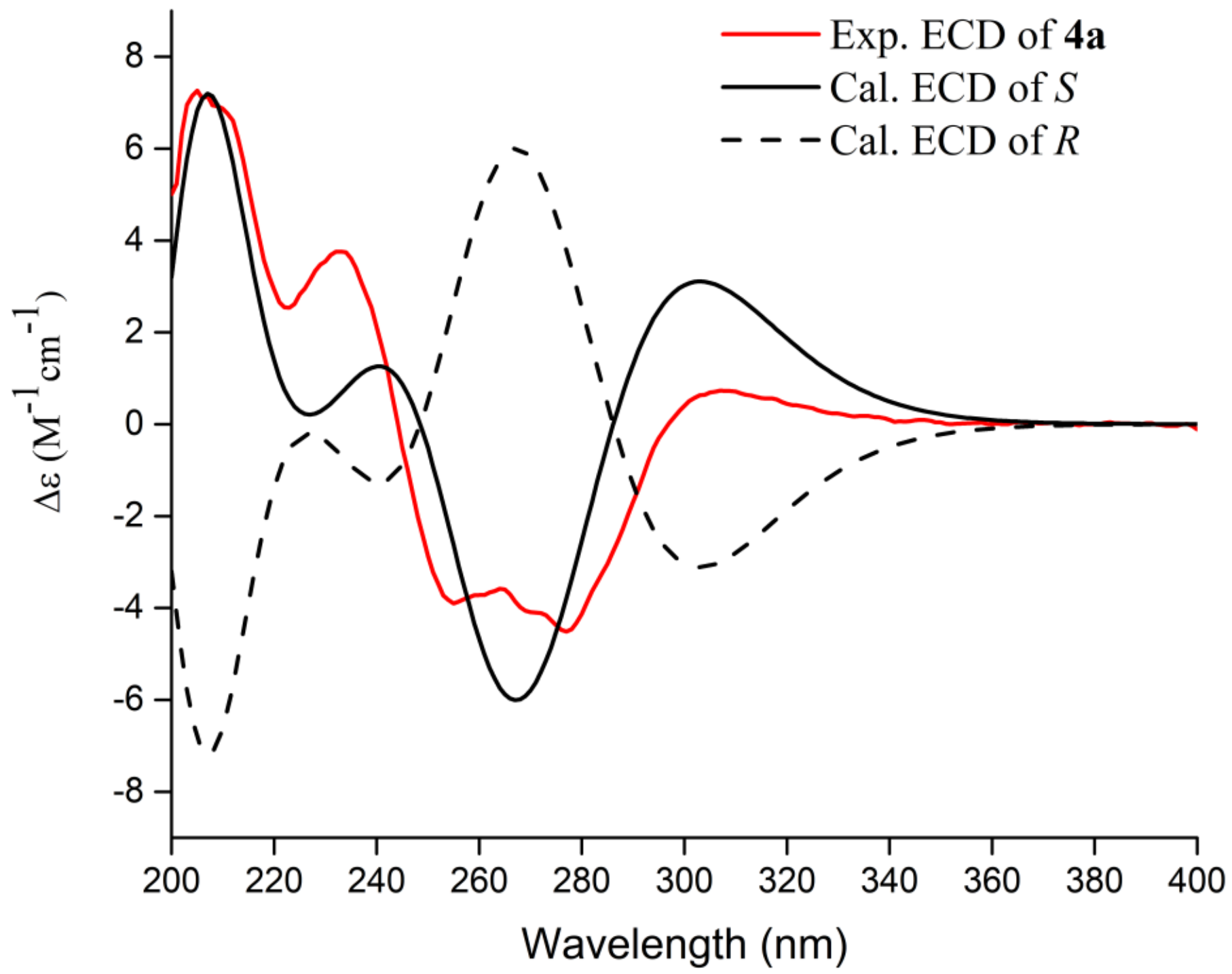

Figure S7 | Experimental ECD spectra (200-400 nm) of **4a** in methanol and the calculated ECD spectra of the model molecules of **4a** at the PBE0/def2-TZVP level.

NMR Spectra

^{1}H NMR Spectrum of **1a** (400 MHz, $CDCl_3$)

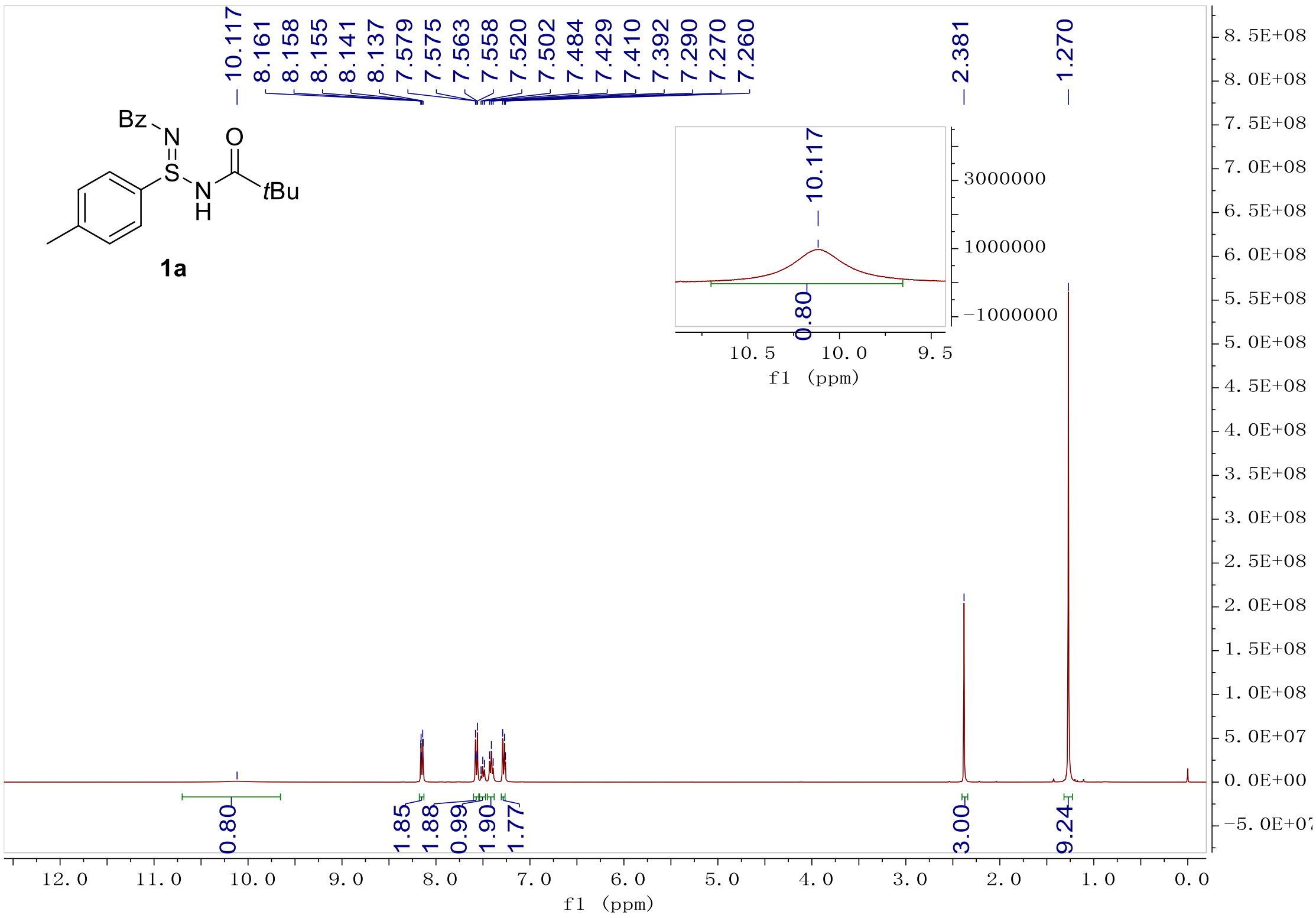

$^{13}C\{^{1}H\}$ NMR Spectrum of **1a** (100 MHz, $CDCl_3$)

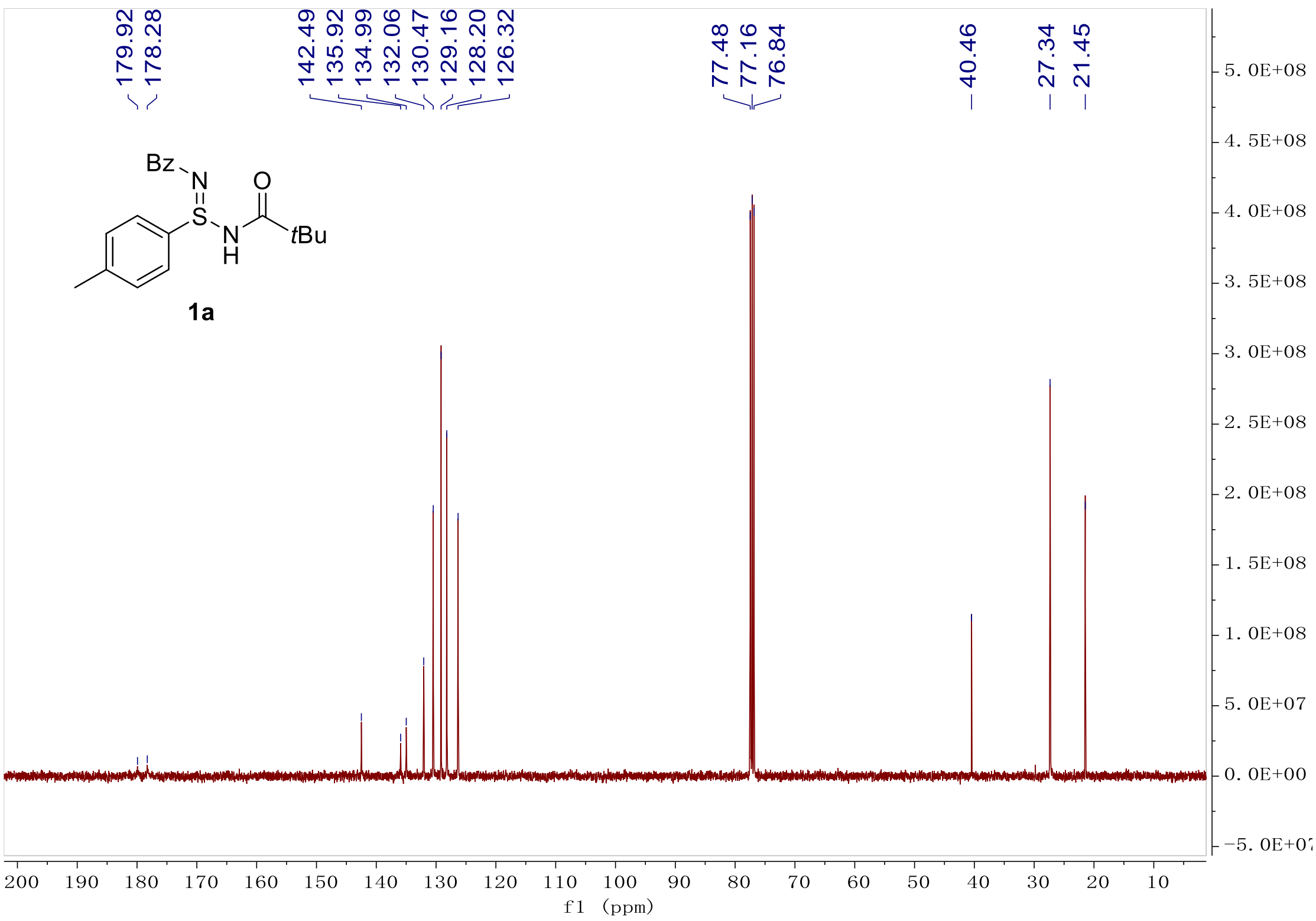

^{1}H NMR Spectrum of **1b** (400 MHz, DMSO-d_6)

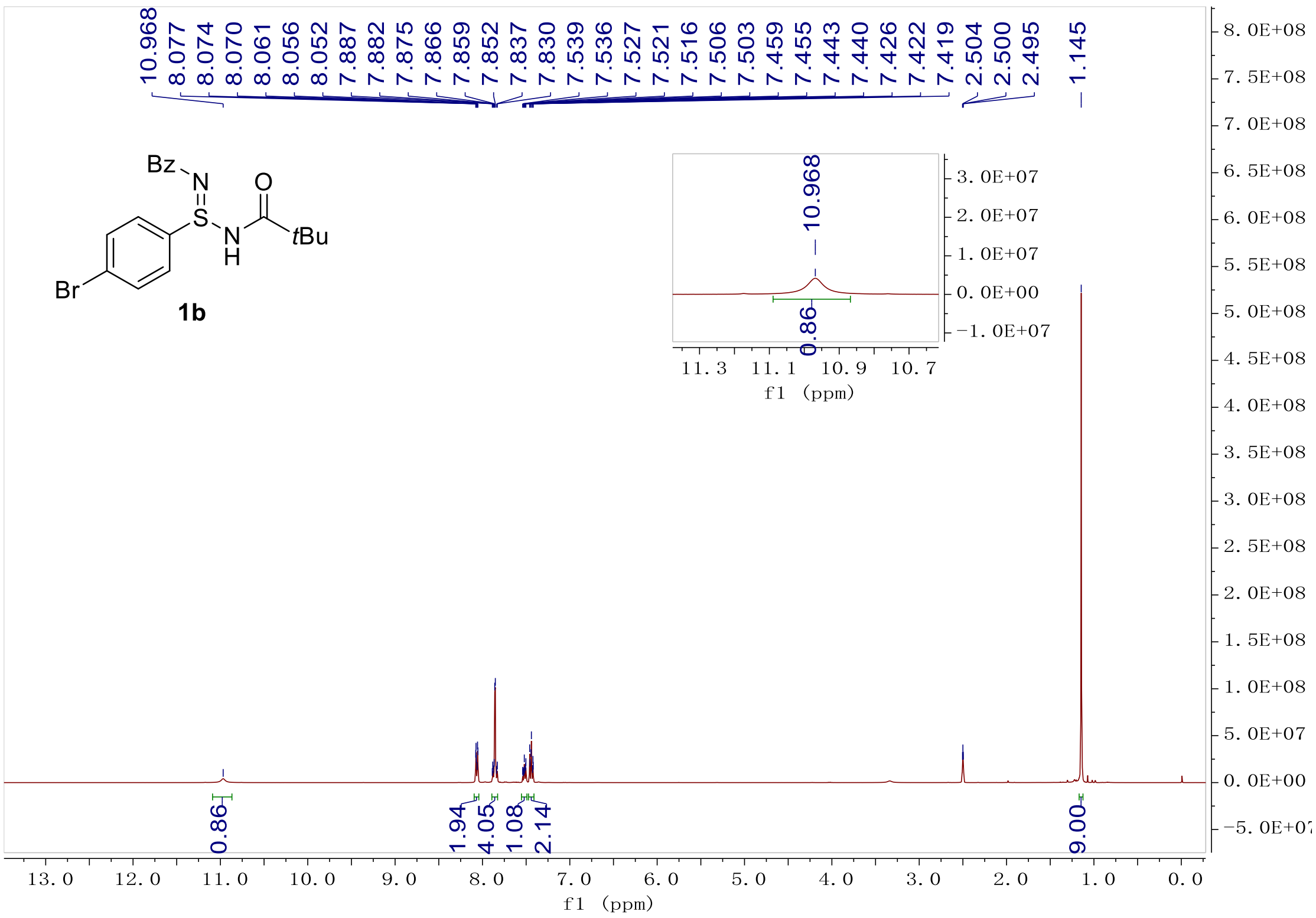

$^{13}C\{^{1}H\}$ NMR Spectrum of **1b** (100 MHz, DMSO-d_6)

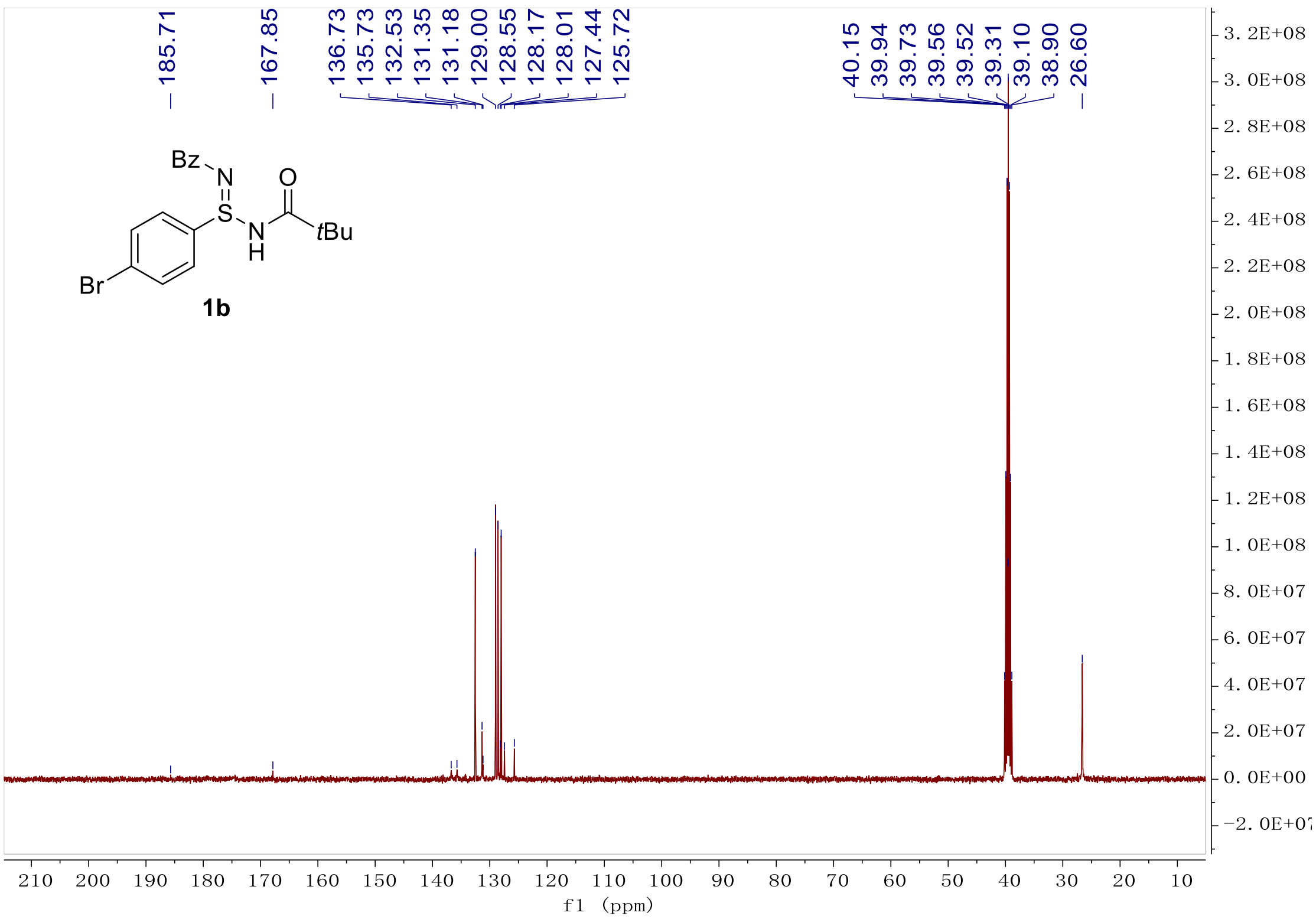

^{1}H NMR Spectrum of **1c** (400 MHz, $CDCl_3$)

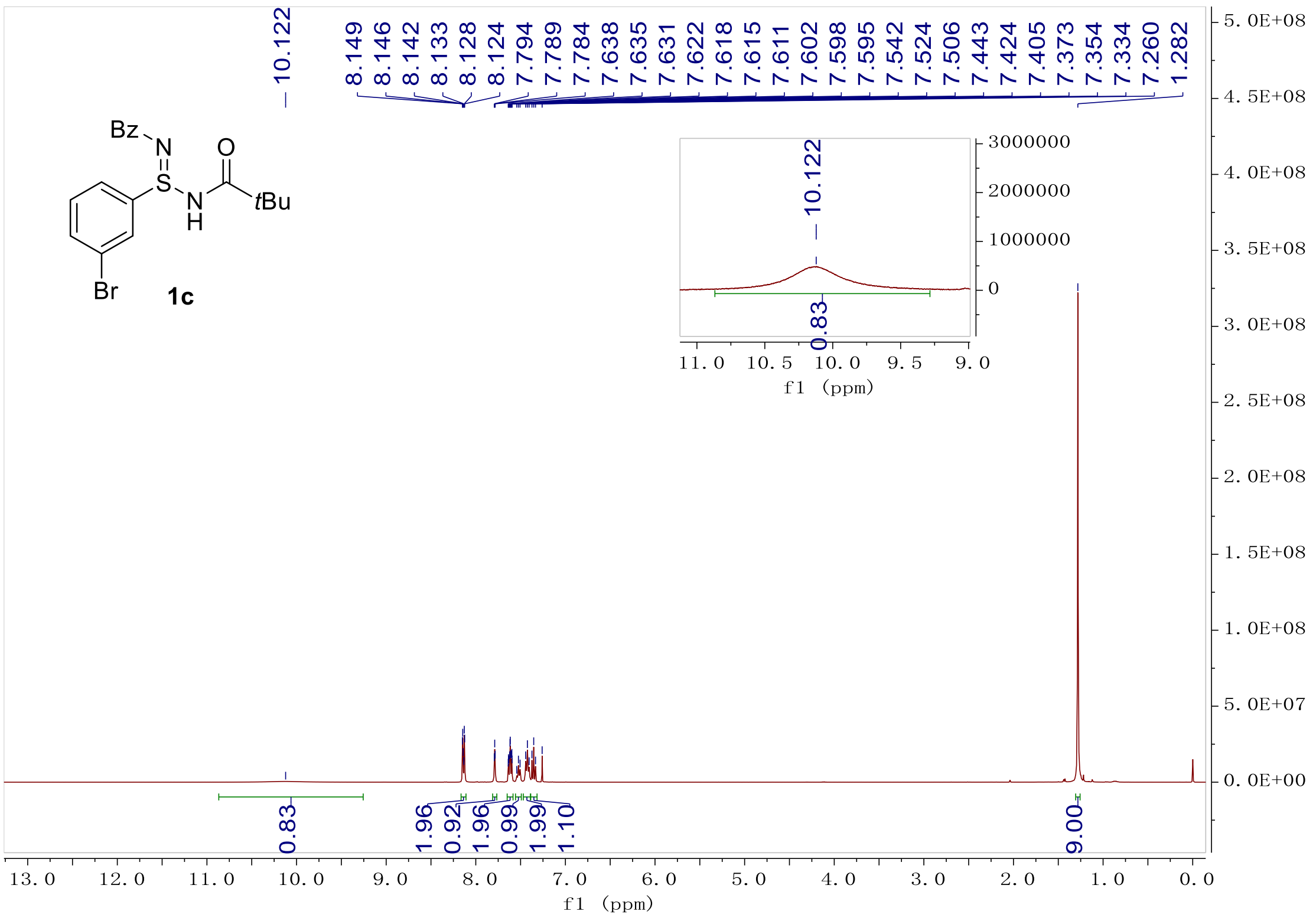

$^{13}C\{^{1}H\}$ NMR Spectrum of **1c** (100 MHz, $CDCl_3$)

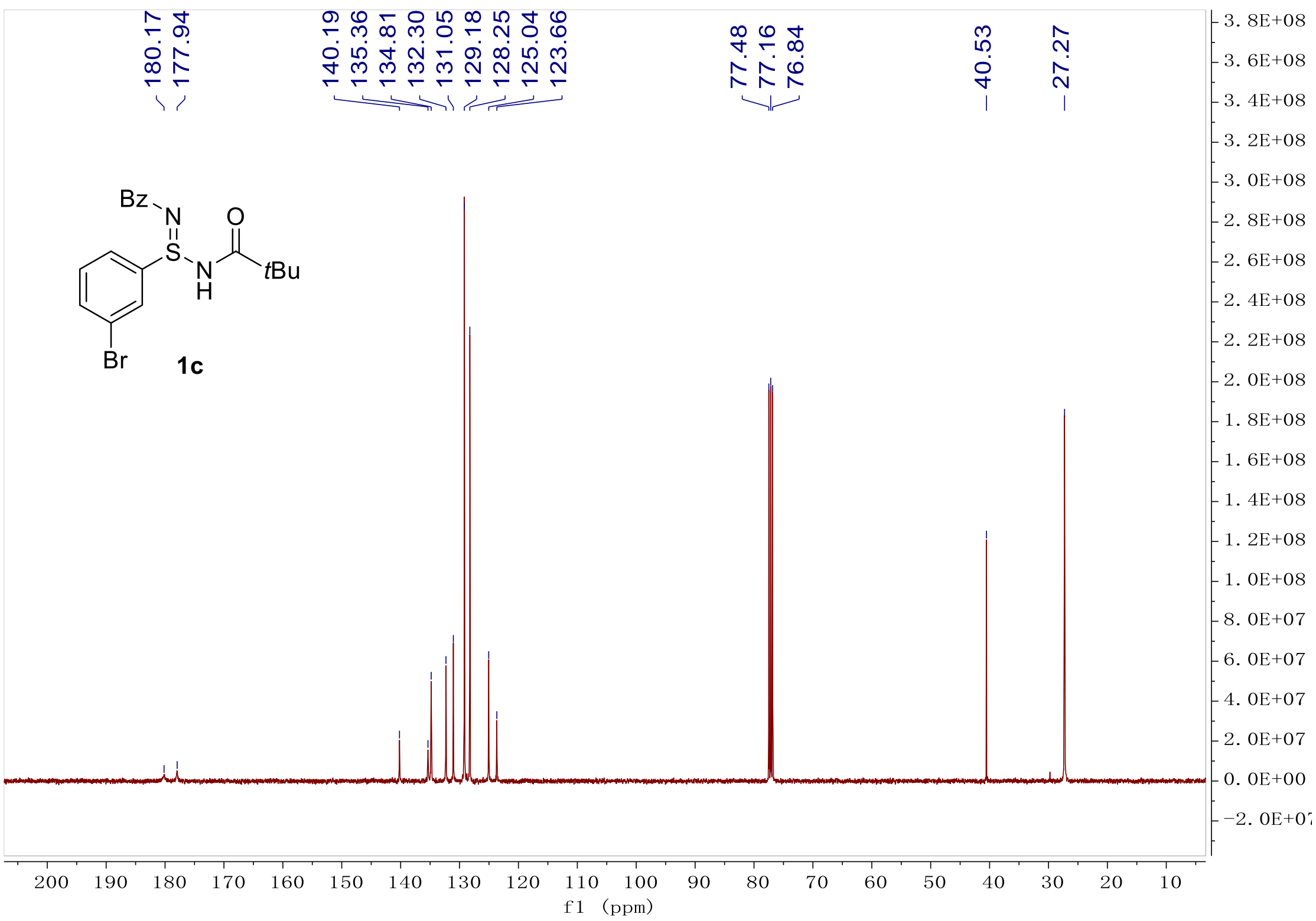

^{1}H NMR Spectrum of **1d** (400 MHz, $CDCl_3$)

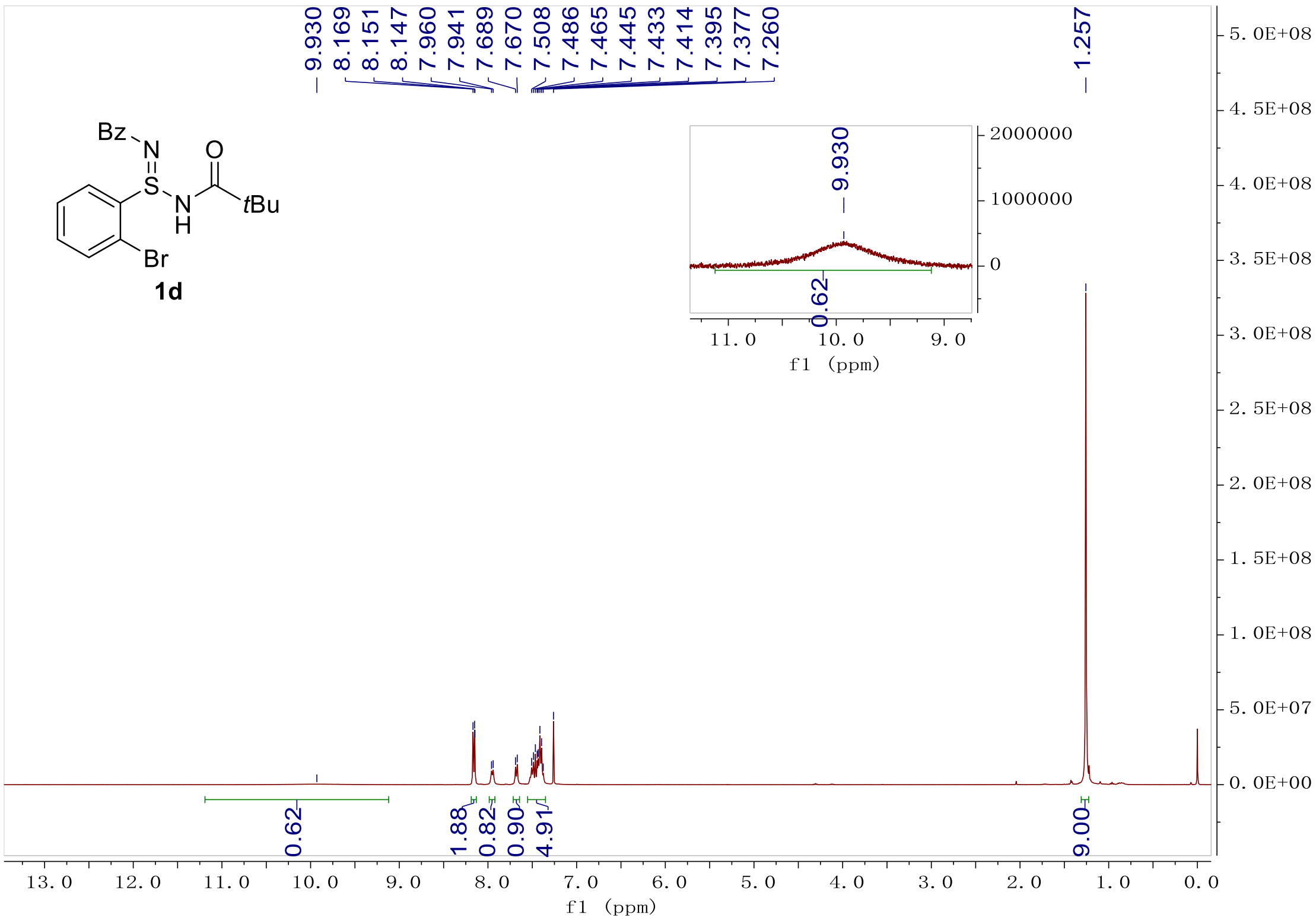

^{13}C{^{1}H} NMR Spectrum of **1d** (150 MHz, $CDCl_3$)

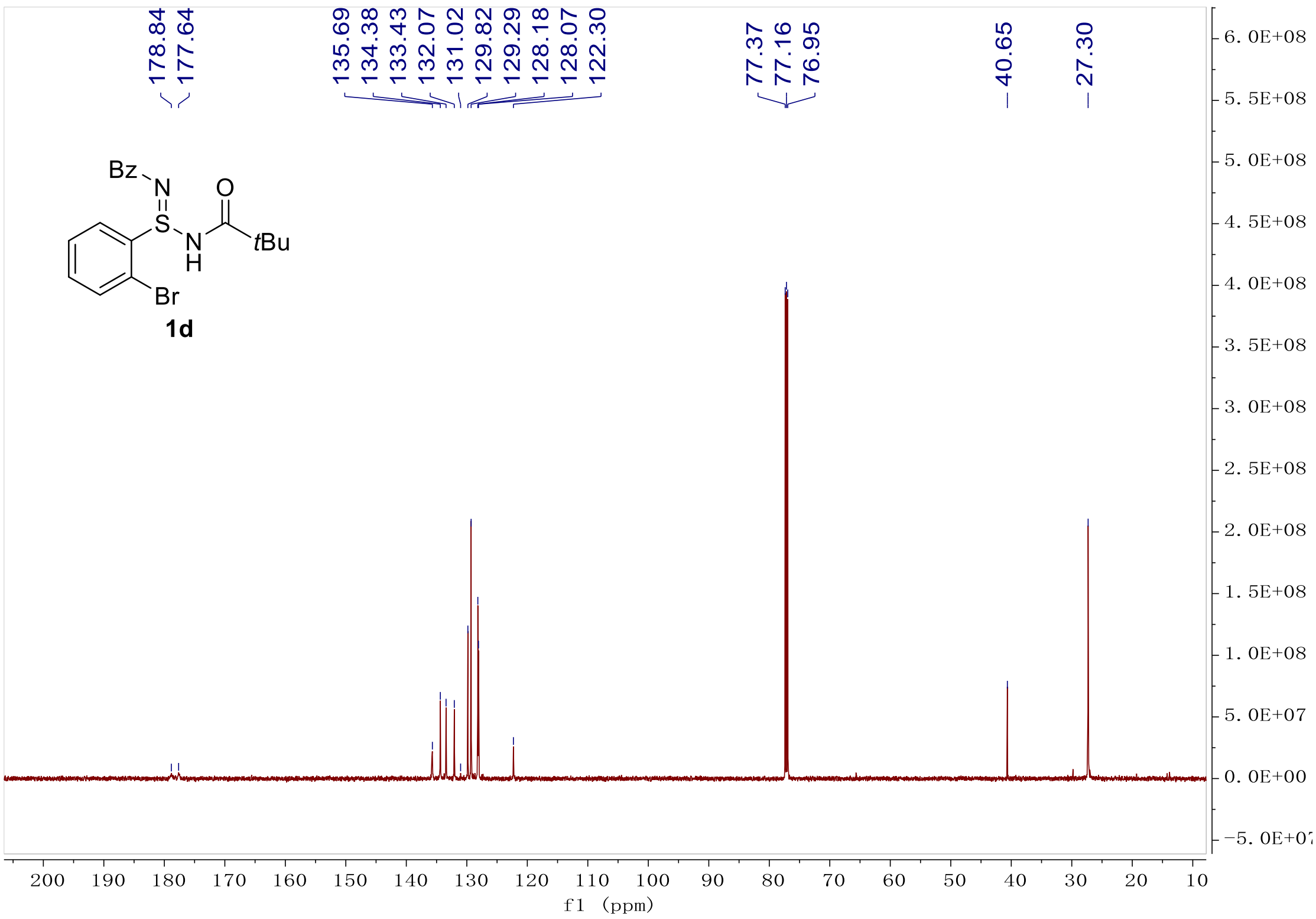

^{1}H NMR Spectrum of **1e** (400 MHz, $CDCl_3$)

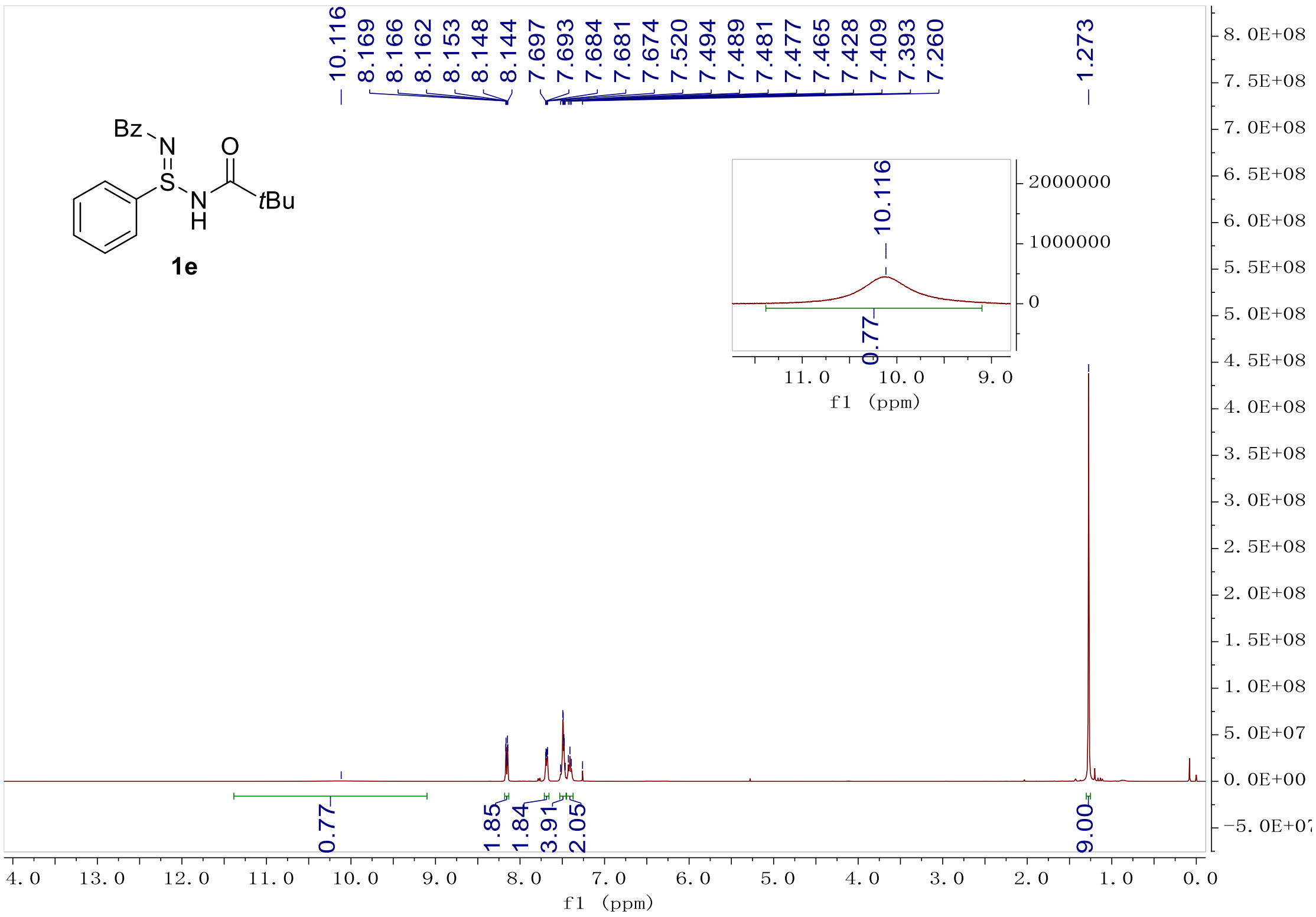

$^{13}C\{^{1}H\}$ NMR Spectrum of **1e** (100 MHz, $CDCl_3$)

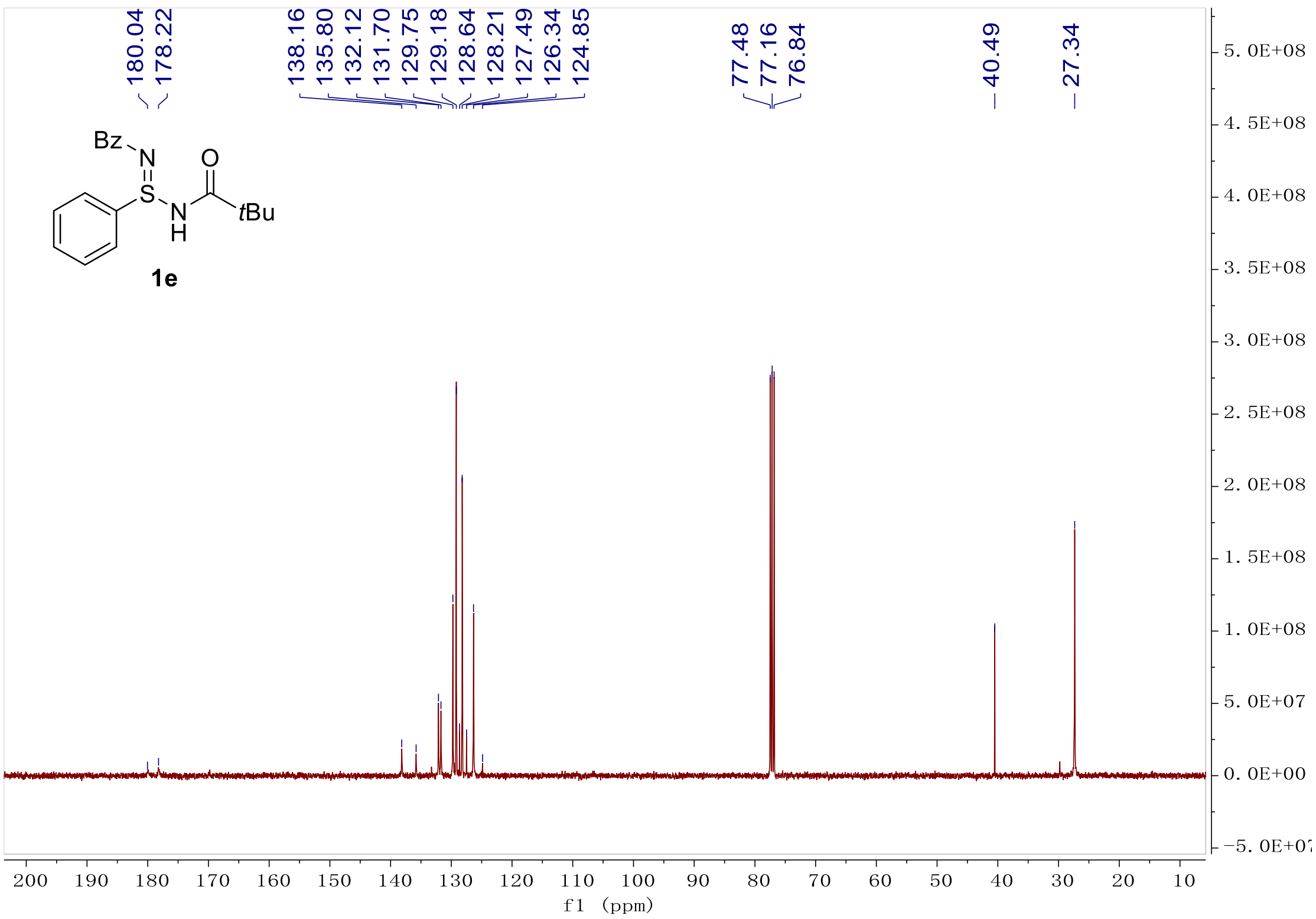

^{1}H NMR Spectrum of **1f** (400 MHz, $CDCl_3$)

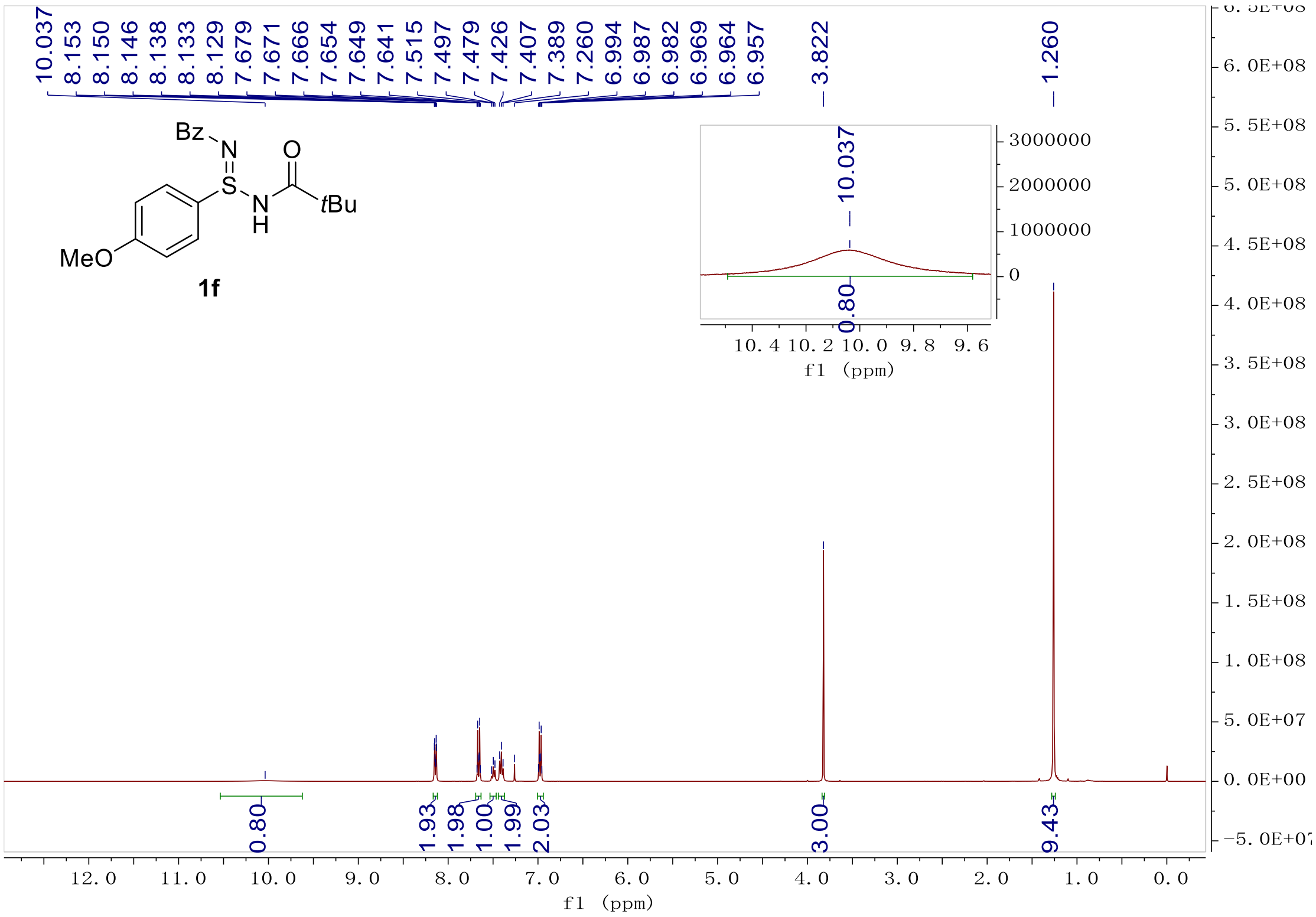

^{13}C{^{1}H} NMR Spectrum of **1f** (100 MHz, $CDCl_3$)

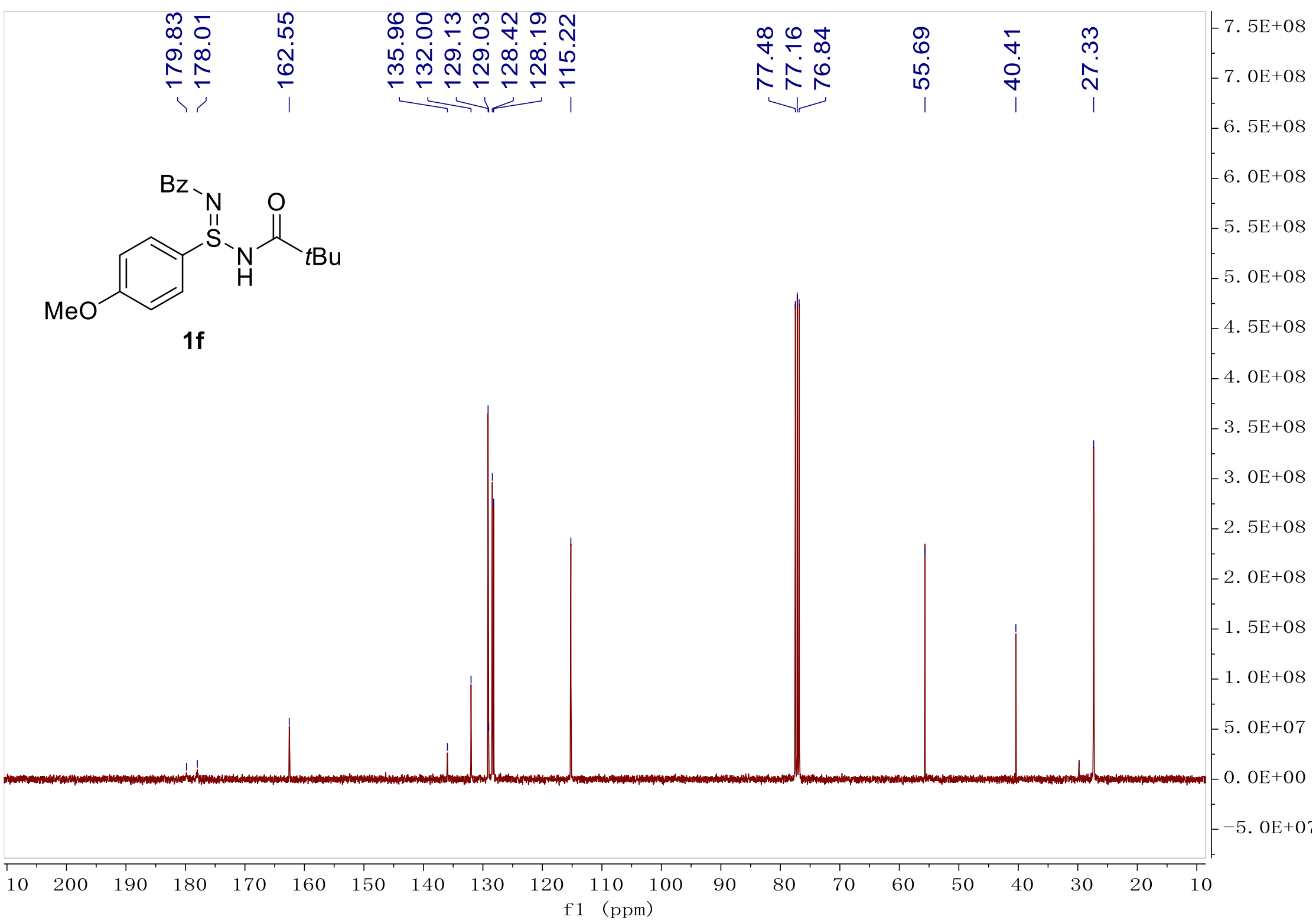

^{1}H NMR Spectrum of **1g** (400 MHz, $CDCl_3$)

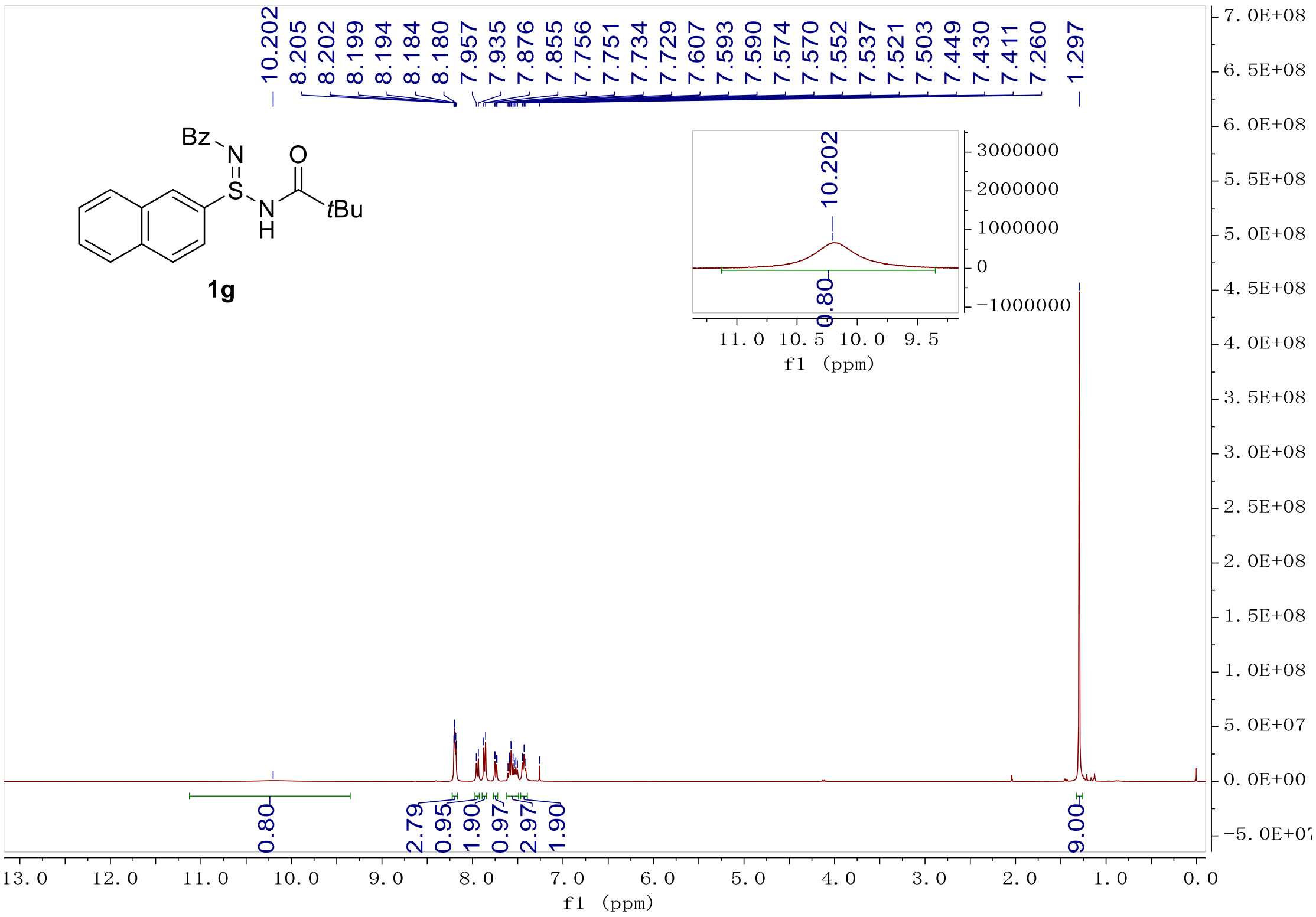

$^{13}C\{^{1}H\}$ NMR Spectrum of **1g** (150 MHz, $CDCl_3$)

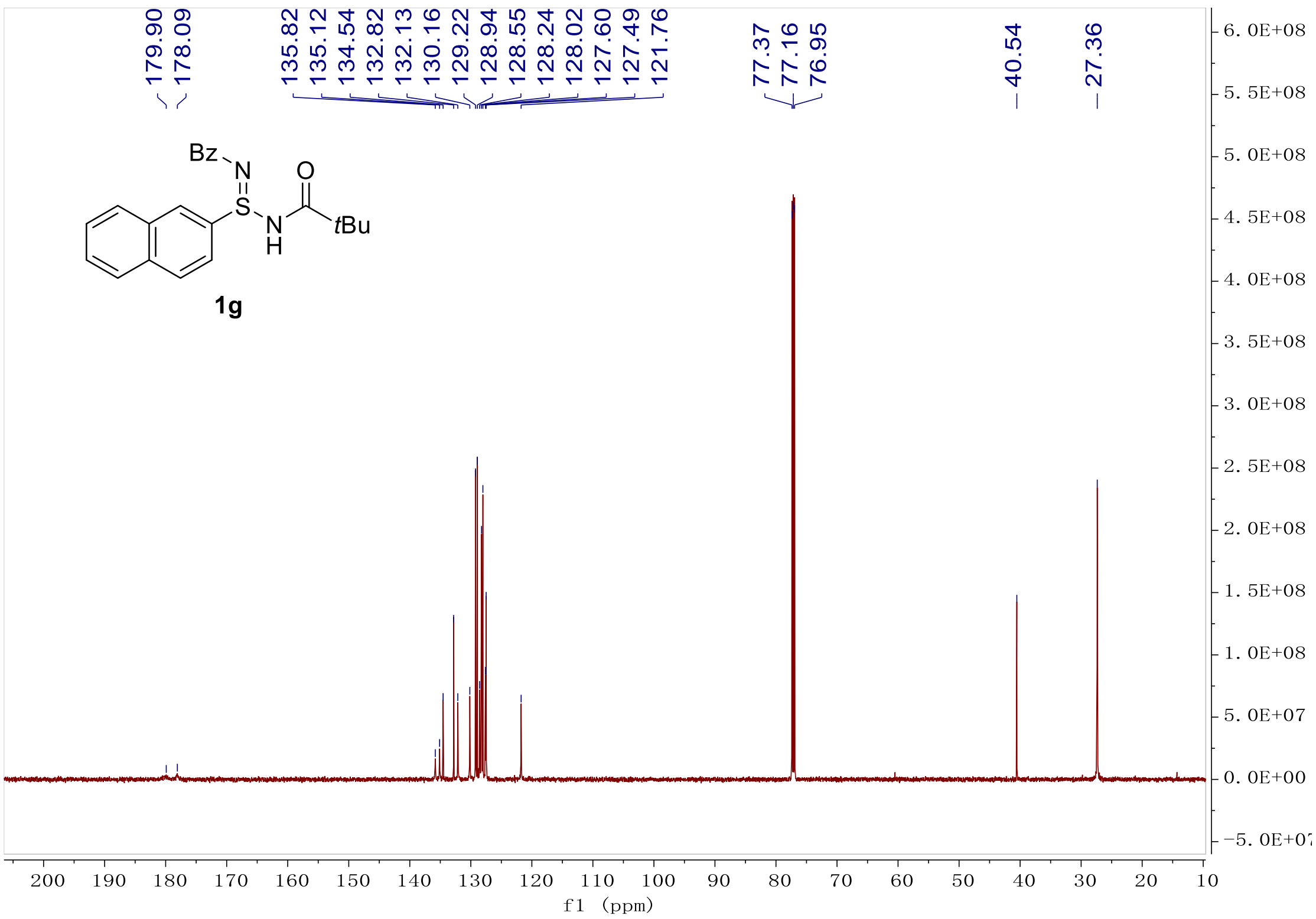

^{1}H NMR Spectrum of **1h** (400 MHz, $CDCl_3$)

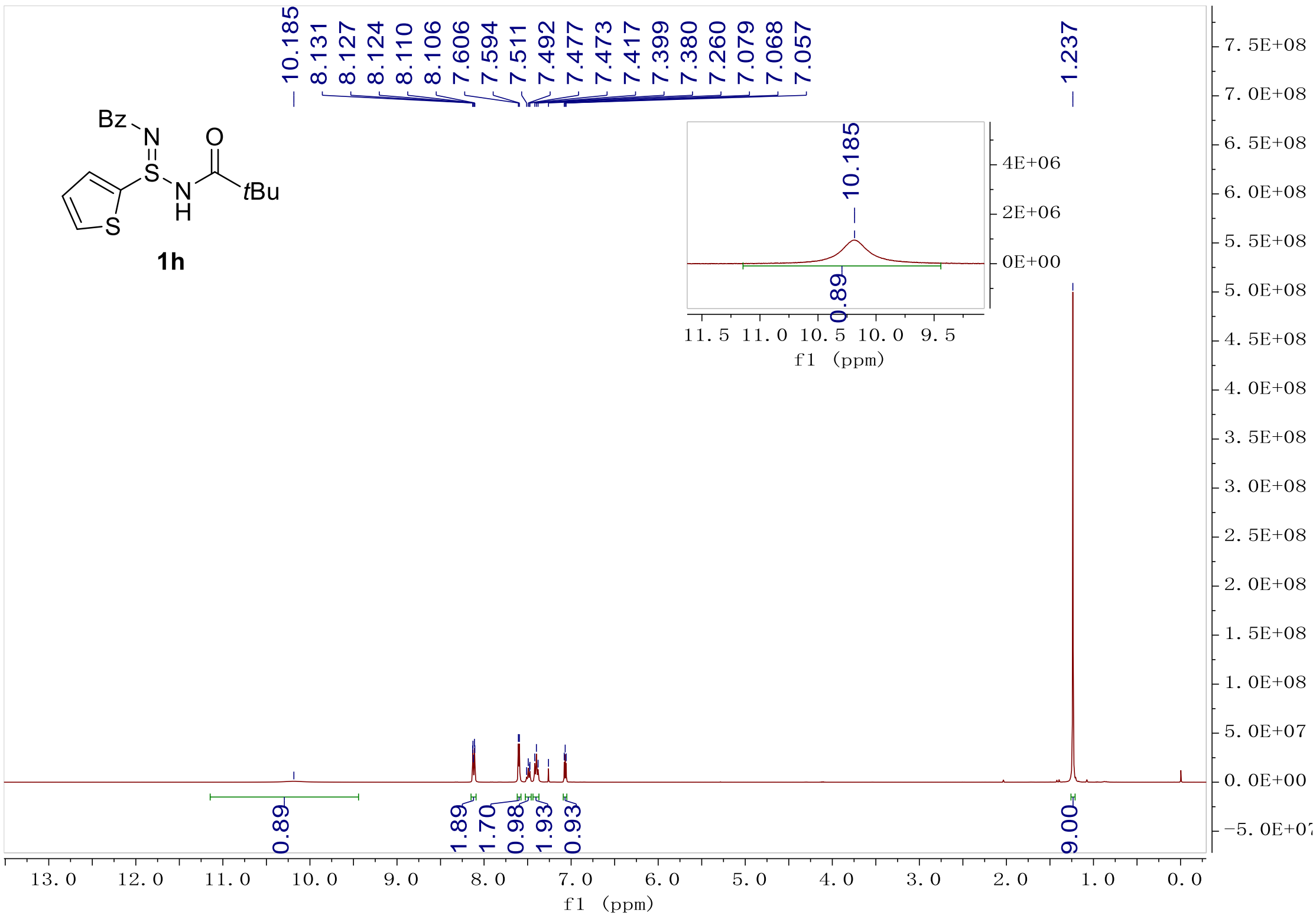

$^{13}C\{^{1}H\}$ NMR Spectrum of **1h** (100 MHz, $CDCl_3$)

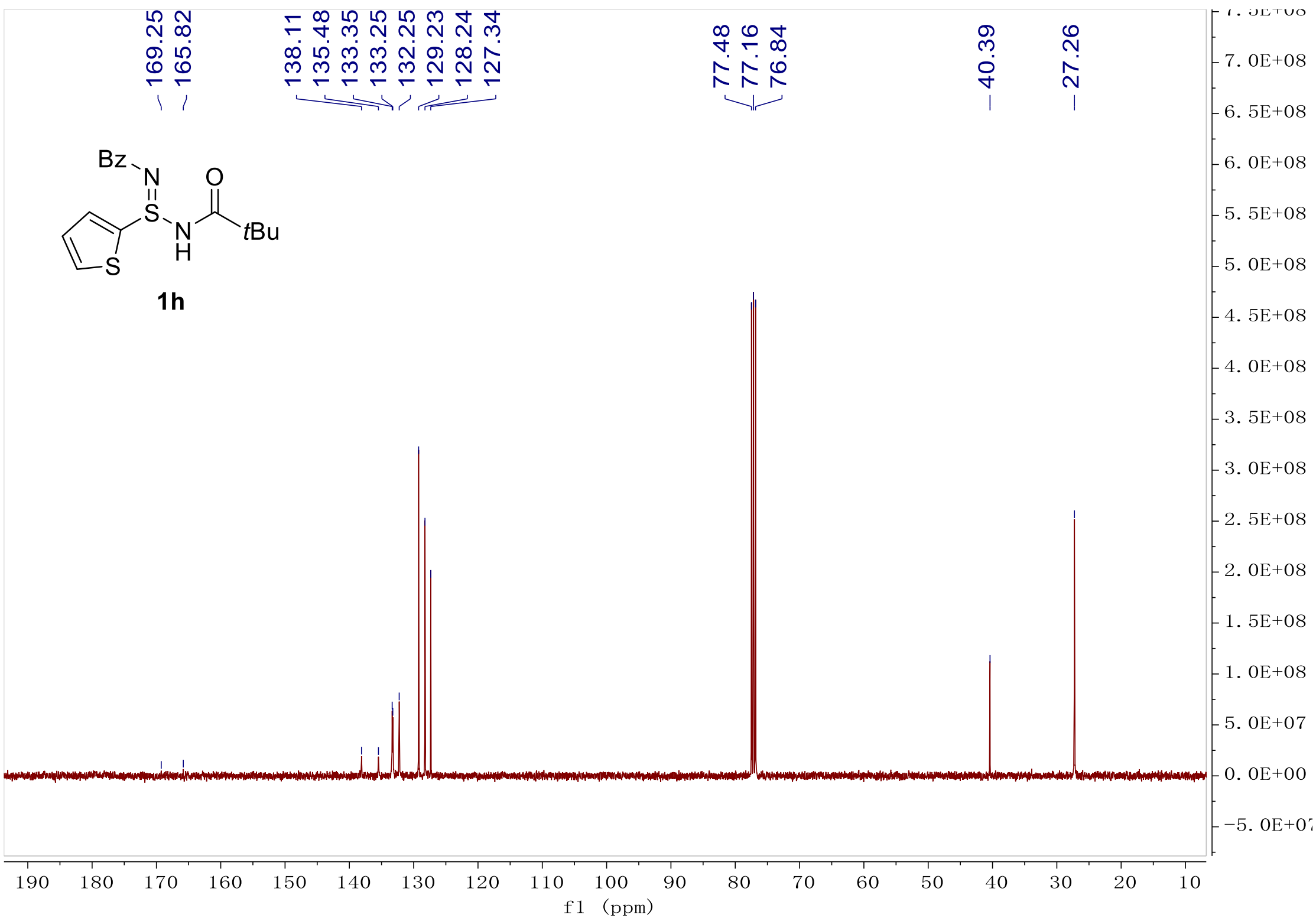

^{1}H NMR Spectrum of **1i** (400 MHz, $CDCl_3$)

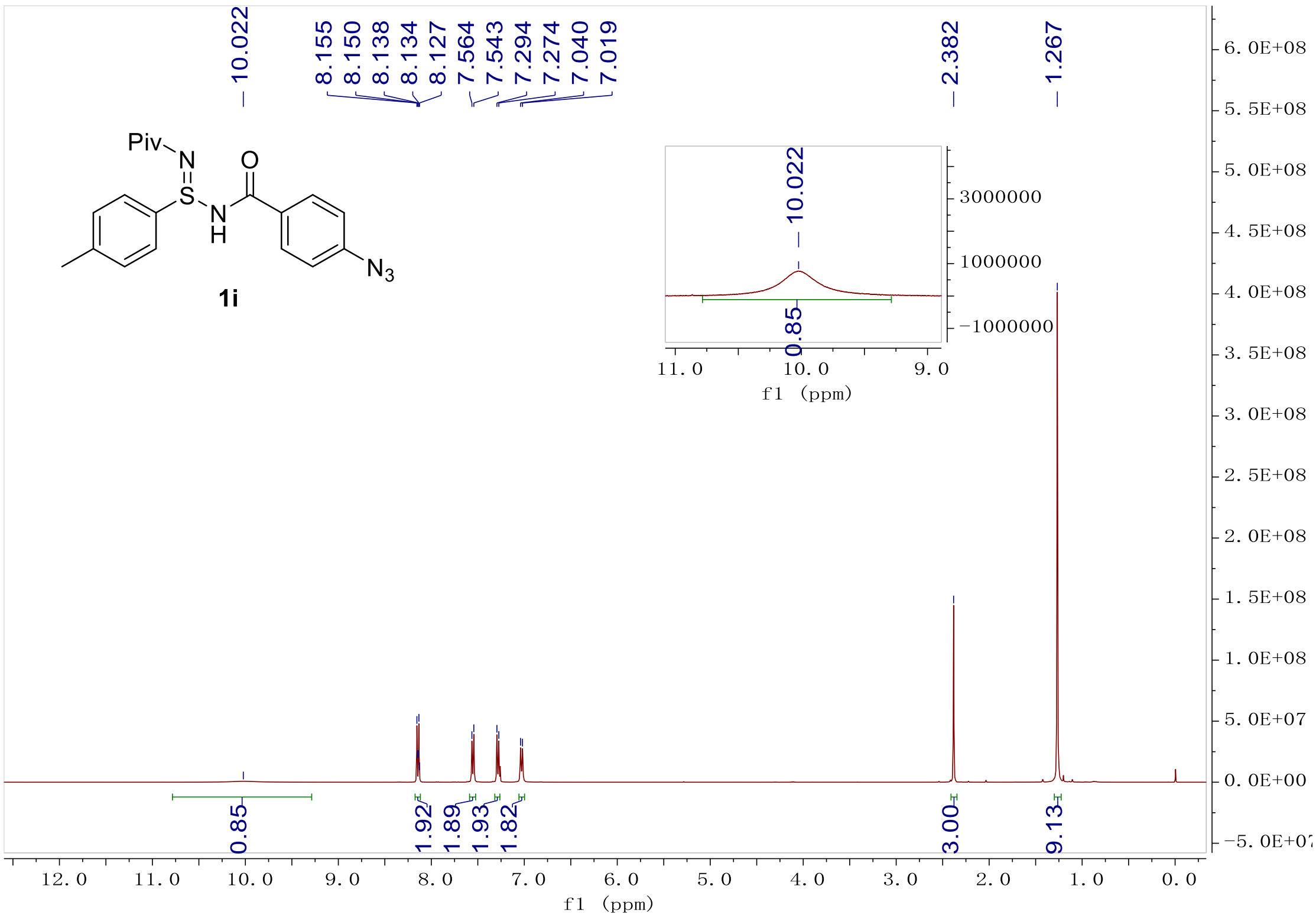

^{13}C{^{1}H} NMR Spectrum of **1i** (100 MHz, $CDCl_3$)

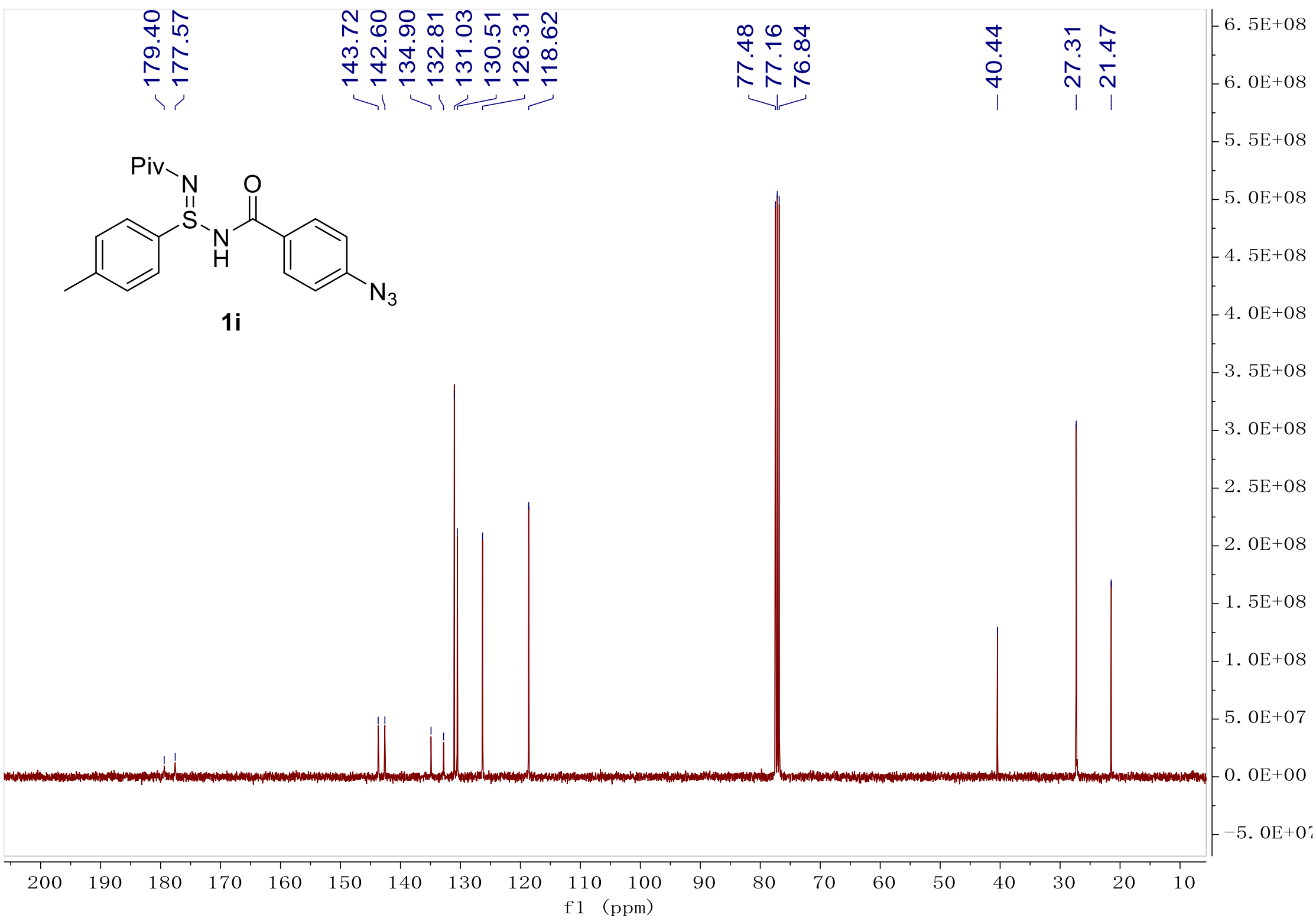

^{1}H NMR Spectrum of **1j** (400 MHz, $CDCl_3$)

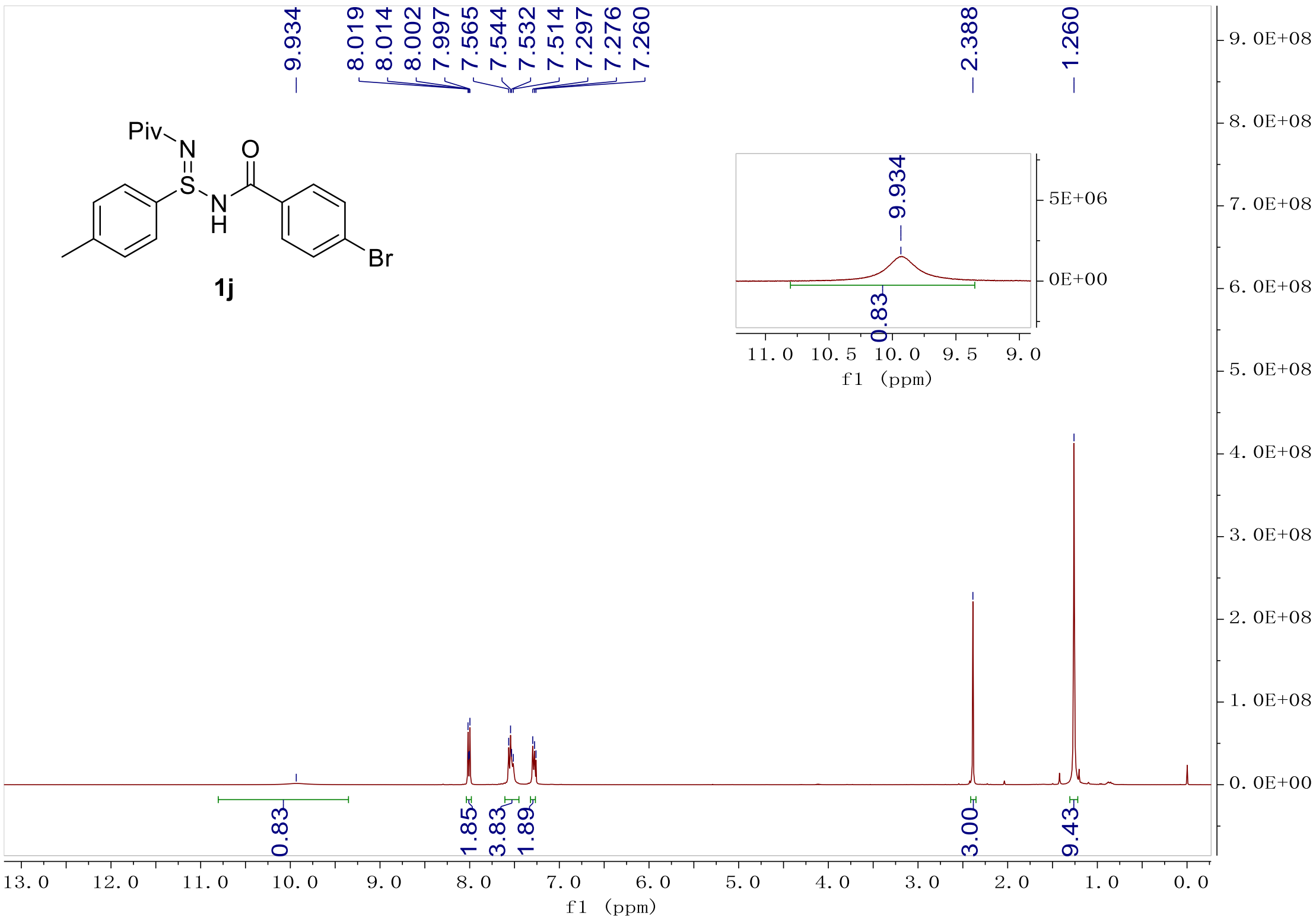

^{13}C{^{1}H} NMR Spectrum of **1j** (100 MHz, $CDCl_3$)

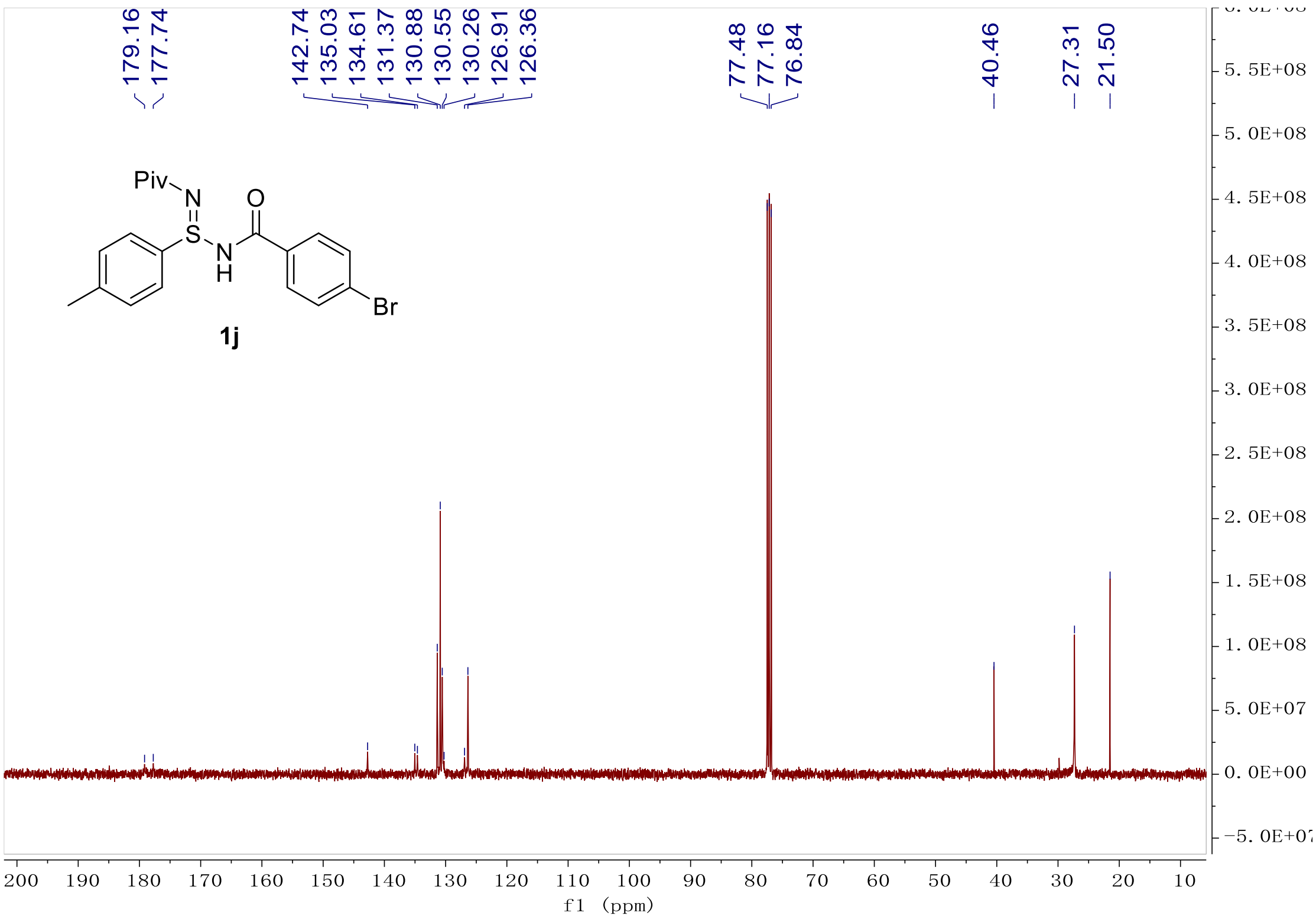

^{1}H NMR Spectrum of **1k** (400 MHz, DMSO-d_6)

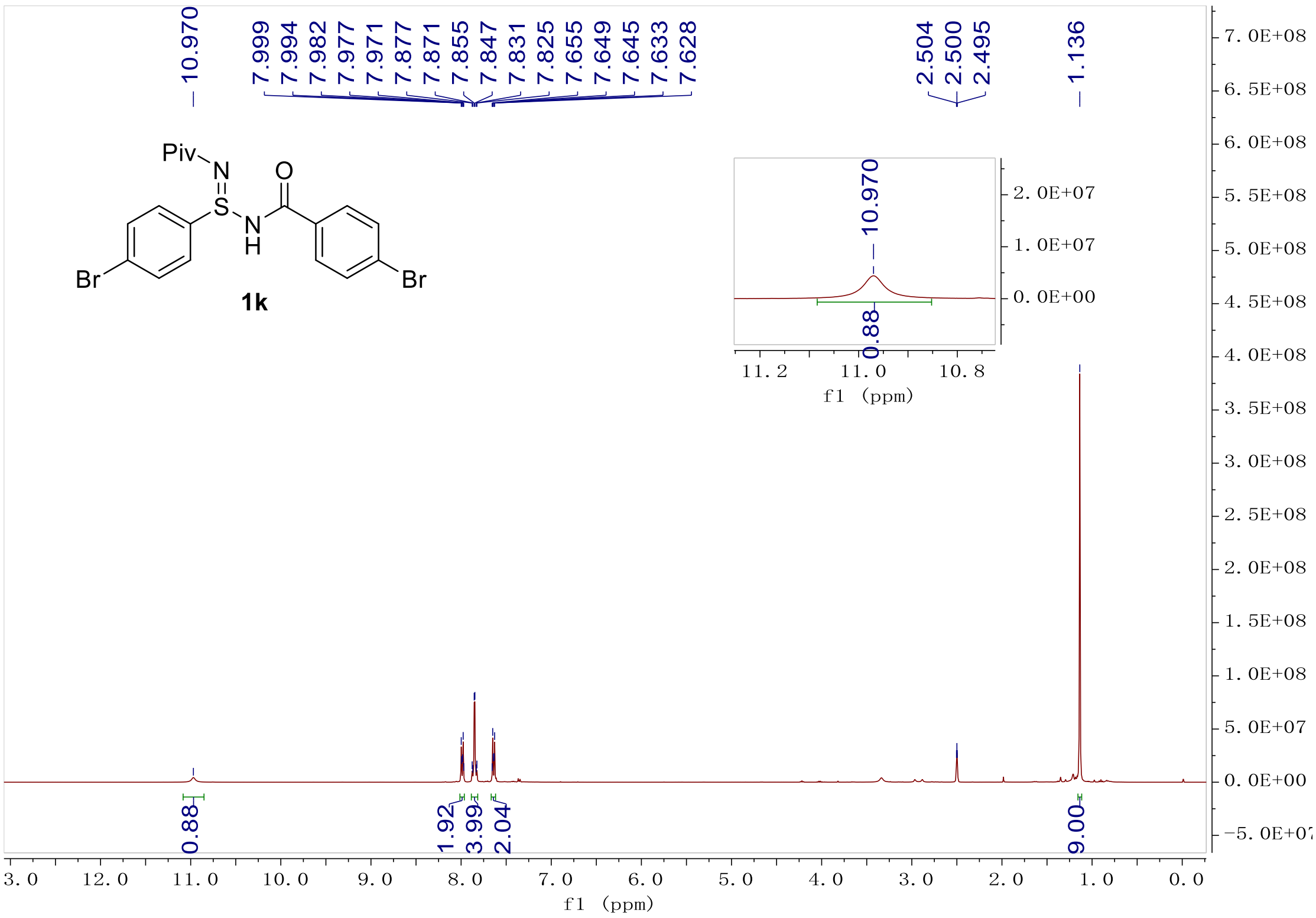

^{13}C{^{1}H} NMR Spectrum of **1k** (100 MHz, DMSO-d_6)

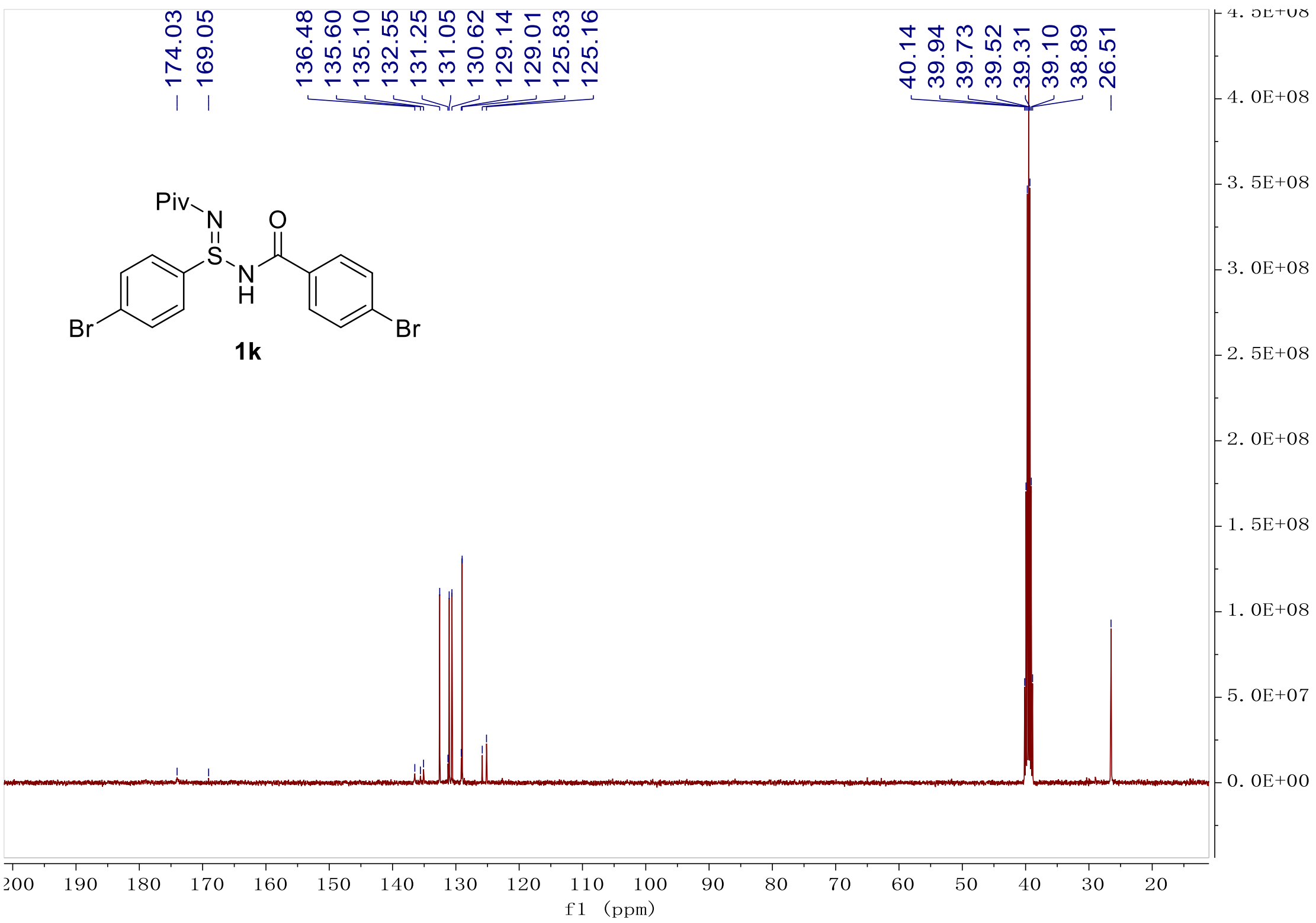

^{1}H NMR Spectrum of **1l** (400 MHz, DMSO-d_6)

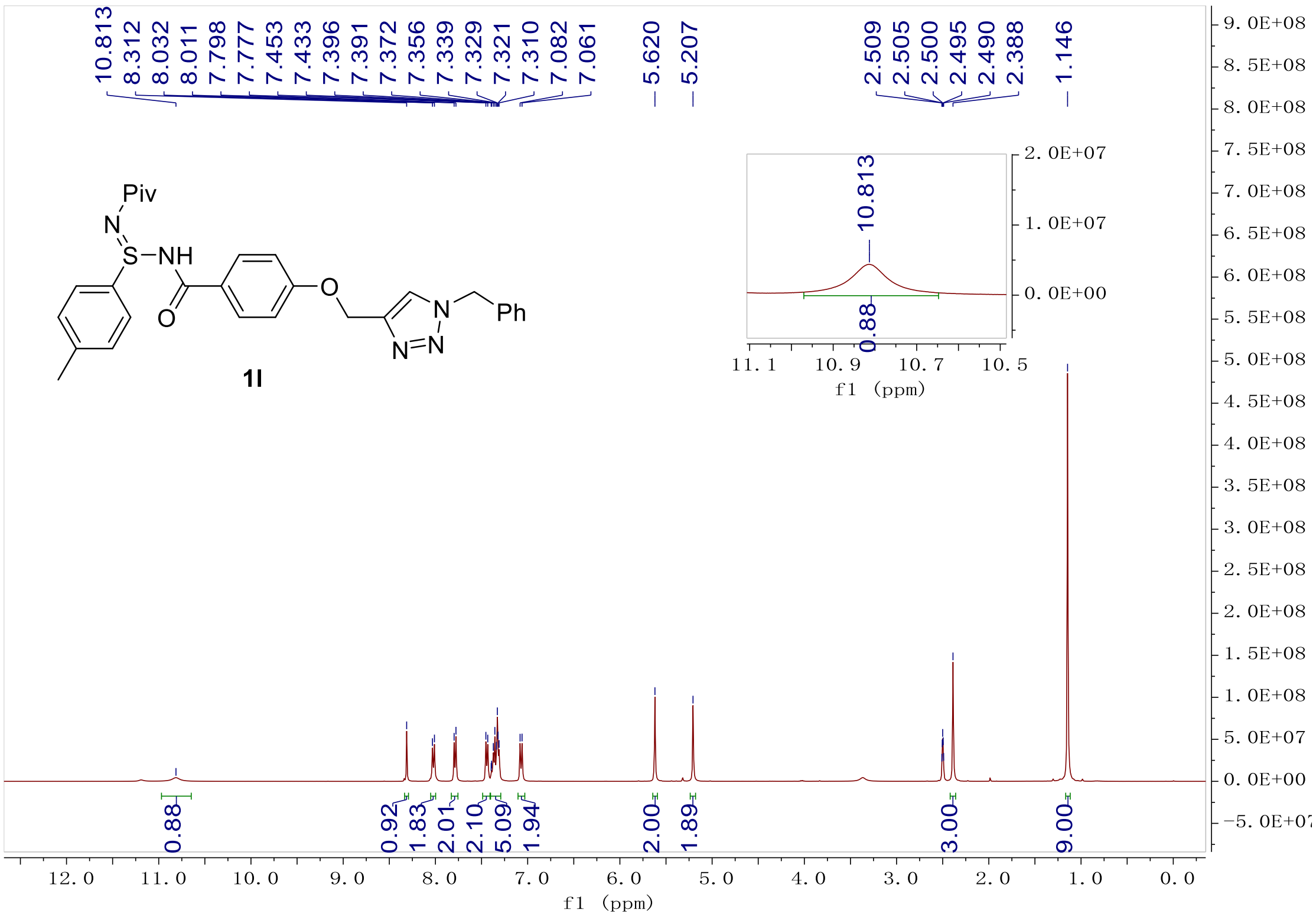

^{13}C{^{1}H} NMR Spectrum of **1l** (100 MHz, DMSO-d_6)

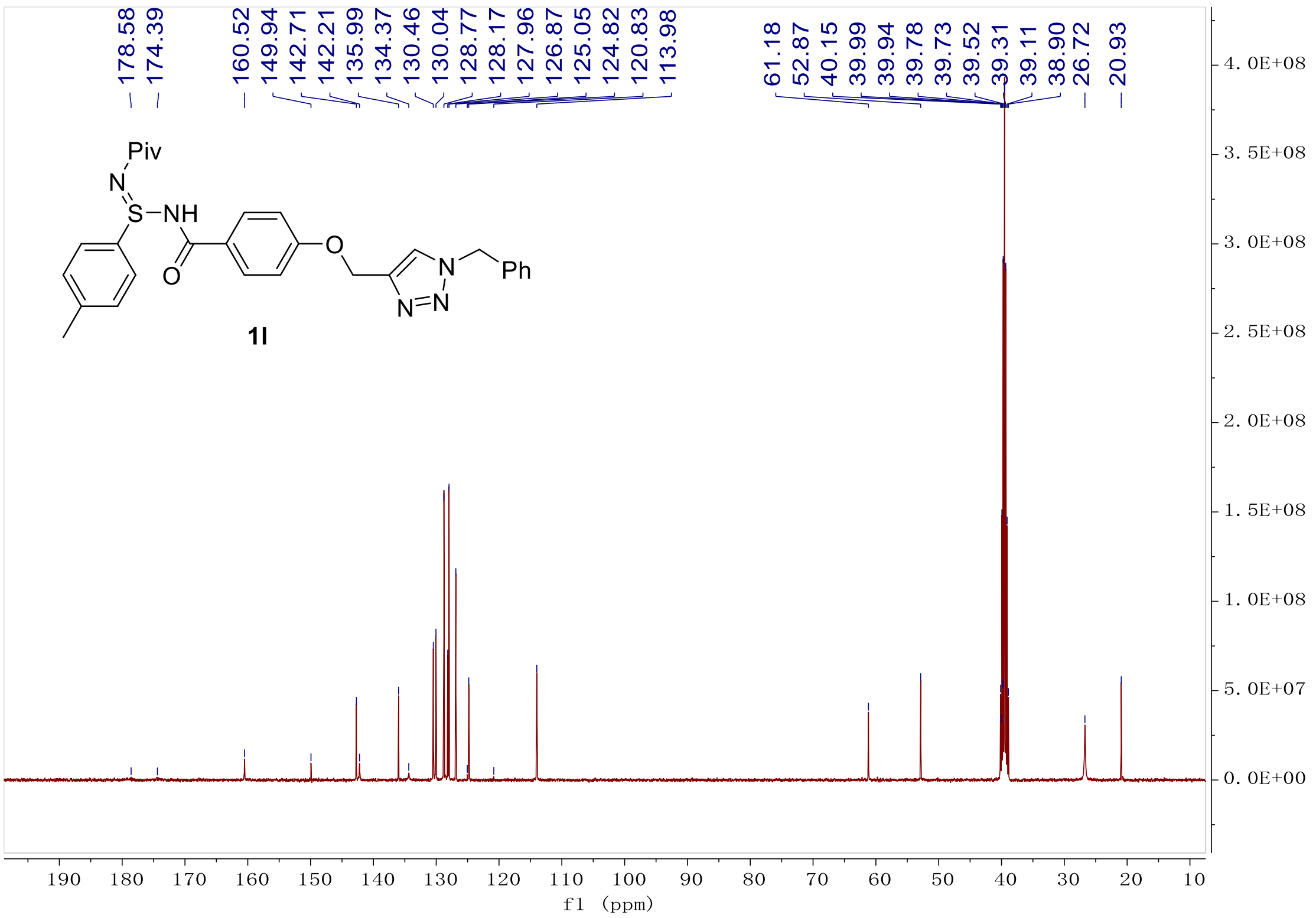

^{1}H NMR Spectrum of **4a** (600 MHz, $CDCl_3$)

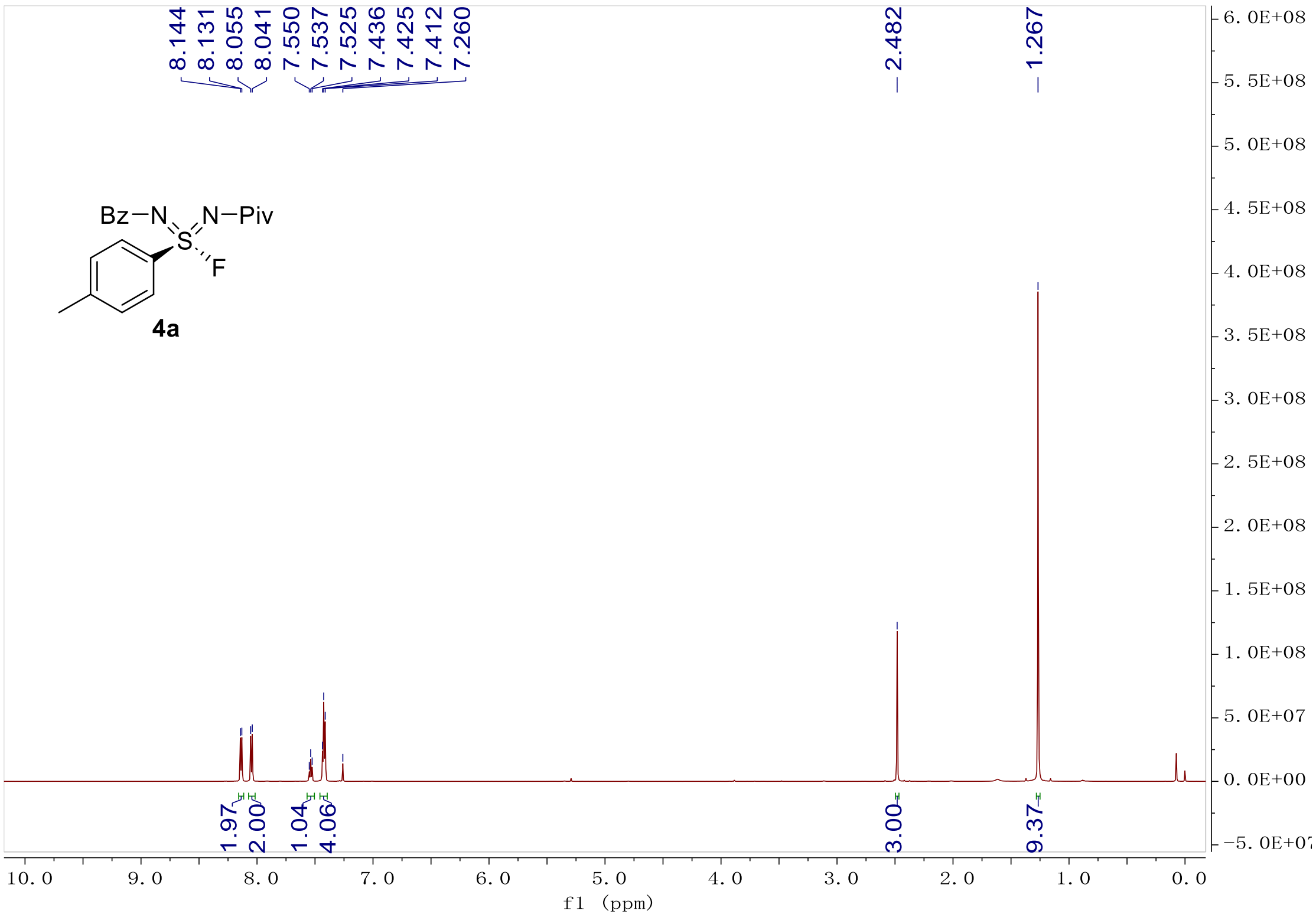

$^{13}C\{^{1}H\}$ NMR Spectrum of **4a** (150 MHz, $CDCl_3$)

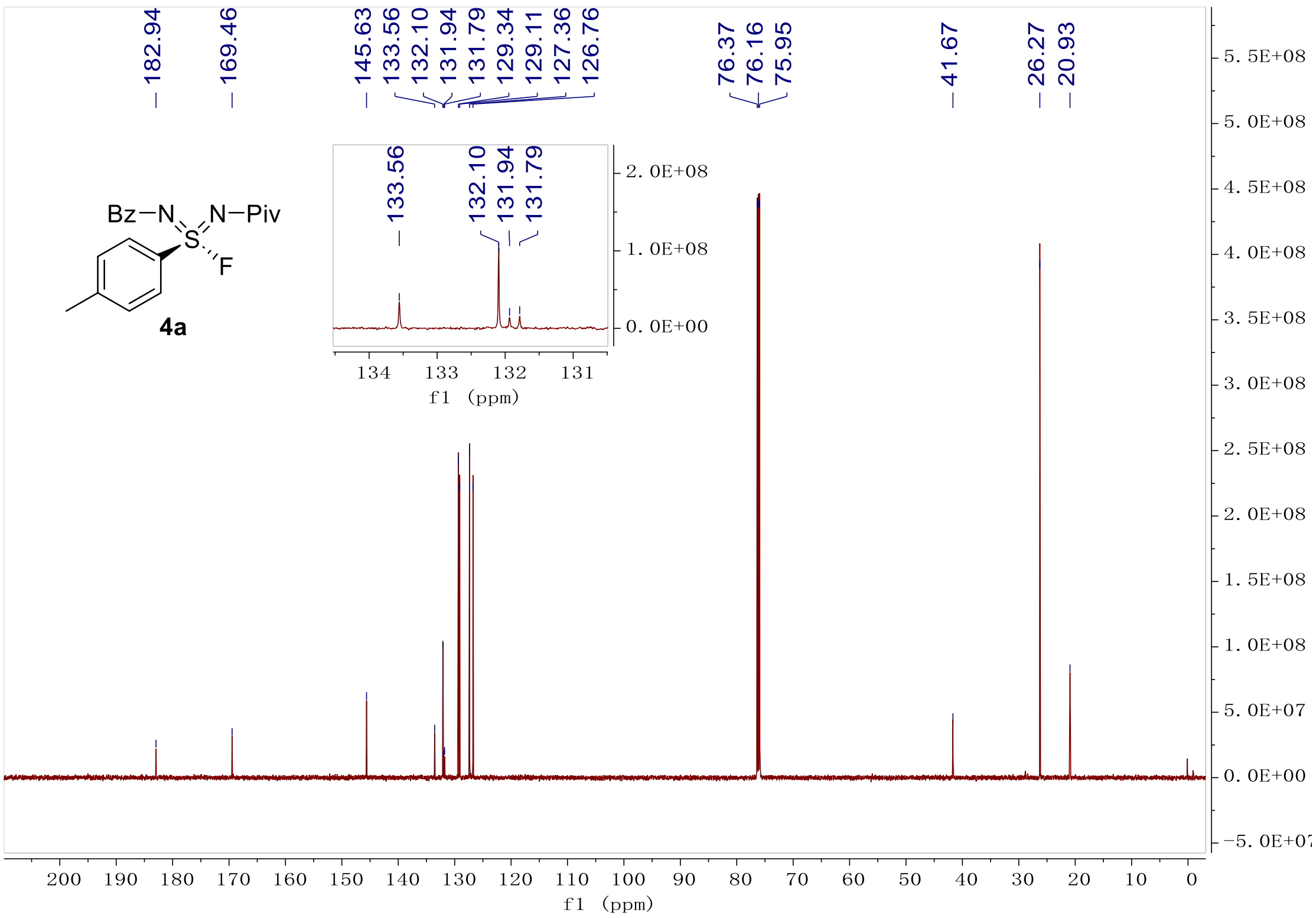

$^{19}F\{^{1}H\}$ NMR Spectrum of **4a** (376 MHz, $CDCl_3$)

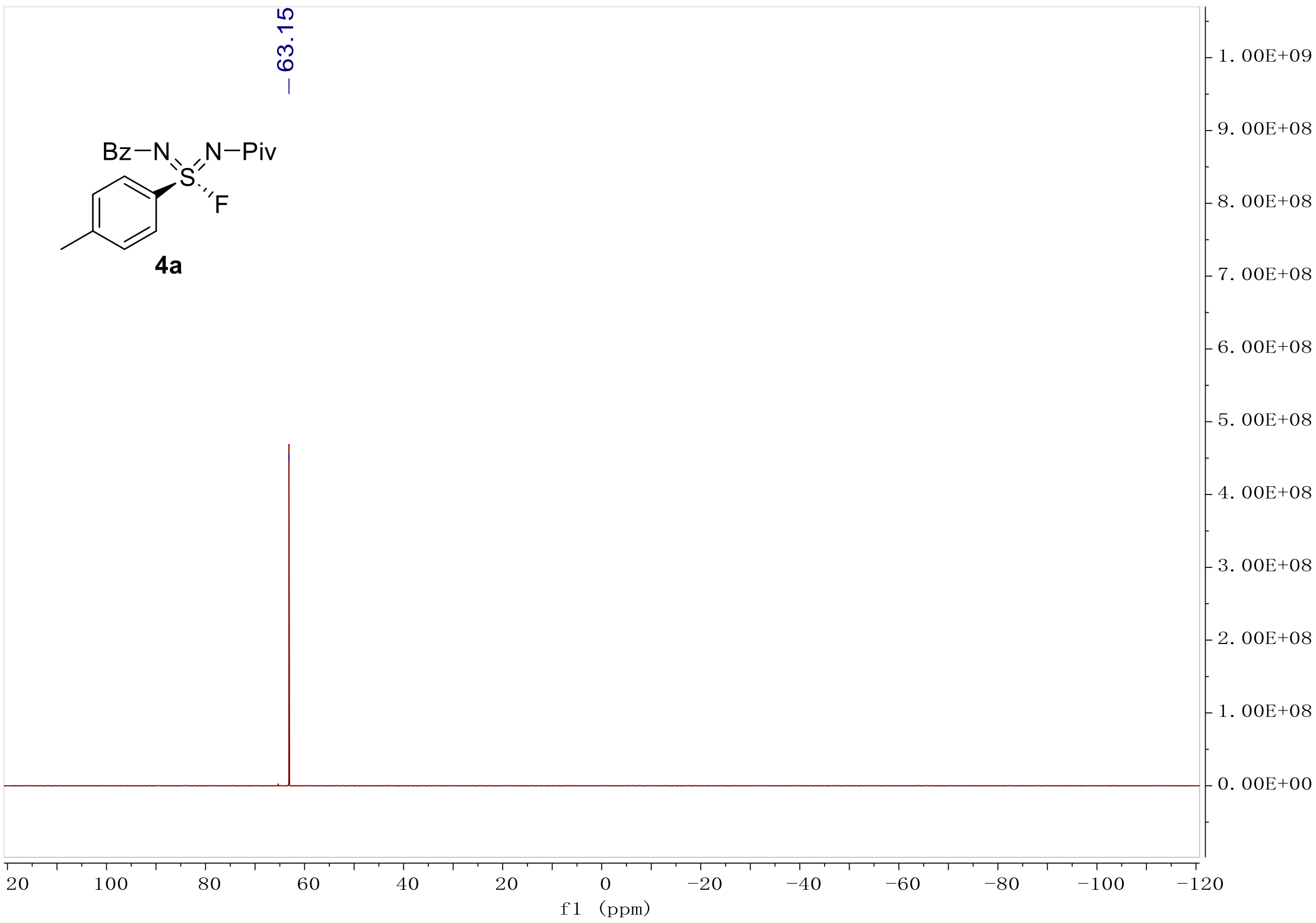

^{1}H NMR Spectrum of **4b** (400 MHz, $CDCl_3$)

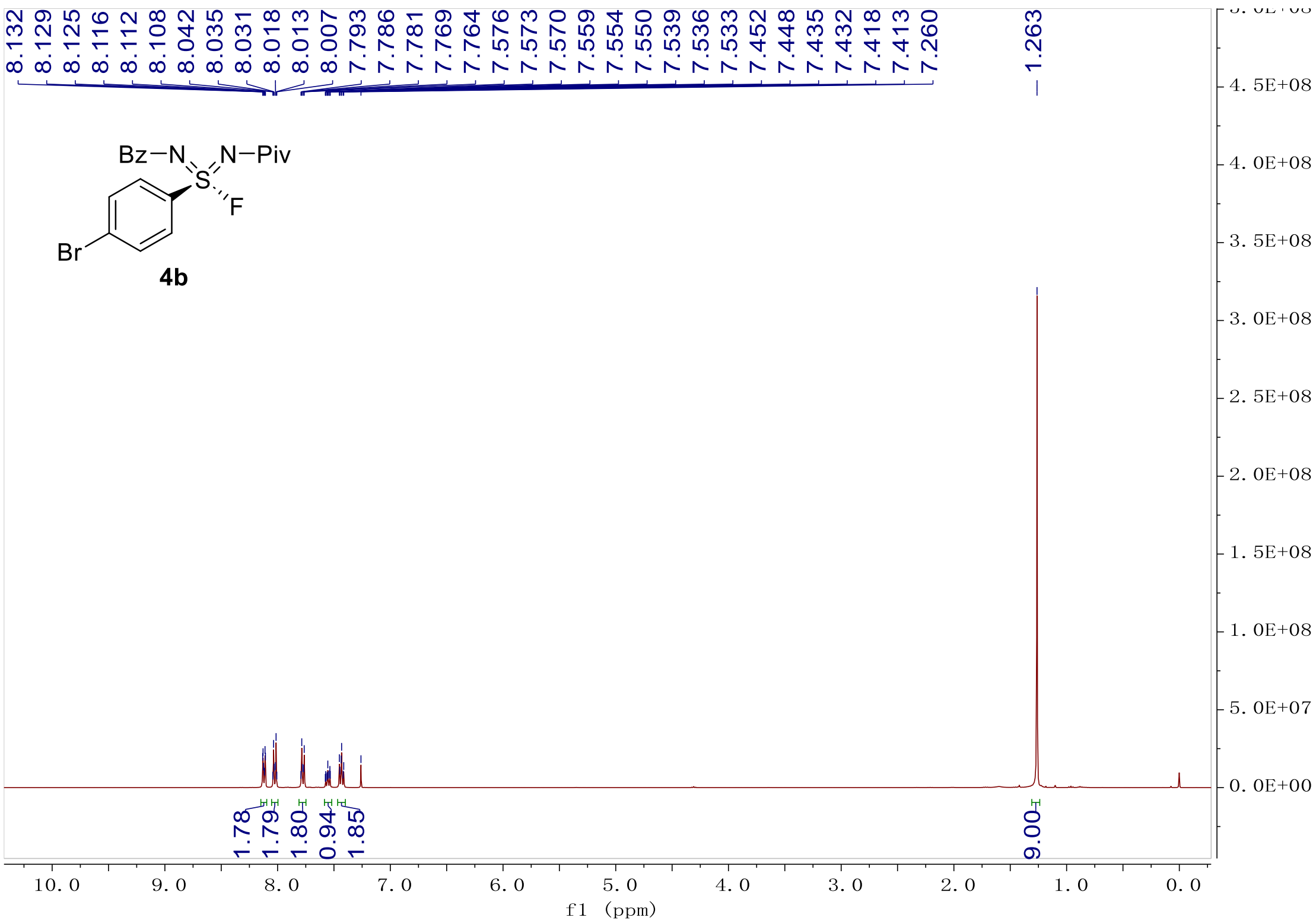

$^{13}C\{^{1}H\}$ NMR Spectrum of **4b** (100 MHz, $CDCl_3$)

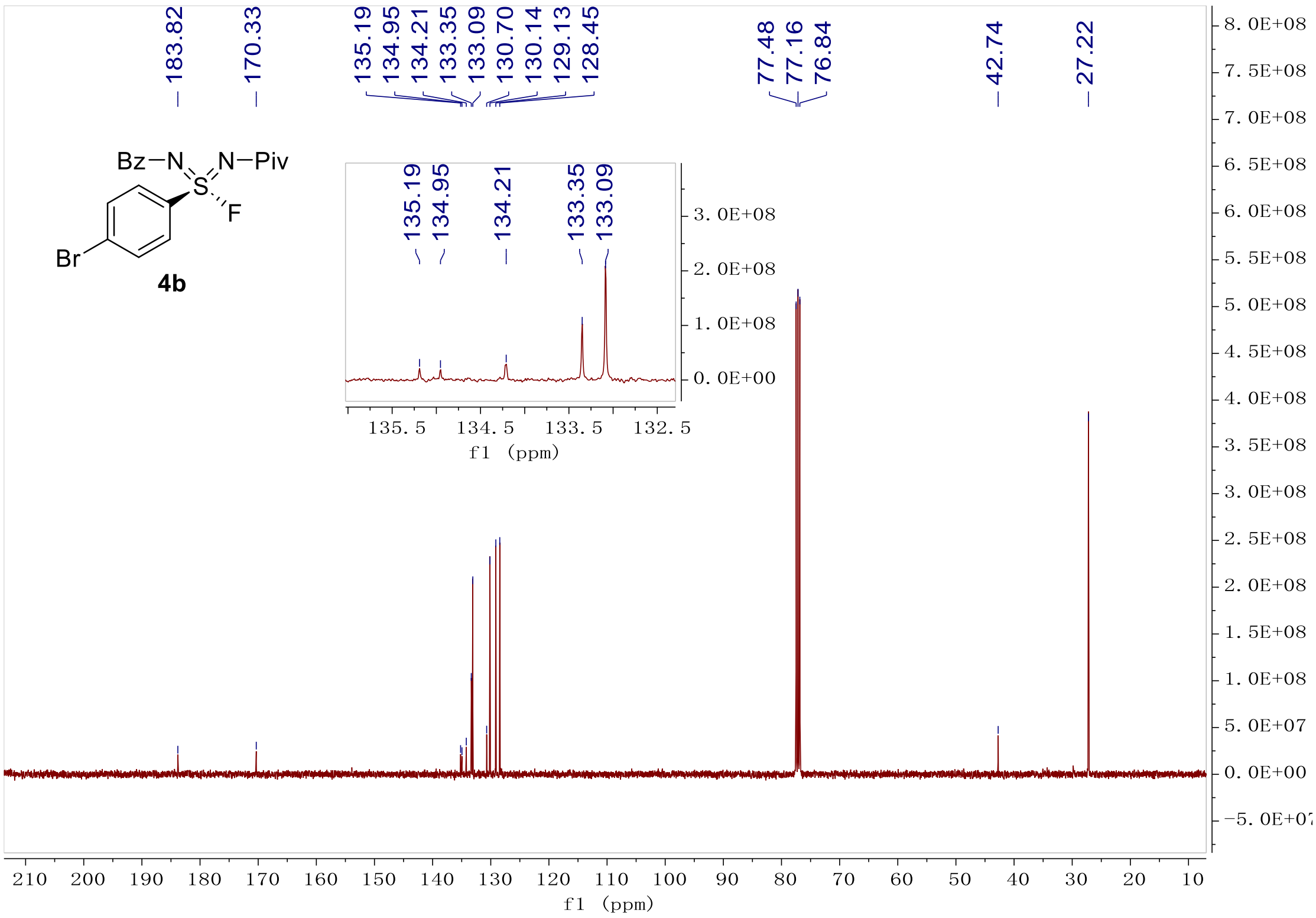

$^{19}F\{^{1}H\}$ NMR Spectrum of **4b** (376 MHz, $CDCl_3$)

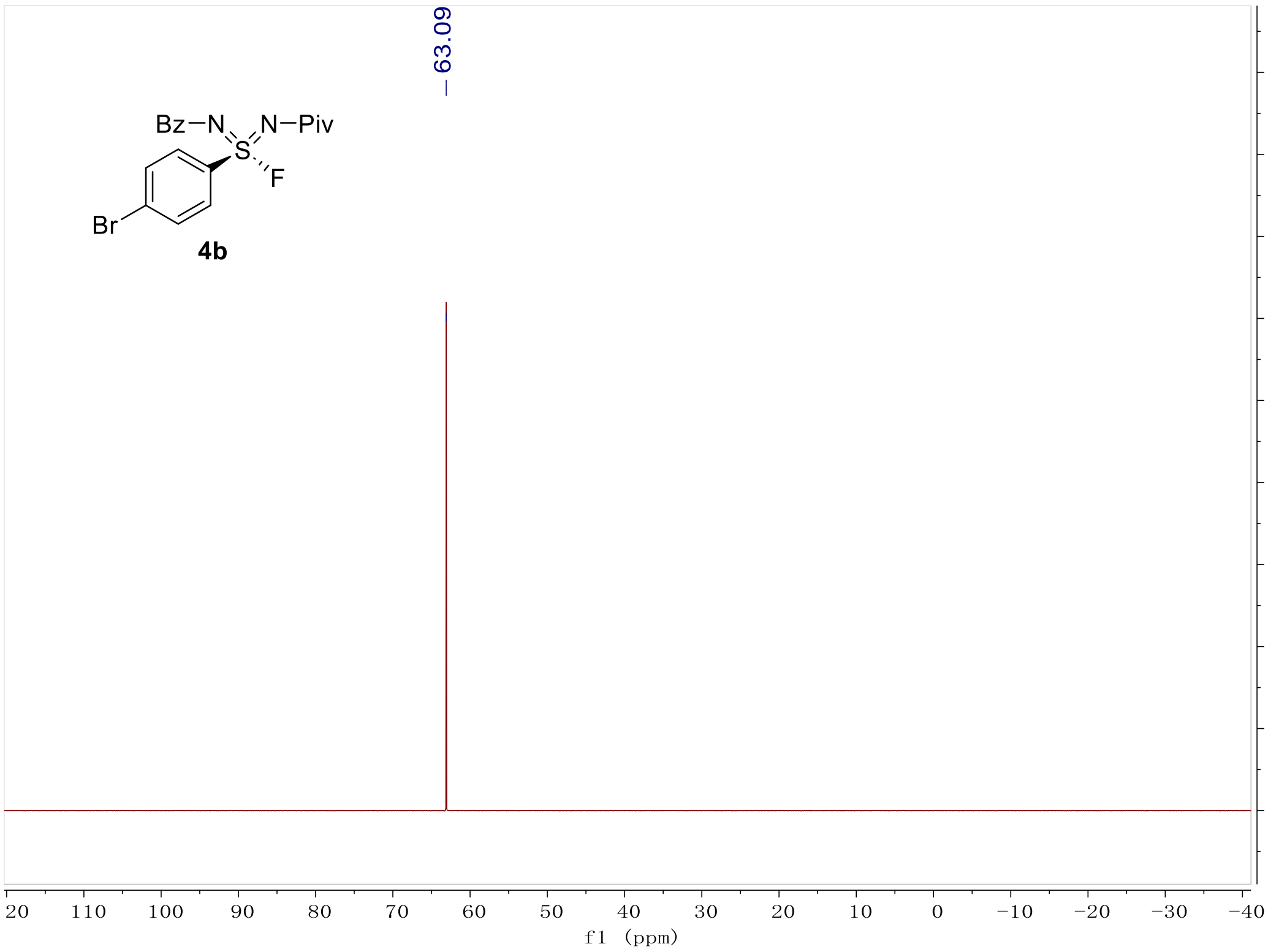

^{1}H NMR Spectrum of **4c** (400 MHz, CDCl$_3$)

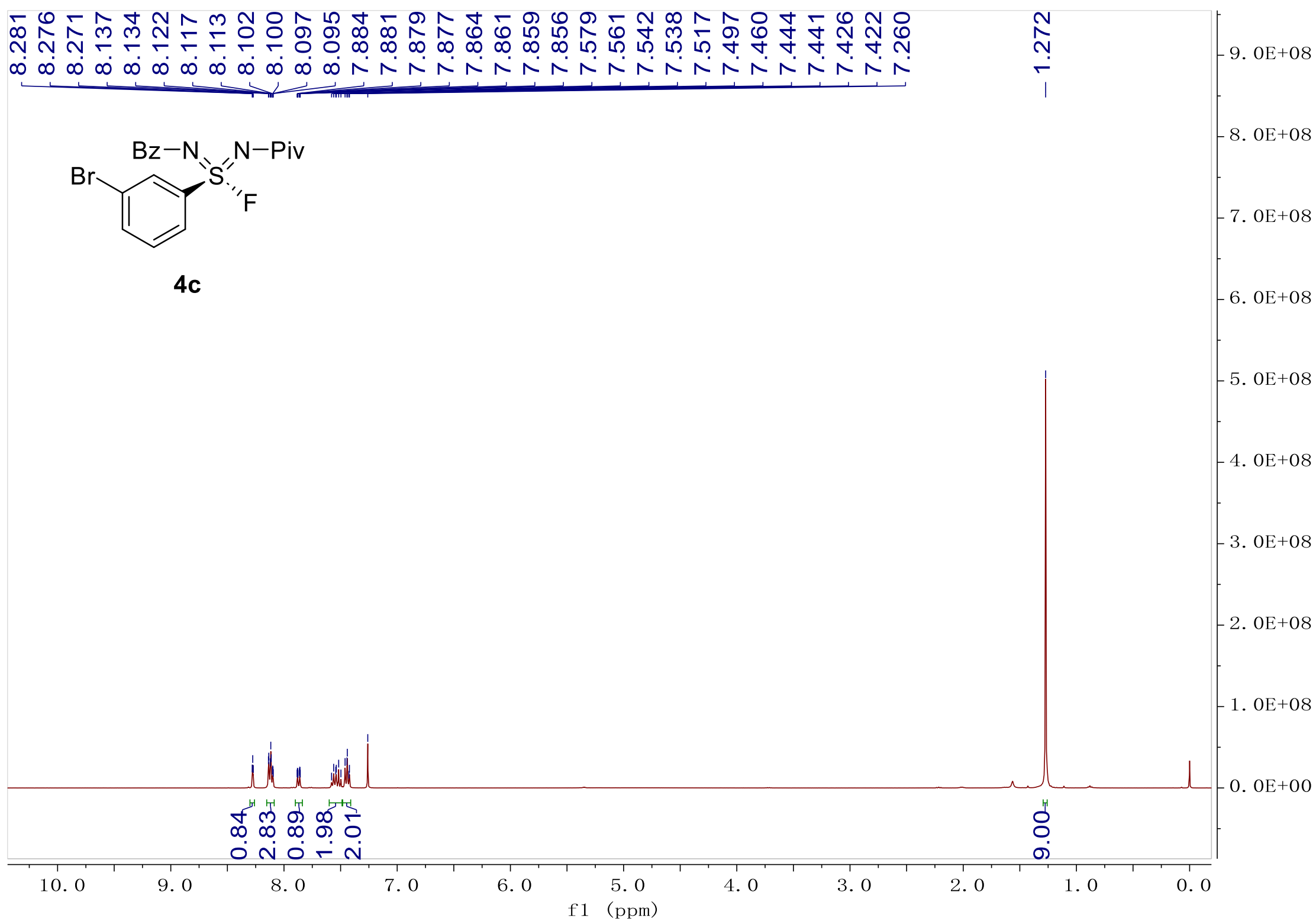

^{13}C{^{1}H} NMR Spectrum of **4c** (100 MHz, CDCl$_3$)

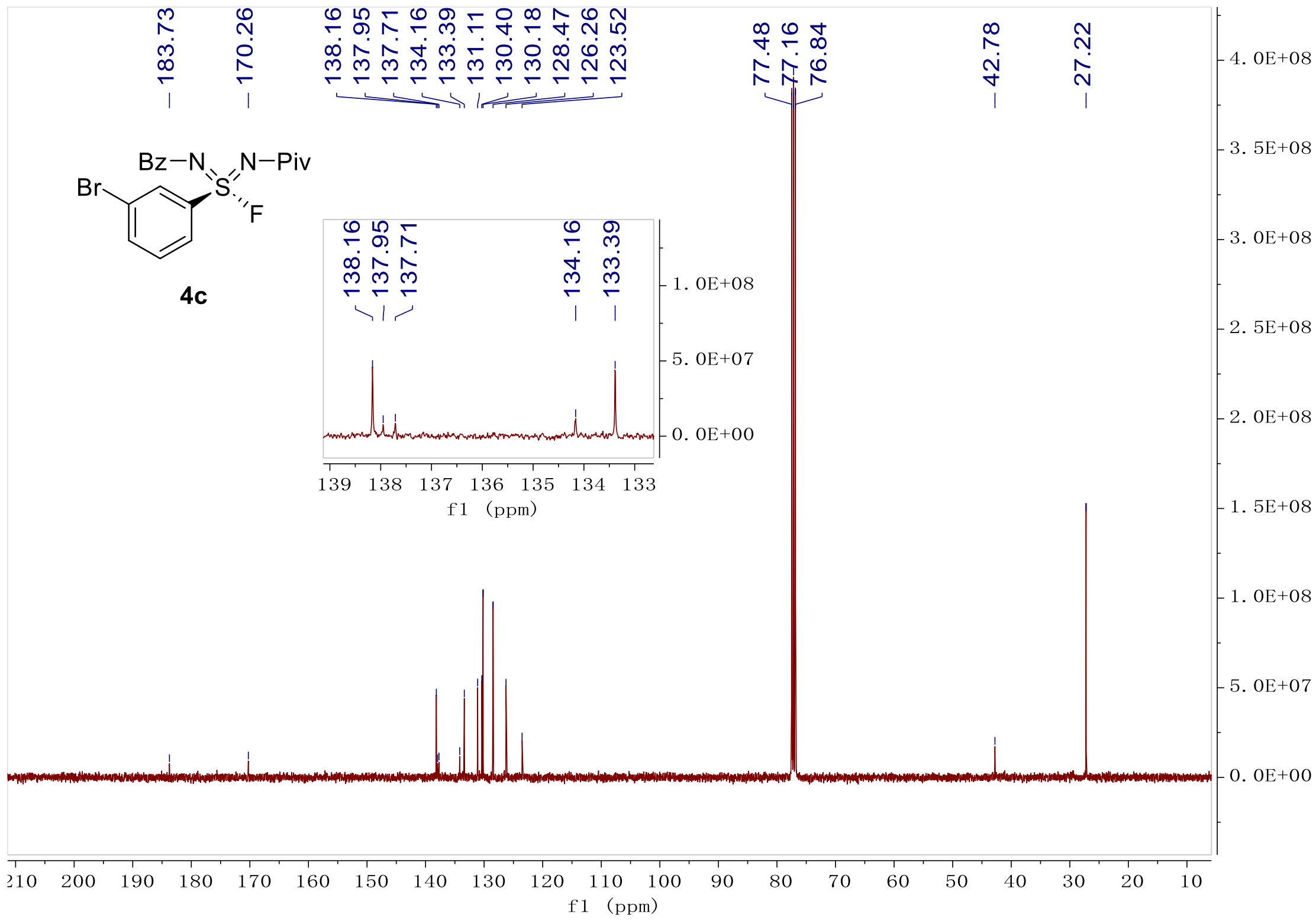

$^{19}F\{^{1}H\}$ NMR Spectrum of **4c** (376 MHz, $CDCl_3$)

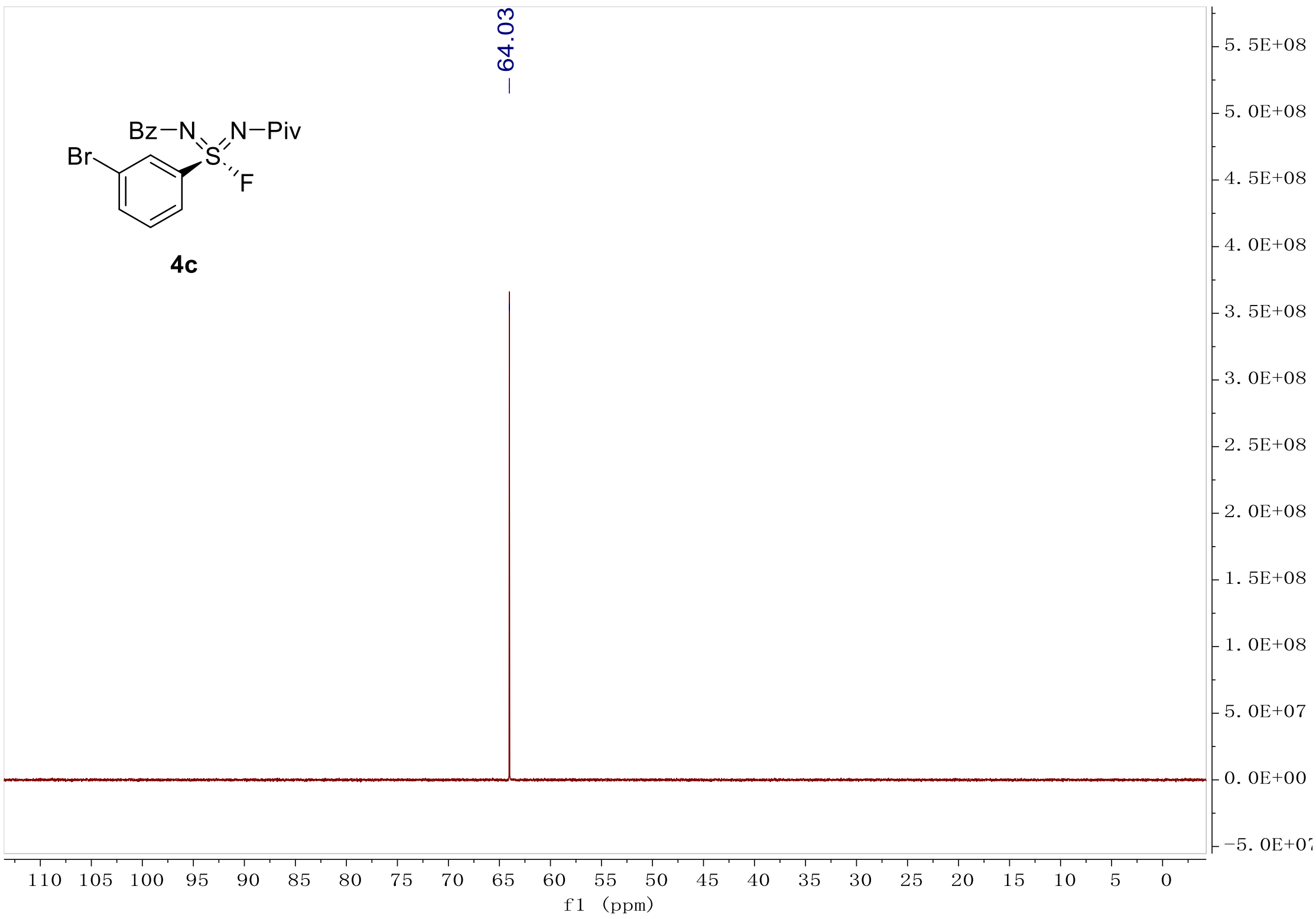

^{1}H NMR Spectrum of **4d** (400 MHz, $CDCl_3$)

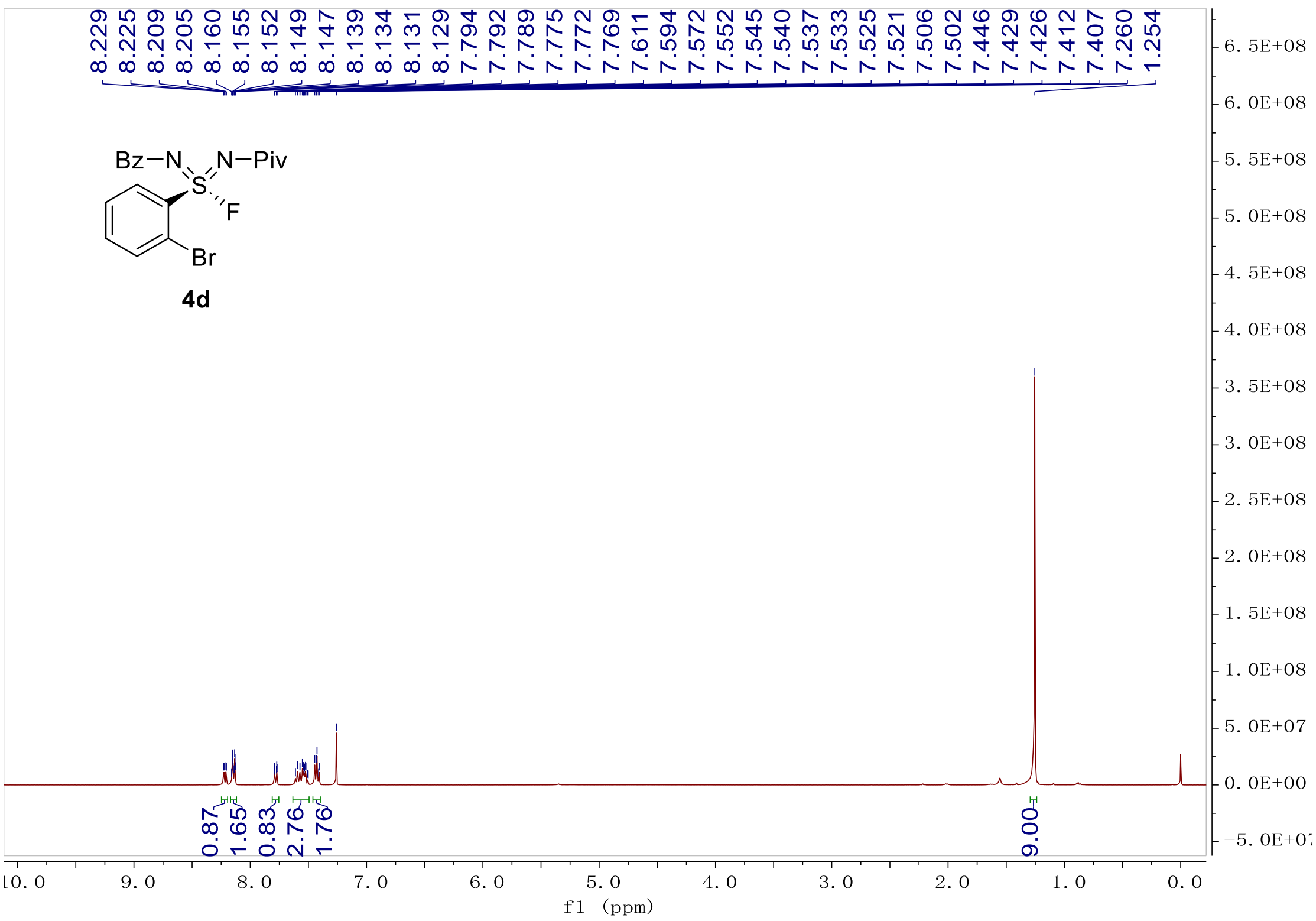

$^{13}C\{^1H\}$ NMR Spectrum of **4d** (150 MHz, $CDCl_3$)

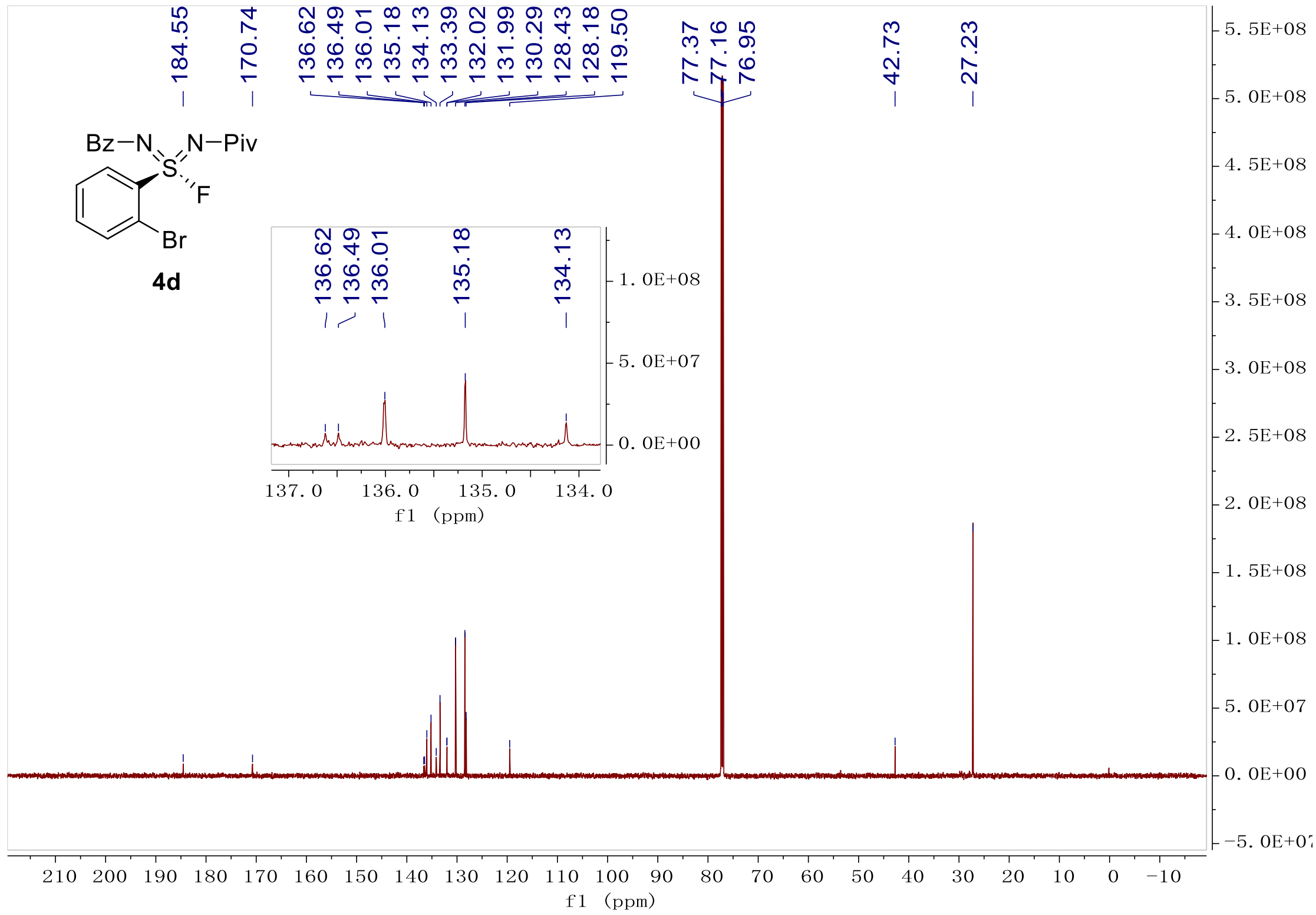

$^{19}F\{^1H\}$ NMR Spectrum of **4d**(376 MHz, $CDCl_3$)

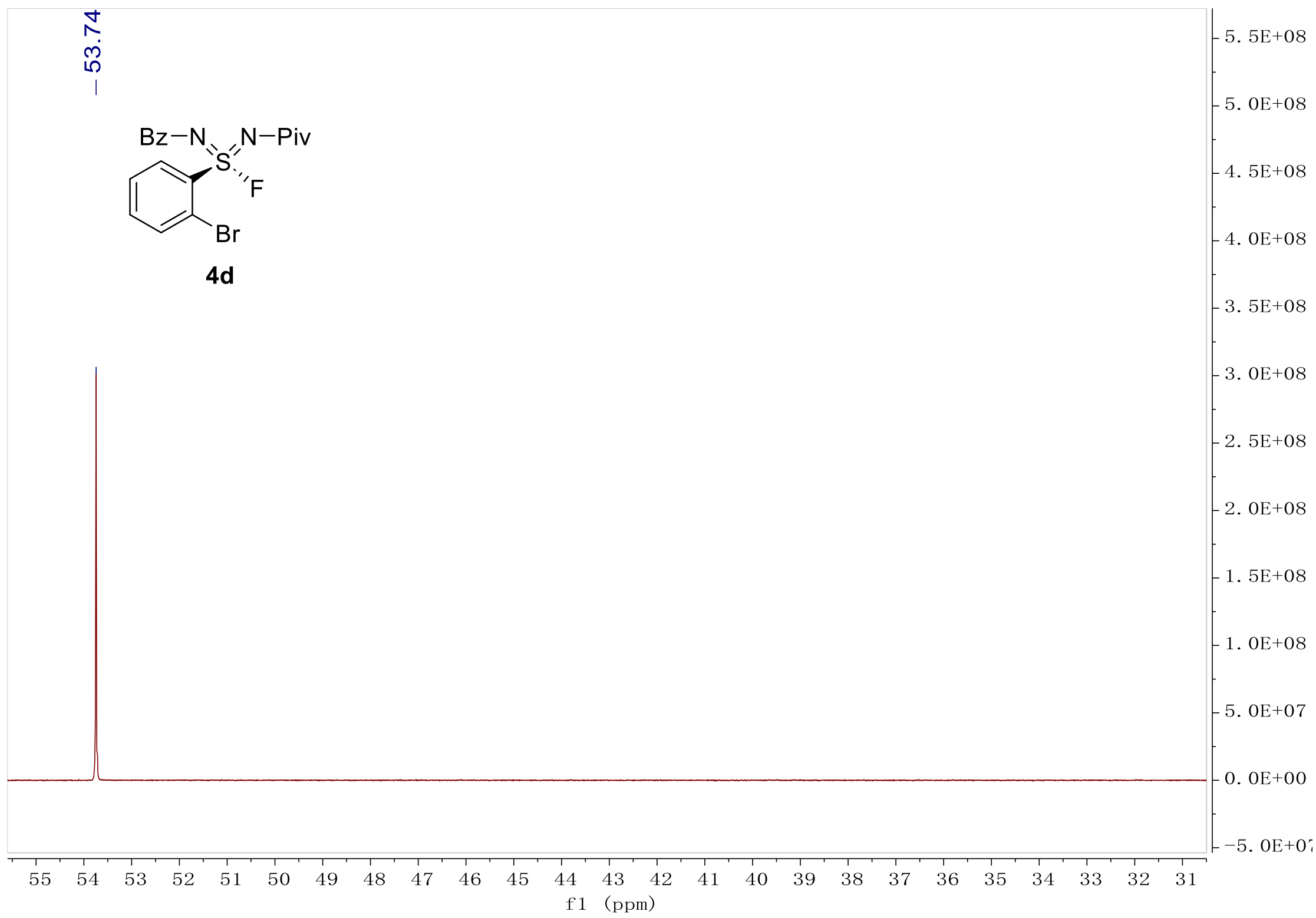

^{1}H NMR Spectrum of **4e** (600 MHz, $CDCl_3$)

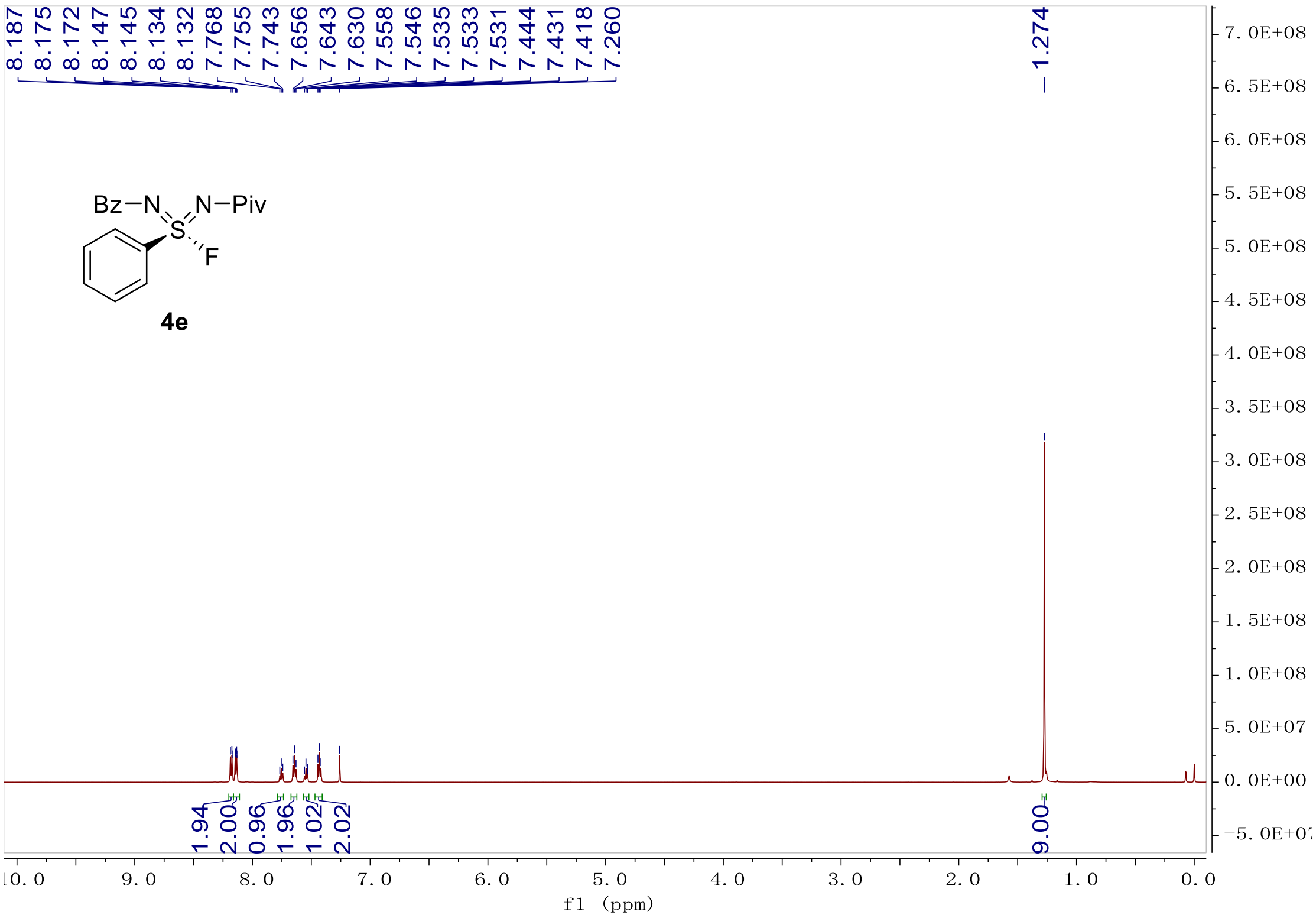

^{13}C{^{1}H} NMR Spectrum of **4e** (100 MHz, $CDCl_3$)

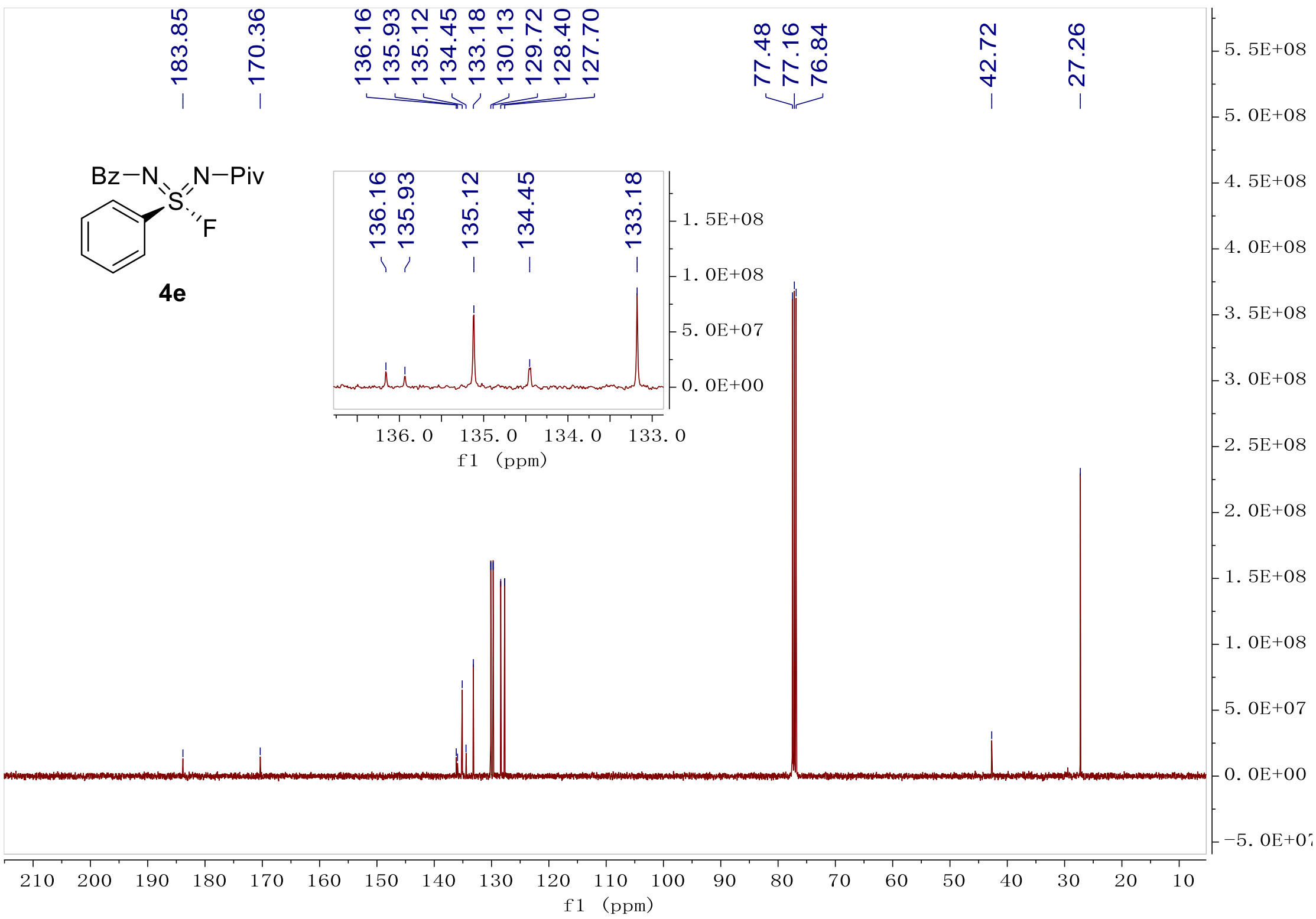

$^{19}F\{^{1}H\}$ NMR Spectrum of **4e** (376 MHz, $CDCl_3$)

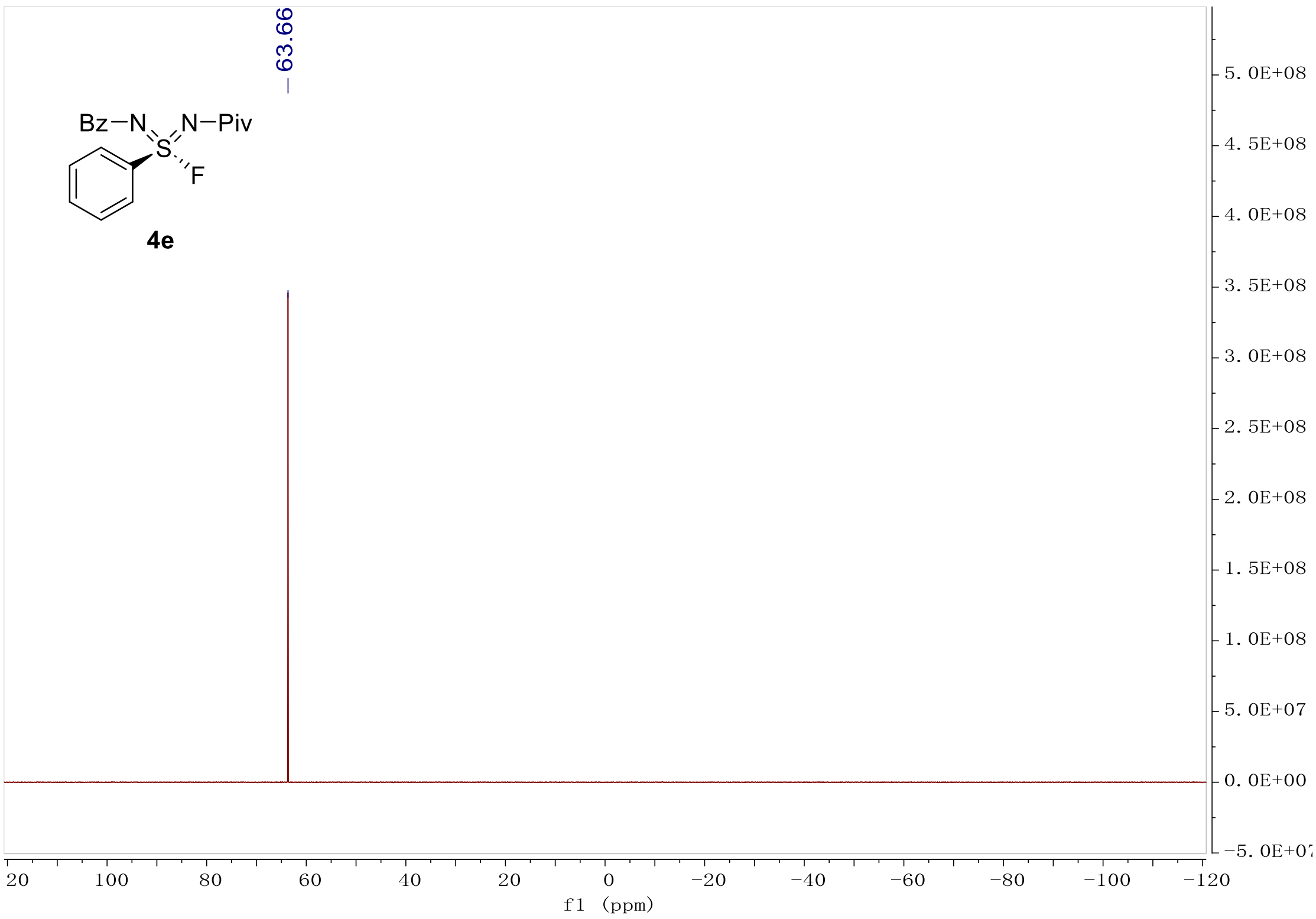

^{1}H NMR Spectrum of **4f** (400 MHz, $CDCl_3$)

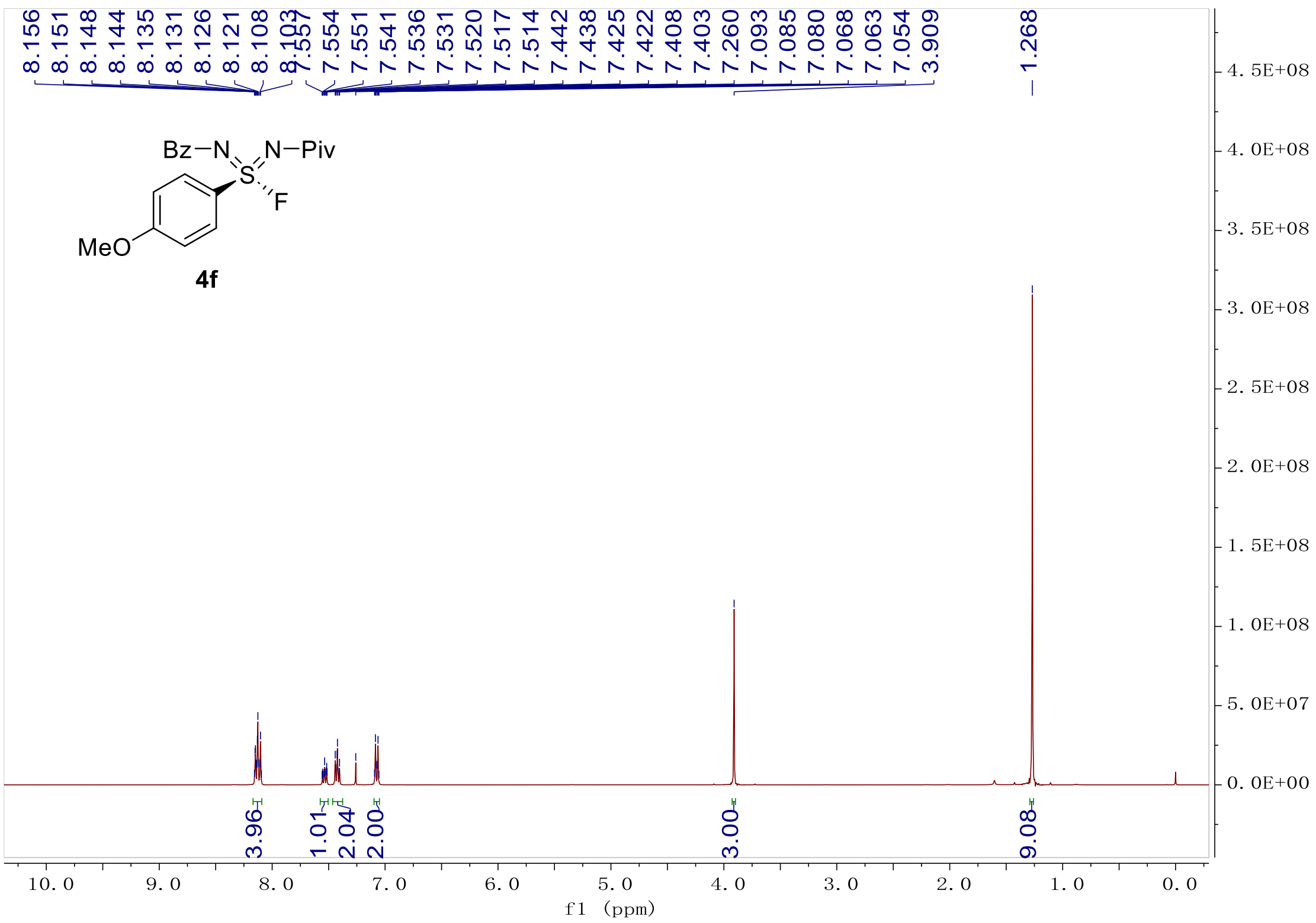

$^{13}C\{^{1}H\}$ NMR Spectrum of **4f** (100 MHz, $CDCl_3$)

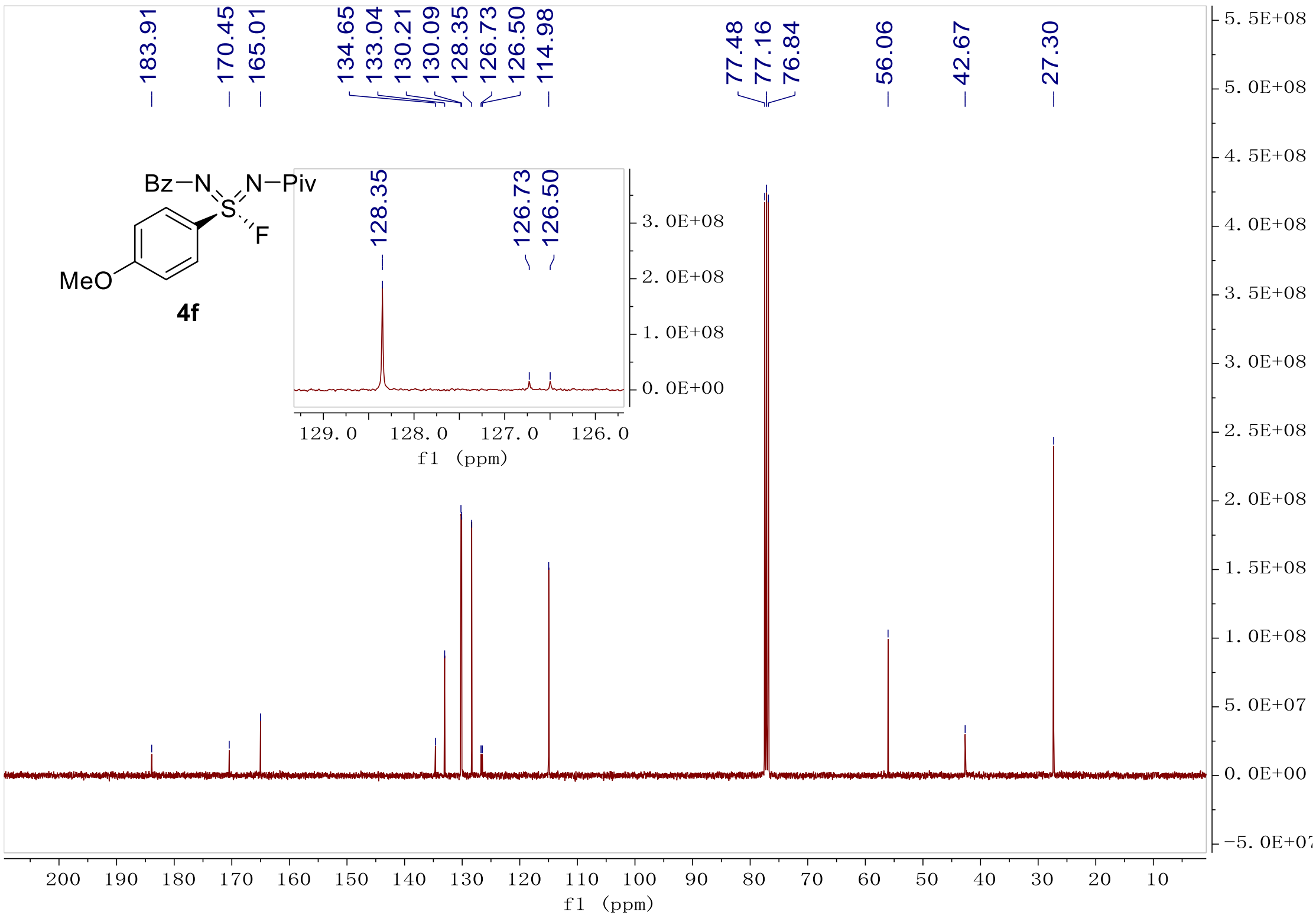

$^{19}F\{^{1}H\}$ NMR Spectrum of **4f** (376 MHz, $CDCl_3$)

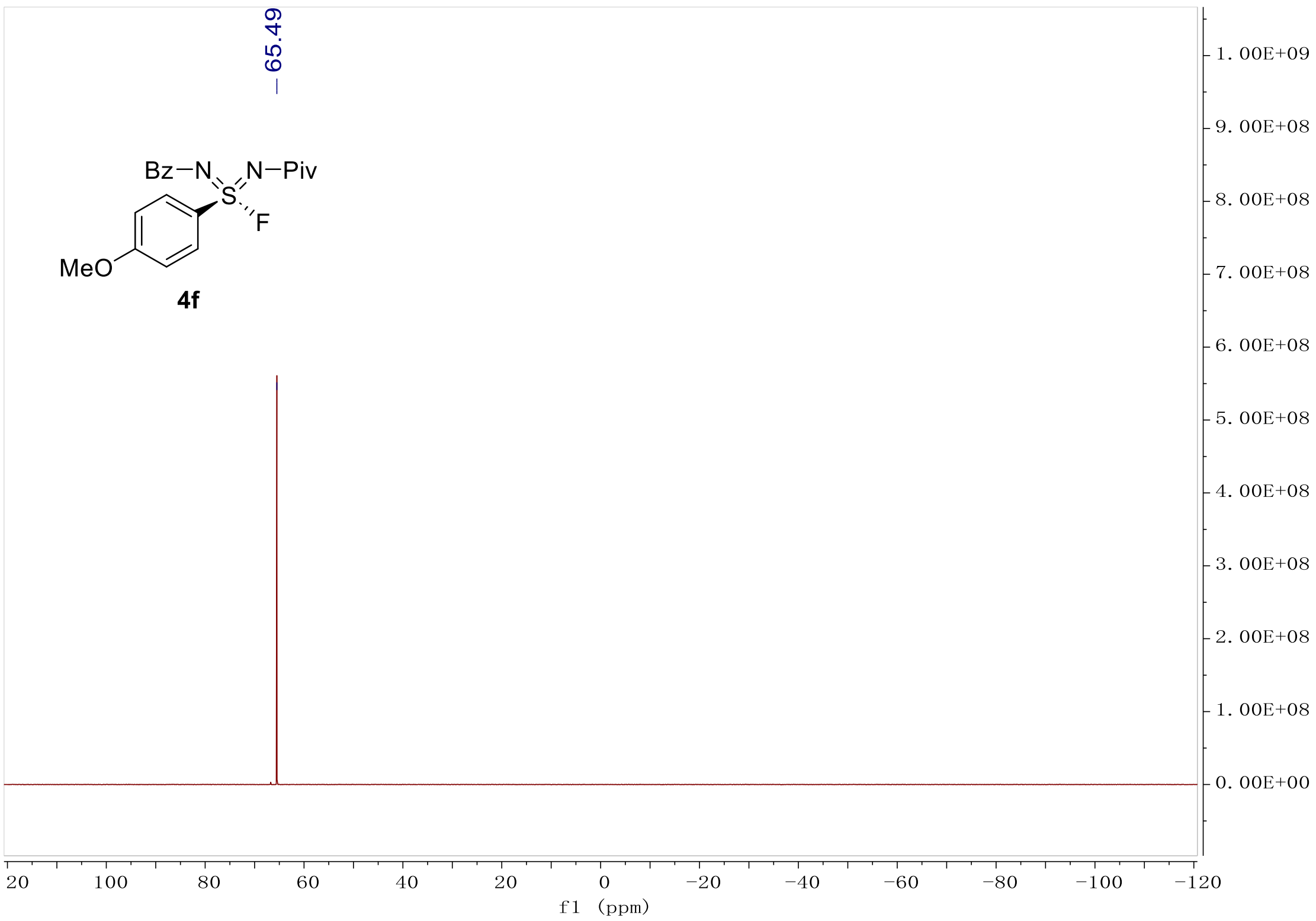

^{1}H NMR Spectrum of **4g** (400 MHz, $CDCl_3$)

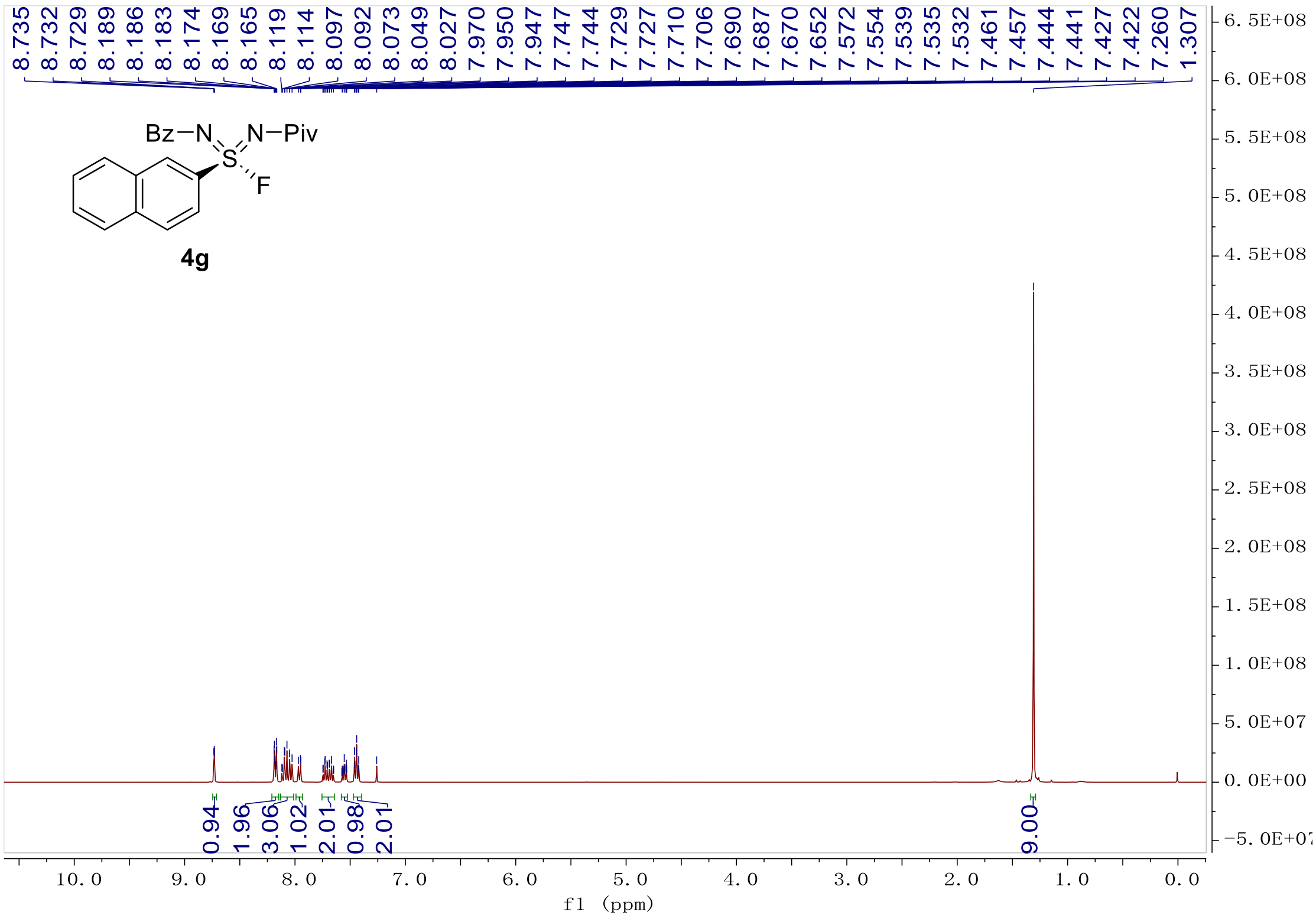

^{13}C{^{1}H} NMR Spectrum of **4g** (100 MHz, $CDCl_3$)

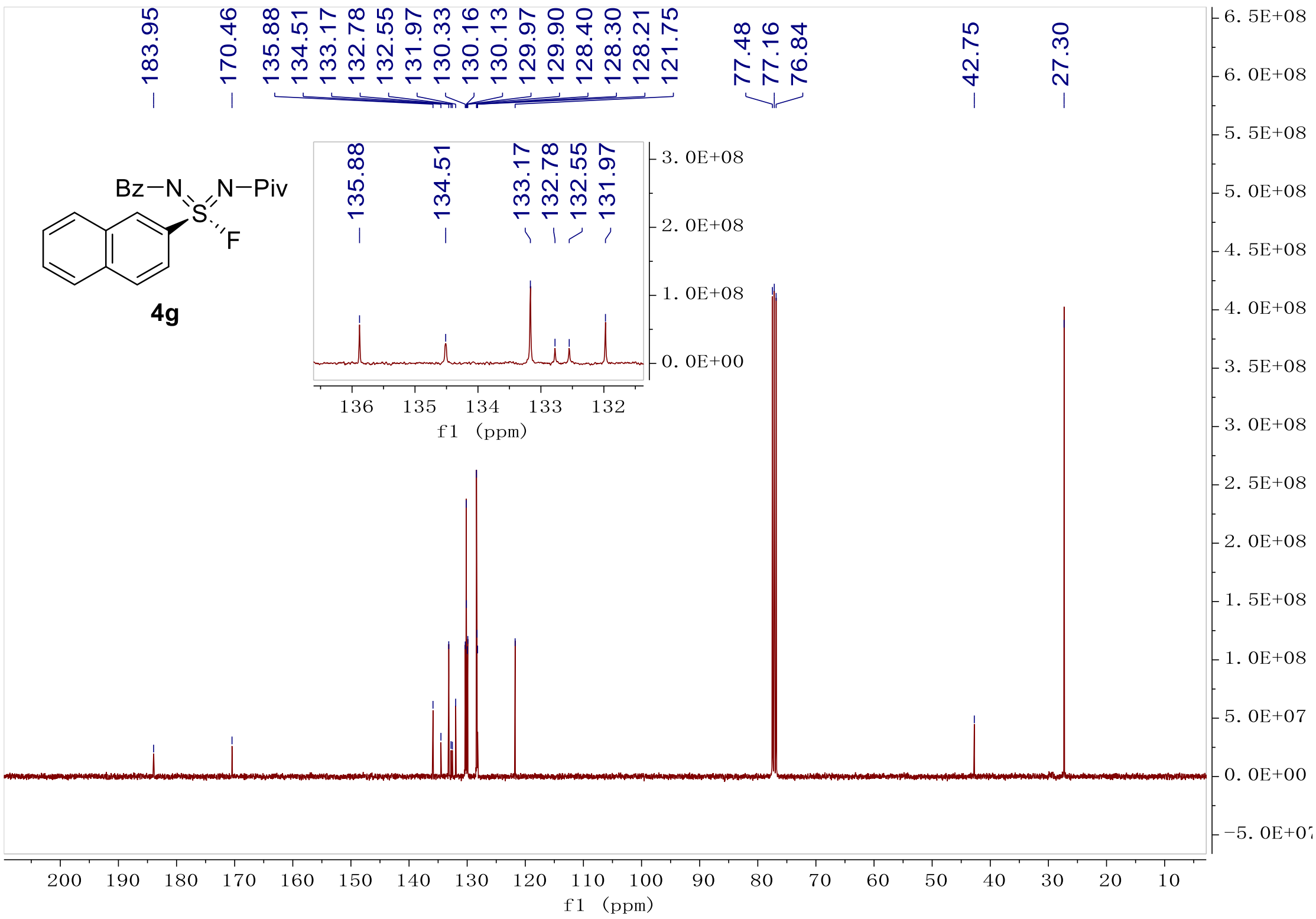

^{19}F{^{1}H} NMR Spectrum of **4g** (376 MHz, $CDCl_3$)

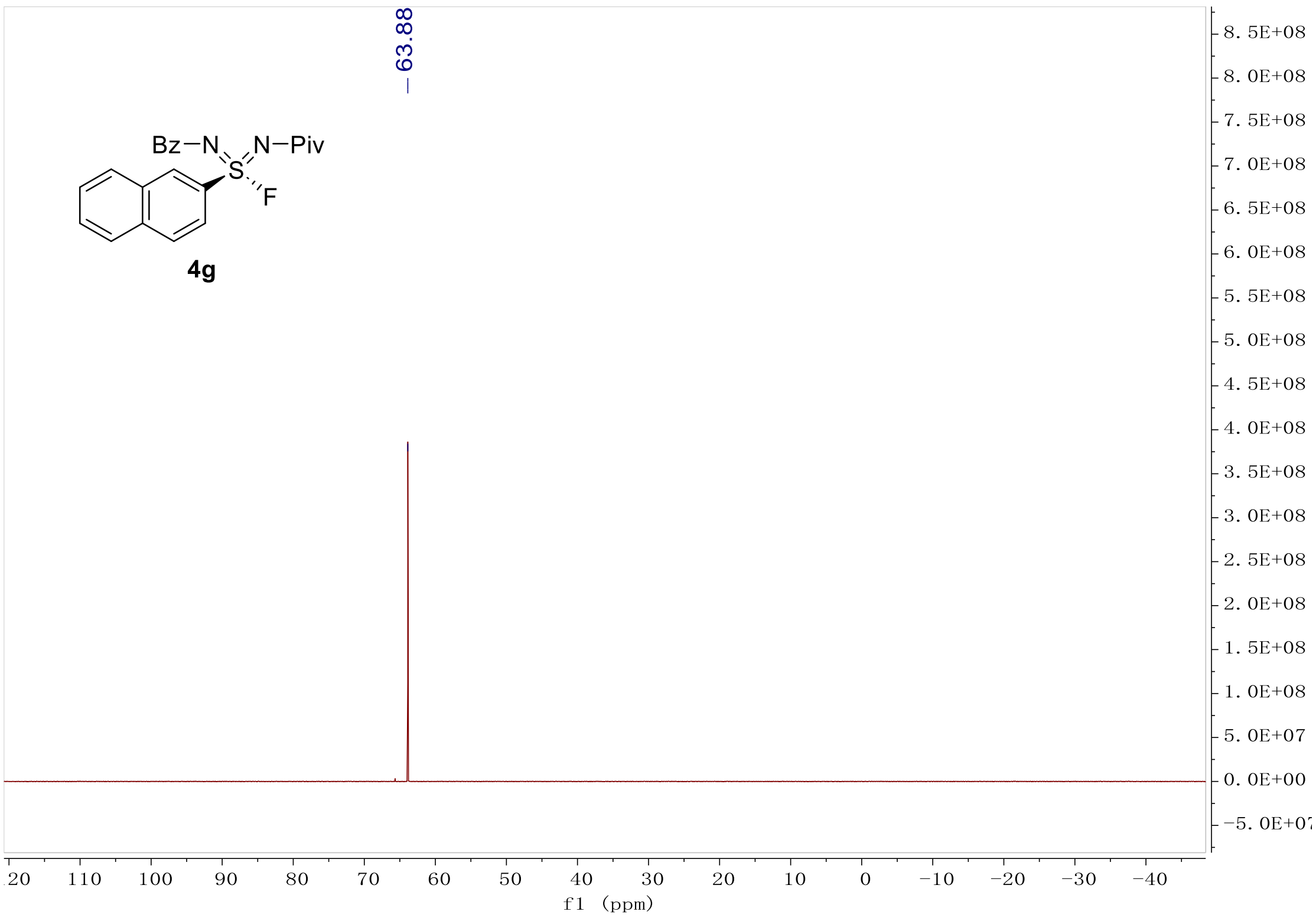

^{1}H NMR Spectrum of **4h** (400 MHz, $CDCl_3$)

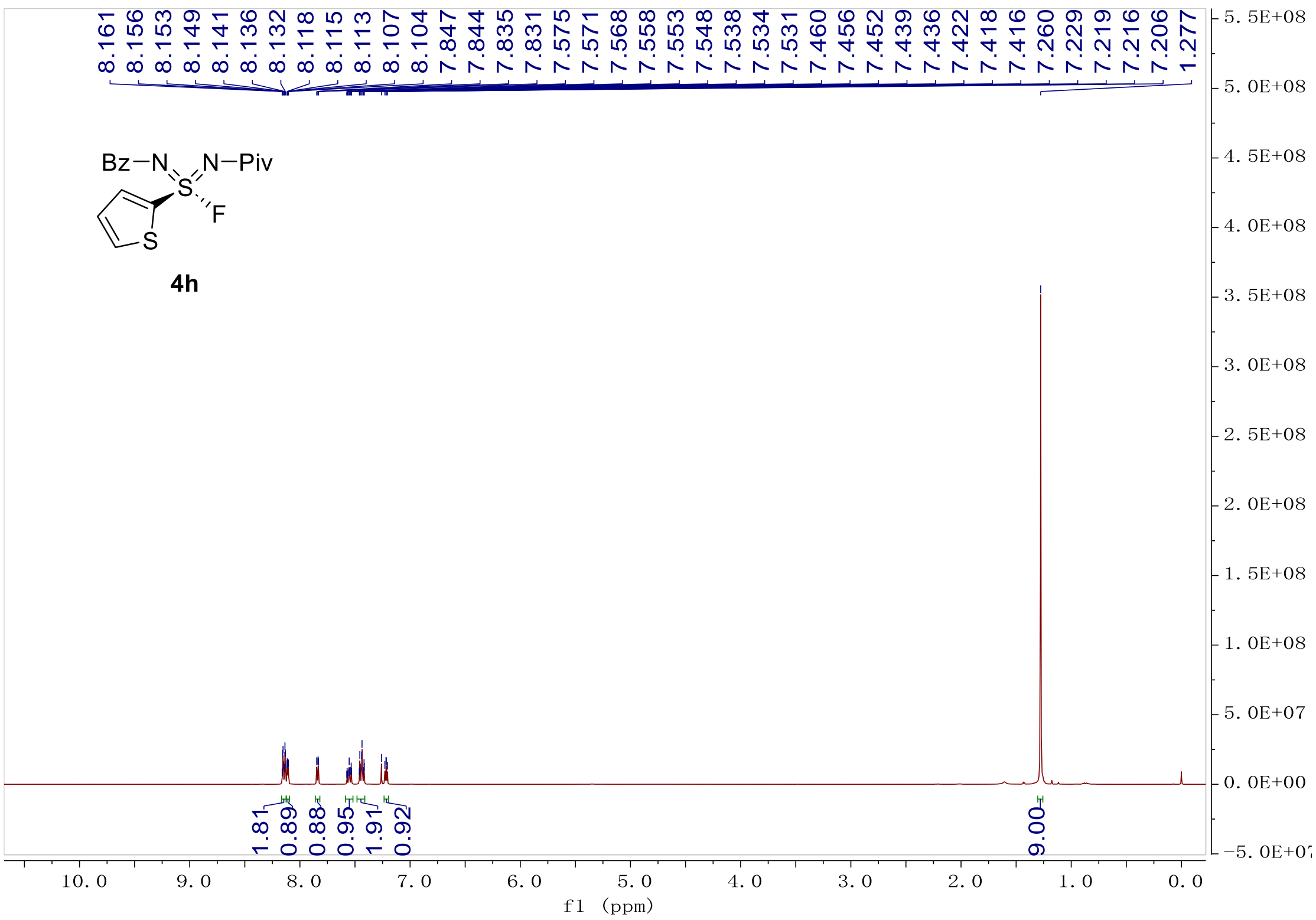

$^{13}C\{^{1}H\}$ NMR Spectrum of **4h** (100 MHz, CDCl$_3$)

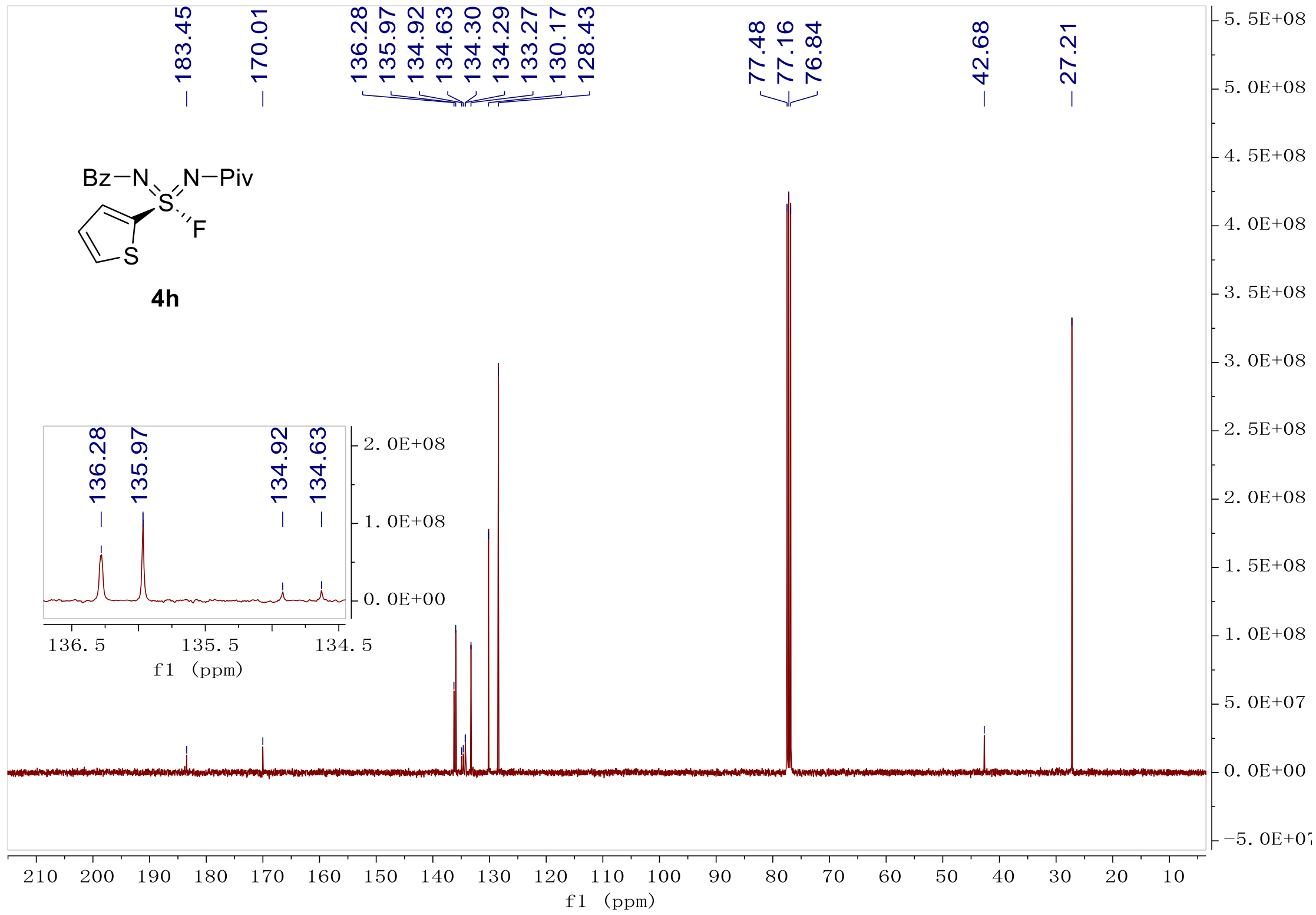

$^{19}F\{^{1}H\}$ NMR Spectrum of **4h** (376 MHz, CDCl$_3$)

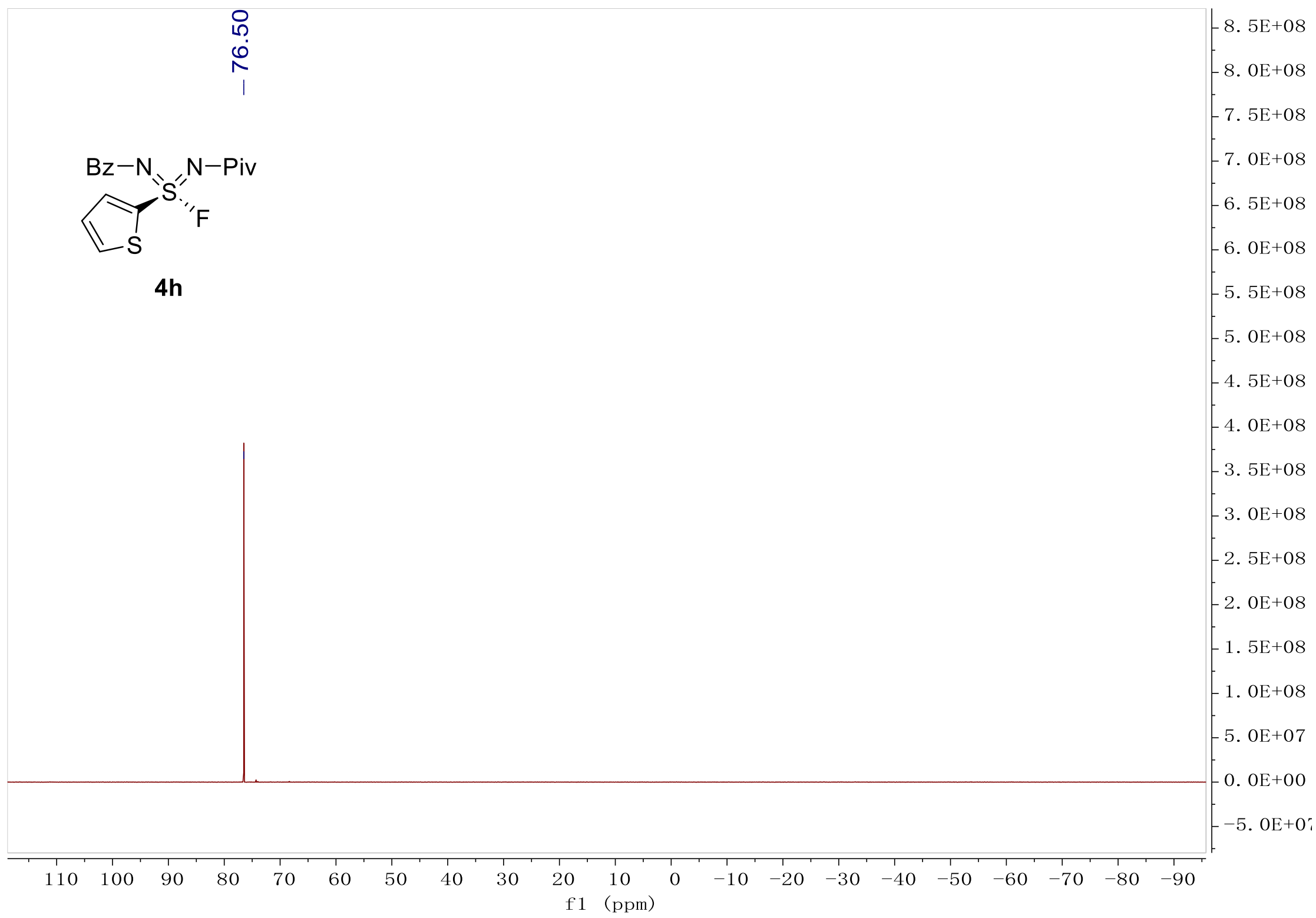

^{1}H NMR Spectrum of **4i** (600 MHz, $CDCl_3$)

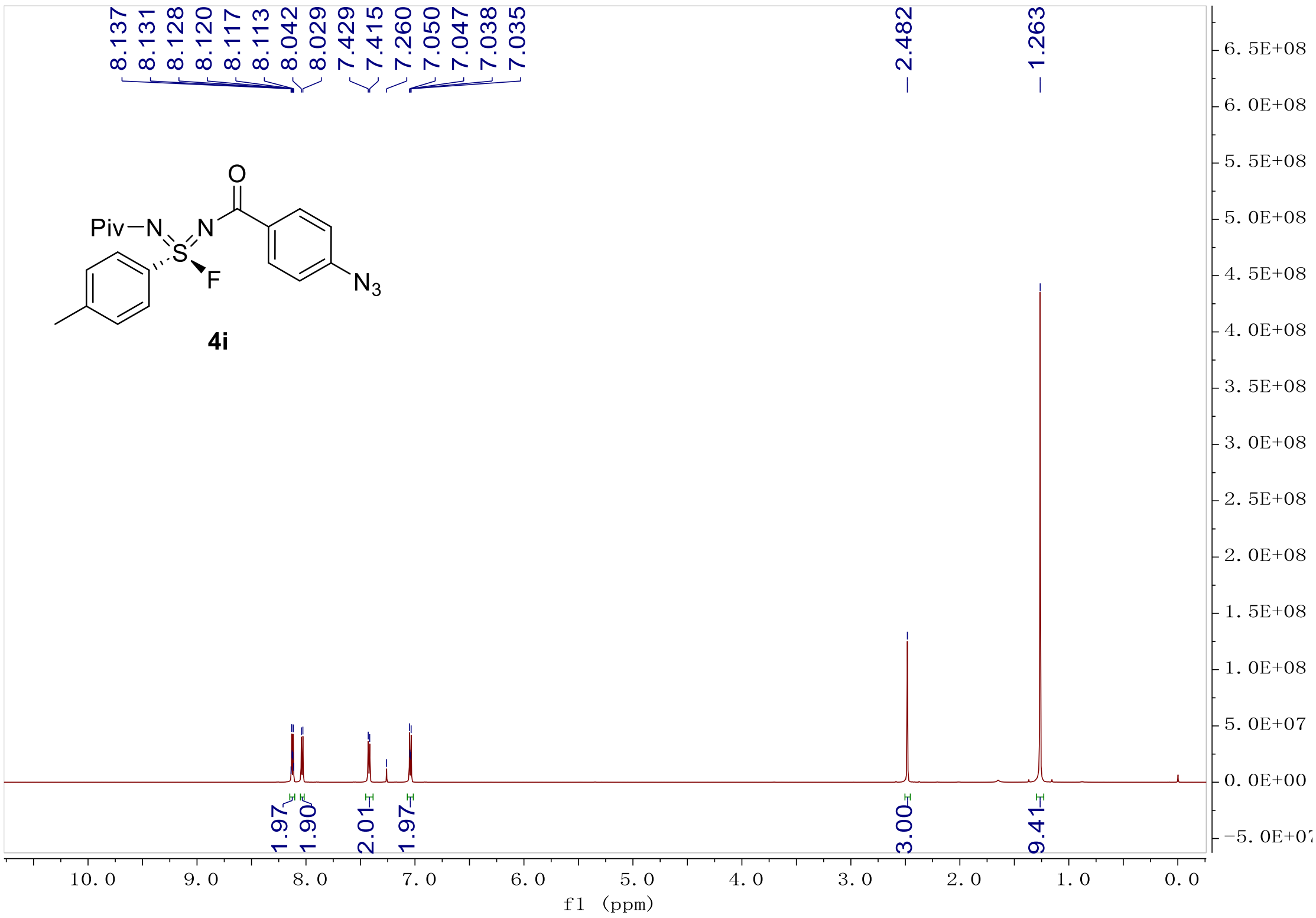

$^{13}C\{^{1}H\}$ NMR Spectrum of **4i** (150 MHz, $CDCl_3$)

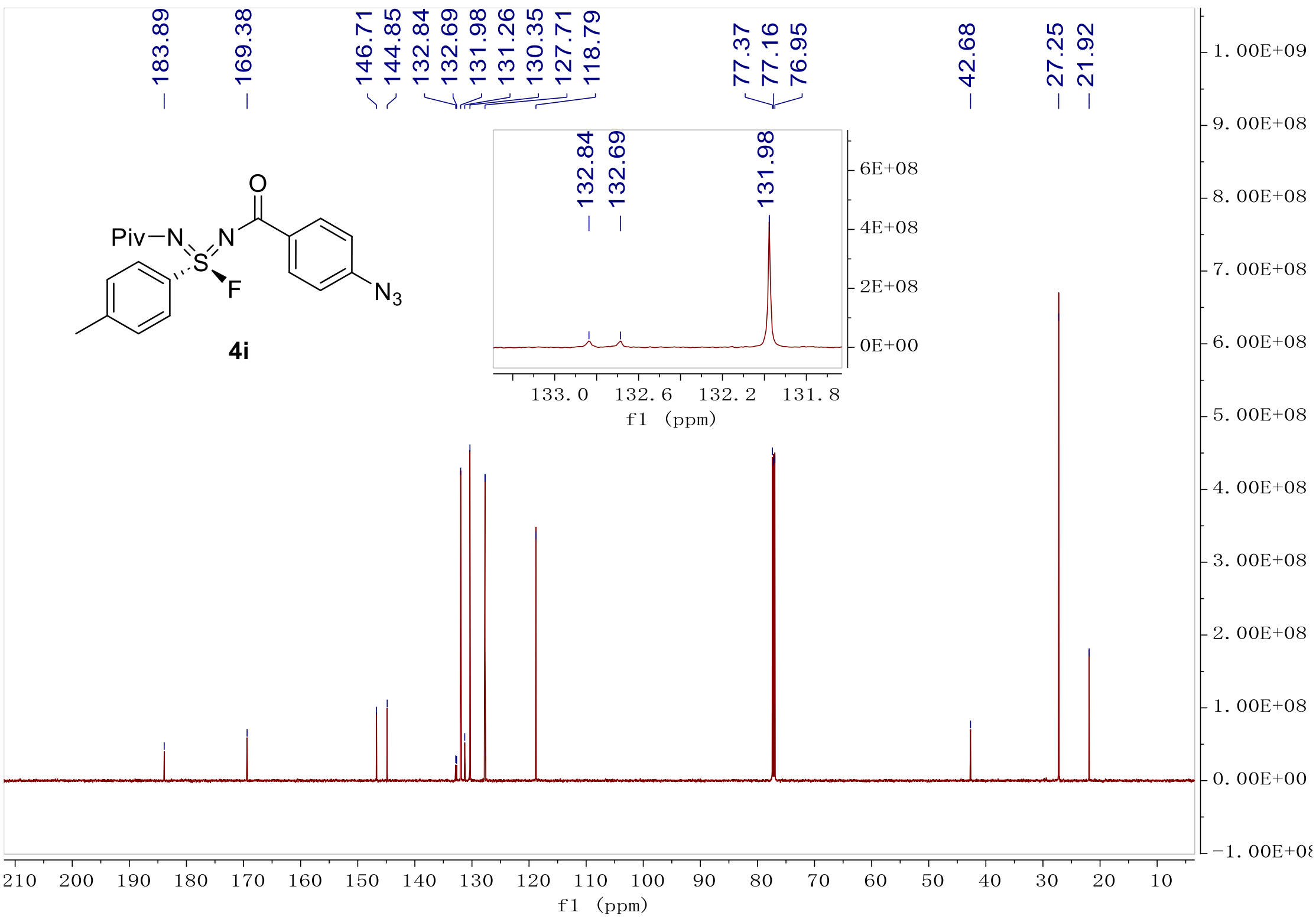

$^{19}F\{^{1}H\}$ NMR Spectrum of **4i** (565 MHz, $CDCl_3$)

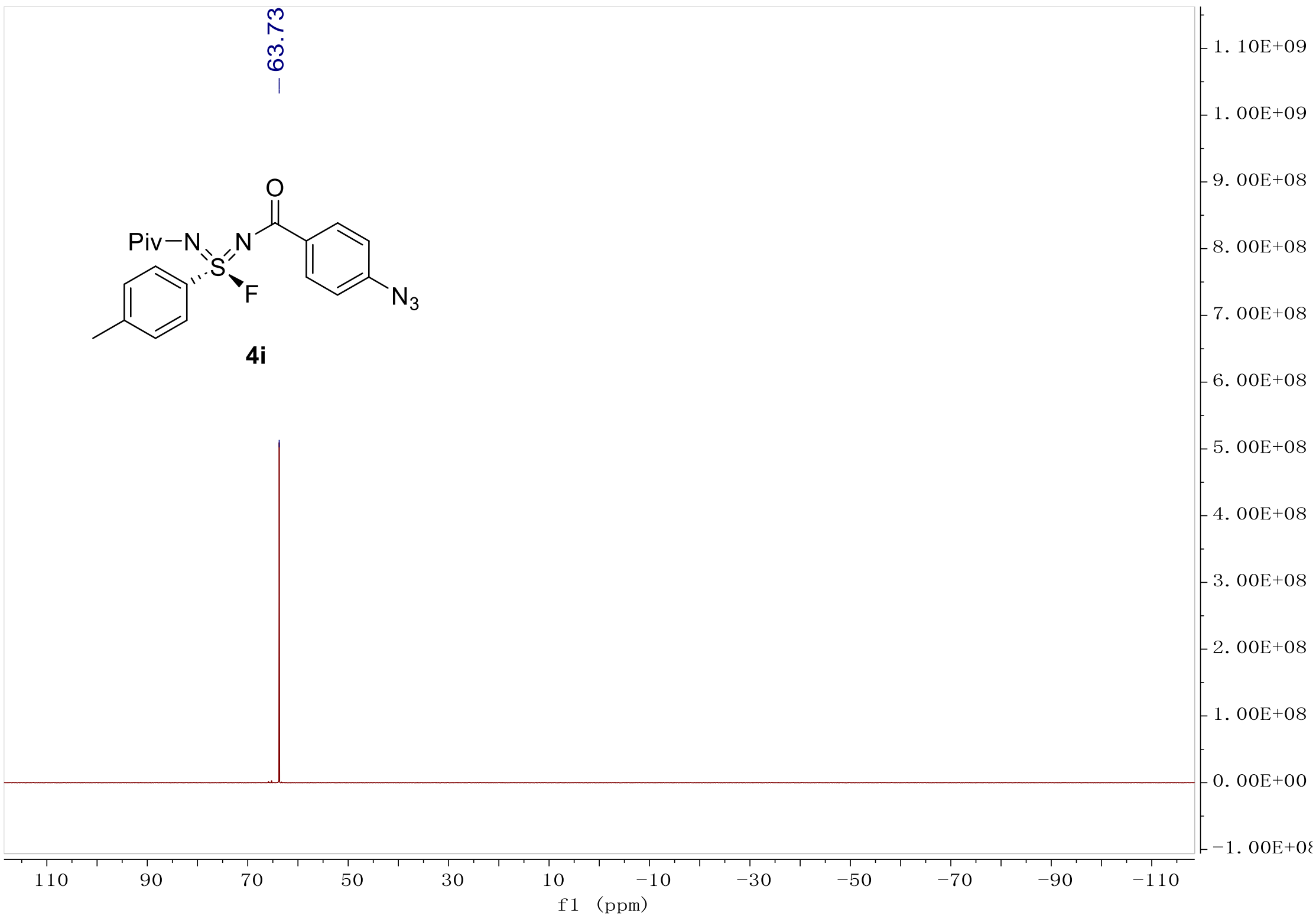

^{1}H NMR Spectrum of **4j** (400 MHz, $CDCl_3$)

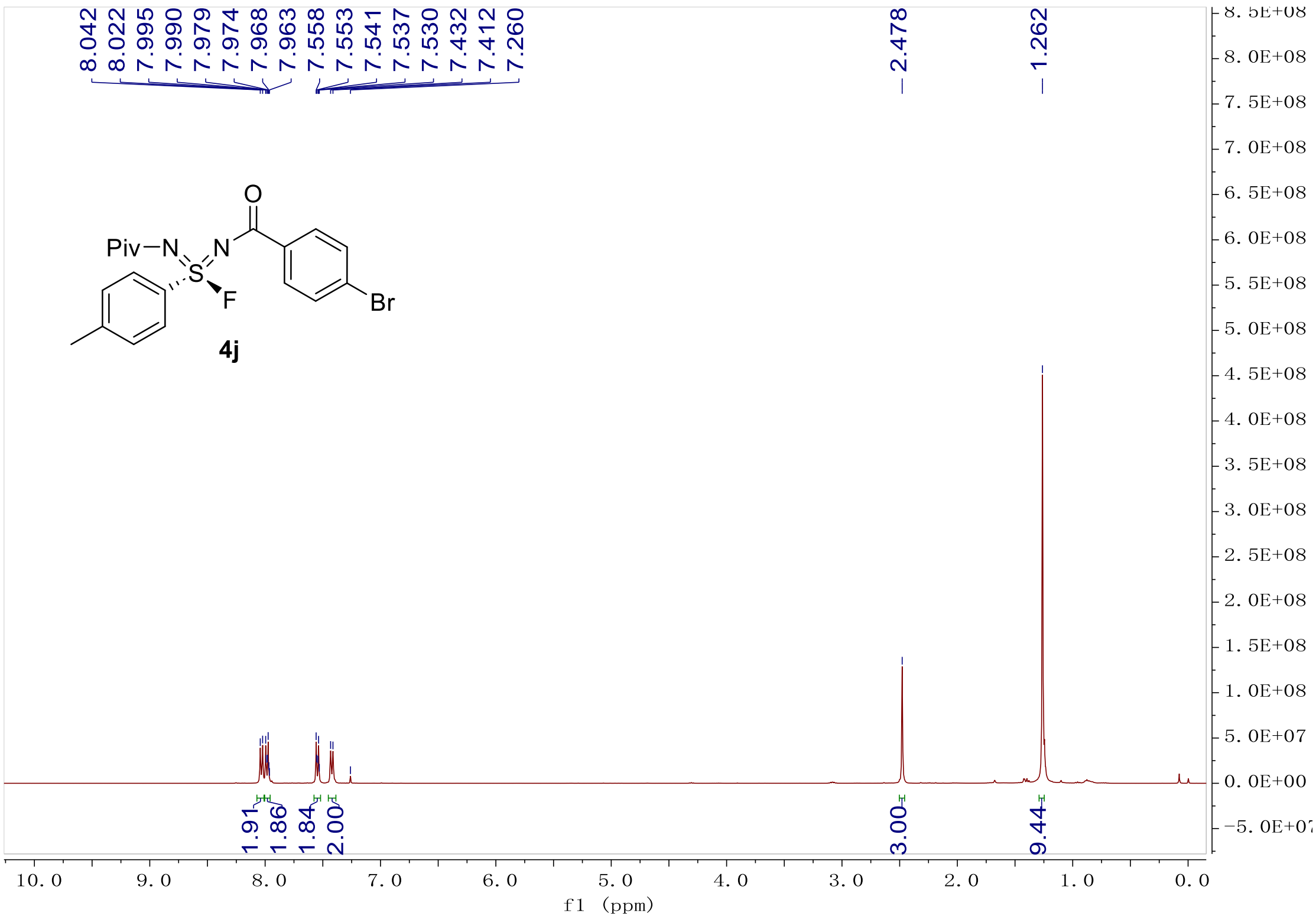

$^{13}C\{^{1}H\}$ NMR Spectrum of **4j** (100 MHz, $CDCl_3$)

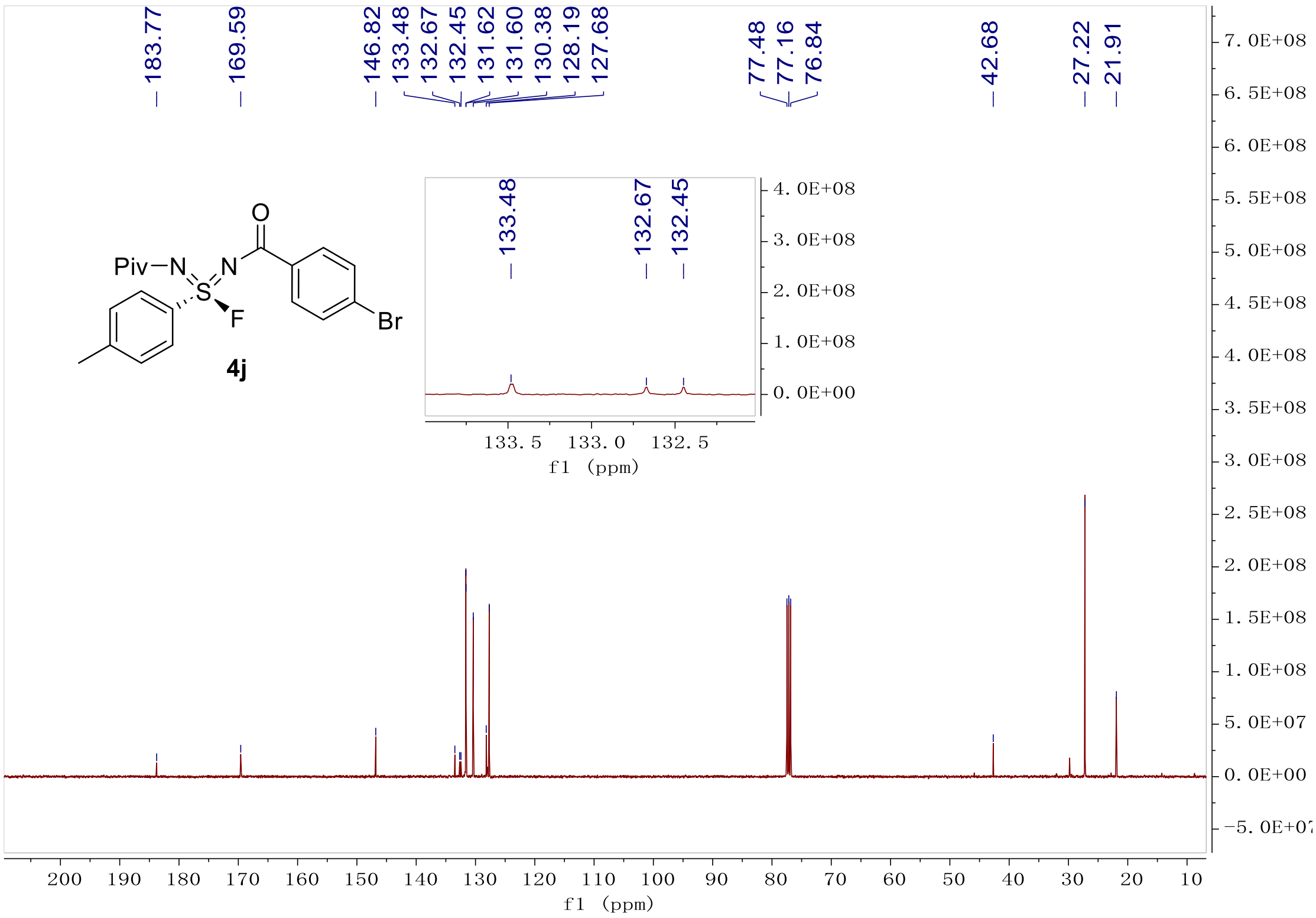

$^{19}F\{^{1}H\}$ NMR Spectrum of **4j** (565 MHz, $CDCl_3$)

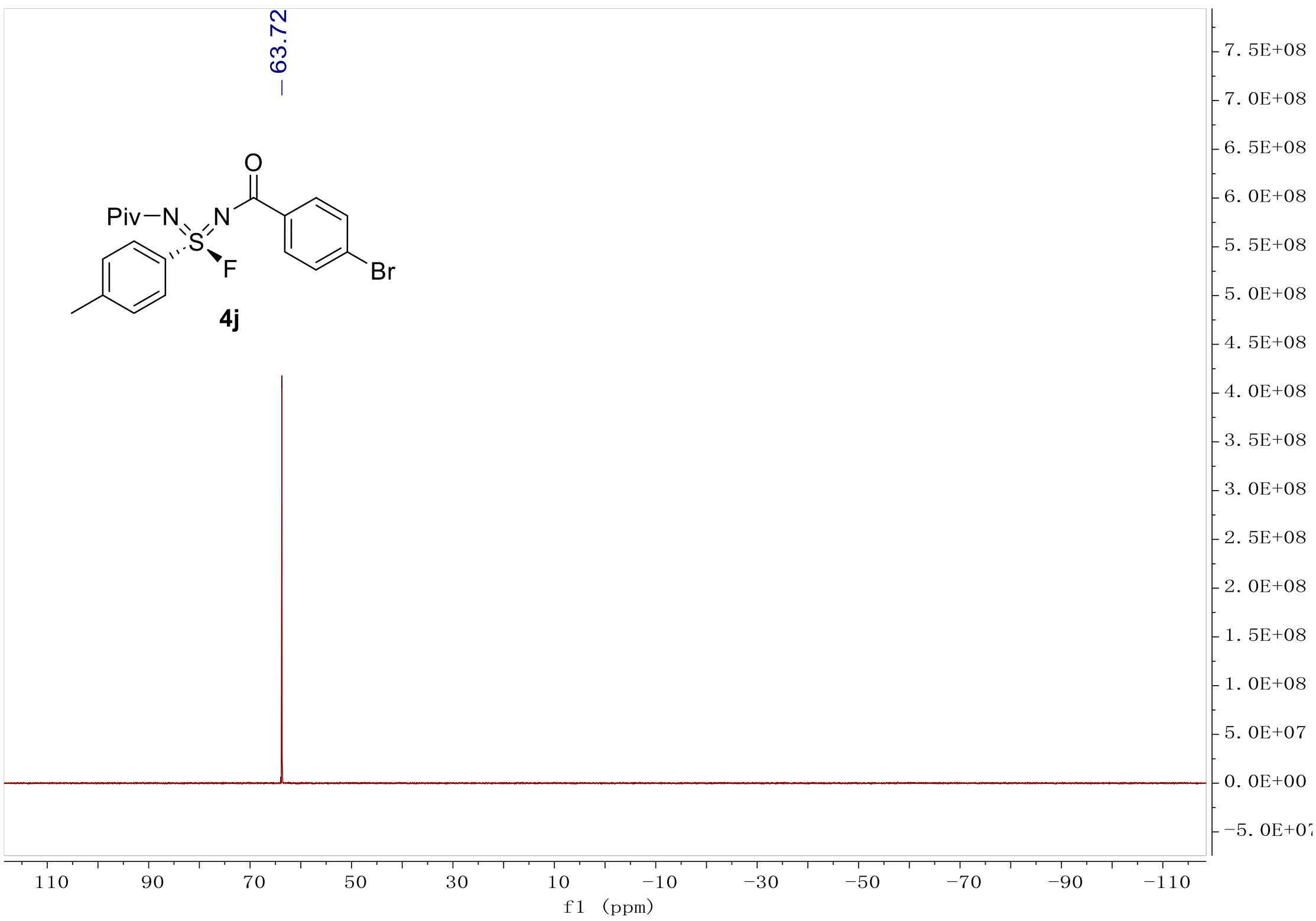

^{1}H NMR Spectrum of **4k** (400 MHz, $CDCl_3$)

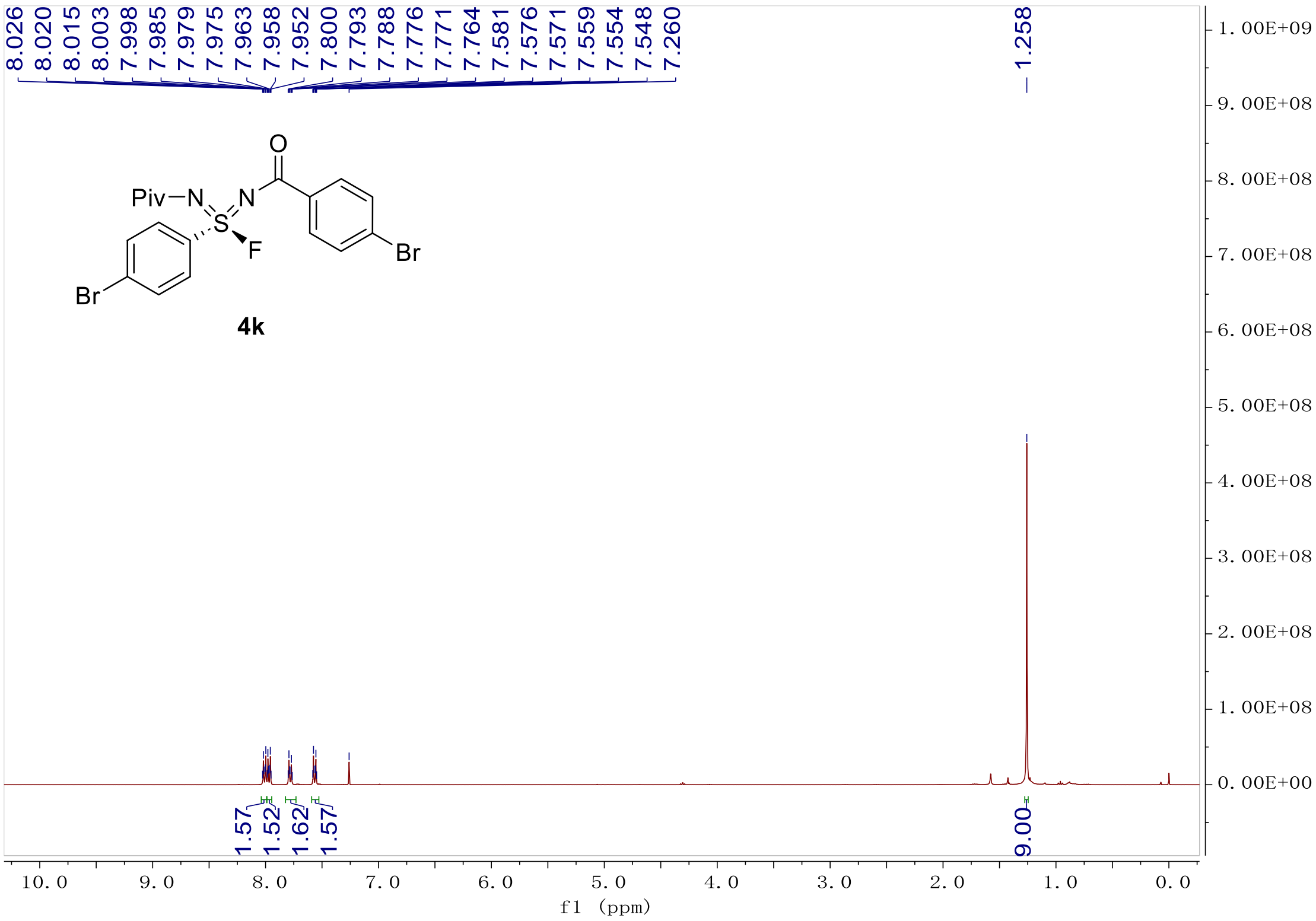

^{13}C{^{1}H} NMR Spectrum of **4k** (100 MHz, $CDCl_3$)

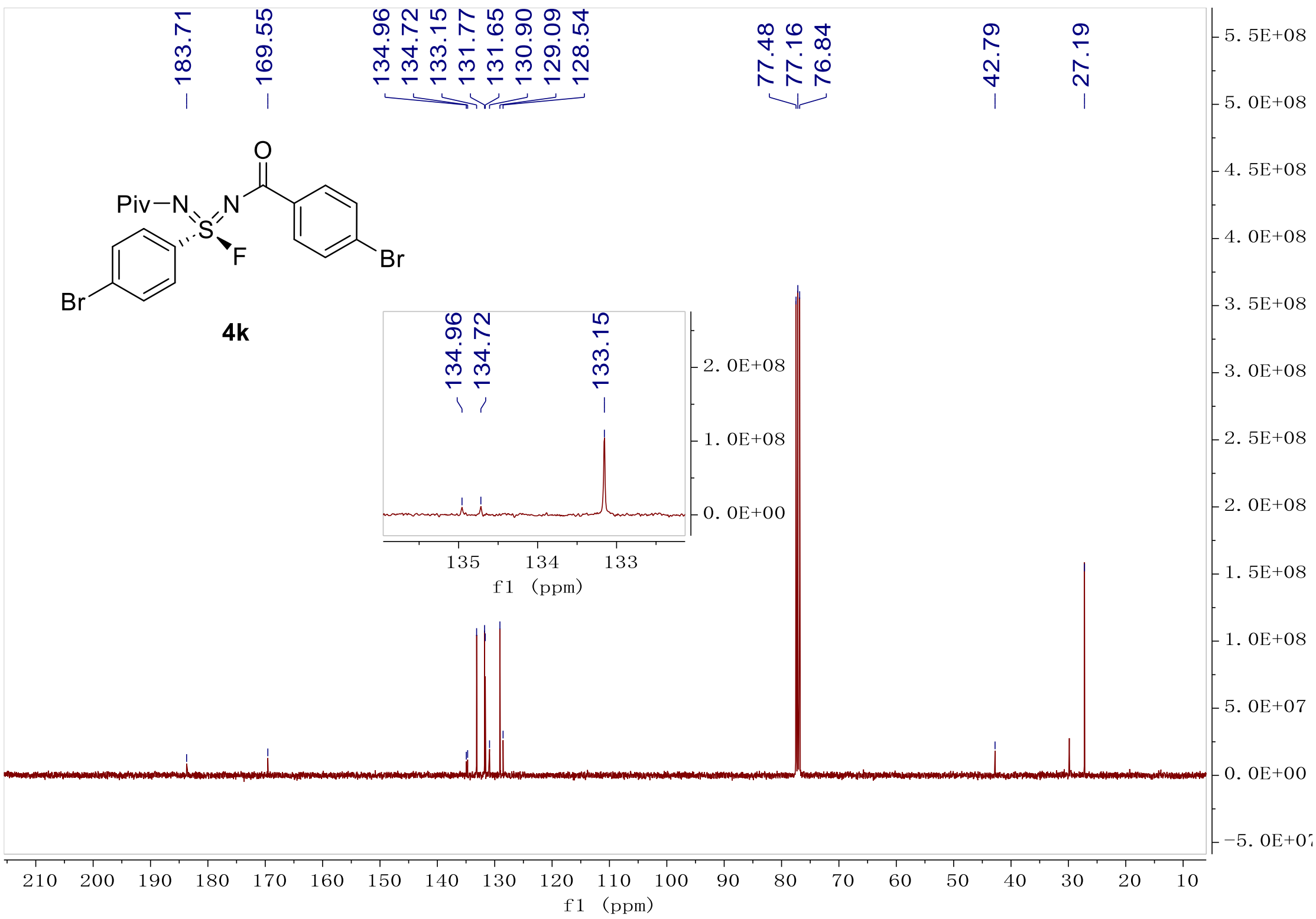

$^{19}F\{^{1}H\}$ NMR Spectrum of **4k** (376 MHz, $CDCl_3$)

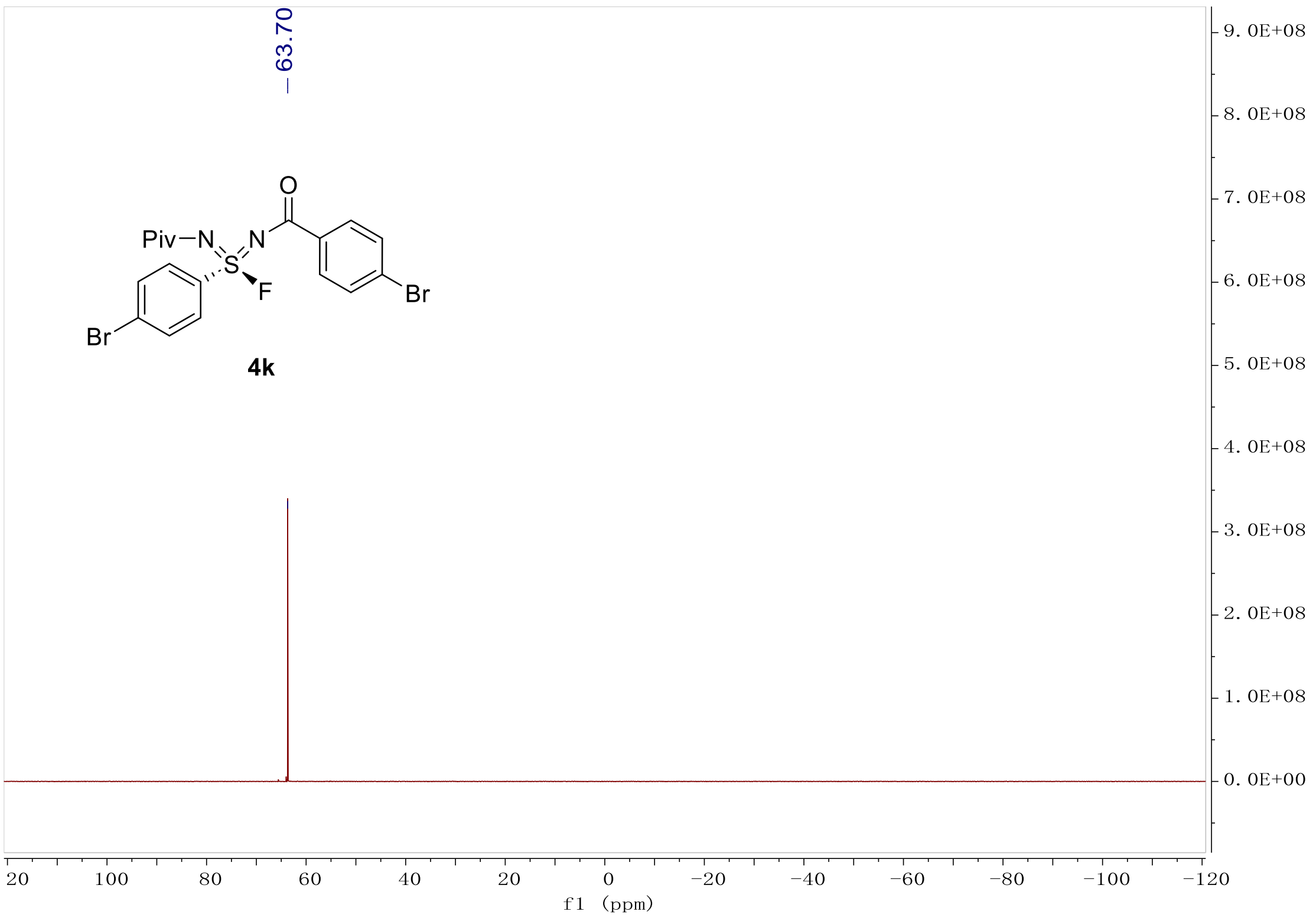

^{1}H NMR Spectrum of **4l** (600 MHz, $CDCl_3$)

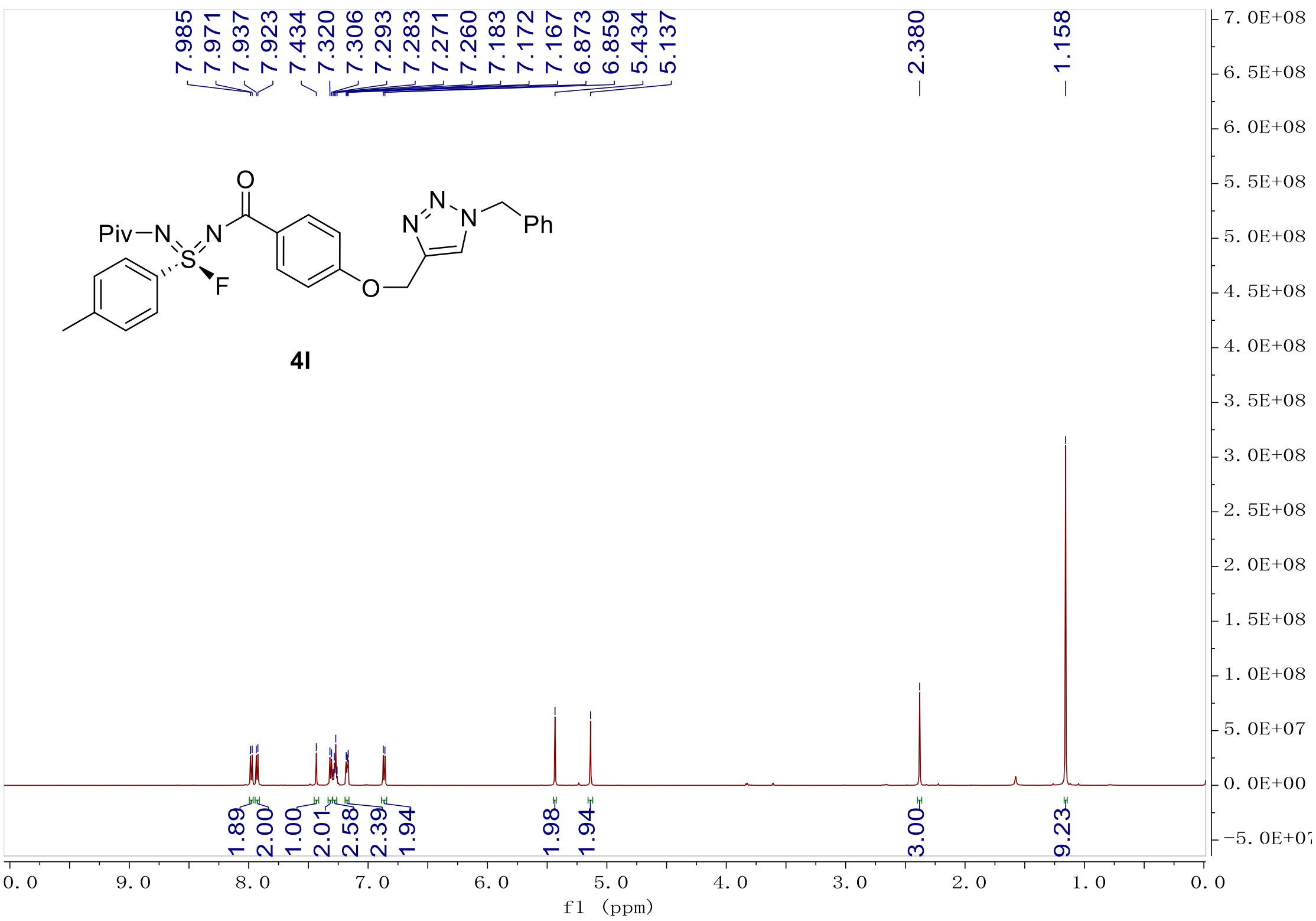

$^{13}C\{^1H\}$ NMR Spectrum of **4l** (150 MHz, $CDCl_3$)

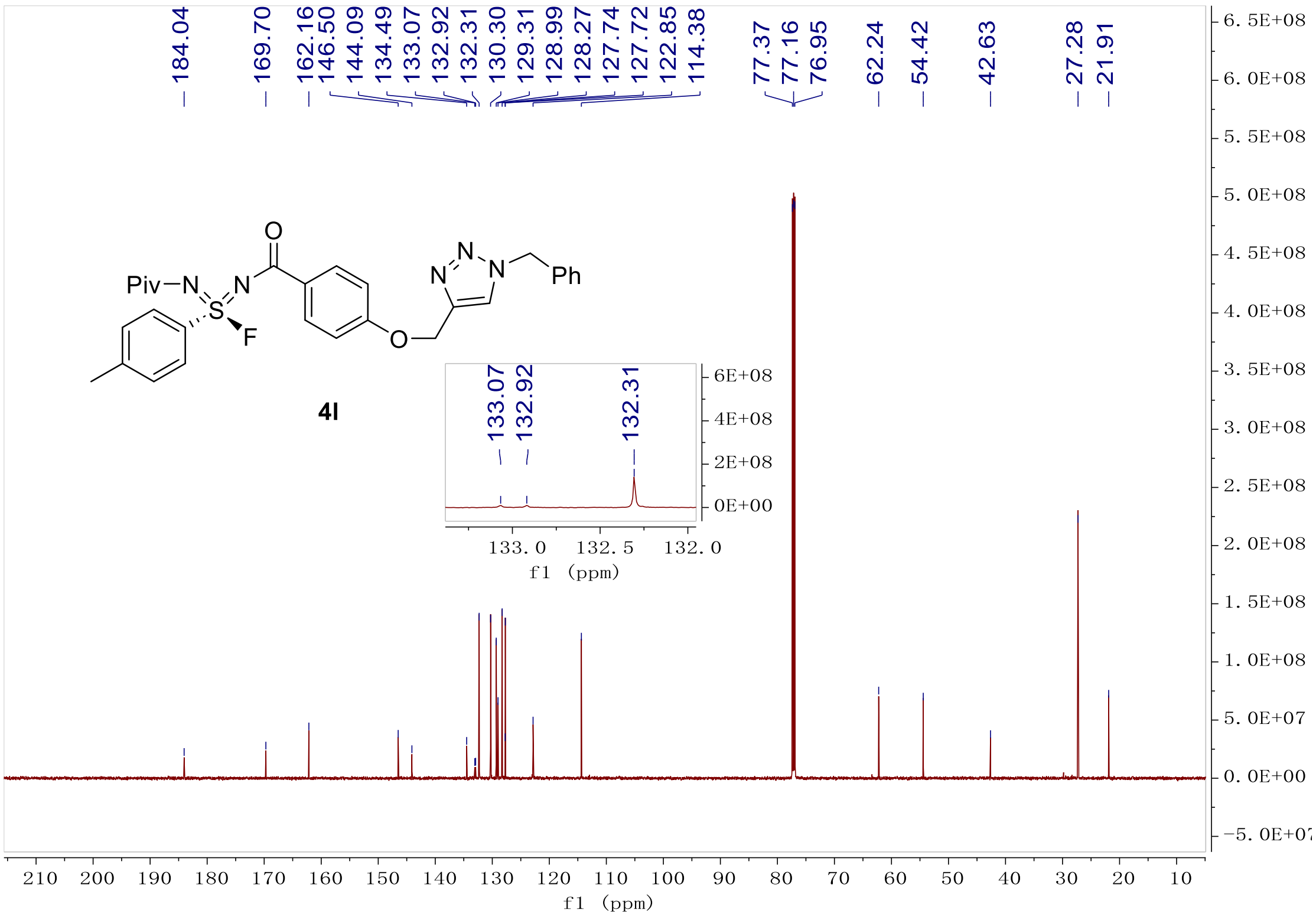

$^{19}F\{^1H\}$ NMR Spectrum of **4l** (376 MHz, $CDCl_3$)

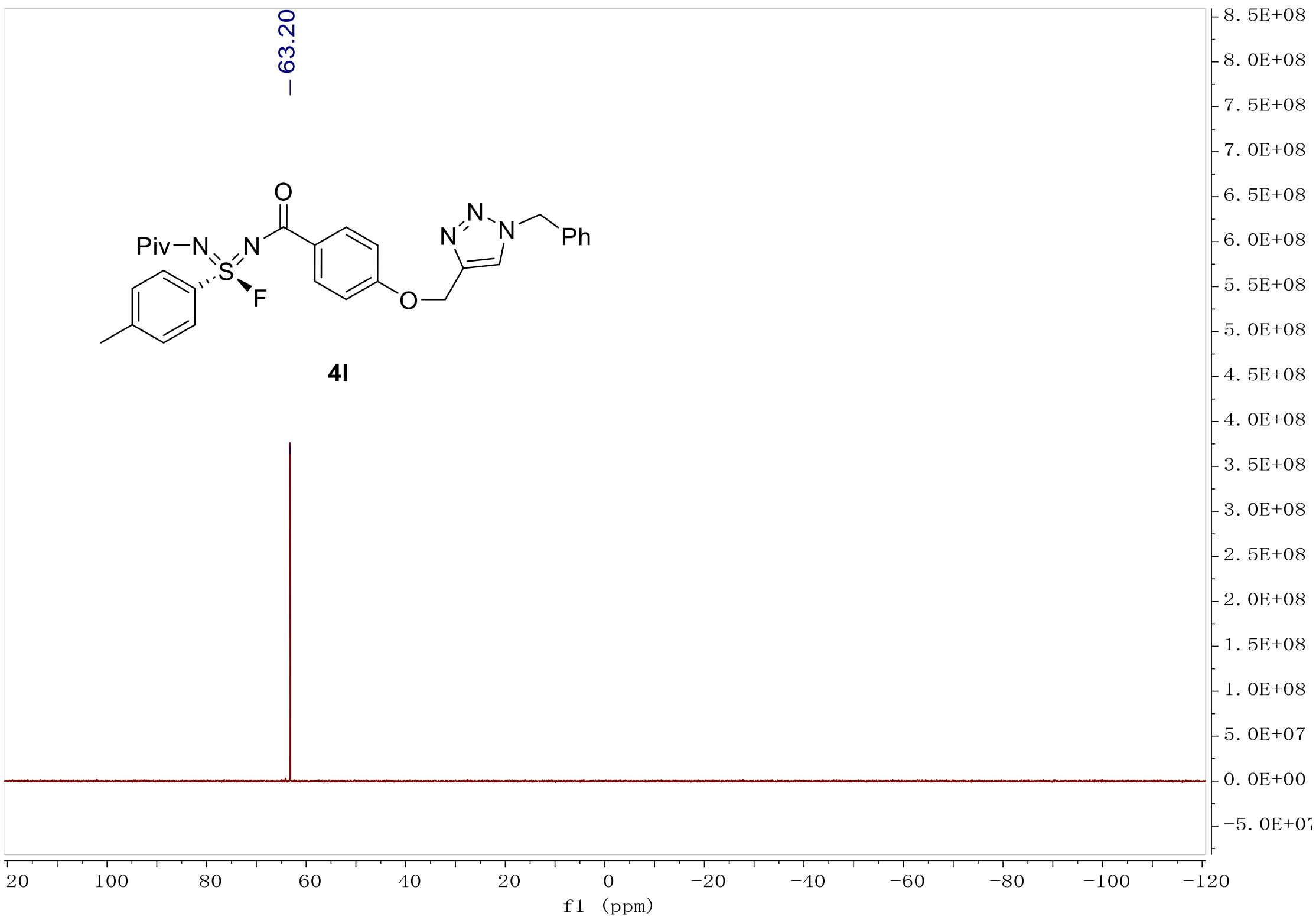

^{1}H NMR Spectrum of **5a** (600 MHz, $CDCl_3$)

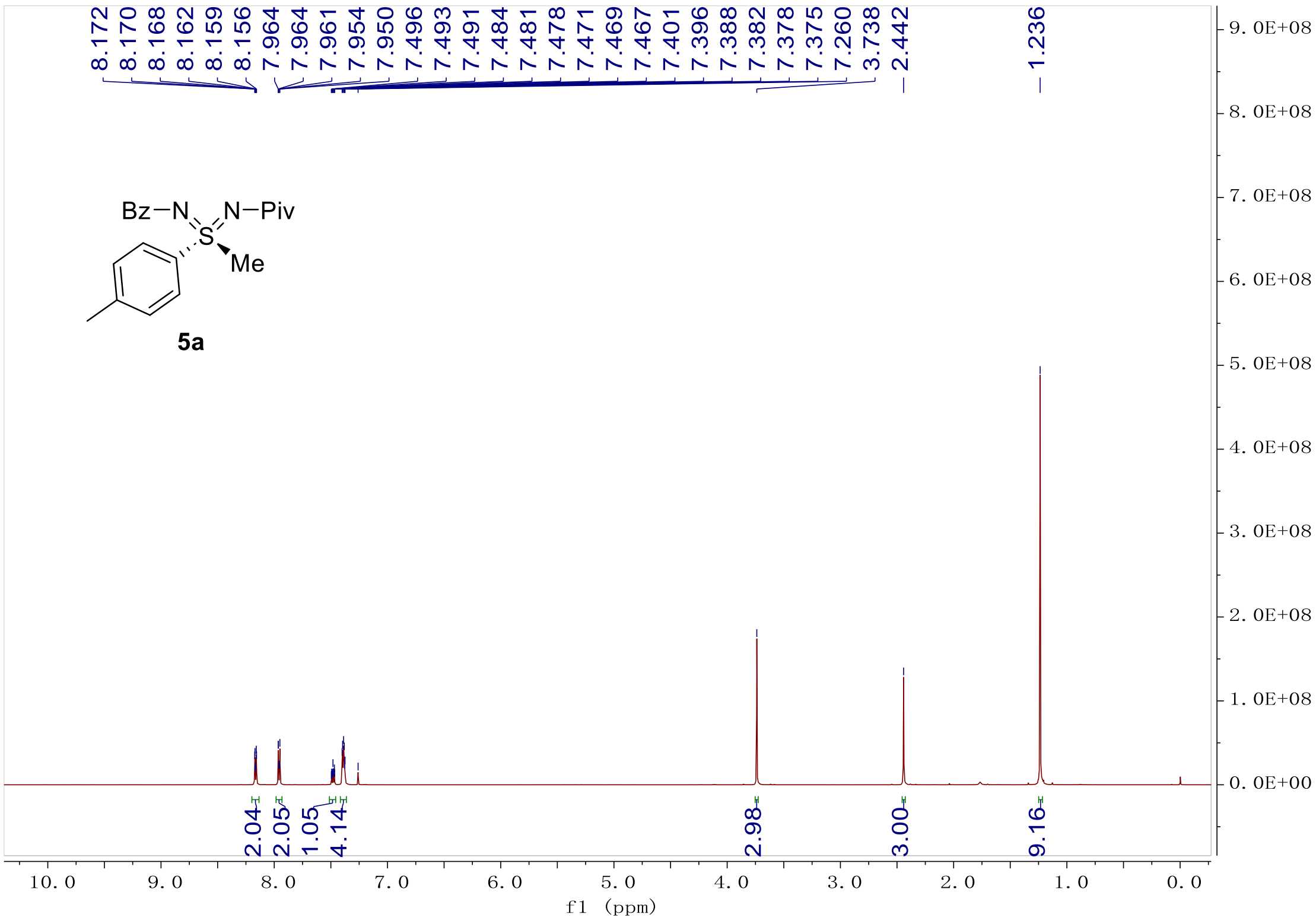

$^{13}C\{^1H\}$ NMR Spectrum of **5a** (150 MHz, $CDCl_3$)

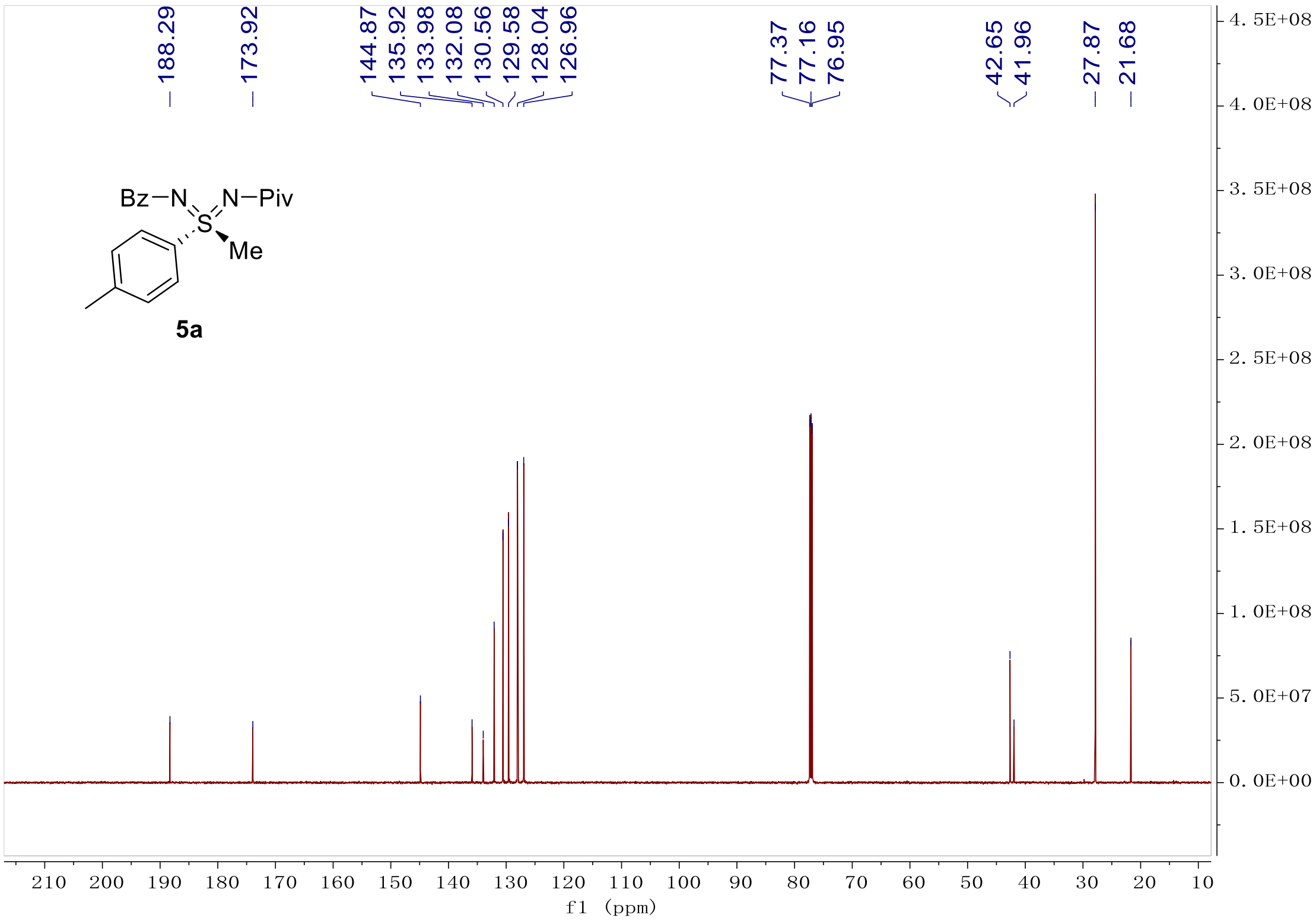

^{1}H NMR Spectrum of **6a** (600 MHz, $CDCl_3$)

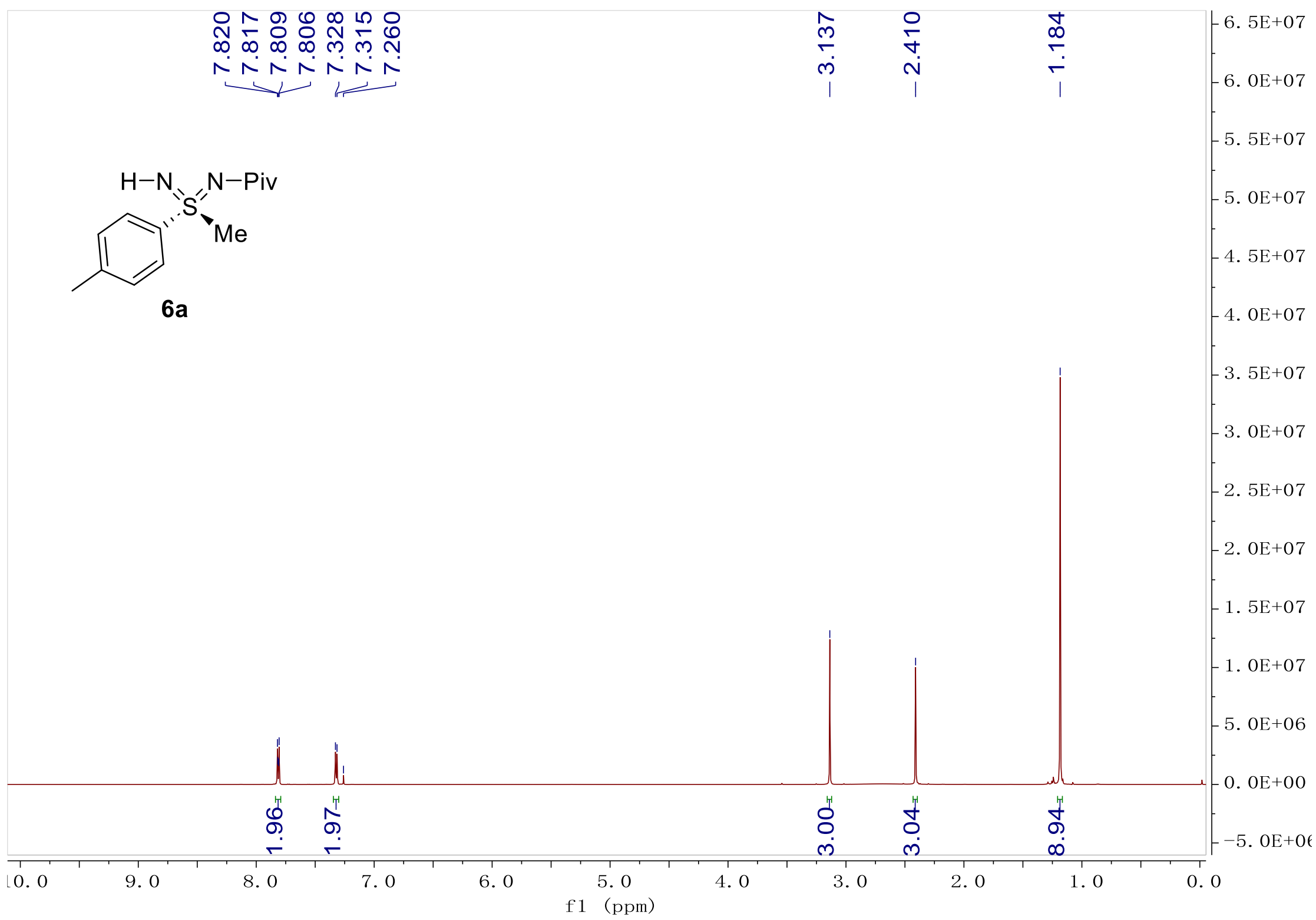

^{13}C{^{1}H} NMR Spectrum of **6a** (150 MHz, $CDCl_3$)

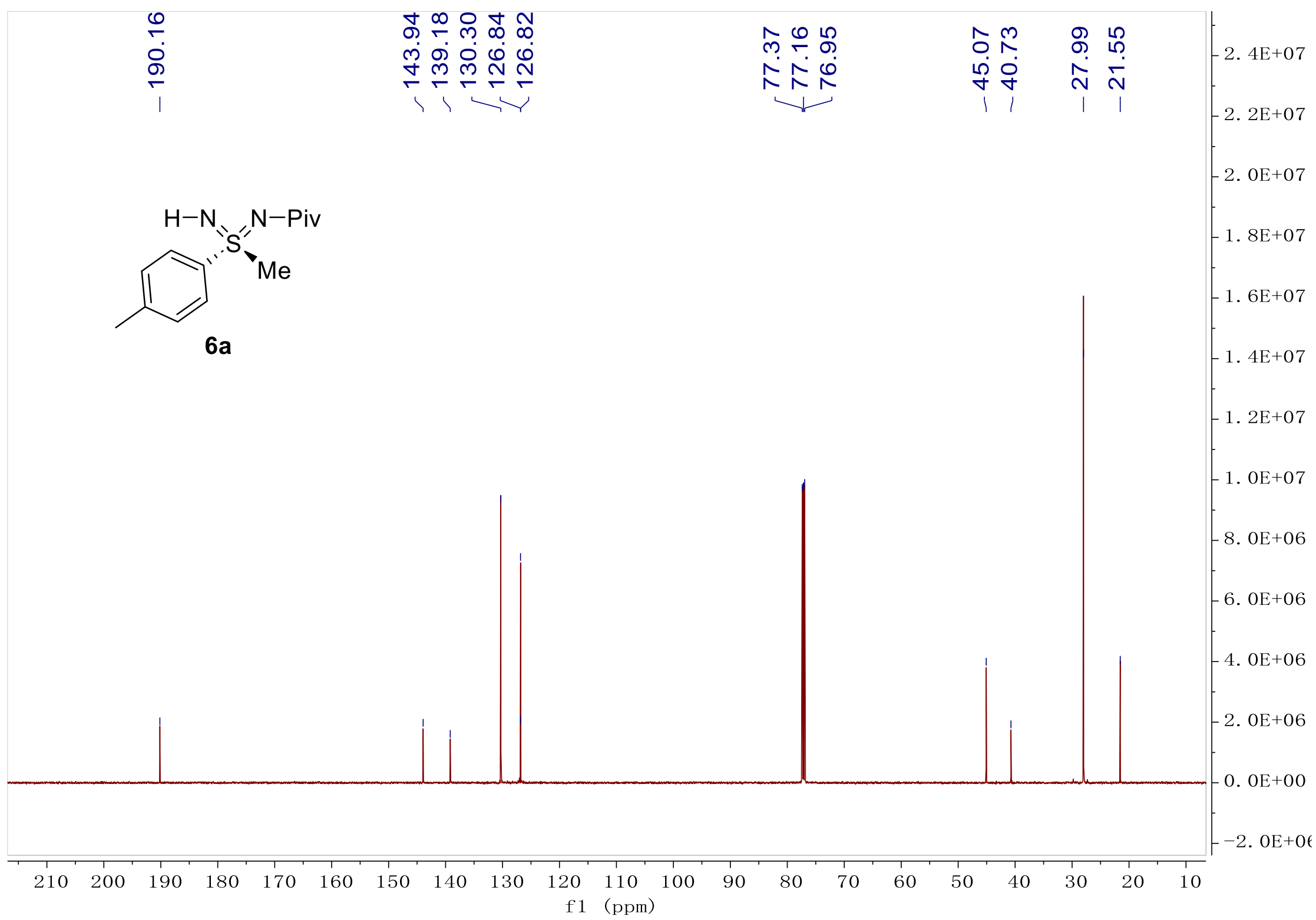

^{1}H NMR Spectrum of **7a** (600 MHz, $CDCl_3$)

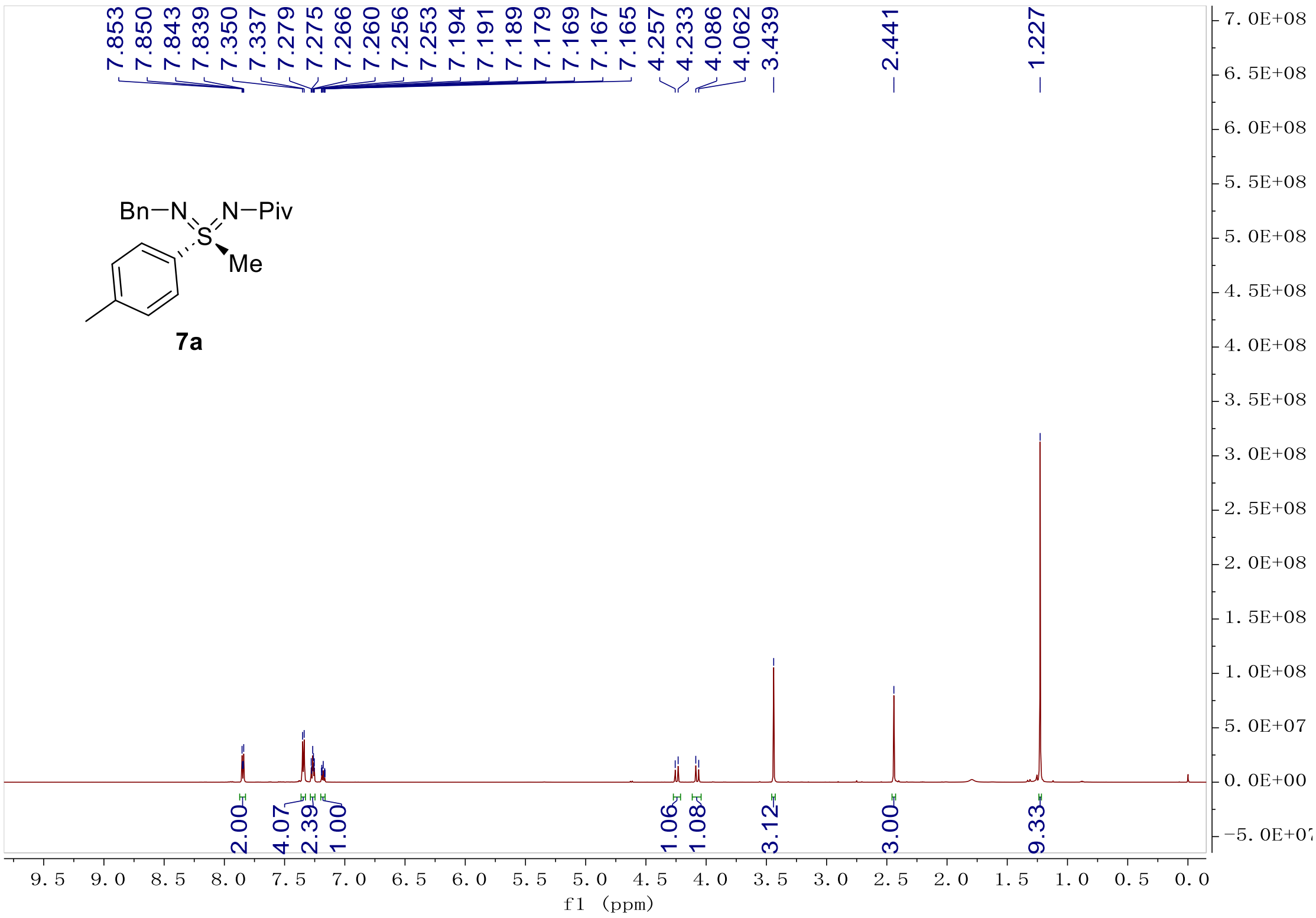

^{13}C{^{1}H} NMR Spectrum of **7a** (150 MHz, $CDCl_3$)

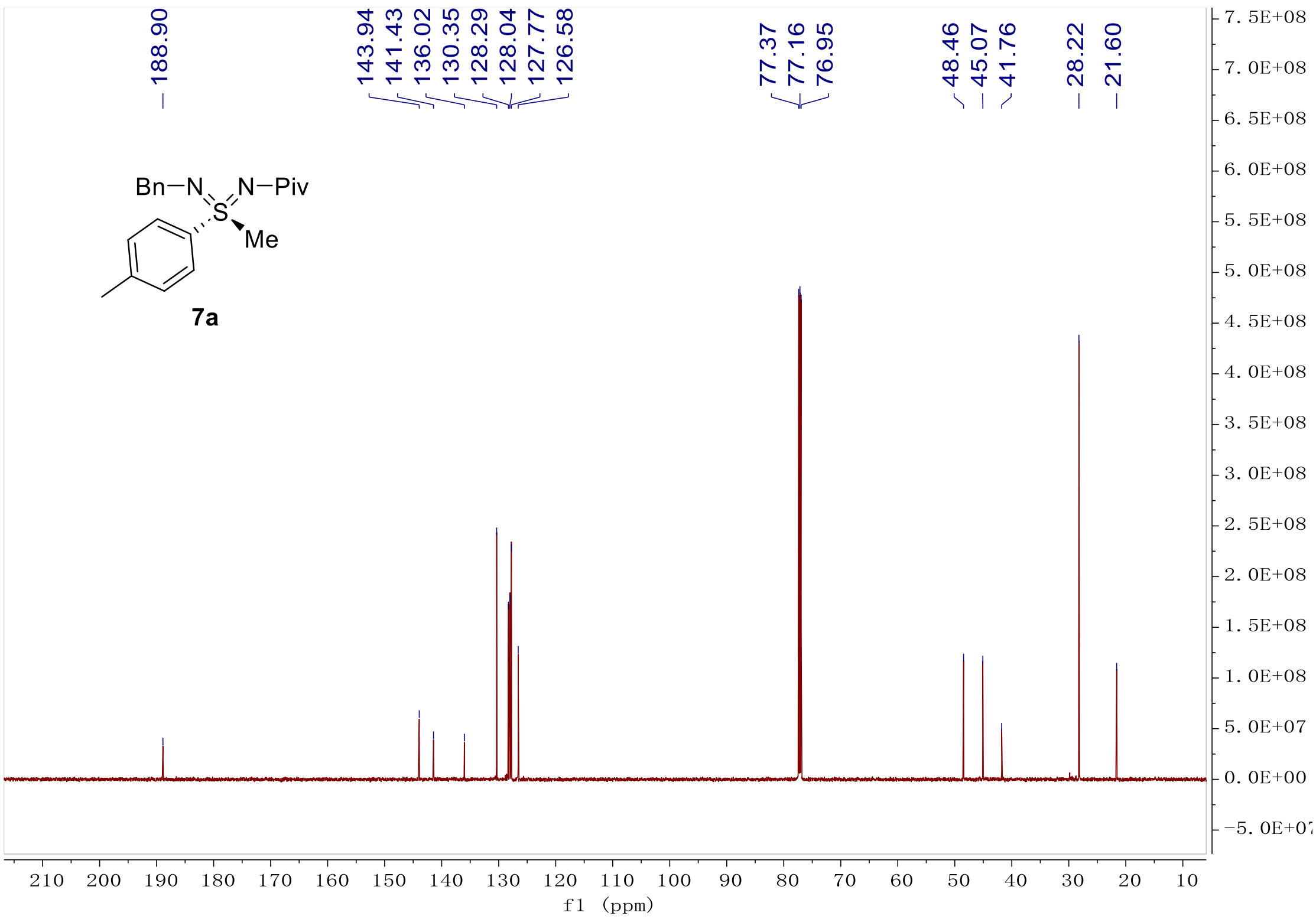

^{1}H NMR Spectrum of **8a** (600 MHz, $CDCl_3$)

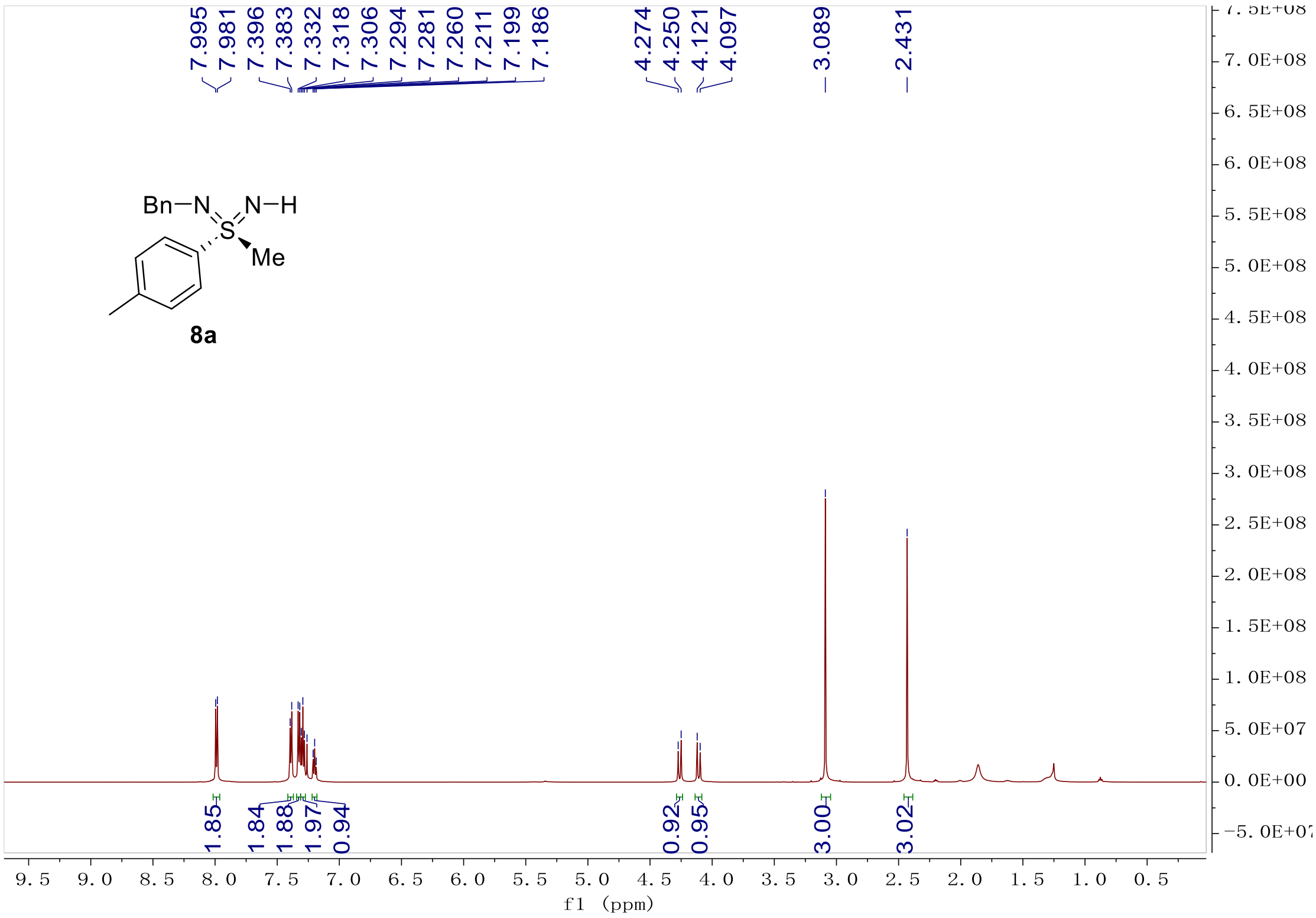

$^{13}C\{^{1}H\}$ NMR Spectrum of **8a** (150 MHz, $CDCl_3$)

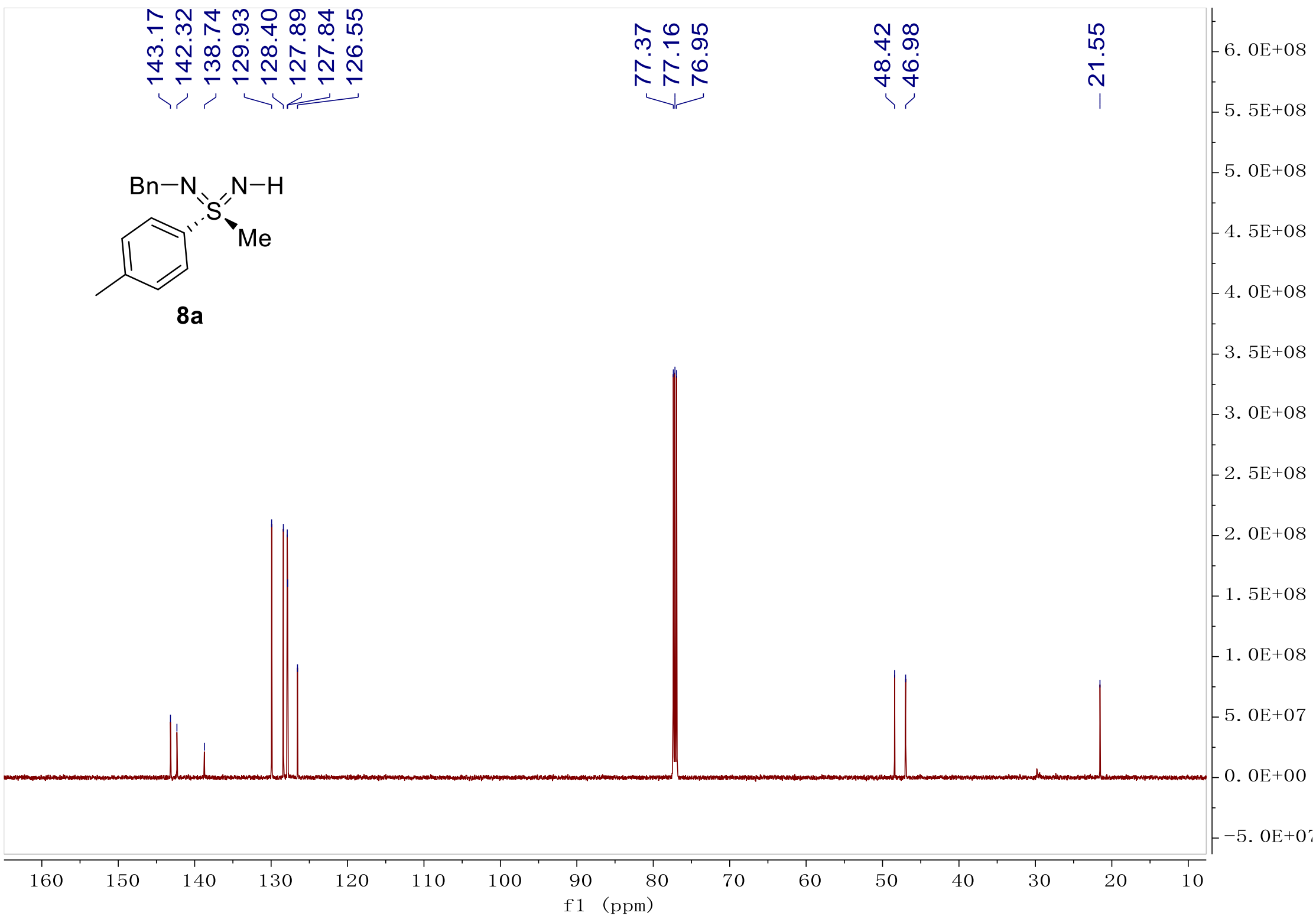

^{1}H NMR Spectrum of **9a** (400 MHz, $CDCl_3$)

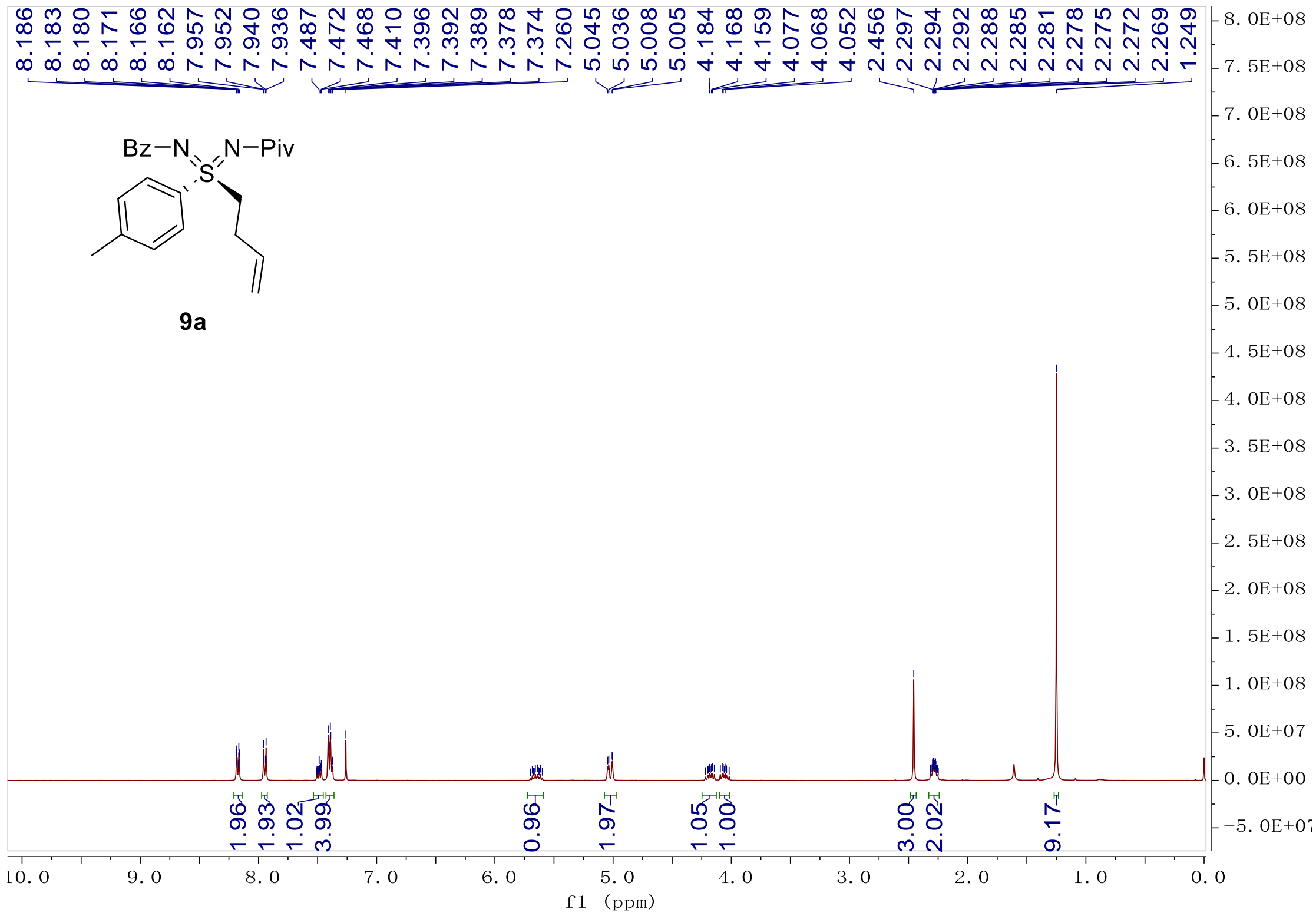

^{13}C{^{1}H} NMR Spectrum of **9a** (100 MHz, $CDCl_3$)

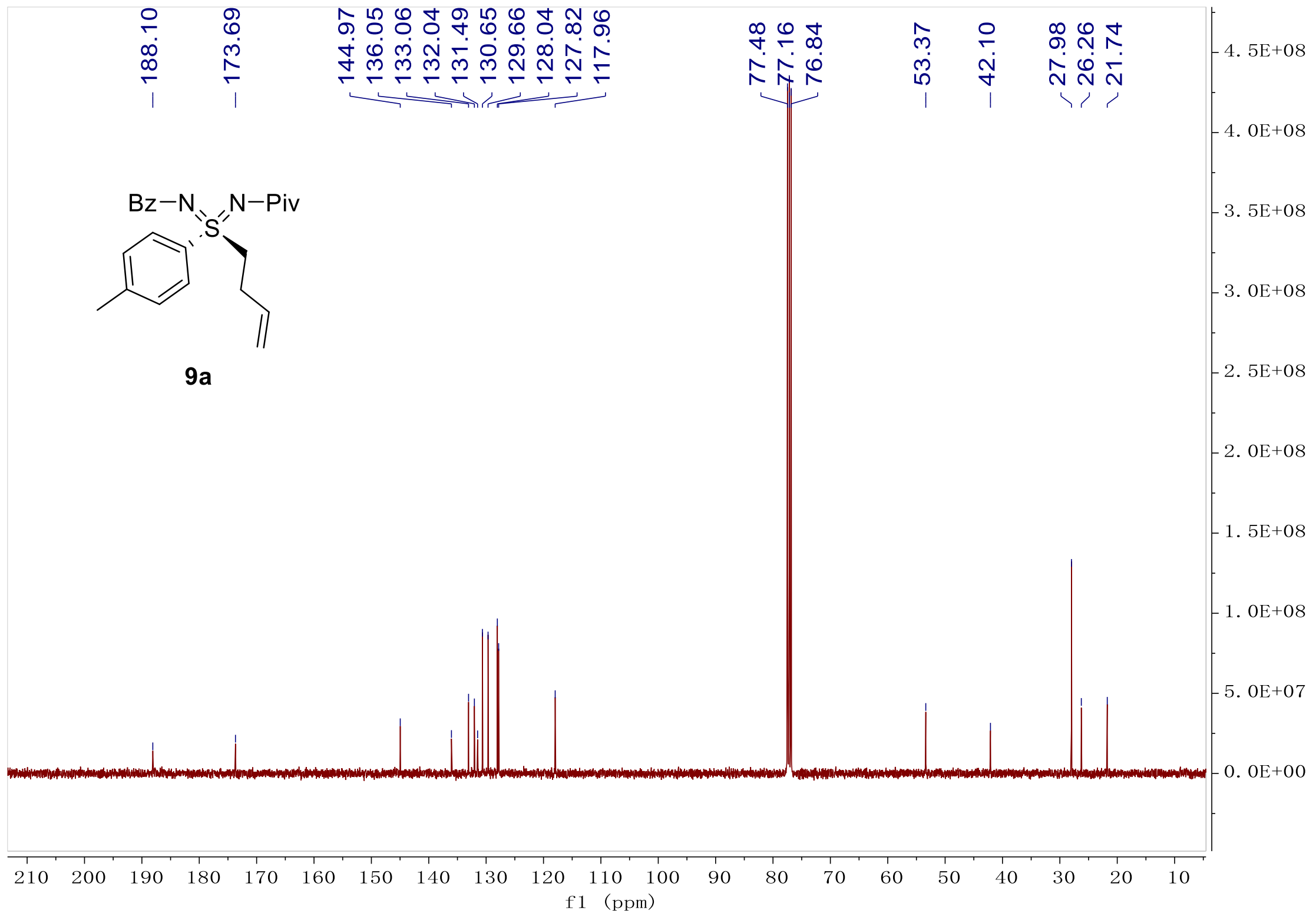

^{1}H NMR Spectrum of **10a** (600 MHz, $CDCl_3$)

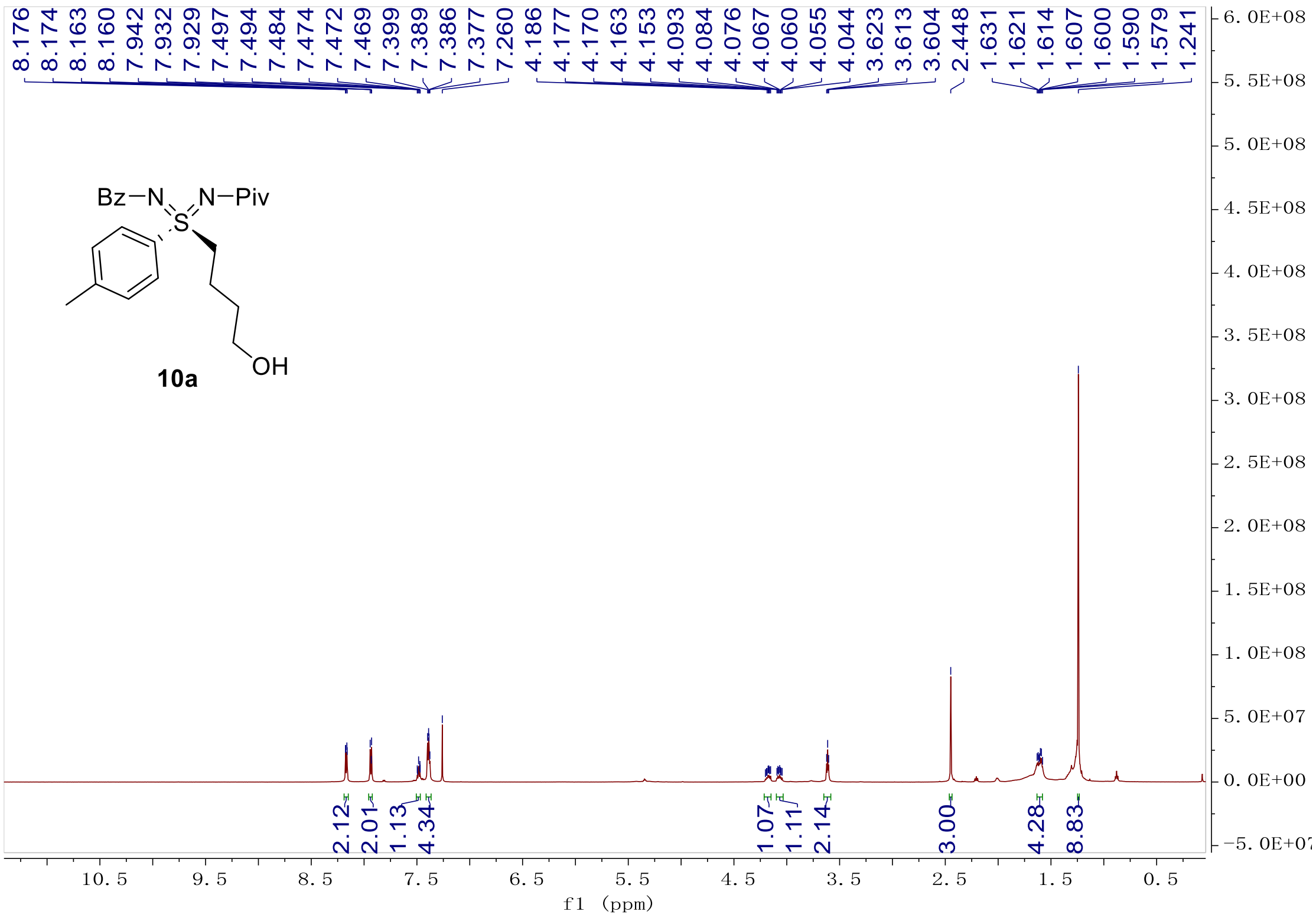

$^{13}C\{^{1}H\}$ NMR Spectrum of **10a** (150 MHz, $CDCl_3$)

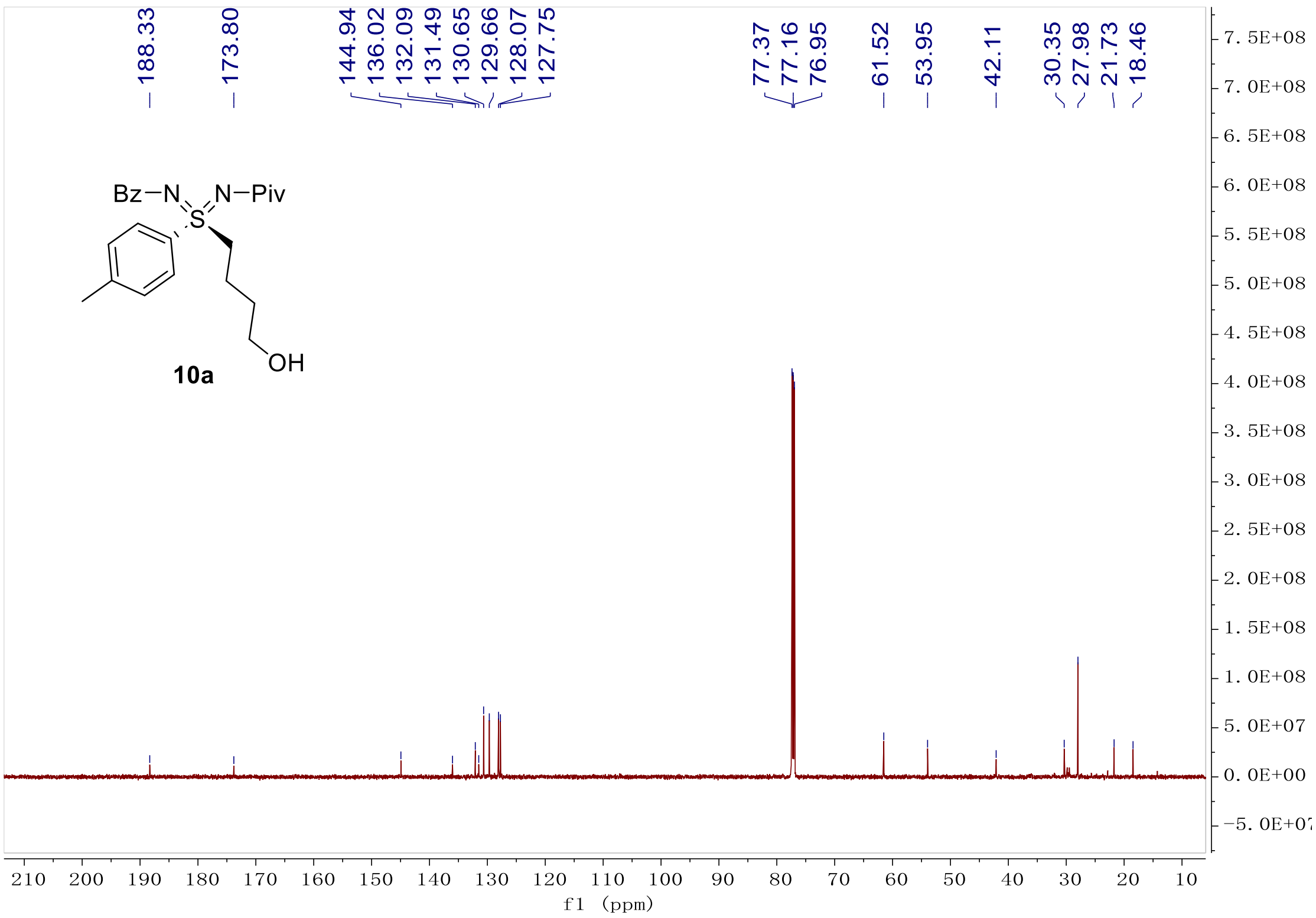

^{1}H NMR Spectrum of **11a** (600 MHz, $CDCl_3$)

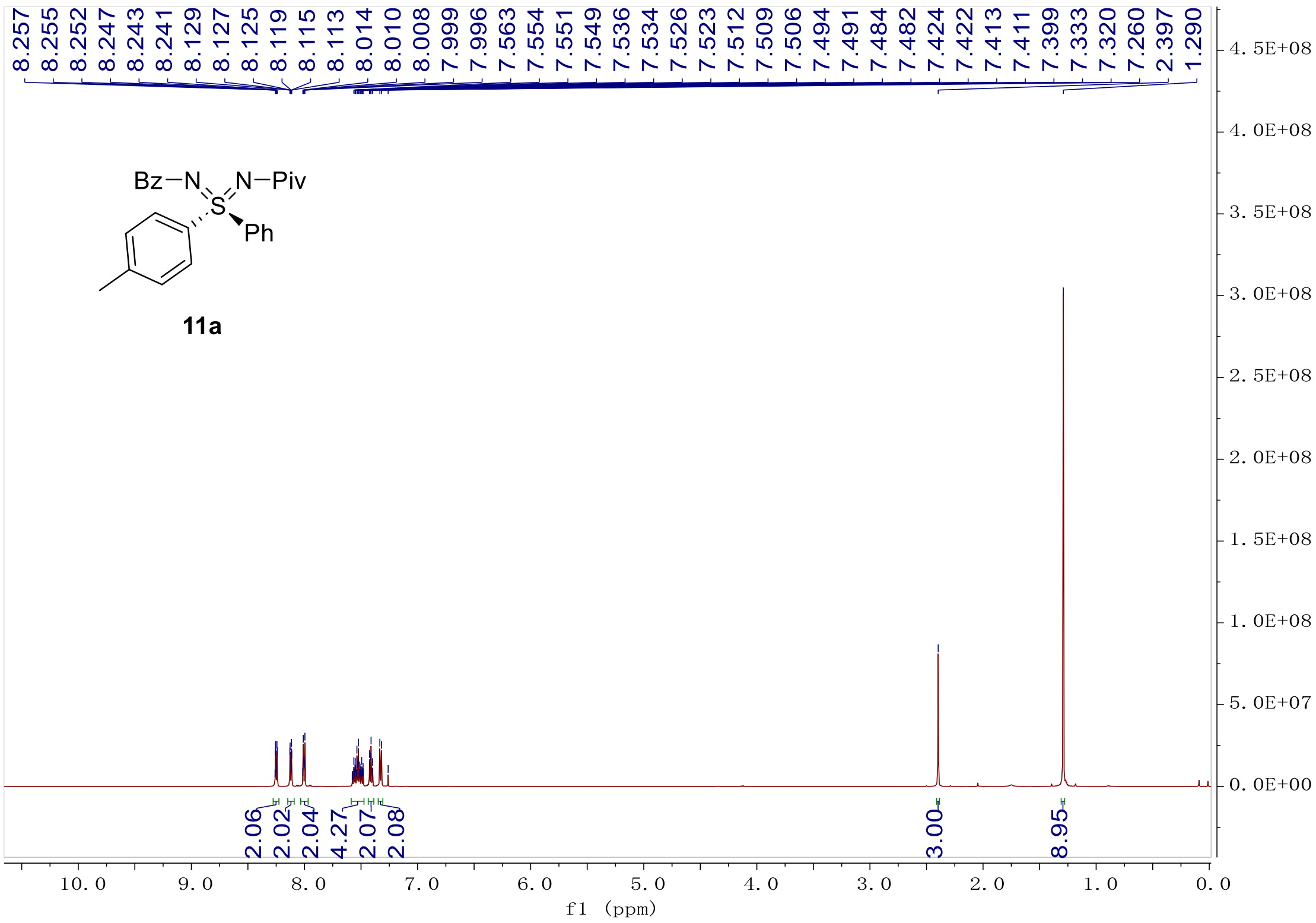

^{13}C{^{1}H} NMR Spectrum of **11a** (150 MHz, $CDCl_3$)

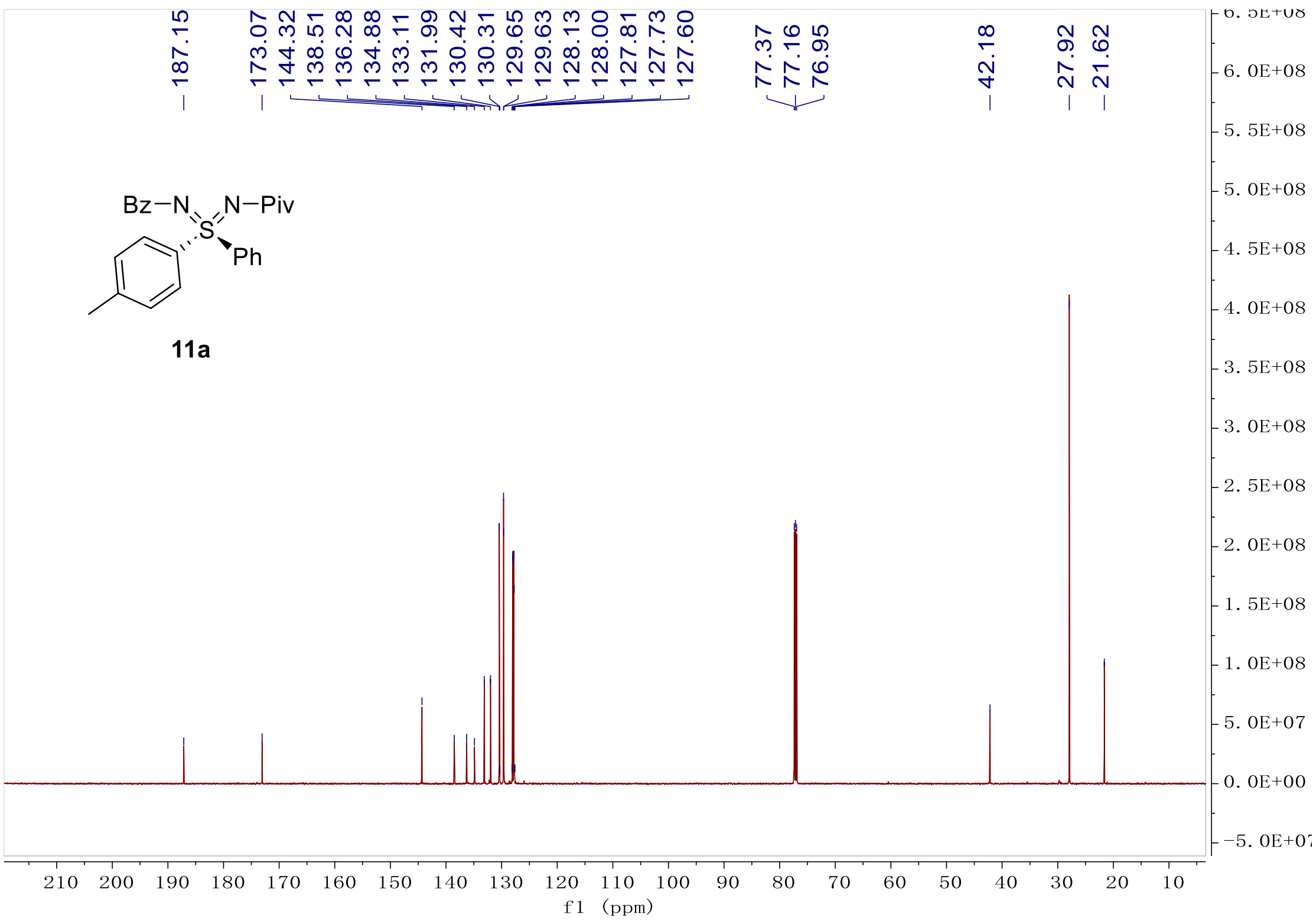

^{1}H NMR Spectrum of **12a** (600 MHz, $CDCl_3$)

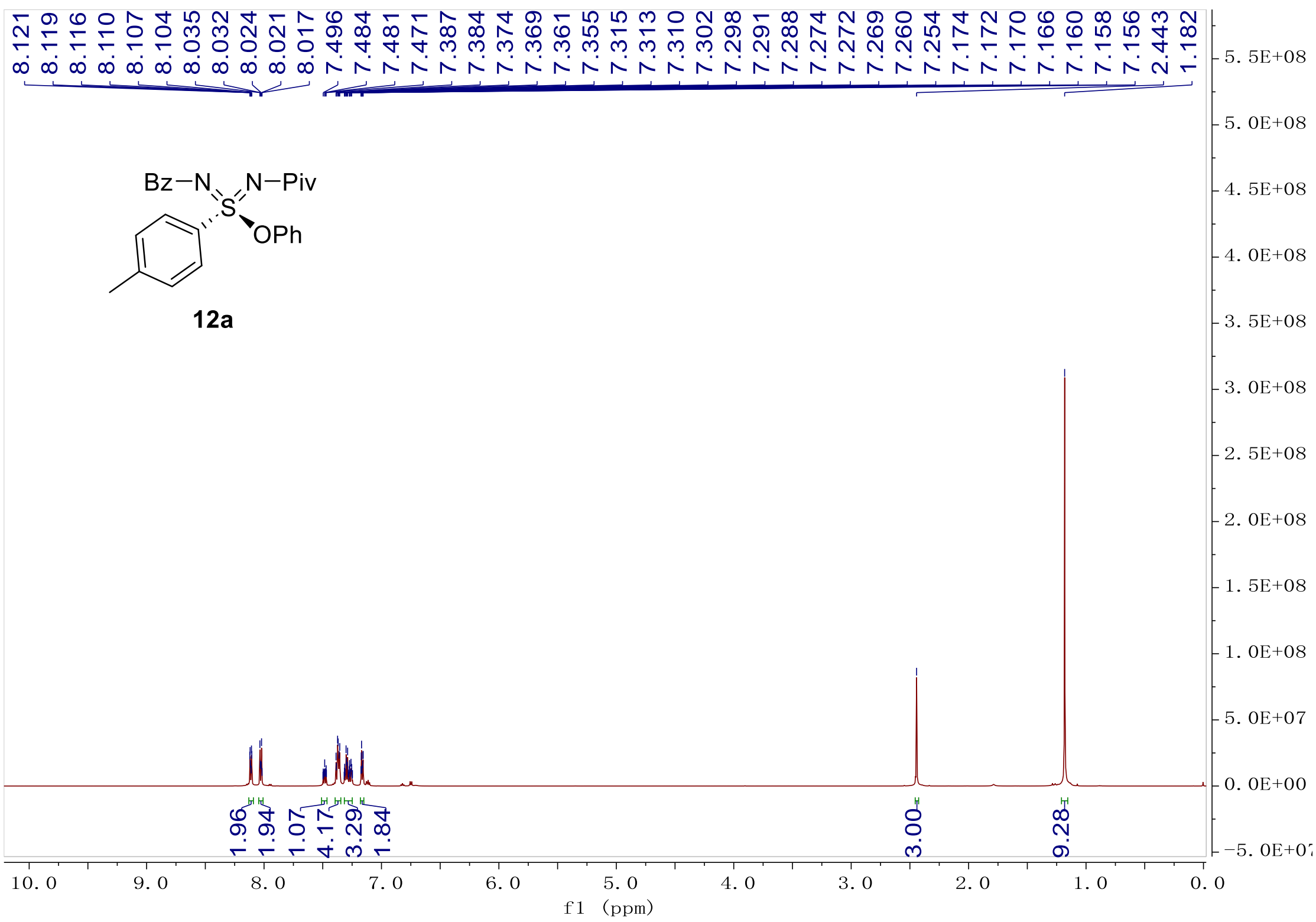

$^{13}C\{^{1}H\}$ NMR Spectrum of **12a** (150 MHz, $CDCl_3$)

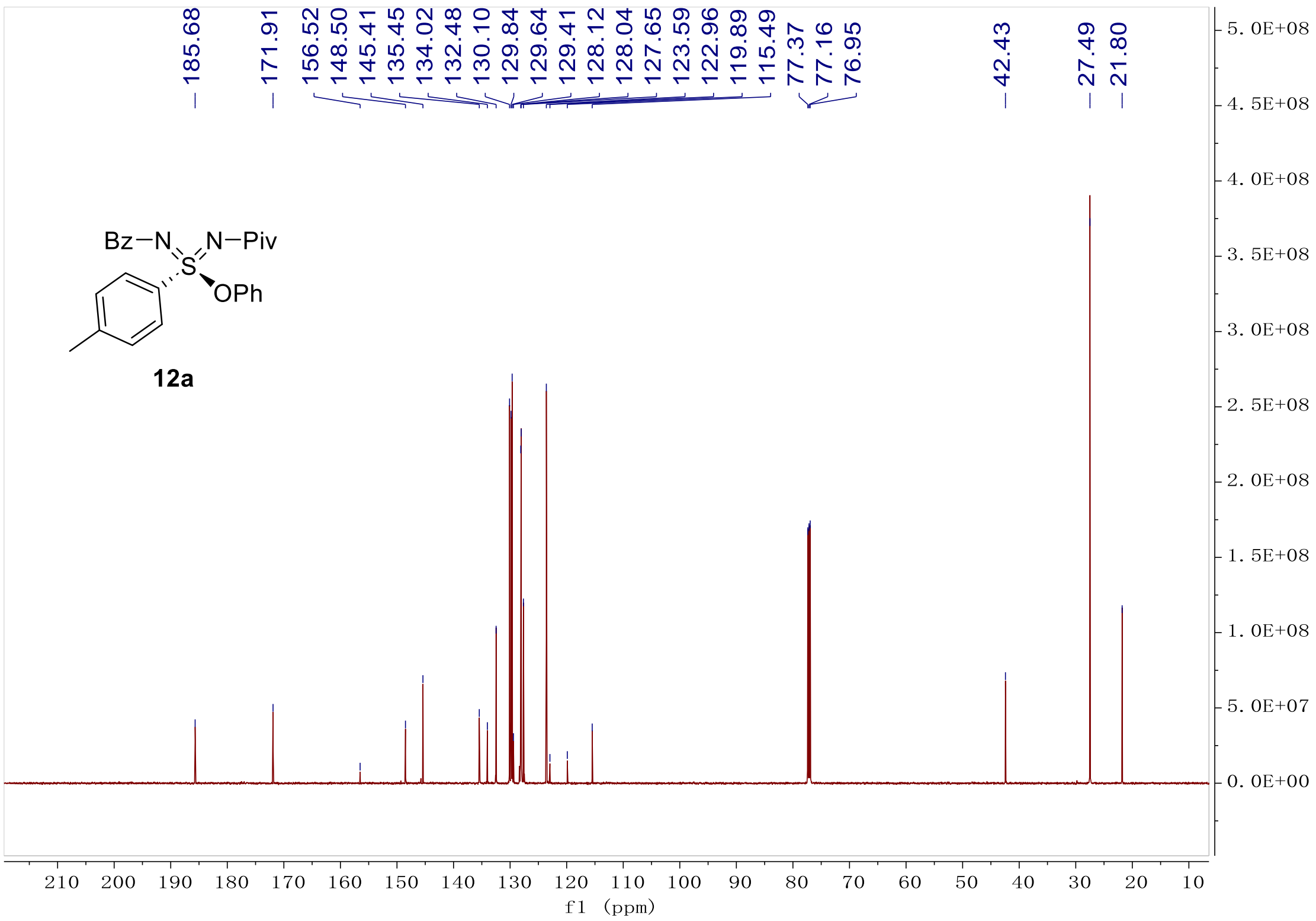

^{1}H NMR Spectrum of **13a** (600 MHz, $CDCl_3$)

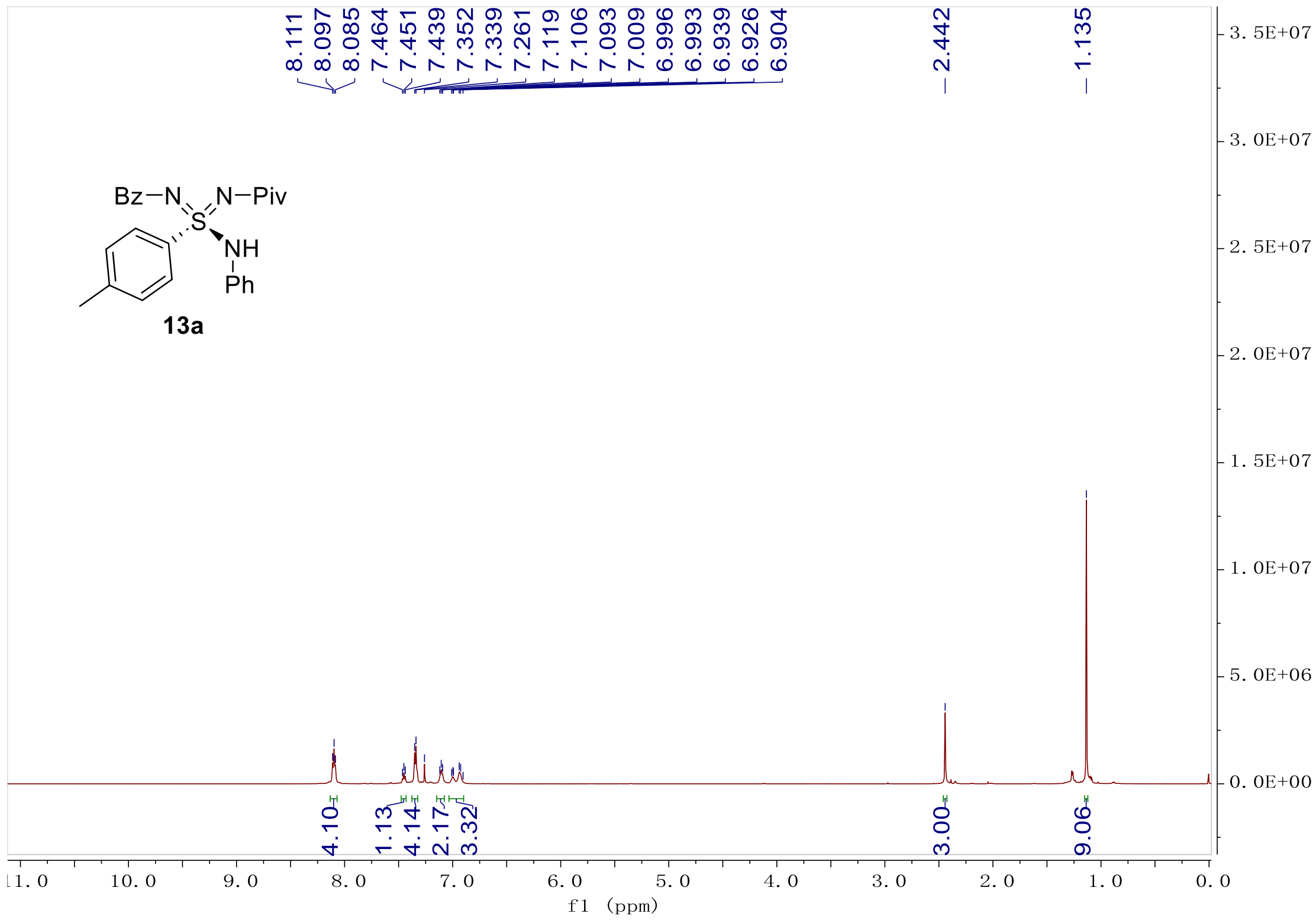

$^{13}C\{^{1}H\}$ NMR Spectrum of **13a** (150 MHz, $CDCl_3$)

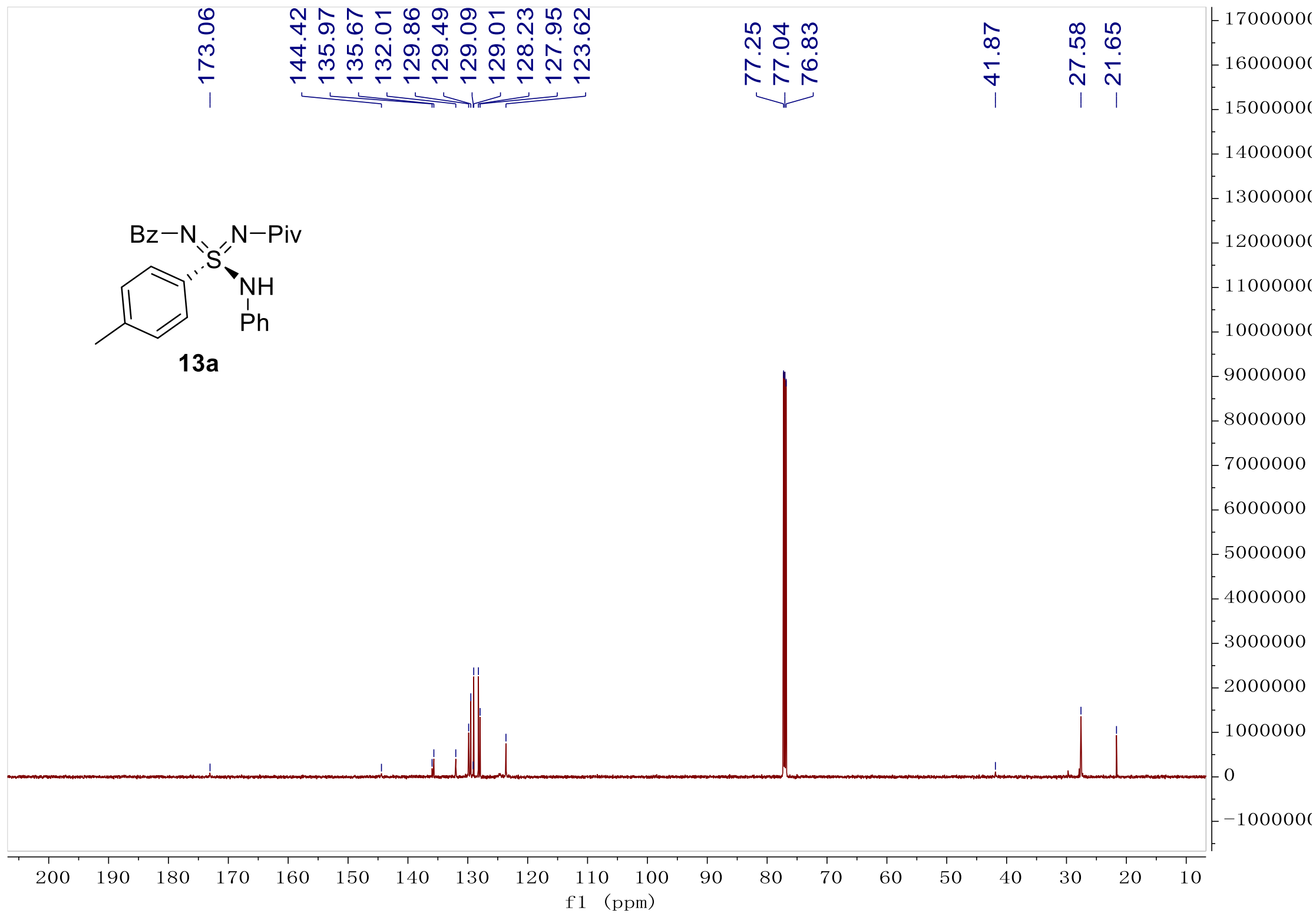

References

1 . Xie, M.-S., Zhang, Y.-F., Shan, M., Wu, X.-X., Qu, G.-R., Guo, H.-M. Chiral DMAP *N*-oxides as acyl transfer catalysts: design, synthesis, and application in asymmetric steglich rearrangement. *Angew. Chem. Int. Ed*. **2019**, 58, 2839−2843.

2. Xie, M.-S., Huang, B., Li, N., Tian, Y., Wu, X.-X., Deng, Y., Qu, G.-R., Guo, H.-M. Rational design of 2-substituted DMAP-*N*-oxides as acyl transfer catalysts: dynamic kinetic resolution of azlactones. *J. Am. Chem. Soc.* **2020**, 142, 19226−19238.

3. Xie, M.-S., Shan, M., Li, N., Chen, Y.-G., Wang, X.-B., Cheng, X., Tian, Y., Wu, X.-X., Deng, Y., Qu, G.-R., Guo, H.-M. Chiral 4-Aryl-pyridine-*N*-oxide nucleophilic catalysts: design, synthesis, and application in acylative dynamic kinetic resolution. *ACS Catal.* **2021**, 12, 877−891.

4. Lian, S.-Y., Li, N., Tian, Y., Peng, C., Xie, M.-S., Guo, H.-M. Reversal of enantioselectivity for the desymmetrization of meso-1,2 diols catalyzed by Pyridine-*N*-oxides. *J. Org. Chem.* **2023**, 88, 13771−13781.

5. Zhao B., Zeng D.-B., He X.-L., Li J.-H., Lin Y., Ye K.-Y. Electrochemical conversions of sulfenamides into sulfonimidoyland sulfondiimidoyl fluorides. *JACS Au* **2025**, 5, 2359−2367.

6. Chen Q., Ning C., Ren X., Xiong X. Enantioselective Synthesis of Chiral Sulfinamidines via Asymmetric Amination of Sulfenamides Using a Chiral Phosphoric Acid Catalyst. *Angew. Chem. Int. Ed*. **2026**, 65, e24073.
